# Health postservice readiness and use of preventive and curative services for suspected childhood pneumonia in Ethiopia: a cross-sectional study

**DOI:** 10.1136/bmjopen-2021-058055

**Published:** 2022-04-26

**Authors:** Amare Tariku, Yemane Berhane, Alemayehu Worku, Gashaw Andargie Biks, Lars Åke Persson, Yemisrach Behailu Okwaraji

**Affiliations:** 1 Department of Human Nutrition, Institute of Public Health, College of Medicine and Health Sciences, University of Gondar, Gondar, Amhara Region, Ethiopia; 2 Department of Epidemiology and Biostatistics and Department of Reproductive Health and Population, Addis Continental Institute of Public Health, Addis Ababa, Ethiopia; 3 School of Public Health, Addis Ababa University, Addis Ababa, Ethiopia; 4 Department of Health System and Policy, Institute of Public Health, College of Medicine and Health Sciences, University of Gondar, Gondar, Ethiopia; 5 London School of Hygiene and Tropical Medicine, London, UK; 6 Ethiopian Public Health Institute, Addis Ababa, Ethiopia

**Keywords:** public health, community child health, respiratory infections, health policy

## Abstract

**Objective:**

Pneumonia is the single-leading cause of infectious disease deaths in children under-5. Despite this challenge, the utilisation of preventive and curative child health services remains low in Ethiopia. We investigated the association between health post service readiness and caregivers’ awareness of pneumonia services, care-seeking and utilisation of pneumonia-relevant immunisation in four Ethiopian regions.

**Design and setting:**

This cross-sectional study was conducted in 52 districts of four regions of Ethiopia from December 2018 to February 2019. The health posts preparedness for sick child care was assessed using the WHO Health Service Availability and Readiness Assessment tool. Multilevel analyses were employed to examine the associations between health post readiness and household-level awareness and utilisation of services.

**Participants:**

We included 165 health posts, 274 health extension workers (community health workers) and 4729 caregivers with 5787 children 2–59 months.

**Outcome measures:**

Awareness of pneumonia treatment, care-seeking behaviour and coverage of pentavalent-3 immunisation.

**Results:**

Only 62.8% of health posts were ready to provide sick child care services. One-quarter of caregivers were aware of pneumonia services, and 56.8% sought an appropriate care provider for suspected pneumonia. Nearly half (49.3%) of children (12–23 months) had received pentavalent-3 immunisation. General health post readiness was not associated with caregivers’ awareness of pneumonia treatment (adjusted OR, AOR 0.9, 95% CI 0.7 to 1.1) and utilisation of pentavalent-3 immunisation (AOR=1.2, 95% CI 0.8 to 1.6), but negatively associated with care-seeking for childhood illnesses (AOR=0.6, 95% CI 0.4 to 0.8).

**Conclusion:**

We found no association between facility readiness and awareness or utilisation of child health services. There were significant deficiencies in health post preparedness for services. Caregivers had low awareness and utilisation of pneumonia-related services. The results underline the importance of enhancing facility preparedness, providing high-quality care and intensifying demand generation efforts to prevent and treat pneumonia.

**Strengths and limitations of this study**

- This is the first study, which assessed the association between first-level healthcare facility readiness for services and caregivers’ awareness and utilisation of pneumonia-relevant preventive and curative services in four of the most populous Ethiopian regions.
- Facility preparedness was assessed using the WHO Health Service Availability and Readiness Assessment tool to generate objective and reliable information that is comparable across or within countries.
- We ascertained information on the utilisation of pneumonia-related preventive and curative services with Demographic and Health Surveys methods, assumed to reduce recall bias.
- The study covered pneumonia services and service readiness of health posts in 52 districts of four Ethiopian regions and findings may not be representative of other geographic areas and health system levels.

## Introduction

Worldwide, pneumonia is responsible for one-fifth of all under-5 deaths.^1^ Nearly half (49%) of these deaths occur in four low-income and middle-income countries, including Ethiopia.^2 3^ There are effective preventive measures, including immunisation and antibiotics that can prevent or treat most cases of pneumonia. Despite these resources, children in low-income countries continue to suffer and die from pneumonia due to lack of access to or availability of these services.^4^

The WHO and UNICEF introduced the integrated Community Case Management (iCCM) strategy in 2004 to increase access and quality of services for childhood pneumonia, diarrhoea and malaria. Effective implementation of this strategy requires uninterrupted stock of drugs and supplies, trained community health workers, and community awareness of these services.^5 6^ Nevertheless, studies in low-income countries reveal that less than one-fifth of sick children were brought to relevant health services for suspected pneumonia.^7 8^ This low level of care-seeking has, among other things, been attributed to the poor quality of health services.^9^ A majority of facilities in these settings have reportedly not had essential drugs, supplies and trained community health workers.^10–13^ The general service readiness index for sick child care has varied between 19% and 69% in reports from sub-Saharan African countries.^11 13^ The readiness has been lower in rural areas and at the lowest primary healthcare level.^14–17^ In Ethiopia, earlier reports have shown a lack of iCCM drugs and supplies at health posts.^9 18^

Inadequate preparedness and low service quality at the primary healthcare level reduce parents’ trust and utilisation of health services.^11 19–21^ In Ethiopia, the low utilisation of iCCM services was also attributed to absent supervision and refresher training of health extension workers.^22–27^ We have earlier shown that a substantial proportion of caregivers were not aware of pneumonia-related health services and, therefore, less likely to seek care when their children got sick or get their children immunised.^28 29^ The low utilisation of iCCM services has also been attributed to the lack of readiness of health posts to care for sick children.^19 30 31^ Thus, there are reasons to investigate the primary-level health facility preparedness to provide child health services and whether this is associated with the coverage of pneumonia-related preventive and treatment services. In Ethiopia, under-5 mortality was reported to be 55 per 1000 live births in the 2019 Demographic and Health Survey. Although reduced in recent decades, the persistently high level and continued pneumonia deaths call for intensified efforts to prevent these preventable deaths.^28 32^ The Ethiopian Ministry of Health in collaboration with non-governmental organisations implemented a complex community-based intervention labelled Optimising the Health Extension Programme (OHEP) in four of the most populous regions. The OHEP aimed to contribute to reductions in under-5 child mortality through enhancing utilisation of iCCM and community-based newborn care services.^33^ This study was part of the evaluation of OHEP intervention. We aimed to examine the association between the health post readiness to serve and caregivers’ awareness of pneumonia-related services, care-seeking for sick children, and whether their 12–23 months old children had got three pentavalent vaccinations.

## Methods

### Study setting and design

The Ethiopian primary healthcare system typically consists of a primary hospital, a health centre and five satellite health posts. A health post is the lowest service delivery point staffed by two health extension workers serving around 5000 rural residents. Since 2003, Ethiopia has implemented the health extension programme to achieve universal coverage of primary healthcare for the rural population. This national programme is implemented by health extension workers, and they provide basic promotive, preventive and curative services through outreach and health post-based approaches. In 2010, after a change in policy that allowed the health extension workers to treat child pneumonia, the Ethiopian Ministry of Health and partners initiated the implementation of iCCM of childhood illnesses as part of the health extension programme. Under the iCCM programme, the health extension workers examine, classify and treat pneumonia.^34 35^

The OHEP intervention had three components, that is, community engagement activities, capacity building of health extension workers and women’s development group leaders, and strengthening of the district health services’ ownership and accountability of the primary newborn and child health services. The intervention was conducted in 26 intervention districts with 26 comparison districts spread in four regions of Ethiopia, namely Tigray, Amhara, Oromia and Southern Nations, Nationalities and Peoples Regions. The intervention started in 2016 and was completed in 2018. For the evaluation, baseline and end line surveys were performed. This paper reports a secondary analysis of end line cross-sectional data.^33^

### Participants

This study included all caregivers and children aged 2–59 months, who resided in 52 study districts. It also includes all health posts with one or two health extension workers per health post serving these families.

### Sampling

This study was based on secondary analysis of data from the endline survey that was part of the evaluation of the OHEP intervention. Sample size for the end line survey was estimated to measure changes in care-seeking and appropriate treatment for childhood illnesses between intervention and comparison areas at baseline and endline. Assumptions considered for the sample size calculation for the main OHEP evaluation^36^ were 80% power to detect differences of 15% for the reported level of care-seeking (55%) and 20% for appropriate treatment for childhood illnesses (47%) at the baseline, design effect of 1.001% and 90% completeness. Accordingly, a sample size of 12 000 households was obtained. With this number of households, 6532 children below the age of 5 years were expected to be reached by the survey, of whom 368 were assumed to have any illnesses and 308 to have suspected pneumonia within 2 weeks before the survey.

The parent study used a sampling frame generated based on the 2007 Ethiopian Central Statistical Agency housing and population survey. Two hundred enumeration areas, that is, clusters, were selected from 52 study districts with probability proportional to size. A two-staged cluster sampling followed by systematic sampling to select 60 representative households from each study cluster. All caregivers of children aged 2–59 months living in the selected households were interviewed. Moreover, all health posts and one or two health extension workers from each health post serving the population of the study clusters were included.^37^

### Data collection

Data were collected using structured and pre-tested interviewer-administered questionnaires through an electronic data collection software (CSpro survey software). The questionnaires were translated into local languages (Amharic, Tigrigna and Oromiffa) and back-translated into English. They comprised three main modules; household, healthcare provider and health facility modules (see online supplemental files 1–3). Data collectors and supervisors, who had bachelor’s degree or above, were trained for 2 weeks on data collection techniques, procedures, quality assurance and ethical considerations of the study. Further detailed information about data collection and quality control is available in the published study protocol.^33^

10.1136/bmjopen-2021-058055.supp1 Supplementary data

10.1136/bmjopen-2021-058055.supp2 Supplementary data

10.1136/bmjopen-2021-058055.supp3 Supplementary data

### Outcomes

The outcomes of this study are caregivers’ awareness of pneumonia treatment, care-seeking behaviour and coverage of pentavalent-3 immunisation as defined in the Demographic and Health Surveys.^28^ The awareness of availability of pneumonia treatment was calculated as the proportion of caregivers who had heard messages regarding pneumonia treatment. Suspected pneumonia was ascertained by asking the caregiver if the child had cough combined with either fast or difficult breathing due to chest problems within 2 weeks before the survey. Care-seeking was defined as children with suspected pneumonia for whom advice or treatment was sought from an appropriate care provider, that is, either government or private providers. The vaccination status of children aged 12–23 months was primarily assessed by reviewing immunisation cards. When cards were not available at home, the caregivers were requested to report the type of vaccines their children had received. Hence, coverage of pentavalent vaccination was estimated as the proportion of children 12–23 months who had received three doses of pentavalent vaccine.

### Health postreadiness

The readiness of health posts for sick child care was assessed using the WHO Service Availability and Readiness Assessment tool.^38^ Using 23 tracer items, the preparedness of facilities was shown in five domains or indices, that is, basic amenities, basic equipment, standard precaution equipment for infection prevention, diagnostic capacity and essential medicines. The mean availability of items across the four domains of readiness was estimated by assigning equal weight to each of the items, and was expressed as a proportion. Health posts’ diagnostic capacity was shown as the proportion of facilities having rapid diagnostic test for malaria. The general service readiness was calculated as the average of percentages depicting mean availability of tracer items in five indices.^38^ The availability of vaccination card at the health posts was also estimated. The number of health extension workers working at the health post and the percentage of these workers trained in iCCM and who had received supportive supervision during 6 months before the survey were also calculated. The health post demand generation activities were recorded as the proportion of health posts showing opening days or that used community forums to deliver maternal and child health education.

### Analyses

The household socioeconomic status was constructed through principal component analysis of household assets, ownership of house, livestock, agricultural land and access to utilities and infrastructures. The factor scores were summed and ranked into quintiles from the poorest to the least poor.

The study linked the household, health facility and care provider information. Our analysis was based on linked samples for outcome variables, that is, caregivers’ awareness of pneumonia treatment (n=4934), care-seeking when the child was sick (n=613) and vaccination of 12–23 months old children with a third dose of pentavalent immunisation (n=860). Care-seeking was assessed for all childhood illness episodes, including symptoms of suspected pneumonia as reported by caregivers for the 2 weeks prior to the survey. The effect of clustering on three of the study outcomes was examined by estimating intracluster correlation coefficients (ICC). A multilevel binary logistic regression model was fitted to examine the association between health post readiness and household level awareness, care-seeking and utilisation of three doses of pentavalent vaccinations. We checked for potential household-level confounders. The fitness of the model was checked through Likelihood Ratio Test. Data were analysed using Stata V.14.

### Patient and public involvement

Patients or the public were not involved in the design or conduct or reporting or dissemination plans of this research.

## Results

### Characteristics of caregivers and children

A total of 10 785 households from 181 study clusters, 165 health posts and 274 health extension workers were included in the survey. A total of 4729 eligible caregivers with 5787 children aged 2–59 months were included in the analyses. A majority of the caregivers had no schooling (59.4%) and were currently married (88.6%). About two-thirds (64.0%) of caregivers were able to access the nearest health facility within 30 min of walk from their home (table 1).

**Table 1:** Sociodemographic characteristics of caregivers and children aged 2–59 months in four regions of Ethiopia, December 2018 to February 2019

Characteristics	Frequency	Percentage
Caregivers’ characteristics (n=4729)		
Age		
<25	885	18.7
25–29	1281	27.1
30–34	1038	22
35–39	867	18.3
≥40	658	13.9
Marital status		
Currently married	4067	88.6
Unmarried	521	11.4
Education		
No schooling	2810	59.4
Schooling	1919	40.6
No of children under-5		
1	3487	73.7
2	1148	24.3
3+	94	2
Socioeconomic quintiles		
Q1 (poorest)	1024	21.7
Q2	982	20.8
Q3	874	18.5
Q3	895	18.9
Q5 (least poor)	954	20.1
Walking distance from household to nearest health facility (n=3918)		
≤30 min	2507	64
>30 min	1411	36
Child (2–59 months) characteristics (n=5787)		
Sex		
Male	2961	51.2
Female	2826	48.8
Age		
2–11 months	959	16.6
12–23 months	992	17.1
24–35 months	1114	19.3
36–59 months	2722	47

### Characteristics of health posts and health extension workers

The median number of households served by the health post was 1181. The majority (85.1%) of the health posts were functionally open for 5 days or more per week. More than 1/10 (12.7%) of the health posts were served by only one health extension worker. Most (78.8%) of the health extension workers were trained in the iCCM of childhood illnesses. More than three-fourth (78.8%) had received supportive supervision within 6 months preceding the survey. Three-quarters used community forums or meetings to deliver maternal and child health education within 3 months prior to the survey (table 2).

**Table 2:** Characteristics of health posts in four regions of Ethiopia, December 2018 to February 2019

Characteristics	Frequency	Percentage
Health posts catchment area population (n=165)		
No of households, median (IQR)	1181 (841–1572)	
No of children under 5 years, median (IQR)	780 (493–1065)	
Health extension workers’ characteristics (274)		
No of health extension workers per health post		
One	35	12.7
Two	142	51.8
Three and above	97	35.4
Health postopening days per week		
1–4 days	41	14.9
5–7 days	233	85.1
Posted health postopening days	54	19.7
Trained for iCCM of childhood illnesses	216	78.8
Received supportive supervision in the last 6 months	216	78.8
Participated in Performance Review and Clinical Mentorship meetings	126	46.0
Used community forums to deliver maternal and child health education	205	74.8

iCCM, integrated Community Case Management.

### Health postpreparedness to deliver sick child care services

**Figure 1:**
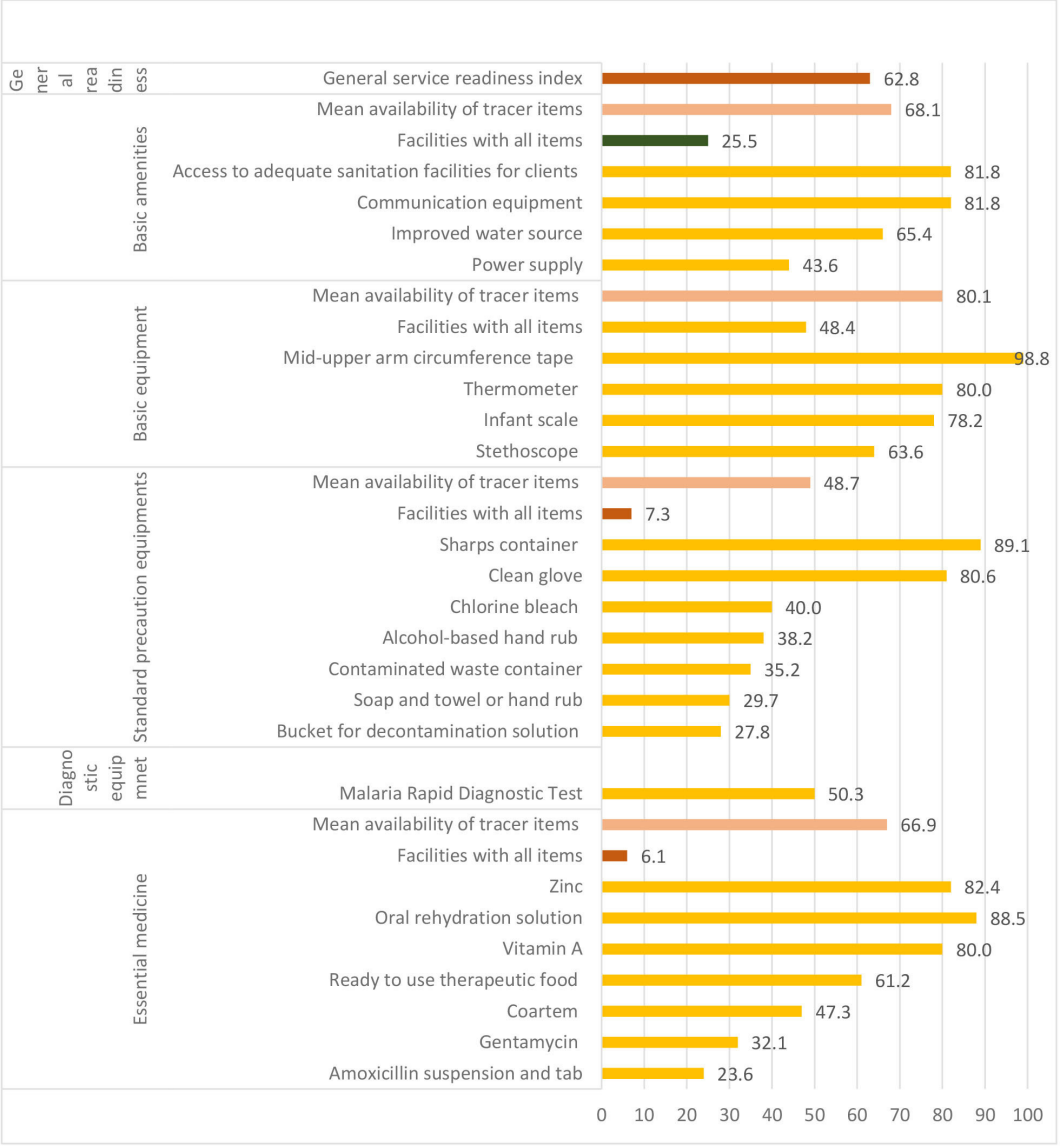
Percentage of health posts (n=165) with drugs and supplies to deliver pneumonia-related and other sick child care services in four regions of Ethiopia, December 2018 to February 2019.

The general service readiness of health posts (n=165) to deliver sick child health services was estimated at 62.8%. Half of the health posts had rapid diagnostic test for malaria, and the mean availability of essential medicines was 66.9%. Relatively higher mean availability (80.1%) of tracer items was shown for basic equipment, while the lowest (48.7%) was for availability of standard precaution items. Very few health posts had all essential medicines and standard precaution equipment. Most of the health posts (84.2%) had vaccination cards (figure 1).

### Awareness of treatment, actual care-seeking and utilisation of preventive immunisation

During the 2 weeks before the survey 704 (12.3%) of the children had any illnesses. Of these, 44 children had symptoms and signs of suspected pneumonia. Only one in five (23.9%) caregivers were aware of the availability of pneumonia treatment services. Healthcare was sought for one-third (36.1%) of children with any illnesses and for 56.8% of children with suspected pneumonia. Almost half (49.3%) of children aged 12–23 months had received three doses of pentavalent vaccinations (table 3).

**Table 3:** Childhood illness and care utilisation by child demographic characteristics in four regions of Ethiopia, December 2018 to February 2019

Service utilisation	Frequency	Percentage
Children with any illnesses		
Yes	704	12.3
No	5021	87.7
Childhood illnesses by sex (n=704)		
Male	362	51.4
Female	342	48.6
Childhood illnesses by age (n=704)		
2–11 months	121	17.2
12–23 months	147	20.9
24–35 months	152	21.6
36–59 months	284	40.3
Child with suspected pneumonia		
Yes	44	0.8
No	5743	99.2
Child with suspected pneumonia treated with antibiotics (n=44)		
Yes	17	38.6
No	27	61.4
Full pentavalent vaccination by sex (n=489)		
Male	258	52.8
Female	231	47.2

### Association between health postreadiness and awareness and utilisation of pneumonia care

The ICC indicated that the study outcomes, that is, awareness of pneumonia treatment (ICC 0.29, 95% CI 0.24 to 0.36), care-seeking behaviour (ICC 0.16, 95% CI 0.10 to 0.27) and pentavalent vaccination (ICC 0.28, 95% CI 0.20, 0.38) significantly varied with level of clusters. While checking for the household-level confounders, we found that none of the household, caregiver and child characteristics were associated with the outcomes and main exposure variables, that is, the five health postreadiness indices. But regardless of the statistical screening criteria (p<0.2), analyses were adjusted for maternal age, education and area of residence (intervention-comparison area) variables.

Analyses tested the association of general health post readiness index with study outcomes were adjusted for health extension workers’ iCCM training, supportive supervision, participation at performance review and clinical mentorship meeting, home vising, use of community forums, opening days and number of staff at health post, mothers’ age, education and area of residence (intervention-comparison area) variables. The adjusted multilevel analyses revealed that general health post readiness was not associated with caregivers’ awareness of availability of pneumonia treatment (adjusted OR, AOR 0.9, 95% CI 0.7 to 1.1) or utilisation of pentavalent-3 immunisation (AOR 1.2, 95% CI 0.8 to 1.6). The general health postreadiness was negatively associated with care-seeking for childhood illnesses (AOR 0.6, 95% CI 0.4 to 0.8)] (table 4).

**Table 4:** General health postreadiness and caregivers’ awareness, care-seeking and utilisation of pentavalent-3 immunisation in four regions of Ethiopia, December 2018 to February 2019

Awareness and utilisation	General readiness	
	Crude OR (95% CI)	Adjusted OR (95% CI)
Awareness of treatment service (N=4934)*	0.9 (0.7 to 1.1)	0.9 (0.7 to 1.1)
Care-seeking (N=613)*	0.7 (0.5 to 0.9)	0.6 (0.4 to 0.8)
Pentavalent-3 (N=860)*	1.2 (0.9 to 1.6)	1.2 (0.8 to 1.6)

*Analyses adjusted for health extension workers’ iCCM training, supportive supervision, participation at performance review and clinical mentorship meeting, home vising, use of community forums, opening days and number of staff at health post, mothers’ age, education and area of residence.

iCCM, integrated Community Case Management.

As illustrated in table 5, none of the health post readiness indices were associated with caregivers’ awareness of availability of pneumonia treatment and care-seeking for childhood illnesses. Only availability of standard precaution equipment for infection prevention was positively associated with utilisation of pentavalent-3 immunisation (AOR 4.5, 95% CI 1.6 to 12.8). Home visiting by the health extension workers was associated with higher odds for caregivers’ awareness of availability of pneumonia treatment (AOR 2.9, 95% CI 2.3 to 3.6).

**Table 5:** Health postpreparedness indices associated with caregivers’ awareness, care-seeking and utilisation of three doses of pentavalent vaccines in four regions of Ethiopia, December 2018 to February 2019

Characteristics	Awareness of treatment service (N=4934)				Care-seeking (N=613)				Pentavalent-3 (N=860)			
	Aware	Unaware	Crude OR (95% CI)	Adjusted OR (95% CI)	Sought care	Didn’t seek care	Crude OR (95% CI)	Adjusted OR (95% CI)	Vaccinated	Not vaccinated	Crude OR (95% CI)	Adjusted OR (95% CI)
Basic amenities												
All not available	923	2867	1.0	1.0	161	270	1.0	1.0	325	337	1.0	1.0
All available	256	888	0.9 (0.6 to 1.5)	0.9 (0.6 to 1.5)	49	133	0.7 (0.4 to 1.1)	0.7 (0.4 to 1.2)	98	100	0.9 (0.6 to 1.8)	0.9 (0.6 to 1.8)
Basic equipment												
All not available	590	2021	1.0	1.0	117	202	1.0	1.0	219	224	1.0	1.0
All available	589	1734	1.3 (0.9 to 1.9)	1.2 (0.8 to 1.9)	93	201	0.8 (0.5 to 1.3)	0.9 (0.5 to 1.4)	204	213	0.9 (0.6 to 1.6)	0.8 (0.5 to 1.4)
Standard precaution equipment for infection prevention												
All not available	1091	3555	1.0	1.0	202	379	1.0	1.0	386	414	1.0	1.0
All available	88	200	1.9 (0.9 to 3.9)	1.9 (0.9 to 4.2)	8	24	0.6 (0.2 to 1.7)	0.8 (0.3 to 2.1)	37	23	2.3 (0.9 to 5.6)	4.5 (1.6 to 12.8)
Rapid diagnostic test for malaria												
No available	597	1858	1.0	1.0	NA	NA	NA	NA	NA	NA	NA	NA
Available	582	1897	0.8 (0.5 to 1.2)	0.7 (0.4 to 1.1)	NA	NA	NA	NA	NA	NA	NA	NA
Essential medicine												
First tercile	354	1025	1.0	1.0	58	98	1	1	108	126	1.0	1.0
Second tercile	396	1355	0.9 (0.5 to 1.4)	0.9 (0.5 to 1.4)	76	142	0.8 (0.4 to 1.4)	0.8 (0.4 to 1.5)	129	145	0.9 (0.5 to 1.8)	0.8 (0.4 to 1.6)
Third tercile	429	1375	0.8 (0.5 to 1.3)	0.8 (0.5 to 1.4)	76	163	0.7 (0.4 to 1.3)	0.7 (0.4 to 1.4)	186	166	1.3 (0.7 to 2.3)	1.1 (0.6 to 2.1)
No of health extension workers per health post												
One	226	784	1.0	1.0	47	71	1.0	1.0	73	68	1.0	1.0
Two and above	953	2971	1.2 (0.7 to 1.9)	1.2 (0.7 to 1.9)	163	332	0.8 (0.4 to 1.4)	0.8 (0.5 to 1.5)	350	369	0.9 (0.5 to 1.6)	0.7 (0.4 to 1.4)
No of health postopening days												
Less than 5 days	209	582	1.0	1.0	43	71	1.0	1.0	61	67	1.0	1.0
Five days and above	970	3173	0.8 (0.5 to 1.3)	0.7 (0.4 to 1.2)	167	332	0.9 (0.5 to 1.5)	0.7 (0.4 to 1.4)	362	370	1.3 (0.7 to 2.5)	1.4 (0.7 to 2.8)
Health extension workers used community forum												
No	297	1003	1.0	1.0	70	139	1.0	1.0	113	131	1.0	1.0
Yes	882	2752	1.2 (0.8 to 1.8)	1.3 (0.8 to 1.9)	140	264	1.0 (0.6 to 1.6)	0.9 (0.6 to 1.5)	310	306	1.3 (0.8 to 2.2)	1.3 (0.7 to 2.2)
Health extension workers received supervision												
No	NA	NA	NA	NA	44	105	1.0	1.0	60	68	1.0	1.0
Yes	NA	NA	NA	NA	166	298	1.3 (0.8 to 2.3)	1.5 (0.8 to 2.6)	363	369	1.4 (0.7 to 2.5)	1.1 (0.5 to 2.2)
Health extension workers received iCCM training												
Did not received training	NA	NA	NA	NA	40	74	1.0	1.0	NA	NA	NA	NA
Received training	NA	NA	NA	NA	170	329	0.9 (0.5 to 1.6)	0.9 (0.5 to 1.7)	NA	NA	NA	NA
Health extension workers participated at Performance Review and Clinical Mentorship meeting												
No	NA	NA	NA	NA	123	252	1.0	1.0	232	252	1.0	1.0
Yes	NA	NA	NA	NA	87	151	1.2 (0.8 to 1.9)	1.2 (0.7 to 2.0)	191	185	1.2 (0.7 to 1.9)	0.9 (0.5 to 1.5)
Home vising												
No	934	3445	1	1	175	357	1	1	372	386	1.0	1.0
Yes	244	309	2.9 (2.4 to 3.7)	2.9 (2.3 to 3.6)	35	46	1.6 (0.9 to 2.6)	1.5 (0.9 to 2.5)	51	38	1.6 (0.9 to 2.7)	1.6 (0.9 to 2.7)

iCCM, integrated Community Case Management; NA, not available.

## Discussion

Overall, this study showed insufficient health post service preparedness and low household awareness and utilisation of preventive and curative services for childhood suspected pneumonia. There was no consistent association between facility preparedness and utilisation of preventive and curative services.

### Strengths and limitations

With an attempt of narrowing the existing scarcity of evidence, our study examined the association between health post readiness and household-level awareness and utilisation of pneumonia relevant health services in a wider geographic area. Readiness of health posts was measured using the standard WHO Health Service Availability and Readiness Assessment tool. This tool is presumed to generate objective and reliable information that is comparable across or within countries. We pretested and adopted the tool to the local context and to level of care provided at health posts.^38^ When vaccination cards were not available at home, children’s pentavalent immunisation status was assessed through interviewing the caregivers. Ascertainment of childhood suspected pneumonia and other illnesses was based on the caregivers’ 2 weeks reported symptoms prior to the survey. These ascertainment techniques have been used by the Demographic and Health Surveys, but might anyhow be influenced by recall bias.^28^ Furthermore, we assessed health post service readiness and pneumonia service utilisation in selected districts of four Ethiopian regions. The findings may not be generalisable to other geographical areas and health system levels.

### Health postpreparedness

This study showed that two-thirds of the health posts were ready to provide sick child care, suggesting that the remaining facilities were not able to deliver such services. More or less similar level of structural preparedness of health posts or primary healthcare facilities for pneumonia and other sick child care were previously reported in Ethiopia and from other low-income and middle-income countries.^10 39 40^ Furthermore, health posts or health centres of Ethiopia and other low-income countries were found with low readiness to provide quality care to sick children.^9^ According to the Ethiopia health system, a health centre is structured to support and strengthen five health posts within their catchment areas, hence insufficient preparedness of the surveyed health posts could be explained by scarcity of supplies at health centres.^29 41^ The weak linkage and inadequate support from the health centres or the health system could further cause scarcity of drugs and supplies and unpreparedness of staff to serve at health posts.^22 42^ The lack of readiness at health posts could also be related to the donor-dependent nature of supplies and the health extension workers’ lack of accountability and capacity in supply-chain management.^34 43 44^

Inadequate readiness of health posts in the study setting and other low-income countries indicates a serious challenge to community case management of pneumonia, particularly in the rural or unreached communities, where a majority of preventable deaths occur.^6 28^ Most importantly, only a few of the surveyed health posts had all essential medicines and just half had diagnostic equipment, clearly indicating their limitations in providing effective pneumonia or sick child treatment.^5 6^ Scarcity of essential medicines in Ethiopia and other sub-Saharan African countries results in missed pneumonia treatments at facility level.^13 17 29^ Unavailability of rapid diagnostic tests impairs community health workers’ ability to differentiate suspected pneumonia from malaria in case of symptom overlap, a common clinical problem in African children.^45–47^

### Awareness and utilisation of pneumonia-related health services

Our study revealed low healthcare utilisation for pneumonia-specific preventive and curative services. These levels of service utilisation were lower compared with the reported regional pentavalent-3 immunisation coverage (80%) and care-seeking behaviour (85%) for childhood suspected pneumonia and other illnesses in sub-Saharan African Countries.^48 49^ Community awareness of illness and sick child care is a prerequisite for timely utilisation of health services.^50^ We found that less than a fifth of caregivers were aware of the availability of pneumonia treatment services, and this might partly explain the observed poor utilisation of pneumonia-related health services in the study setting.^51 52^ The reported low care utilisation could also be explained by inadequate readiness or service quality of the primary healthcare facilities for pneumonia-related preventive and treatment services.^39 53^ The OHEP evaluation studies have revealed a low quality of sick child care services provided at the primary healthcare facilities, and caregivers of children have also mentioned this as a key barrier to seek care at health posts.^54–56^ A study in 22 African countries noted a low level of community trust in public health facilities as an important reason to the low coverage of child vaccination.^57^

### Association between health post preparedness and utilisation of preventive immunisation and care-seeking behaviour

Earlier studies have shown a positive association between health facility readiness and utilisation of first-level sick child care.^11 21^ We did not find any consistent pattern of relationship between facility readiness indices and utilisation of services. There was a positive association between the availability of standard precaution equipment for infection prevention and utilisation of pentavalent-3 immunisation. However, the general health post readiness had no association with awareness and coverage of pentavalent-3 immunisation, but a negative association with care-seeking for childhood suspected pneumonia and other illnesses. Studies in Haiti and Ethiopia have shown absence of association between readiness of primary healthcare facilities for sick child care and caregivers’ utilisation and satisfaction to the respective services.^40 58^ The lack of consistent positive association may be linked to the dominating low level of health post preparedness. The community’s value to quality of healthcare service is a key driver of their decision to seek care, and this may subserve the lack of consistent association between facility readiness and utilisation of pneumonia services. Irrespective of the readiness of health posts, caregivers’ preconceived lack of trust to quality of primary child health services could motivate the use of other facilities with perceived higher service quality.^54^ Equipping healthcare facilities with relevant equipment is a prerequisite to enhance the quality of iCCM services.^6^ Hence, the reported lack of association of health post readiness with awareness and utilisation of pneumonia treatment services suggests that simply equipping facilities with necessary supplies is not a guarantee to reach the intended level of community awareness and utilisation of health services. Health facility strengthening efforts should go along with implementation of awareness creation and demand generation interventions to increase the community awareness, trust and utilisation of pneumonia related and other child health services.^50^ Our previous study showed higher parents’ care-seeking for childhood suspected pneumonia among those with improved awareness of treatment service.^59^ The current study also illustrated that parents’ awareness of pneumonia treatment was higher when health extension workers had visited at home. Home visiting by the community health workers is a vital strategy to promote child health and enhance awareness and utilisation of health services.^50 60^ A substudy of the same project also showed that awareness creation and delivery of preventive child health interventions (such as immunisations) were the main components of outreach services delivered by the health extension workers.^54^

### Relation of findings with already published OHEP evaluation studies

This study was part of the end line evaluation of the OHEP intervention. The findings showed sizeable gaps in structural readiness of health posts for sick child care. Results of our published baseline study also illustrated the scarcity of essential drugs and other supplies at the health posts.^29^ Substudies of the same project investigated quality of sick child care, showing low clinical performance of the health extension workers to identify and treat childhood suspected pneumonia and other illnesses.^55 56^ A qualitative study noted lack of caregivers’ trust in the health extension workers’ clinical competency to manage sick children and a low availability of essential drugs, diagnostics and other supplies at health posts. The low quality of sick child care at health posts is a barrier to use these services.^54^

## Conclusions

This study has shown a low health post readiness for services, and low household awareness and utilisation of pneumonia-relevant preventive and curative services. Parents’ awareness and utilisation of pneumonia-specific preventive and curative services were not consistently associated with the health post readiness. The results underline the critical importance of intensifying the health extension workers’ awareness creation and demand generation efforts in each kebele (the lowest administrative unit in Ethiopia). Enhancing the coverage of home visiting and other awareness creation activities are crucial to boost community awareness and utilisation of pneumonia and other sick child care services. Our findings also underline the pivotal role of improving the availability and quality of pneumonia and other sick child care services to ensure optimal uptake of the services. It is imperative that the district health offices strengthen the linkages within the primary healthcare units to increase the availability of essential medicines and readiness of the health posts for sick child care. The office should also optimise the availability of essential medicines and supplies at health centres that are the suppliers to the satellite health posts. Improving the coverage of regular supportive supervision, performance reviews and clinical mentorship could also help to timely identify and solve gaps in the availability of drug and other supplies at health posts. Community awareness creation and demand generation efforts should simultaneously be accompanied with health facility strengthening strategies.

## Supplementary Material

Reviewer comments

Author's manuscript

## Data Availability

Data are available on reasonable request. The data for this manuscript were primarily collected by the Ethiopian Public Health Institute and London School of Hygiene & Tropical Medicine. Interested researchers may contact the focal person, YBO through email: Yemisrach.Okwaraji@lshtm.ac.uk. All requests will be reviewed by this committee and if granted, data will be shared without any identifiers.

## References

[1] Liu L, Johnson HL, Cousens S, et al. Global, regional, and national causes of child mortality: an updated systematic analysis for 2010 with time trends since 2000. Lancet 2012;379:2151–61. 10.1016/S0140-6736(12)60560-1 22579125

[2] United Nations Inter-agency Group for Child Mortality Estimation (UN IGME). ‘Levels & Trends in Child Mortality: Report 2020, Estimates developed by the United Nations Inter-agency Group for Child Mortality Estimation’. New York: United Nations Children’s Fund, 2020.

[3] McAllister DA, Liu L, Shi T, et al. Global, regional, and national estimates of pneumonia morbidity and mortality in children younger than 5 years between 2000 and 2015: a systematic analysis. Lancet Glob Health 2019;7:e47–57. 10.1016/S2214-109X(18)30408-X 30497986 PMC6293057

[4] Bhutta ZA, Das JK, Walker N, et al. Interventions to address deaths from childhood pneumonia and diarrhoea equitably: what works and at what cost? Lancet 2013;381:1417–29. 10.1016/S0140-6736(13)60648-0 23582723

[5] World Health Organization. Management of pneumonia in community settings: World Health Organization, Department of Child and Adolescent Health and … 2004.

[6] World Health Organization. WHO/UNICEF joint statement integrated community case management (iCCM): an equity-focused strategy to improve access to essential treatment services for children. Geneva and New York, 2012. 10.4269/ajtmh.2012.12-0221 PMC3748523 23136272

[7] Geldsetzer P, Williams TC, Kirolos A, et al. The recognition of and care seeking behaviour for childhood illness in developing countries: a systematic review. PLoS One 2014;9:e93427. 10.1371/journal.pone.0093427 24718483 PMC3981715

[8] Adedokun ST, Yaya S. Factors influencing mothers' health care seeking behaviour for their children: evidence from 31 countries in sub-Saharan Africa. BMC Health Serv Res 2020;20:842. 10.1186/s12913-020-05683-8 32894107 PMC7487813

[9] Macarayan EK, Gage AD, Doubova SV, et al. Assessment of quality of primary care with facility surveys: a descriptive analysis in ten low-income and middle-income countries. Lancet Glob Health 2018;6:e1176–85. 10.1016/S2214-109X(18)30440-6 30322648 PMC6187280

[10] Acharya K B J, Tuladhar S. Client satisfaction and quality of curative services for sick children in Nepal. DHS further analysis reports No. 114. Rockville, Maryland, USA: ICF, 2018.

[11] Liu L, Leslie HH, Joshua M, et al. Exploring the association between sick child healthcare utilisation and health facility quality in Malawi: a cross-sectional study. BMJ Open 2019;9:e029631. 10.1136/bmjopen-2019-029631 PMC6661667 31352421

[12] Shawon MSR, Adhikary G, Ali MW, et al. General service and child immunization-specific readiness assessment of healthcare facilities in two selected divisions in Bangladesh. BMC Health Serv Res 2018;18:39. 10.1186/s12913-018-2858-7 29370842 PMC5784675

[13] Taylor C, Linn A, Wang W, et al. Examination of malaria service utilization and service provision: an analysis of DHS and spa data from Malawi, Senegal, and Tanzania. Malar J 2019;18:258. 10.1186/s12936-019-2892-x 31358005 PMC6664566

[14] Blanas DA, Ndiaye Y, Nichols K, et al. Barriers to community case management of malaria in Saraya, Senegal: training, and supply-chains. Malar J 2013;12:95. 10.1186/1475-2875-12-95 23497188 PMC3607880

[15] Masters SH, Burstein R, DeCenso B, et al. Pharmaceutical availability across levels of care: evidence from facility surveys in Ghana, Kenya, and Uganda. PLoS One 2014;9:e114762. 10.1371/journal.pone.0114762 25500832 PMC4263677

[16] Ssempiira J, Kasirye I, Kissa J, et al. Measuring health facility readiness and its effects on severe malaria outcomes in Uganda. Sci Rep 2018;8:17928. 10.1038/s41598-018-36249-8 30560884 PMC6298957

[17] Bagonza J, Rutebemberwa E, Eckmanns T. What influences availability of medicines for the community management of childhood illnesses in central Uganda? implications for scaling up the integrated community case management programme. BMC Public Health 2015;15:1–8. 26608029 10.1186/s12889-015-2525-4 PMC4660670

[18] International EPHIEFMoHI. Ethiopia service provision assessment plus survey, 2014.

[19] Shaw B, Amouzou A, Miller NP, et al. Determinants of utilization of health extension workers in the context of scale-up of integrated community case management of childhood illnesses in Ethiopia. Am J Trop Med Hyg 2015;93:636–47. 10.4269/ajtmh.14-0660 26195461 PMC4559711

[20] Anselmi L, Lagarde M, Hanson K. Health service availability and health seeking behaviour in resource poor settings: evidence from Mozambique. Health Econ Rev 2015;5:26. 10.1186/s13561-015-0062-6 PMC4556719 26329425

[21] Kuwawenaruwa A, Wyss K, Wiedenmayer K, et al. The effects of medicines availability and stock-outs on household's utilization of healthcare services in Dodoma region, Tanzania. Health Policy Plan 2020;35:323–33. 10.1093/heapol/czz173 31942625 PMC7152726

[22] Kok MC, Kea AZ, Datiko DG, et al. A qualitative assessment of health extension workers' relationships with the community and health sector in Ethiopia: opportunities for enhancing maternal health performance. Hum Resour Health 2015;13:80. 10.1186/s12960-015-0077-4 26423049 PMC4589131

[23] Befekadu A, Yitayal M. Knowledge and practice of health extension workers on drug provision for childhood illness in West Gojjam, Amhara, Northwest Ethiopia. BMC Public Health 2020;20:1–10. 32295548 10.1186/s12889-020-08602-y PMC7160904

[24] Miller NP, Amouzou A, Tafesse M, et al. Integrated community case management of childhood illness in Ethiopia: implementation strength and quality of care. Am J Trop Med Hyg 2014;91:424–34. 10.4269/ajtmh.13-0751 24799369 PMC4125273

[25] Tilahun H, Fekadu B, Abdisa H, et al. Ethiopia's health extension workers use of work time on duty: time and motion study. Health Policy Plan 2017;32:320–8. 10.1093/heapol/czw129 27658649

[26] Datiko DG, Bunte EM, Birrie GB. Community participation and maternal health service utilization: lessons from the health extension programme in rural southern Ethiopia. Journal of Global Health Reports 2019;3.

[27] Mangham-Jefferies L, Mathewos B, Russell J, et al. How do health extension workers in Ethiopia allocate their time? Hum Resour Health 2014;12:61. 10.1186/1478-4491-12-61 25315425 PMC4209031

[28] Central Statistical Agency [Ethiopia] and ICF International. Ethiopia demographic and health survey 2016. Addis Ababa, Ethiopia and Calverton, Maryland, USA: Central Statistical Agency and ICF International, 2017.

[29] Tariku A, Okwaraji YB, Worku A. Prevention and treatment of suspected pneumonia in Ethiopian under‐five children from household to primary care. Acta Paediatrica 2020. 10.1111/apa.15380 PMC7891650 32478446

[30] Berhanu A, Alemayehu M, Daka K, Kassa Daka WB, et al. Utilization of integrated community case management of childhood illnesses at health posts in southern Ethiopia. Pediatric Health Med Ther 2020;11:459. 10.2147/PHMT.S282698 33273879 PMC7705263

[31] Ethiopian Public Health Institute. World Health organization. Ethiopia service availability and readiness assessment 2016 summary report, 2016.

[32] Deribew A, Tessema GA, Deribe K, et al. Trends, causes, and risk factors of mortality among children under 5 in Ethiopia, 1990-2013: findings from the global burden of disease study 2013. Popul Health Metr 2016;14:42. 10.1186/s12963-016-0112-2 27891065 PMC5109762

[33] Berhanu D, Okwaraji YB, Belayneh AB, et al. Protocol for the evaluation of a complex intervention aiming at increased utilisation of primary child health services in Ethiopia: a before and after study in intervention and comparison areas. BMC Health Serv Res 2020;20:1–12. 10.1186/s12913-020-05151-3 PMC7171736 32316969

[34] Ministry of Health. Realizing universal health coverage through primary health care: a roadmap for optimizing the Ethiopian health extension program 2020 – 2035. Addis Ababa, Ethiopia: Ministry of Health, 2020.

[35] Wang, Huihui, Roman Tesfaye, Gandham N. V. Ramana, Chala Tesfaye Chekagn. 2016. Ethiopia health Externsion program: an institutionalized community approach for universal health coverage. world bank studies. Washington, DC: world bank. 10.1596/978-1-4648-0815-9. license: creative commons attribution CC BY3.0 IGO.

[36] Berhanu D, Okwaraji YB, Defar A, et al. Does a complex intervention targeting communities, health facilities and district health managers increase the utilisation of community-based child health services? A before and after study in intervention and comparison areas of Ethiopia. BMJ Open 2020;10:e040868. 10.1136/bmjopen-2020-040868 PMC7493123 32933966

[37] Schellenberg J, LÅ P, Defar A. Endline evaluation of improved integrated community case management and community-based newborn care in Ethiopia: Ethiopian Public Health Institute & London School of Hygien and Tropical Medicine 2018.

[38] Organization WH. Service availability and readiness assessment (SARA): an annual monitoring system for service delivery: reference manual. World Health Organization, 2013.

[39] Hailemariam S, Gebeyehu Y, Loha E, et al. Inadequate management of pneumonia among children in South Ethiopia: findings from descriptive study. BMC Health Serv Res 2019;19:426. 10.1186/s12913-019-4242-7 31242946 PMC6595689

[40] Gage AD, Leslie HH, Bitton A. Does quality influence utilization of primary health care? Evidence from Haiti. Global Health 2018;14:1–9. 29925416 10.1186/s12992-018-0379-0 PMC6011404

[41] de Sousa A, Tiedje KE, Recht J, et al. Community case management of childhood illnesses: policy and implementation in countdown to 2015 countries. Bull World Health Organ 2012;90:183–90. 10.2471/BLT.11.093989 22461713 PMC3314214

[42] Assefa Y, Gelaw YA, Hill PS. Community health extension program of Ethiopia, 2003–2018: successes and challenges toward universal coverage for primary healthcare services. Global Health 2019;15:1–11. 30914055 10.1186/s12992-019-0470-1 PMC6434624

[43] Chandani Y, Noel M, Pomeroy A, et al. Factors affecting availability of essential medicines among community health workers in Ethiopia, Malawi, and Rwanda: solving the last mile puzzle. Am J Trop Med Hyg 2012;87:120. 10.4269/ajtmh.2012.11-0781 23136287 PMC3748512

[44] Ademe BW, Tebeje B, Molla A. Availability and utilization of medical devices in Jimma zone hospitals, Southwest Ethiopia: a case study. BMC Health Serv Res 2016;16:1–10. 27435014 10.1186/s12913-016-1523-2 PMC4952070

[45] Ukwaja KN, Aina OB, Talabi AA. Clinical overlap between malaria and pneumonia: can malaria rapid diagnostic test play a role? J Infect Dev Ctries 2011;5:199–203. 10.3855/jidc.945 21444988

[46] Kyabayinze DJ, Asiimwe C, Nakanjako D. Use of RDTs to improve malaria diagnosis and fever case management at primary health care facilities in Uganda. Malar J 2010;9:1–10. 20624312 10.1186/1475-2875-9-200 PMC2914063

[47] Yeboah-Antwi K, Pilingana P, Macleod WB, et al. Community case management of fever due to malaria and pneumonia in children under five in Zambia: a cluster randomized controlled trial. PLoS Med 2010;7:e1000340. 10.1371/journal.pmed.1000340 20877714 PMC2943441

[48] Ahinkorah BO, Budu E, Seidu A-A, et al. Barriers to healthcare access and healthcare seeking for childhood illnesses among childbearing women in sub-Saharan Africa: a multilevel modelling of demographic and health surveys. PLoS One 2021;16:e0244395. 10.1371/journal.pone.0244395 33556057 PMC7870045

[49] Fenta SM, Biresaw HB, Fentaw KD, et al. Determinants of full childhood immunization among children aged 12-23 months in sub-Saharan Africa: a multilevel analysis using demographic and health survey data. Trop Med Health 2021;49:29. 10.1186/s41182-021-00319-x 33795028 PMC8017626

[50] Miller NP, Bagheri Ardestani F, Wong H, et al. Barriers to the utilization of community-based child and newborn health services in Ethiopia: a scoping review. Health Policy Plan 2021;36:1187-1196. 10.1093/heapol/czab047 33885143 PMC8496769

[51] Sadruddin S, Khan IUH, Bari A, et al. Effect of community mobilization on appropriate care seeking for pneumonia in Haripur, Pakistan. J Glob Health 2015;5:010405. 10.7189/jogh.05.010405 25798232 PMC4357212

[52] Noordam AC, Sharkey AB, Hinssen P, et al. Association between caregivers' knowledge and care seeking behaviour for children with symptoms of pneumonia in six sub-Saharan African countries. BMC Health Serv Res 2017;17:107. 10.1186/s12913-017-2060-3 28153011 PMC5290628

[53] Tadesse T, Gelaw B, Haile Y. Immunization service availability and readiness in primary health care in pastoral and semi-pastoral CGPP Ethiopia implementation districts. Ethiopian Journal of Health Development 2019;33.

[54] Defar A, Alemu K, Tigabu Z, et al. Caregivers' and health extension workers' perceptions and experiences of outreach management of childhood illnesses in Ethiopia: a qualitative study. Int J Environ Res Public Health 2021;18:3816. 10.3390/ijerph18073816 33917415 PMC8038672

[55] Daka DW, Wordofa MA, Woldie M, et al. Quality of clinical assessment and management of sick children by health extension workers in four regions of Ethiopia: a cross-sectional survey. PLoS One 2020;15:e0239361. 10.1371/journal.pone.0239361 32976508 PMC7518593

[56] Getachew T, Mekonnen S, Yitayal M. Health Extension Workers’ diagnostic accurac y for common childhood illnesses in four regions of Ethiopia: a cross-sectional study. Acta Pædiatrica 2019. 10.1111/apa.14888 PMC7154548 31162734

[57] Stoop N, Hirvonen K, Maystadt J-F. Institutional mistrust and child vaccination coverage in Africa. BMJ Glob Health 2021;6:e004595. 10.1136/bmjgh-2020-004595 PMC8094341 33926893

[58] Getachew T, Abebe SM, Yitayal M, et al. Assessing the quality of care in sick child services at health facilities in Ethiopia. BMC Health Serv Res 2020;20:1–12. 10.1186/s12913-020-05444-7 PMC7313135 32576187

[59] Tariku A, Berhane Y, Worku A. Household wealth and utilization of preventive and treatment services for suspected childhood pneumonia in Ethiopia. Submitted for publication at PLOS ONE 2021.

[60] Tripathi A, Kabra SK, Sachdev HPS, et al. Home visits by community health workers to improve identification of serious illness and care seeking in newborns and young infants from low- and middle-income countries. J Perinatol 2016;36 Suppl 1:S74–82. 10.1038/jp.2016.34 27109094 PMC4848742

