# Supplementary material for: Health postservice readiness and use of preventive and curative services for suspected childhood pneumonia in Ethiopia: a cross-sectional study
Source: BMJ Open. 2022 Apr 26;12(4):e058055. doi: 10.1136/bmjopen-2021-058055 (PMC9047705; doi:10.1136/bmjopen-2021-058055)
Supplement: Supplementary data [file bmjopen-2021-058055supp001.pdf]

Dagu End line Household Survey 2018

Module 1 – Household

Section 1.1: Household identifiers				
100h	**Date** (dd/mm/yyyy)	_ _/ _ _/ _ _		
101h	Region	________________		
102h	Zone	________________		
103h	Woreda	_________________		
105h	Kebele	__________________		
106h	Gote	__________________		
107h	Cluster Code	_____		
108h	Household Code	__ __		
109h	Unique household ID<br>(cluster / household code)	__ __ __/ __ __		
110h	GPS Latitude	____ DEG ____ MIN ____ SEC		
111h	GPS Longitude	____ DEG ____ MIN ____ SEC		
113h	Elevation	__ __ __ __		
114h	Interviewer initials	__ __		
115h	***Interviewer***: Have you read him/her the consent form?	1=Yes<br>2=No-one is available		If the answer is No, fill the revisit form
116h	***Interviewer***: Does the respondent agree?	1=Yes<br>2=No. END INTERVIEW		If No Why? State the reason.
112h	Name of household head	_____________________		
117h	What is the ethnic group of the household head?	01=Agew<br>02=Amhara<br>03=Konso<br>04=Burji<br>05=Kore<br>06=Gedeo<br>08=Derashe<br>09=Dawuro	11=Konta<br>12=Ale<br>13=Oromo<br>15=Tigray<br>17=Other, Specify	____

Household Listing with Household Head: Now I would like to ask you about people who are residents of this house. By resident, I mean everyone who has been present for the last 3 months and who normally eat together. *Start with head of the household and older people, then children from oldest to youngest.*

No. of Family members: ***Including yourself how many family members do you have?*** ___________

118h_1<br>**Unique ID**	118h_2<br>**NAME**	118h_3<br>**RELATIONSHIP**	118h_4<br>**SEX**	118h_5<br>**AGE**	118h_E<br>**Eligible**	118h_6<br>**DATE OF BIRTH**	118h_7<br>**MARITAL STATUS**	118h_8<br>**RELIGION**	118h_9<br>**EDUCATION**	118h_10-118h_15<br>**ELIGIBILITY**		
		**What is the relationship of [Name] to the household head?**<br><br>01=Head<br>02=Wife or husband<br>03=Son or daughter<br>04=In-law<br>05=Grandchild<br>06=Parent<br>08=Brother or sister<br>09=Niece or nephew<br>10=Adopted/stepchild<br>11=Other relative<br>12=Not related<br>99=Don't know	**Is [Name] male or female?**<br><br>1=Male<br>2=Female	**How old is [Name]?**<br><br>If less than 1 year write in 0		**What is [Name] date of birth?**<br><br>dd/mm/yyyy	***For all born after 1966:*** **Is [name] married?**<br><br>1=Currently married<br>2= In a union<br>3=Not married or in a union<br>4=Divorced<br>5=Widowed<br>6=N/A	***For all:*** **What religion is [name]?**<br><br>1=Orthodox<br>3=Protestant<br>4=Muslim<br>8=Other	**How many years of formal education have they completed?**<br><br>*Enter number of formal years of education completed*	***CHECK: Is the person a woman 13-49 years?***<br>From 1969 to 2005 (Eth: 1961-1997)<br>1=Yes<br>2=No	***CHECK*:** **Is the person a child under 5 years?**<br><br>1=Yes<br>2=No	***CHECK*:** **If the household has a child under 5 years, is this person the primary caregiver?**<br><br>1=Yes<br>2=No
01	___	___	__	___		__ _/__ _/__ __	_	_	___	_	_	_
02	___	___	__	___		__ _/__ _/__ __	_	_	___	_	_	_
03	___	___	__	___		__ _/__ _/__ __	_	_	___	_	_	_
04	___	___	__	___		__ _/__ _/__ __	_	_	___	_	_	_
05	___	___	__	___		__ _/__ _/__ __	_	_	___	_	_	_
06	___	___	__	___		__ _/__ _/__ __	_	_	___	_	_	_
07	___	___	__	___		__ _/__ _/__ __	_	_	___	_	_	_
08	___	___	__	___		__ _/__ _/__ __	_	_	___	_	_	_
09	___	___	__	___		__ _/__ _/__ __	_	_	___	_	_	_

**Section 1.2: Characteristics of house and assets**			
119h	What is the main material of the roof? *Select one*	1 = Thatch/grass or leaves<br>2 = Iron sheets<br>8 = Other…specify	__
120h	What is the main material of the walls? *Select one*	2 = Natural materials cane, wood, mud<br>4 = Stone with mud<br>5 = Stone with cement/bricks<br>8 = Other	__
121h	What is the main floor material? *Select one*	1 = Natural floor (earth/sand/dung)<br>2 = Rudimentary floor (wood/palm/bamboo)<br>3 = Finished floor (polished wood, cement, carpet)<br>8 = Other	__
122h	What kind of toilet facilities does your household have?	1= No facility/bush/field<br>2= Pit toilet/latrine<br>3= Ventilated improved pit latrine<br>8=Other	__
123h	What is the main source of drinking water for members of your household?<br><br>*Do Not read list*	1=Piped water into dwelling<br>2=Piped water into compound<br>3=Public tap<br>4=Borehole in compound<br>6=Water from protected spring<br>8=Surface water (river/dam/lake etc)<br>9=Hand pump<br>10=Unprotected spring<br>88=Other	__
124h	Do you do anything to the water to make it safer to drink?	1 = Yes<br>2 = No (SKIP TO 126)<br>3 = Don't know (SKIP TO 126)	__
125h	If Yes, what is the main thing you do?<br><br>*Do Not read list*	1 = Let it stand and settle<br>2 = Strain through a cloth<br>3 = Use water filter (ceramic/sand/composite/etc)<br>4 = Boil<br>5 = Solar disinfection<br>6 = Add bleach/chlorine<br>8 = Other	__
126h	What type of fuel does your household mainly use for cooking? *Select one*	1=Electricity<br>4=Charcoal<br>5=Firewood/straw<br>6=Dung<br>8=Other	__
127h	Is the house connected to electricity?	1=Yes<br>2=No	__
**In total, how many the following items owned by residents of this household?** *Enter number of items (zero if None)*			
128h	How many **Wrist watch**?		____
129h	How many **Gold (in grams)?**		____
130h	How many **kerosene lamp/pressure lamps?**		____

131h	How many **Solar lamp?**		____
132h	How many **beds?**		____
134h	How many **Mobile phone?**		____
137h	How many **Radio?**		____
138h	How many **TV**?		____
142h	Do you own this house?	1 = Yes<br>2 = No	__
143h	Does any member of the household own any agricultural land?	1 = Yes<br>2 = No **SKIP TO 145**	
144h	How many hectares of agricultural land do members of this household own?	*Enter total number of hectares*<br>*(If less than 1, Enter in decimals (example 0.5)*<br>*Enter 9999 if hectares are Not known*	____.____
145h	Does this household own any livestock, herds, other farm animals, or poultry?	1 = Yes<br>2 = No **SKIP TO 155**	__
	For each: Enter number. If None, enter 000		
	**How many of the following animals does this household own?**		
146h	How many c**hickens?**		______
147h	How many **goats?**		___
148h	How many **sheep?**		___
149h	How many **donkeys?**		___
150h	How many **horses?**		___
153h	How many **milk cows?**		___
154h	How many **ox (bulls)?**		___
**155h**	At any time in the past 12 months, has anyone come into your house to spray the interior walls of your dwelling against mosquitoes?	1=Yes<br>2=No **SKIP TO 158**<br>9=Don't know **SKIP TO 158**	__
156h	What type of residual spray was done? *(Probe if indoor residual spraying (IRS)*	1=IRS<br>8=Other<br>9=Don't know	__
157h	How many months ago was the house last sprayed?	If less than one month write 00. If don't know write 99	__
158h	How many mosquito nets does your household have?	Write total number Count those in use plus those Not in use. If don't know write 9 If "0" SKIP TO MODULE 2	__

Section 1.3: Household protection from malaria

Write total number of count those in use plus those Not in use (If don't know write 9)

			Net #1	Net #2	Net #3
159h	*Interviewer*: Is the Net 1 observed?	1=Yes<br>2=No	__	__	__
160h	How many months ago did your household obtain the mosquito Net1?	Estimate the number of months ago each net was obtained. If don't know 99	__	__	__
161h	Where did your household get the mosquito Net1 from?	1=Gov. hospital or health centre<br>2=Private hospital or health centre<br>3=HEW/HDA<br>5=Bought from shop/open market<br>7=Mass campaign<br>8=Other<br>9=Don't know	__	__	__
162h	What kind of Net 1 is it?	1= Long-lasting net **SKIP TO 166**<br>2=Factory net<br>8=Other<br>9=Don't know	__	__	__
163h	When you got Net 1 was it already treated with an insecticide to kill or repel mosquitos?	1=Yes<br>2=No<br>9=Don't know	__	__	__
164h	Did anyone sleep under the mosquito Net 1 last night?	1=Yes<br>2=No **If 2 or 9 then, SKIP TO 166**<br>9=Don't know	__	__	__
165h	Who slept under any of the nets last night?	(*record the Person ID from household listing)*	*Enter Person ID*<br>____<br>____<br>____	____<br>____<br>____	___<br>____<br>____
159h 2	***Interviewer***: Is the Net 2observed?	1=Yes<br>2=No	__	__	__
160h 2	How many months ago did your household obtain the mosquito Net2	*Estimate the number of months ago each net was obtained. If don't know 99*	____	____	____
161h 2	Where did your household get the mosquito Net2 from?	1=Gov. hospital or health centre<br>2=Private hospital or health centre<br>3=HEW/HDA<br>5=Bought from shop/open market<br>7=Mass campaign<br>8=Other<br>9=Don't know	__	__	__
162 h2	What kind of Net 2 is it?	1= Long-lasting net **SKIP TO 166**<br>2=Factory net<br>8=Other<br>9=Don't know	__	__	__
163h 2	When you got Net 2 was it already treated with an insecticide to kill or repel mosquitos?	1=Yes<br>2=No<br>9=Don't know	__	__	__
164h 2	Did anyone sleep	1=Yes	__	__	__

	under the mosquito Net 2 last night?	2=No **If 2 or 9 then, SKIP TO 166**<br>9=Don't know			
165h 2	Who slept under any of nets	*(Record the Person ID from household listing)*	*Enter Person ID*<br>____<br>____<br>____	____<br>____<br>____	____<br>____<br>____
159h 3	***Interviewer***: Is the Net 3 observed	1=Yes<br>2=No	__	__	__
160h 3	How many months ago did your household obtain the mosquito Net3?	Estimate the number of months ago each net was obtained. If don't know 99	__	__	__
161h 3	Where did your household get the mosquito Net3 from?	1=Gov. hospital or health centre<br>2=Private hospital or health centre<br>3=HEW/HDA<br>5=Bought from shop/open market<br>7=Mass campaign<br>8=Other<br>9=Don't know	__	__	__
162h 3	What kind of Net 3 is it?	1= Long-lasting net (**SKIP TO 166)**<br>2=Factory net<br>8=Other<br>9=Don't know	__	__	__
163h 3	When you got Net 3 was it already treated with an insecticide to kill or repel mosquitos?	1=Yes<br>2=No<br>9=Don't know	__	__	__
164h 3	Did anyone sleep under the mosquito Net 3 last night?	1=Yes<br>2=No **If 2 or 9 then, SKIP TO 166**<br>9=Don't know	__	__	__
165h 3	Who slept under this mosquito Net 3 last night	*(record the Person ID from household listing)*	*Enter Person ID*<br>____<br>____<br>____	____<br>____<br>____	____<br>____<br>____

Module 2 – Woman aged 13-49 years

Section 2.1: Identification and consent of eligible women			
166h	*Name of women*		___________________
167h	*Woman ID*		_____
168h	Is it possible to interview the woman?	1=Yes **SKIP TO 170**<br>2=No	__
169h	If No, why Not possible to interview the woman?	1=Travelled away<br>2=Sick<br>3=Other<br>4=Currently Not present	__
170h	*Interviewer*: Have you read her the consent form?	1=Yes<br>2= No	__
171h	Does she give her consent to be interviewed?	1=Yes<br>2=No **SKIP TO 179**	__
Thank you for agreeing to participate in this interview. I would like to first ask you about your involvement in the women development army (WDA)			
172h	Do you participate in WDA?	1=Yes<br>2=No **SKIP TO 175**	__
173h	Are you a 1 to 5 WDA leader?	1=Yes<br>2=No	__
174h	Are you a 1 to 30 WDA team leader?	1=Yes<br>2=No	__

Section 2.2: Pregnancy history			
175h	Are you pregnant Now?	1=Yes<br>2=No<br>8=Unsure	__
176h	Have you ever been pregnant before?	1 = Yes<br>2 = No **(End Interview)**	__
177h	Have you ever given birth to a child?	1 = Yes<br>2 = No	__
178h	How many children have you given birth to in total?	Enter number	__
I understand that it is not easy to talk about children who have died, or pregnancies that did not end up not in live birth, but it is important that you tell us about all of them, so that the government can develop programs to improve children's health.			
179h	Have you ever had a pregnancy that's lost before full term?	1=Yes<br>2=No **SKIP TO 181**	__
180h	How many pregnancies have you had that did Not end in a live birth?	Enter number	__
Now would like to ask about the information regarding pregnancies beginning of 2009 (Ethiopian calendar).			
181h	Were you pregnant at the time of 2009 or any time afterwards?	1 = Yes<br>2 = No **(End interview)**	__

Now I would like to record your births since 2009 (Ethiopian Calendar), whether still alive or not, starting with THE MOST RECENT ONE. *Record twins and triplets on separate lines.*

No_of_Preg. How many times were you pregnant at the time of 2009 or any time afterwards?

Is your younger child 2 or under2 years of age?

167h<br>**Woman ID**	181h_a<br>**Pregnancy ID**	181h_b<br>**Outcome of pregnancy**	181h_c<br>**Baby's name**	181h_d<br>**Date of birth/ Pregnancy ended**	181h_e<br>**Born as a twin?**	181h_f<br>**Sex**	181h_g<br>**Still alive?**	181h_h<br>**If still alive, how old in months?**	181h_i<br>**If the child died, when did s/he die?**
		1=Live birth<br>2=Baby born dead, **Skip to 181**<br>3=Lost before full-term **Skip to 181**	*Enter "Not given" if Not given a name*	*Enter 01 for day if Not known.*<br>*Probe for months/ years if Not known*	1 = Yes<br>2 = No<br>3=Don't know	1=Male<br>2=Female<br>3=Don't know	1=Yes<br>2=No	*If less than 28 days enter number of days*	*Enter 01 for day if Not known.*<br>*Probe for months/years if Not known*
	1	__	__	DD/MM/ YYYY	—	—	—	DD/MM	DD/MM/ YYYY
	2	—	__	DD/MM/ YYYY	—	—	—	DD/MM	DD/MM/ YYYY
	3	—	__	DD/MM/ YYYY	—	—	—	DD/MM	DD/MM/ YYYY
	4	—	__	DD/MM/ YYYY	—	—	—	DD/MM	DD/MM/ YYYY
	5	—	__	DD/MM/ YYYY	—	—	—	DD/MM	DD/MM/ YYYY
	6	—	__	DD/MM/ YYYY	—	—	—	DD/MM	DD/MM/ YYYY

Tariku A, *et al. BMJ Open* 2022; 12:e058055. doi: 10.1136/bmjopen-2021-058055

**Section 2.3: Pregnancy care for last birth (in the last two years.**			
Now I want to talk to you about the last birth you had that ended in [DATE}, with the birth of [NAME}			
182h	Do you have a family health card with information about that pregnancy and birth?	1=Yes<br>2=No **SKIP TO 184**	__
183h	May I see your family health card?	1=Yes<br>2=No because it isn't at home<br>3=No, refused permission	__
184h	When pregnant with [NAME], did you receive any care during pregnancy? *(Probe: by care I mean any care from HEW, HW, nurses, midwives or doctors, clinics)*	1=Yes<br>2=No **SKIP TO 236**	__
185h_a	Where did you go for pregnancy care?<br>**At home**	1=Yes<br>2=No	__
185h_b	Where did you go for pregnancy care?<br>**At Health Post**	1=Yes<br>2=No	__
185h_c	Where did you go for pregnancy care?<br>**At Health Centre**	1=Yes<br>2=No	__
185h_d	Where did you go for pregnancy care?<br>**At Hospital**	1=Yes<br>2=No	__
185h_e	Where did you go for pregnancy care?<br>**Other**	1=Yes<br>2=No	__
For women who received pregnancy care at a hospital, health center or health post			
186h	How many times did you attend the hospital for pregnancy (antenatal) care for that pregnancy?	Enter the number of times<br>If the answer is "0" Skip to 190	__
188h	How old was your pregnancy at the first visit?	*Record No. of weeks. If don't know 99. Record from FHC/ANC appointment card if available*	__
190h	How many times did you attend the health center for pregnancy (antenatal) care for that pregnancy?	Enter the number of times<br>If the answer is "0" Skip to 194	__
192h	How old was your pregnancy at the first visit?	*Record No. of weeks. If don't know 99. Record from FHC/ANC appointment card if available*	__
194h	How many times did you attend the health post for pregnancy (antenatal) care for that pregnancy?	Enter the number of times<br>If the answer is "0" Skip to 199	__
196h	How old was your pregnancy at the first visit?	*Record No. of weeks. If don't know 99. Record from FHC/ANC appointment card if available*	__
For women who receive pregnancy care at home			
199h	How many times <u>did the HEW visit you</u> at home for pregnancy care that pregnancy?	Enter number of times<br>If answer is "0" Skip to 201	__
200h	How old was your pregnancy at the first HEW visit?	Record No. of weeks. If don't know 99. Record from FHC/ANC appointment card if available	__
Now I want to ask you about the pregnancy care you received (Verify with family health card if available)			
201h	Was your weight measured?	1=Yes<br>2=No **SKIP TO 203**	__
202h	In which location was this service first provided?	1=Home by HEW<br>2=Health Post<br>3=Health Centre<br>4=Hospital<br>8=Other	__
203h	Was your height measured?	1=Yes<br>2=No **SKIP TO 205**	__
204h	In which location was this service first provided?	1=Home by HEW<br>2=Health Post<br>3=Health Centre	__

		4=Hospital<br>8=Other	
205h	Did you receive information about breastfeeding?	1=Yes<br>2=No **SKIP TO 207**	__
206h	In which location was this service first provided?	1=Home by WDA<br>2=Home by HEW<br>3=Health post<br>4=Health center<br>5=Hospital<br>8=Other	__
207h	Was your blood pressure measured?<br>*(Probe: when a strap was put around your upper arm and a measure taken).*	1=Yes<br>2=No **SKIP TO 209**	__
208h	In which location was this service first provided?	1=Home by HEW<br>2=Health post<br>3=Health center<br>4=hospital<br>8=other	__
209h	Did you give a urine sample for a test?	1=Yes<br>2=No **SKIP TO 211**	__
210h	In which location was this service provided?	1=Home by HEW<br>2=Health post<br>3=Health center<br>4=Hospital<br>8=other	__
211h	Did you give blood for any test for syphilis?	1=Yes<br>2=No **SKIP TO 213**	__
212h	In which location was this service provided?	1=Home by HEW<br>2=Health Post<br>3=Health Centre<br>4=Hospital<br>8=Other	__
213h	Did you receive iron folate tablets or iron syrup?	1=Yes<br>2=No **SKIP TO 216**	__
214h	In which location was this service provided?	1=Home by HEW<br>2=Health post<br>3=Health Centre<br>4=Hospital<br>8=other	__
215h	For how many days did you take the tablets or syrup?	Enter number of days<br>Enter 999 if Not known	__
216h	Were you given an injection in the arm to prevent the baby from getting tetanus, that is, convulsions after birth?	1=Yes<br>2=No **SKIP TO 222**	__
217h	If Yes: How many times did you get a tetanus injection?	Write number of times	__
218h	If less than 2 times: **At any time before this pregnancy did you receive any tetanus injections?**	1=Yes<br>2=No **SKIP TO 221**	__
221h	In which health facility was tetanus injection given?	1=Home by HEW<br>2=Health post<br>3=Health Centre<br>4=Hospital<br>8=Other	__
222h	Did you receive HIV information?	1=Yes<br>2=No **SKIP TO 224**	__

223h	In which location was this service first provided?	1=Home by WDA<br>2=Home by HEW<br>3=Health post<br>4=Health Centre<br>5=Hospital<br>8=other	__
224h	Did you receive STI testing?	1=Yes<br>2=No **SKIP TO 226**	__
225h	In which location was this service provided?	1=Health Centre<br>2=Hospital<br>8=Other	__
226h	Did you receive any STI treatment?	1=Yes<br>2=No **SKIP TO 228**	__
227h	In which location was this service provided?	1=Health center<br>2=Hospital<br>8=Other	__
228h	Did you receive information on nutrition?	1=Yes<br>2=No **SKIP TO 230**	__
229h	In which location was this service first provided?	1=Home by WDA<br>2=Home by HEW<br>3=Health post<br>4=Health Centre<br>5=Hospital<br>8=Other	__
230h	Did you receive information on pregnancy danger signs?	1=Yes<br>2=No **SKIP TO 232**	__
231h	In which location was this service first provided?	1=Home by WDA<br>2=Home by HEW<br>3=Health post<br>4=Health Centre<br>5=Hospital<br>8=Other	__
232h	Were you advised on birth preparedness and complication readiness? *Probe: for finances, for help during delivery, for transport, for emergencies?*	1=Yes<br>2=No **SKIP TO 236**	__
233h	In which location was this service first provided?	1=Home by WDA<br>2=Home by HEW<br>3=Health post<br>4=Health Centre<br>5=Hospital<br>8=Other	__
234h	**Was your birth preparedness and complication readiness plan recorded?**	1=Yes<br>2=No **SKIP TO 236**	__
235h	**In which location was this service provided?**	1=Home by WDA<br>2=Home by HEW<br>3=Health post<br>4=Health Centre<br>5=Hospital<br>8=Other	__
Do Not read out list, prompt, 'anything else' record 1=Yes for all that apply			
236h	Can you tell me what are problems in pregnancy might need medical treatment? **Severe headache**	1=Yes<br>2= No	__
237h	Can you tell me what are problems in pregnancy might need	1=Yes	__

	medical treatment? **Blurry vision**	2= No	
238h	Can you tell me what are problems in pregnancy might need medical treatment? **Reduced or absent fetal movement**	1=Yes<br>2= No	__
239h	Can you tell me what are problems in pregnancy might need medical treatment? **High blood pressure**	1=Yes<br>2= No	__
240h	Can you tell me what are problems in pregnancy might need medical treatment? **Edema of the face/hands (i.e. swelling)**	1=Yes<br>2= No	__
241h	Can you tell me what are problems in pregnancy might need medical treatment? **Convulsions**	1=Yes<br>2= No	__
242h	Can you tell me what are problems in pregnancy might need medical treatment? **Excessive vaginal bleeding**	1=Yes<br>2= No	__
243h	Can you tell me what are problems in pregnancy might need medical treatment? **Severe lower abdominal pain**	1=Yes<br>2= No	__
244h	Can you tell me what are problems in pregnancy might need medical treatment? **Fever**	1=Yes<br>2= No	__
245h	Can you tell me what are problems in pregnancy might need medical treatment? **Anemia**	1=Yes<br>2= No	__
246h	Can you tell me what are problems in pregnancy might need medical treatment? **Other**	Specify	__
247h	Were you told where to go if you had any complications?	1=Yes<br>2= No **SKIP TO 249**	__
248h	If Yes, where were you told to go?	1= Health post<br>2= Health Centre<br>3= Hospital<br>8=Other (specify)<br>9=Don't know	__
249h	During your last pregnancy did you make any preparations for your delivery? *Probe: for finances, for help during delivery, transport, emergencies?*	1=Yes<br>2= No **SKIP TO 260**	__
Do Not read out list, prompt, 'anything else' record 1=Yes for all that apply			
250h	What preparations did you make for the delivery? **Financial**	1=Yes<br>2= No	__
251h	What preparations did you make for the delivery? **Transport**	1=Yes<br>2= No	__
252h	What preparations did you make for the delivery? **Food**	1=Yes<br>2= No	__
253h	What preparations did you make for the delivery? **Identification of birth attendant**	1=Yes<br>2= No	__
254h	What preparations did you make for the delivery? **Identification of facility**	1=Yes<br>2= No	__
255h	What preparations did you make for the delivery? **Clean clothes**	1=Yes<br>2= No	__
256h	What preparations did you make for the delivery?	1=Yes<br>2= No	__

Tariku A, *et al. BMJ Open* 2022; 12:e058055. doi: 10.1136/bmjopen-2021-058055

	**Cover to deliver on**		
257h	What preparations did you make for the delivery? **Gloves**	1=Yes<br>2= No	__
258h	What preparations did you make for the delivery? **Cotton gauze**	1=Yes<br>2= No	__
259h	What preparations did you make for the delivery? **Other**	Specify	__

**Section 2.4 Delivery for last birth**			
260h	Who assisted with the delivery?	1=Doctor<br>2=Nurse/Midwife<br>3=Health Extension Worker<br>4=Traditional Birth Attendant<br>5=Relative/friend<br>6=No-one **SKIP TO 263**<br>8= Other (specify)	__
261h	When you gave birth, did the person assisting you wear gloves during delivery?	1=Yes<br>2= No<br>9=Don't know	__
262h	When you gave birth, did the person assisting you wash her hands before the delivery?	1=Yes<br>2= No<br>9=Don't know	__
263h	Where did you give birth?	1=Home **SKIP TO 266**<br>2=Health post<br>3=Health Centre<br>4=Hospital<br>8=Other	__
264h	If health post/health centre/hospital: After giving birth, for how many days did you stay at the health post/health Centre/hospital in total?	Enter number of days, enter 0 if she left on the same day as delivery	__
265h	Was [NAME] delivered by caesarean, that is, did they cut your belly open to take the baby out?	1=Yes<br>2= No	__
Read out the list			
266h	During the delivery of [NAME] did you experience any of the following? **Heavy bleeding**	1=Yes<br>2= No	__
267h	During the delivery of [NAME] did you experience any of the following? **Labour more than 12 hours**	1=Yes<br>2= No	__
268h	During the delivery of [NAME] did you experience any of the following? **Loss of consciousness**	1=Yes<br>2= No	__
269h	During the delivery of [NAME] did you experience any of the following? **Premature labour**	1=Yes<br>2= No	__
270h	During the delivery of [NAME] did you experience any of the following? **Foul discharge**	1=Yes<br>2= No	__
271h	During the delivery of [NAME] did you experience any of the following? **Baby in abnormal position**	1=Yes<br>2= No	__
272h	During delivery were you advised to go to a different facility for specialist care (if home birth to a health post/health center/hospital, if health post/facility to a hospital/different	1=Yes<br>2= No **SKIP TO 275**	__

	facility)?		
273h	If Yes: Did you go to that different facility to get the specialist care (referral)?	1=Yes<br>2= No **SKIP TO 275**	__
274h	If Yes: What transport did you take to get there?	1=Own transport<br>2=Public transport<br>3=Hired transport<br>4=District/ambulance<br>5=Traditional ambulance (Qareza/cart)<br>6=Motor Bike/Bajaj<br>7=Walked<br>8=Other (specify)	__
275h	Did anyone check on your health after the delivery?	1=Yes<br>2= No **SKIP TO 288**	__
276h	How many times did anyone check on your health in the first month after delivery?	Write number. If don't know 99.	__
277h	How long after delivery did the first check take place?	Record number of days if same day as delivery enter 0. If don't know 99.	__
278h	Who checked on your health for the first time after you gave birth to [NAME]? *(Probe for most qualified person)*	1=Doctor/Health Officer<br>2=Nurse/Midwife/Health Officer<br>3=Health Extension Worker<br>4=Traditional birth attendant<br>8=Other (specify)	__
279h	Where did this check take place?	1=Home<br>2=Health post<br>3=Health center<br>4=Hospital<br>8=Other (specify)	__
280h	*If her health was checked at least twice (see Q above):* How long after delivery did the second check take place?	Record number of days. If don't know 99.	__
Do Not read out list, prompt, 'anything else' record 1=Yes for all that apply			
281h	During any of the health checks what was done to check on your health? **Examined body**	1=Yes<br>2= No	__
282h	During any of the health checks what was done to check on your health? **Checked breasts**	1=Yes<br>2= No	__
283h	During any of the health checks what was done to check on your health? **Checked for heavy bleeding**	1=Yes<br>2= No	__
284h	During any of the health checks what was done to check on your health? **Counselled on danger signs**	1=Yes<br>2= No	__
285h	During any of the health checks what was done to check on your health? **Counselled on family planning**	1=Yes<br>2= No	__
286h	During any of the health checks what was done to check on your health? **Counselled on nutrition**	1=Yes<br>2= No	__
287h	During any of the health checks what was done to check on your health? **Referred to a health facility**	1=Yes<br>2= No	__
287h_n	During any of the health checks what was done to check on your health? **None from the list**	1=Yes<br>2= No	__

Tariku A, *et al. BMJ Open* 2022; 12:e058055. doi: 10.1136/bmjopen-2021-058055

Section 2.5: Newborn Care			
Now I have some questions about what happened to [NAME] at the birth and immediately after.			
288h	Can I see a card recording information about the birth? (*use the card to verify all information if possible*)	1=Yes<br>2=No	__
289h	Was [NAME] weighed at birth?	1=Yes<br>2=No **SKIP TO 291**	__
290h	If Yes, how much did [NAME] weigh at birth? (*use the weight recorded on the card if possible)*	*Weight in grams e.g. if the weight was 1.9 kilograms enter 1900, If don't know 9999.*	__
291h	Did [NAME] have any difficulty breathing/crying at birth?	1=Yes<br>2=No **SKIP TO 293**	
292h_a	Did anyone do **Rubbing** to [NAME] immediately after birth?	1= Yes<br>2= No<br>9 = Don't know	__
292h_b	Did anyone do **Stimulating** to [NAME] immediately after birth?	1= Yes<br>2= No<br>9 = Don't know	__
292h_c	Did anyone do **Mouth-to-mouth** to [NAME] immediately after birth?	1= Yes<br>2= No<br>9= Don't know	__
292h_d	Did anyone do **Resuscitation** to [NAME] immediately after birth?	1= Yes<br>2= No<br>99 = Don't know	__
292h_e	Did anyone do **None of these** to [NAME] immediately at birth?	1= Yes<br>2= No<br>9= Don't know	__
293h	Where was [NAME] placed immediately after delivery?	1=Alone/on the floor<br>2=On the mother's belly/chest<br>3=Beside the mother<br>4=With someone else<br>8=Other (specify)<br>9= Don't know	__
294h	When [NAME] was born, was she/he dried/wiped?	1=Yes<br>2=No **If 2 or 9 Skip to 296**<br>9=Don't know	__
295h	How long after [NAME] was born was she/he dried/wiped?	*Enter in minutes, check for time after the baby was born (Not time after the placenta came out). If don't know 99*	__
296h	When [NAME] was born, was she/he wrapped with a cloth?	1=Yes<br>2= No **If 2 or 9 SKIP TO 298**<br>9=Don't know	__
297h	How long after [NAME] was born was she/he wrapped with a cloth?	*Enter in minutes, check for time after the baby was born (Not time after the placenta came out). If don't know 99*	__
298h	What was used to tie the cord?	1=New string/thread<br>2=Boiled string/thread<br>3=Any string/thread<br>4=Nothing<br>8=Other (specify)<br>9=Don't know	__
299h	What was used to cut the cord?	1=New razor blade<br>2=Any razor blade<br>3=Scissors<br>8=Other (specify)<br>9=Don't know	__
300h	Was anything applied to the cord after cutting and tying?	1=Yes<br>2= No **SKIP TO 308**	__

		9=Don't know **SKIP TO 308**	
Do Not read out list, prompt, 'anything else' record 1=Yes for all that apply			
301h	What was applied to the cord just after cutting the cord? **Butter**	1= Yes<br>2= No	__
302h	What was applied to the cord just after cutting the cord? **Ash**	1= Yes<br>2= No	__
303h	What was applied to the cord just after cutting the cord? **Ointment**	1= Yes<br>2= No	__
304h	What was applied to the cord just after cutting the cord? **Animal dung**	1= Yes<br>2= No	__
305h	What was applied to the cord just after cutting the cord? **Oil**	2= No<br>1= Yes	__
306h	What was applied to the cord just after cutting the cord? **Cold water**	1= Yes<br>2= No	__
307h	What was applied to the cord just after cutting the cord? **Other**	Specify________	__
308h	When [NAME] was born, how soon was [NAME] bathed?	1=In the first hour<br>2=After one-hour **SKIP TO 310**<br>3=After one day **SKIP TO 311**	__
309h	If in the first hour: After how many minutes would you say?	Write number of minutes.<br>**SKIP TO 310**	__
310h	If after one hour: After how many hours would you say?	*Write number of hours. If response is 'after one hour' enter 1. If response is 'after one and a half hours' enter 1.*<br>**SKIP TO 312**	__
311h	If after one day: After how many days would you say?	*Write number of days. If response is 'after one day' enter 1. If response is 'after one and a half days' enter 1.*	__
312h	In the first week of life, did you hold [NAME] skin to skin against your breasts during the daytime and night time?	1=Yes always<br>2=Yes very often<br>3=Yes sometime<br>4=Never<br>9=Don't know	__
313h	In the first week of life, did you sleep with [NAME] against you at night, or did you lay him/her alone on the bed or elsewhere?	1=Slept with mother<br>2=Baby slept alone<br>3=Baby slept with another person	__
314h	Did you ever breastfeed [NAME]?	1=Yes<br>2= No **SKIP TO 319**	__
315h	How long after birth did you first put [NAME] to the breast?	1=In the first hour<br>2=After one-hour **SKIP TO 317**<br>3=After one day **SKIP TO 318**	__
316h	If in the first hour: After how many minutes would you say?	Write number of minutes.<br>**SKIP TO 319**	__
317h	If after one hour: After how many hours would you say?	*Write number of hours. If response is 'after one hour' enter 1. If response is 'after one and a half hours' enter 1.*<br>**SKIP TO 319**	__
318h	How soon after the baby born did you bath him/ her?	*Write number of days. If response is 'after one day' enter 1. If response is 'after one and a half days' enter 1.*	__
319h	Did [NAME] receive vitamin K injection after delivery?	1 = Yes<br>2 = No<br>9= Don't know	__

320h	Did [NAME] receive TTC eye ointment after delivery?	1 = Yes<br>2 = No<br>9= Don't know	—
321h	Did [NAME] receive oral polio vaccine-after delivery?	1 = Yes<br>2 = No<br>9= Don't know	—
322h	Did you squeeze out and throw away the first milk?	1=Yes<br>2= No	—
323h	In the first three days after delivery, was [NAME] given anything to drink other than breast milk?	1=Yes<br>2= No	—
Now about care for [name] in the first month of his/her life			
324h	In the month after [NAME] was born, did any health care provider or a traditional birth attendant check on his/her health? *Probe for checks done at the place of birth on the same day as birth, and checks after*	1=Yes<br>2= No **SKIP TO 340**	—
325h	If Yes: In the month after [NAME] was born, how many times did a health care provider or a traditional birth attendant check on his/her health?	Write number of times.	—
326h	If Yes: How long after delivery did the first check take place?	*Record number of days if same day as delivery enter 0*	—
327h	If more than one time: How long after delivery did the second check take place?	*Record number of days*	—
328h	Who checked on [NAME] health the first time? (Probe for most qualified person)	1=Doctor/Health Officer<br>2=Nurse/Midwife<br>3=Health Extension Worker<br>4=Traditional birth attendant<br>8=Other (specify)	—
329h	Where did the first check on [NAME] take place?	1=Home<br>2=Health post<br>3=Health centre<br>4=Hospital<br>8=Other (specify)	—
330h	At any of the health checks in the first month, what was done to check the health of baby? **Generally examined/ looked at baby's body**	1=Yes<br>2= No	—
331h	At any of the health checks in the first month, what was done to check the health of baby? **Weighed baby**	1=Yes<br>2= No	—
332h	At any of the health checks in the first month, what was done to check the health of baby? **Checked cord**	1=Yes<br>2= No	—
333h	At any of the health checks in the first month, what was done to check the health of baby? **Counselled on breastfeeding**	1=Yes<br>2= No	—
334h	At any of the health checks in the first month, what was done to check the health of baby? **Observed breastfeeding**	1=Yes<br>2= No	—
335h	At any of the health checks in the first month, what was done to check the health of baby? **Counselled on skin-to-skin contact/warmth**	1=Yes<br>2= No	—
336h	At any of the health checks in the first month, what was done to check the health of baby?	1=Yes<br>2= No	—

	**Checked baby for danger signs**		
337h	At any of the health checks in the first month, what was done to check the health of baby? **Counselled on danger signs**	1=Yes<br>2= No	—
338h	At any of the health checks in the first month, what was done to check the health of baby? **Referred to a health facility**	1=Yes<br>2= No	—
339h	At any of the health checks in the first month, what was done to check the health of baby? **Nothing**	1=Yes<br>2= No	—
	*Now I want to talk to you about any sickness your child experienced in the **first month of life.***		
341h	Was [NAME] sick in the first month?	1=Yes<br>2= No **SKIP TO 396**	—
342h	Can I just check, did [NAME] have any of the following symptoms? **Stopped feeding well**	1=Yes<br>2= No	—
343h	Can I just check, did [NAME] have any of the following symptoms? **Difficult or fast breathing**	1=Yes<br>2= No	—
344h	Can I just check, did [NAME] have any of the following symptoms? **Chest in-drawing**	1=Yes<br>2= No	—
345h	Can I just check, did [NAME] have any of the following symptoms? **Unusually hot or cold**	1=Yes<br>2= No	—
346h	Can I just check, did [NAME] have any of the following symptoms? **Baby less active than usual**	1=Yes<br>2= No	—
347h	Can I just check, did [NAME] have any of the following symptoms? **Body became yellow**	1=Yes<br>2= No	—
348h	Can I just check, did [NAME] have any of the following symptoms? **Other**	**Specify**	—
349h	If any sickness/symptom reported: How old was [NAME] when sick for the first time?	*Record number of days of age when [NAME] was first sick if on first day of life enter 0*	—
	Check all the following symptoms: (Read all)		
350h	When [NAME] was sick that first time what was the problem? **Fever**	1=Yes<br>2= No	—
351h	When [NAME] was sick that first time what was the problem? **Unable to suckle/feed**	1=Yes<br>2= No	—
352h	When [NAME] was sick that first time what was the problem? **Difficult/fast breathing**	1=Yes<br>2= No	—
353h	When [NAME] was sick that first time what was the problem? **Severe chest in-drawing**	1=Yes<br>2= No	—
354h	When [NAME] was sick that first time what was the problem? **Diarrhea**	1=Yes<br>2= No	—
355h	When [NAME] was sick that first time what was the problem? **Convulsions**	1=Yes<br>2= No	—
356h	When [NAME] was sick that first time what was the problem? **Persistent vomiting**	1=Yes<br>2= No	—
357h	When [NAME] was sick that first time what was the problem? **Yellow palms/soles/eyes**	1=Yes<br>2= No	—

Tariku A, *et al. BMJ Open* 2022; 12:e058055. doi: 10.1136/bmjopen-2021-058055

358h	When [NAME] was sick that first time what was the problem? **Lethargy**	1=Yes<br>2= No	__
359h	When [NAME] was sick that first time what was the problem? **Unconscious**	1=Yes<br>2= No	__
360h	When [NAME] was sick that first time what was the problem? **Red/discharge eyes**	*1=Yes*<br>*2=No*	__
361h	When [NAME] was sick that first time what was the problem? **Skin pustules**	*1=Yes*<br>*2=No*	__
362h	When [NAME] was sick that first time what was the problem? **Skin around cord was red**	*1=Yes*<br>*2=No*	__
363h	When [NAME] was sick that first time what was the problem? **Pus from cord**	*1=Yes*<br>*2=No*	__
364h	When [NAME] was sick that first time what was the problem? **Other**	Specify	______
Select all that apply for the time before the baby was taken for extra care (do Not read out list, prompt, 'anything else' record 1=Yes for all that apply)			
365h	How was [NAME] treated for this illness at home? **Giving drugs**	*1=Yes*<br>*2=No*	__
366h	How was [NAME] treated for this illness at home? **Giving herbs**	*1=Yes*<br>*2=No*	__
367h	How was [NAME] treated for this illness at home? **Contacting a provider for advice**	*1=Yes*<br>*2=No*	__
368h	How was [NAME] treated for this illness at home? **Contacting someone else for advice**	*1=Yes*<br>*2=No*	__
369h	How was [NAME] treated for this illness at home? **No treatment given at home**	*1=Yes*<br>*2=No*	__
370h	How was [NAME] treated for this illness at home? **Don't remember**	*1=Yes*<br>*2=No*	__
371h	Did you seek care for [NAME] outside the home at that time?	1=Yes **SKIP TO 373**<br>2= No	__
372h_a	If No care sought at that time: Why didn't you seek care for [NAME] outside the home during that first illness?<br>**Expected him/her to get better**	*1=Yes*<br>*2=No*	__
372h_b	If No care sought at that time: Why didn't you seek care for [NAME] outside the home during that first illness? **Health facility too far**	*1=Yes*<br>*2=No*	__
372h_c	If No care sought at that time: Why didn't you seek care for [NAME] outside the home during that first illness?<br>**Cost of treatment too expensive**	*1=Yes*<br>*2=No*	__
372h_d	If No care sought at that time: Why didn't you seek care for [NAME] outside the home during that first illness? **Don't trust the facility**	*1=Yes*<br>*2=No*	__
372h_e	If No care sought at that time: Why didn't you seek care for [NAME] outside the home during that first illness? **Family member didn't allow**	*1=Yes*<br>*2=No*	__
372h_f	If No care sought at that time: Why didn't you seek care for [NAME] outside the home during that first illness?<br>**Community advisor/TBA advised against it**	*1=Yes*<br>*2=No*	__
372h_g	If No care sought at that time: Why didn't you seek care for [NAME] outside the home during that first illness? **Other**	Specify	__

373h	If sought care: How many times did you seek care for that illness?	Write number of times	__
374h	Where outside the home did you seek care from the first time?	1=Health post<br>2=Health centre<br>3=Hospital<br>4=Pharmacy/shop<br>8=Other (specify)	__
375h	Do you have any medical record from when you went for health care outside the home the first time?	1=Yes<br>2= No **SKIP TO 377**	__
376h	If Yes, can I see it?	1=Yes<br>2= No	__
377h	After how many days did you seek care the first time?	*Write number of days from the onset of illness, if first day of illness write 0. If possible use the medical record to confirm*	__
378h	If Yes to any of the symptoms (342-348): At any time during the illness, did [NAME] take any drugs for the illness?	*If the information is available from the card use it*<br>1=Yes<br>2= No ***(END OF MODULE)***	__
Select all mentioned (do Not read out list, prompt, 'anything else' record 1=Yes for all that apply)			
379h	What drugs did [NAME] take?<br>**Antimalarial tablet**	1=Yes<br>2= No	__
382h	What drugs did [NAME] take?<br>**Antibiotic: Gentamicin injection**	1=Yes<br>2= No	__
383h	What drugs did [NAME] take?<br>**Antibiotic: Amoxicillin syrup or tablet**	1=Yes<br>2= No	__
384h	What drugs did [NAME] take?<br>**Antibiotic: Cotrimoxazole syrup**	1=Yes<br>2= No	__
385h	What drugs did [NAME] take?<br>**Antibiotic: Other /Not known**	1=Yes<br>2= No	__
386h	What drugs did [NAME] take?<br>**Tetracycline eye ointment**	1=Yes<br>2= No	__
387h	What drugs did [NAME] take? **Zinc**	1=Yes<br>2= No	__
388h	What drugs did [NAME] take? **ORS**	1=Yes<br>2= No	__
389h	What drugs did [NAME] take?<br>**Combined Zinc-ORS**	1=Yes<br>2= No	__
390h	What drugs did [NAME] take? **Vitamin A**	1=Yes<br>2= No	__
391h	What drugs did [NAME] take? **Paracetamol**	1=Yes<br>2= No	__
392h	What drugs did [NAME] take?<br>**Traditional remedies herbs/local remedy**	1=Yes<br>2= No	__
393h	What drugs did [NAME] take? **Other**	Specify	________
394h	If Yes to gentamicin injection: Did [NAME] receive<br>**7 consecutive days of gentamycin injection?**	1=Yes<br>2= No<br>9 = Don't know	__
395h	If Yes to amoxicillin: Did [NAME] receive<br>**7 consecutive days of amoxicillin?**	1=Yes<br>2= No<br>9= Don't know	__

End – thank the participant. Check to see if the participant needs to answer the child module. If so, continue to Module 3. Otherwise check whether there is another woman aged 13-49 in the house.

Module 3 – Child Health

**Section 3.1: Identification of and consent from caregivers**		
396h	***Interviewer***: How many children under 5 years of age live in this household? Check household listing.	____
397h	***Interviewer***: select name of caregiver from household listing	____
398h	***Interviewer***: select ID of the caregiver from household listing	____
399h	Is it possible to interview the caregiver?	1=Yes **SKIP TO 401**<br>2=No
400h	If No, why Not possible to interview the caregiver?	1=Travelled away<br>2=Sick<br>3=Currently Not present<br>8=Other reason
401h	***Interviewer***: Have you read her the consent form?	1=Yes<br>2= No-one is available (add reason)
402h	***Interviewer***: Does he/she give her consent to be interviewed??	1=Yes<br>2=No END INTERVIEW.

**Section 3.2: Knowledge of malaria, diarrhea & pneumonia**		
Do Not read out list, prompt, 'anything else' record 1=Yes for all that apply		
403h	Why do you sleep under a mosquito net?<br>**Protects from malaria**	1=Yes<br>2=No
404h	Why do you sleep under a mosquito net?<br>**Protects from mosquitoes**	1=Yes<br>2=No
405h	Why do you sleep under a mosquito net?<br>**Given free**	1=Yes<br>2=No
406h	Why do you sleep under a mosquito net?<br>**Bought cheaply**	1=Yes<br>2=No
407h	Why do you sleep under a mosquito net?<br>**Look attractive**	1=Yes<br>2=No
408h	Why do you sleep under a mosquito net? Health worker said to use	1=Yes<br>2=No
409h	Why do you sleep under a mosquito net?<br>**Someone told me to use it**	1=Yes<br>2=No
410h	Why do you sleep under a mosquito net?<br>**Neighbor uses it**	1=Yes<br>2=No
411h	Why do you sleep under a mosquito net?<br>**Other**	Specify
412h	Why do you sleep under a mosquito net?<br>**Don't know**	1=Yes<br>2=No

Do Not read out list, prompt, 'anything else' record 1=Yes for all that apply		
413h	If you don't want to get sick from malaria, how can you protect yourself? **Use a Bednet**	1=Yes<br>2=No
414h	If you don't want to get sick from malaria, how can you protect yourself? **Use repellent or spray**	1=Yes<br>2=No
415h	If you don't want to get sick from malaria, how can you protect yourself? **Avoid mosquitoes/bites**	1=Yes<br>2=No
416h	If you don't want to get sick from malaria, how can you protect yourself? **Take medication for**	1=Yes<br>2=No
417h	If you don't want to get sick from malaria, how can you protect yourself? **prevention Keep surroundings clean**	1=Yes<br>2=No
418h	If you don't want to get sick from malaria, how can you protect yourself? **Eat well**	1=Yes<br>2=No
419h	If you don't want to get sick from malaria, how can you protect yourself? **Drink boiled water**	1=Yes<br>2=No
420h	If you don't want to get sick from malaria, how can you protect yourself? **Avoid getting wet from rain**	1=Yes<br>2=No
421h	If you don't want to get sick from malaria, how can you protect yourself? **Other**	Specify
422h	If you don't want to get sick from malaria, how can you protect yourself? **Don't know**	1=Yes<br>2=No
**423h**	Have you seen or hear any messages on: **Bed nets**	1=Yes<br>2=No
	Multiple options are possible. (do Not read out list, prompt, 'anything else' record 1=Yes for all that apply)	
423h_1	Where did you hear the message about **Bed nets** from? **Friend**	1=Yes<br>2=No
423h_2	Where did you hear the message about **Bed nets** from? **At Health Centre**	1=Yes<br>2=No
423h_3	Where did you hear the message about **Bed nets** from? **HEW**	1=Yes<br>2=No
423h_4	Where did you hear the message about **Bed nets** from? **WDA**	1=Yes<br>2=No
423h_5	Where did you hear the message about **Bed nets** from? **Newspaper**	1=Yes<br>2=No
423h_6	Where did you hear the message about **Bed nets** from? **TV**	1=Yes<br>2=No
423h_7	Where did you hear the message about **Bed nets** from? **Radio**	1=Yes<br>2=No
423h_8	Where did you hear the message about **Bed nets** from? **Government official**	1=Yes<br>2=No
423h_9	Where did you hear the message about **Bed nets** from? **Church/Mosque**	1=Yes<br>2=No
423h_10	Where did you hear the message about **Bed nets** from? **School**	1=Yes<br>2=No
423h_11	Where did you hear the message about **Bed nets** from? **Poster/flier**	1=Yes<br>2=No

Tariku A, *et al. BMJ Open* 2022; 12:e058055. doi: 10.1136/bmjopen-2021-058055

423h_12	Where did you hear the message about **Bed nets** from? **Meeting**	1=Yes<br>2=No
423h_13	Where did you hear the message about **Bed nets** from? **Street announcement**	1=Yes<br>2=No
423h_14	Where did you hear the message about **Bed nets** from? **Other**	Specify
**424h**	Have you seen or hear any messages on: **Malaria treatment using ACT**	1=Yes<br>2=No
Multiple options are possible. (do Not read out list, prompt, 'anything else' for all that apply)		
424h_1	Where did you hear the message about **ACT** from? **Friend**	1=Yes<br>2=No
424h_2	Where did you hear the message about **ACT** from? **At Health Centre**	1=Yes<br>2=No
424h_3	Where did you hear the message about **ACT** from? **HEW**	1=Yes<br>2=No
424h_4	Where did you hear the message about **ACT** from? **WDA**	1=Yes<br>2=No
424h_5	Where did you hear the message about **ACT** from? **Newspaper**	1=Yes<br>2=No
424h_6	Where did you hear the message about **ACT** from? **TV**	1=Yes<br>2=No
424h_7	Where did you hear the message about **ACT** from? **Radio**	1=Yes<br>2=No
424h_8	Where did you hear the message about **ACT** from? **Government official**	1=Yes<br>2=No
424h_9	Where did you hear the message about **ACT** from? **Church/Mosque**	1=Yes<br>2=No
424h_10	Where did you hear the message about **ACT** from? **School**	1=Yes<br>2=No
424h_11	Where did you hear the message about **ACT** from? **Poster/flier**	1=Yes<br>2=No
424h_12	Where did you hear the message about **ACT** from? **Meeting**	1=Yes<br>2=No
424h_13	Where did you hear the message about **ACT** from? **Street announcement**	1=Yes<br>2=No
424h_14	Where did you hear the message about **ACT** from? **Other**	Specify
**425h**	Have you seen or hear any messages on: **Rapid diagnostic tests (RDT) for malaria**	1=Yes<br>2=No
Multiple options are possible. (do Not read out list, prompt, 'anything else' for all that apply)		
425h_1	Where did you hear the message about **RDT** from? **Friend**	1=Yes<br>2=No
425h_2	Where did you hear the message about **RDT** from? **At Health Centre**	1=Yes<br>2=No
425h_3	Where did you hear the message about **RDT** from? **HEW**	1=Yes<br>2=No

Tariku A, *et al. BMJ Open* 2022; 12:e058055. doi: 10.1136/bmjopen-2021-058055

425h_4	Where did you hear the message about **RDT** from? **WDA**	1=Yes<br>2=No
425h_5	Where did you hear the message about **RDT** from? **Newspaper**	1=Yes<br>2=No
425h_6	Where did you hear the message about **RDT** from? **TV**	1=Yes<br>2=No
425h_7	Where did you hear the message about **RDT** from? **Radio**	1=Yes<br>2=No
425h_8	Where did you hear the message about **RDT** from? **Government official**	1=Yes<br>2=No
425h_9	Where did you hear the message about **RDT** from? **Church/Mosque**	1=Yes<br>2=No
425h_10	Where did you hear the message about **RDT** from? **School**	1=Yes<br>2=No
425h_11	Where did you hear the message about **RDT** from? **Poster/flier**	1=Yes<br>2=No
425h_12	Where did you hear the message about **RDT** from? **Meeting**	1=Yes<br>2=No
425h_13	Where did you hear the message about **RDT** from? **Street announcement**	1=Yes<br>2=No
425h_14	Where did you hear the message about **RDT** from? **Other**	Specify
**426h**	Have you seen or hear any messages on: **Rectal artesunate**	1=Yes<br>2=No
Multiple options are possible. (do Not read out list, prompt, 'anything else' for all that apply)		
426h_1	Where did you hear the message about **Rectal artesunate** from? **Friend**	1=Yes<br>2=No
426h_2	Where did you hear the message about **Rectal artesunate** from? **At Health Centre**	1=Yes<br>2=No
426h_3	Where did you hear the message about **Rectal artesunate** from? **HEW**	1=Yes<br>2=No
426h_4	Where did you hear the message about **Rectal artesunate** from? **WDA**	1=Yes<br>2=No
426h_5	Where did you hear the message about **Rectal artesunate** from? **Newspaper**	1=Yes<br>2=No
426h_6	Where did you hear the message about **Rectal artesunate** from? **TV**	1=Yes<br>2=No
426h_7	Where did you hear the message about **Rectal artesunate** from? **Radio**	1=Yes<br>2=No
426h_8	Where did you hear the message about **Rectal artesunate** from? **Government official**	1=Yes<br>2=No
426h_9	Where did you hear the message about **Rectal artesunate** from? **Church/Mosque**	1=Yes<br>2=No
426h_10	Where did you hear the message about **Rectal artesunate** from? **School**	1=Yes<br>2=No
426h_11	Where did you hear the message about **Rectal artesunate** from? **Poster/flier**	1=Yes<br>2=No

426h_12	Where did you hear the message about **Rectal artesunate** from? **Meeting**	1=Yes<br>2=No
426h_13	Where did you hear the message about **Rectal artesunate** from? **Street announcement**	1=Yes<br>2=No
426h_14	Where did you hear the message about **Rectal artesunate** from? **Other**	Specify
**427h**	Have you seen or hear any messages on:<br>**Diarrhea treatment**	1=Yes<br>2=No
Multiple options are possible. (do Not read out list, prompt, 'anything else' for all that apply)		
427h_1	Where did you hear the message about **Diarrhea treatment** from? **Friend**	1=Yes<br>2=No
427h_2	Where did you hear the message about **Diarrhea treatment** from? **At Health Centre**	1=Yes<br>2=No
427h_3	Where did you hear the message about **Diarrhea treatment** from? **HEW**	1=Yes<br>2=No
427h_4	Where did you hear the message about **Diarrhea treatment** from? **WDA**	1=Yes<br>2=No
427h_5	Where did you hear the message about **Diarrhea treatment** from? **Newspaper**	1=Yes<br>2=No
427h_6	Where did you hear the message about **Diarrhea treatment** from? **TV**	1=Yes<br>2=No
427h_7	Where did you hear the message about **Diarrhea treatment** from? **Radio**	1=Yes<br>2=No
427h_8	Where did you hear the message about **Diarrhea treatment** from? **Government official**	1=Yes<br>2=No
427h_9	Where did you hear the message about **Diarrhea treatment** from? **Church/Mosque**	1=Yes<br>2=No
427h_10	Where did you hear the message about **Diarrhea treatment** from? **School**	1=Yes<br>2=No
427h_11	Where did you hear the message about **Diarrhea treatment** from? **Poster/flier**	1=Yes<br>2=No
427h_12	Where did you hear the message about **Diarrhea treatment** from? **Meeting**	1=Yes<br>2=No
427h_13	Where did you hear the message about **Diarrhea treatment** from? **Street announcement**	1=Yes<br>2=No
427h_14	Where did you hear the message about **Diarrhea treatment** from? **Other**	Specify
**428h**	Have you seen or hear any messages on:<br>**Pneumonia treatment**	1=Yes<br>2=No
Multiple options are possible. (do Not read out list, prompt, 'anything else' for all that apply)		
428h_1	Where did you hear the message about **Pneumonia treatment** from? **Friend**	1=Yes<br>2=No
428h_2	Where did you hear the message about **Pneumonia treatment** from? **At Health Centre**	1=Yes<br>2=No
428h_3	Where did you hear the message about **Pneumonia treatment** from? **HEW**	1=Yes<br>2=No

428h_4	Where did you hear the message about **Pneumonia treatment** from? **WDA**	1=Yes<br>2=No
428h_5	Where did you hear the message about **Pneumonia treatment** from? **Newspaper**	1=Yes<br>2=No
428h_6	Where did you hear the message about **Pneumonia treatment** from? **TV**	1=Yes<br>2=No
428h_7	Where did you hear the message about **Pneumonia treatment** from? **Radio**	1=Yes<br>2=No
428h_8	Where did you hear the message about **Pneumonia treatment** from? **Government official**	1=Yes<br>2=No
428h_9	Where did you hear the message about **Pneumonia treatment** from? **Church/Mosque**	1=Yes<br>2=No
428h_10	Where did you hear the message about **Pneumonia treatment** from? **School**	1=Yes<br>2=No
428h_11	Where did you hear the message about **Pneumonia treatment** from? **Poster/flier**	1=Yes<br>2=No
428h_12	Where did you hear the message about **Pneumonia treatment** from? **Meeting**	1=Yes<br>2=No
428h_13	Where did you hear the message about **Pneumonia treatment** from? **Street announcement**	1=Yes<br>2=No
428h_14	Where did you hear the message about **Pneumonia treatment** from? **Other**	Specify
**429h**	Have you seen or hear any messages on:<br>**Treatment for sick newborns**	1=Yes<br>2=No
429h_1	Where did you hear the message about **Pneumonia treatment** from? **Friend**	1=Yes<br>2=No
Multiple options are possible. (do Not read out list, prompt, 'anything else' for all that apply)		
429h_2	Where did you hear the message **Treatment for sick newborns** from? **At Health Centre**	1=Yes<br>2=No
429h_3	Where did you hear the message about **Treatment for sick newborns** from? **HEW**	1=Yes<br>2=No
429h_4	Where did you hear the message about **Treatment for sick newborns** from? **WDA**	1=Yes<br>2=No
429h_5	Where did you hear the message about **Treatment for sick newborns** from? **Newspaper**	1=Yes<br>2=No
429h_6	Where did you hear the message about **Treatment for sick newborns** from? **TV**	1=Yes<br>2=No
429h_7	Where did you hear the message about **Treatment for sick newborns** from? **Radio**	1=Yes<br>2=No
429h_8	Where did you hear the message about **Treatment for sick newborns** from? **Government official**	1=Yes<br>2=No
429h_9	Where did you hear the message about **Treatment for sick newborns** from? **Church/Mosque**	1=Yes<br>2=No
429h_10	Where did you hear the message about **Treatment for sick newborns** from? **School**	1=Yes<br>2=No
429h_11	Where did you hear the message about **Treatment for sick newborns** from? **Poster/flier**	1=Yes<br>2=No

429h_12	Where did you hear the message about **Treatment for sick newborns** from? **Meeting**	1=Yes<br>2=No
429h_13	Where did you hear the message about **Treatment for sick newborns** from? **Street announcement**	1=Yes<br>2=No
429h_14	Where did you hear the message about **Treatment for sick newborns** from? **Other**	Specify

---

Do *Not read out list, prompt, 'anything else' record 1=Yes for all that apply)*			
444h	If your child is sick with a **fever** what should you do? **Ignore it will go away**	1=Yes<br>2=No	__
445h	If your child is sick with **a fever** what should you do? **Go to health centre**	1=Yes<br>2=No	__
446h	If your child is sick with **a fever** what should you do? **Go to HEW**	1=Yes<br>2=No	__
447h	If your child is sick with **a fever** what should you do? **Go to WDA**	1=Yes<br>2=No	__
448h	If your child is sick with **a fever** what should you do? **Go to traditional healer**	1=Yes<br>2=No	__
449h	If your child is sick with **a fever** what should you do? **Ask to be tested for malaria**	1=Yes<br>2=No	__
450h	If your child is sick with **a fever** what should you do? **Treat with antimalarial / ACT**	1=Yes<br>2=No	__
451h	If your child is sick with **a fever** what should you do? **Treat with paracetamol**	1=Yes<br>2=No	__
452h	If your child is sick with **a fever** what should you do? **Treat with antibiotic**	1=Yes<br>2=No	__
453h	If your child is sick with **a fever** what should you do? **Treat with other medicine**	1=Yes<br>2=No	__
454h	If your child is sick with **a fever** what should you do? **Other**	Specify	__
Do *Not read out list, prompt, 'anything else' record 1=Yes for all that apply)*			
455h	If your child is sick with **a diarrhea** what should you do? **Ignore it will go away**	1=Yes<br>2=No	__
456h	If your child is sick with **a diarrhea** what should you do? **Go to health centre**	1=Yes<br>2=No	__
457h	If your child is sick with **a diarrhea** what should you do? **Go to HEW**	1=Yes<br>2=No	__
458h	If your child is sick with **a diarrhea** what should you do? **Go to WDA**	1=Yes<br>2=No	__
459h	If your child is sick with **a diarrhea** what should you do? **Go to traditional healer**	1=Yes<br>2=No	__
460h	If your child is sick with **a diarrhea** what should you do? **Fluid made from a special**	1=Yes<br>2=No	__

	**packet / ordinary rehydration salts (ORS)**		
461h	If your child is sick with **a diarrhea** what should you do? **A gov-recommended home-made fluid for diarrhoea**	1=Yes<br>2=No	__
462h	If your child is sick with **a diarrhea** what should you do? **Treat with paracetamol**	1=Yes<br>2=No	__
463h	If your child is sick with **a diarrhea** what should you do? **Treat with antibiotic**	1=Yes<br>2=No	__
464h	If your child is sick with **a diarrhea** what should you do? **Treat with other medicine**	1=Yes<br>2=No	__
465h	If your child is sick with a diarrhea what should you do? **Other**	Specify	__
Do *Not read out list, prompt, 'anything else' record 1=Yes for all that apply)*			
466h	If your child is sick with **a cough/ respiratory** infection what should you do? **Ignore it will go away**	1=Yes<br>2=No	__
467h	If your child is sick with **a cough/ respiratory infection** what should you do? **Go to health centre**	1=Yes<br>2=No	__
468h	If your child is sick with **a cough/ respiratory infection** what should you do? **Go to HEW**	1=Yes<br>2=No	__
469h	If your child is sick with **a cough/ respiratory infection** what should you do? **Go to WDA**	1=Yes<br>2=No	__
470h	If your child is sick with **a cough/ respiratory infection** what should you do? **Go to traditional healer**	1=Yes<br>2=No	__
471h	If your child is sick with **a cough/ respiratory infection** what should you do? **Treat with antibiotic**	1=Yes<br>2=No	__
472h	If your child is sick with **a cough/ respiratory infection** what should you do? **Treat with paracetamol**	1=Yes<br>2=No	__
473h	If your child is sick with **a cough/ respiratory infection** what should you do? **Treat with other medicine**	1=Yes<br>2=No	__
474h	If your child is sick with **a cough/ respiratory infection** what should you do? **Other action**	Specify	__
Do *Not read out list, prompt, 'anything else' record 1=Yes for all that apply)*			
475h	What are the signs of illness for a newborn baby? **Reduced feeding**	1=Yes<br>2=No	__
476h	What are the signs of illness for a newborn baby? **Difficult or fast breathing**	1=Yes<br>2=No	__
477h	What are the signs of illness for a newborn baby? **Movement only when stimulated or No movement even when stimulated**	1=Yes<br>2=No	__

Tariku A, *et al. BMJ Open* 2022; 12:e058055. doi: 10.1136/bmjopen-2021-058055

478h	What are the signs of illness for a newborn baby? **Unusually hot or cold**	1=Yes<br>2=No	__
479h	What are the signs of illness for a newborn baby? **Convulsions**	1=Yes<br>2=No	__
480h	What are the signs of illness for a newborn baby? **Severe chest in-drawing**	1=Yes<br>2=No	__
481h	What are the signs of illness for a newborn baby? **Yellow palms/soles/eyes**	1=Yes<br>2=No	__
482h	What are the signs of illness for a newborn baby? **Diarrhea**	1=Yes<br>2=No	__
483h	What are the signs of illness for a newborn baby? **Skin pustules**	1=Yes<br>2=No	__
484h	What are the signs of illness for a newborn baby? **Cord red or draining puss**	1=Yes<br>2=No	__
485h	What are the signs of illness for a newborn baby? **Small infant (weight <2000 grams)**	1=Yes<br>2=No	__
Symptoms are: reduced feeding, difficult or fast breathing, Movement only when stimulated or No movement even when stimulated, unusually hot or cold, convulsions, severe chest in-drawing			
486h	If your newborn child **has <u>symptoms of neonatal sepsis</u>** what should you do? **Ignore it will go away**	1=Yes<br>2=No	__
487h	If your newborn child has **<u>symptoms of neonatal sepsis</u>** what should you do? **Go to health centre**	1=Yes<br>2=No	__
488h	If your newborn child has **<u>symptoms of neonatal sepsis</u>** what should you do? **Go to HEW**	1=Yes<br>2=No	__
489h	If your newborn child has **<u>symptoms of neonatal sepsis</u>** what should you do? **Go to WDA**	1=Yes<br>2=No	__
490h	If your newborn child has **<u>symptoms of neonatal sepsis</u>** what should you do? **Go to traditional healer**	1=Yes<br>2=No	__
491h	If your newborn child has **<u>symptoms of neonatal sepsis</u>** what should you do? **Treat with antibiotic**	1=Yes<br>2=No	__
492h	If your newborn child has **<u>symptoms of neonatal sepsis</u>** what should you do? **Treat with paracetamol**	1=Yes<br>2=No	__
493h	If your newborn child has **<u>symptoms of neonatal sepsis</u>** what should you do? **Treat with other medicine**	1=Yes<br>2=No	__
494h	If your newborn child has **<u>symptoms of neonatal sepsis</u>** what should you do? **Other action**	Specify	__

Tariku A, *et al. BMJ Open* 2022; 12:e058055. doi: 10.1136/bmjopen-2021-058055

Section 3.3: Health care available in kebele and recent interactions			
495h	Is there a health post in your kebele?	1= Yes<br>2=No **SKIP TO 506**	__
496h	How long does it take you to walk to the nearest health post?	*Record the distance in minutes. If don't know, record 99*	__
497h	How many times have you visited the health post in the last six months?	*Record the number of times*<br>*If any visits last 6 months,* ***SKIP TO 504***	__
Do Not read out list, prompt, 'anything else' record 1=Yes for all that apply then SKIP TO 504			
498h	If never in the last 6 months: What are the reasons why you have Not visited the health post in the last six months? **No illness in the family/No births**	1=Yes<br>2=No	__
499h	If never in the last 6 months: What are the reasons why you have Not visited the health post in the last six months? **Health post is too far away**	1=Yes<br>2=No	__
500h	If never in the last 6 months: What are the reasons why you have Not visited the health post in the last six months? **health post Costs too much to go to**	1=Yes<br>2=No	__
501h	If never in the last 6 months: What are the reasons why you have Not visited the health post in the last six months? **Not enough time to visit**	1=Yes<br>2=No	__
502h	If never in the last 6 months: What are the reasons why you have Not visited the health post in the last six months? **Poor services available at the health post**	1=Yes<br>2=No	__
503h	If never in the last 6 months: What are the reasons why you have Not visited the health post in the last six months? **Other**	Specify	_________________
504h	When was the last time you visited that health post?	*Record DD/MM. If don't know 99/99. If know month but Not day record 99/MM*	____/____
505h	The last time you visited the health post, what was the primary reason?	01=Family planning<br>02=Child immunisation<br>03=Antenatal care<br>04=Delivery care<br>05=Postnatal care<br>06=Neonatal care<br>07=Health education<br>08=Growth monitoring<br>09=Referral of sick child<br>10=Diarrhea treatment<br>11=Malaria treatment<br>13=Pneumonia treatment<br>14=other treatment of sickness<br>15=receive mosquito nets<br>88=other (specify)	____
506h	Is there another health facility in your kebele?	1= Yes<br>2=No **SKIP TO 518**	__
507h	What kind of a health facility	1=Health Centre<br>2=Hospital	__

		3=Private clinic<br>8=Other	
508h	How long does it take you to walk to the nearest health facility?	*Record the distance in minutes. If don't know, record 99*	____
509h	How many times have you visited the health facility in the last 6 months?	*Record the number of times*<br>*If any visits last 6 months,*<br>***SKIP TO 516***	__
Do Not read out list, prompt, 'anything else' record 1=Yes for all that apply Then ***SKIP TO 516***			
510h	If never in the last 6 months: What are the reasons why you have Not visited the health facility in the last six months?<br>**No illness in the family/No births**	1=Yes<br>2=No	__
511h	If never in the last 6 months: What are the reasons why you have Not visited the health facility in the last six months?<br>**Health facility is too far away**	1=Yes<br>2=No	__
512h	If never in the last 6 months: What are the reasons why you have Not visited the health facility in the last six months?<br>**Costs too much to go to health post**	1=Yes<br>2=No	__
513h	If never in the last 6 months: What are the reasons why you have Not visited the health facility in the last six months?<br>**Not enough time to visit**	1=Yes<br>2=No	__
514h	If never in the last 6 months: What are the reasons why you have Not visited the health facility in the last six months? **Poor services available at the health facility**	1=Yes<br>2=No	__
515h	If never in the last 6 months: What are the reasons why you have Not visited the health facility in the last six months? **Other**	Specify	_________________
516h	When was the last time you visited that health facility?	*Record DD/MM. If don't know 99/99. If know month but Not day record 99/MM*	____/___
517h	The last time you visited the health facility, what was the primary reason?	01=Family planning<br>02=Child immunisation<br>03=Antenatal care<br>04=Delivery care<br>05=Postnatal care<br>06=Neonatal care<br>07=Health education<br>08=Growth monitoring<br>09=Referral of sick child<br>10=Diarrhoea treatment<br>11=Malaria treatment<br>13=Pneumonia treatment<br>14=Other treatment of sickness<br>15=Receive mosquito nets<br>88=Other (specify)	____
518h	Have you been visited at home during the past 6 months by a Health Extension Worker to talk about health related issues?	1= Yes<br>2=No<br>**SKIP TO 537**	__
519h	When was the last time the HEW visited you at home?	*Record DD/MM. If don't know 99/99. If know month but Not day record 99/MM*	____/____
Do Not read out list, prompt, 'anything else' record 1=Yes for all that apply			

Tariku A, et al. BMJ Open 2022; 12:e058055. doi: 10.1136/bmjopen-2021-058055

520h	Who did the HEW talk to the last time she visited you at home? **Myself**	1=Yes<br>2=No	__
521h	Who did the HEW talk to the last time she visited you at home? **Other adult woman**	1=Yes<br>2=No	__
522h	Who did the HEW talk to the last time she visited you at home? **Head of household**	1=Yes<br>2=No	__
523h	Who did the HEW talk to the last time she visited you at home? **Other adult male**	1=Yes<br>2=No	__
Do Not read out list, prompt, 'anything else' record 1=Yes for all that apply			
524h	What was discussed the last time the HEW visited you at home? **Immunisation**	1= Yes<br>2=No	__
525h	What was discussed the last time the HEW visited you at home? **Child nutrition**	1=Yes<br>2=No	__
526h	What was discussed the last time the HEW visited you at home? **Family planning**	1=Yes<br>2=No	__
527h	What was discussed the last time the HEW visited you at home? **Pregnancy care**	1=Yes<br>2=No	__
528h	What was discussed the last time the HEW visited you at home? **Delivery care**	1=Yes<br>2=No	__
529h	What was discussed the last time the HEW visited you at home? **Newborn care / postnatal care**	1=Yes<br>2=No	__
530h	What was discussed the last time the HEW visited you at home? **Information about HIV/AIDS**	1=Yes<br>2=No	__
531h	What was discussed the last time the HEW visited you at home? **Information on hygiene**	1=Yes<br>2=No	__
532h	What was discussed the last time the HEW visited you at home? **Diarrhea treatment**	1=Yes<br>2=No	__
533h	What was discussed the last time the HEW visited you at home? **Malaria treatment**	1=Yes<br>2=No	__
534h	What was discussed the last time the HEW visited you at home? **Pneumonia treatment**	1=Yes<br>2=No	__
535h	What was discussed the last time the HEW visited you at home? **Promotion of latrine use**	1=Yes<br>2=No	__
536h	What was discussed the last time the HEW visited you at home? **Promotion of safe water use**	1=Yes<br>2=No	__
537h	Are you aware of women development army who visit people at home to talk about health issues?	1= Yes<br>2=No **SKIP TO 556**	__
538h	If Yes When was the last time the WDA visited you at home?	*Record DD/MM. If don't know 99/99. If know month but Not day record 99/MM*	____
Do Not read out list, prompt, 'anything else' record 1=Yes for all that apply			
539h	Who did the WDA talk to at that last visited to your home? **Myself**	1=Yes<br>2=No	__
540h	Who did the WDA talk to at that last visited to your home? **Other adult woman**	1=Yes<br>2=No	__
541h	Who did the WDA talk to at that last visited to your home? **Head of household**	1=Yes<br>2=No	__
542h	Who did the WDA talk to at that last visited to your home? **Other adult male**	1=Yes<br>2=No	__
Do Not read out list, prompt, 'anything else' record 1=Yes for all that apply			
543h	What was discussed the last time the WDA visited you at home? **Immunisation**	1=Yes<br>2=No	
544h	What was discussed the last time the WDA visited you at home? **Child nutrition**	1=Yes<br>2=No	__

545h	What was discussed the last time the WDA visited you at home? **Family planning**	1=Yes<br>2=No	__
546h	What was discussed the last time the WDA visited you at home? **Pregnancy care**	1=Yes<br>2=No	__
547h	What was discussed the last time the WDA visited you at home? **Delivery care**	1=Yes<br>2=No	__
548h	What was discussed the last time the WDA visited you at home? **Newborn care / postnatal care**	1=Yes<br>2=No	__
549h	What was discussed the last time the WDA visited you at home? **Information about HIV/AIDS**	1=Yes<br>2=No	__
550h	What was discussed the last time the WDA visited you at home? **Information on hygiene**	1=Yes<br>2=No	__
551h	What was discussed the last time the WDA visited you at home? **Diarrhoea treatment**	1=Yes<br>2=No	__
552h	What was discussed the last time the WDA visited you at home? **Malaria treatment**	1=Yes<br>2=No	__
553h	What was discussed the last time the WDA visited you at home? **Pneumonia treatment**	1=Yes<br>2=No	__
554h	What was discussed the last time the WDA visited you at home? **Promotion of latrine use**	1=Yes<br>2=No	__
555h	What was discussed the last time the WDA visited you at home? **Promotion of safe water use**	1=Yes<br>2=No	__
556h	Have you participated in developing local community-based action plan addressing maternal newborn and child health issues about health issues in the past 12 months?	1= Yes<br>2=No **SKIP TO 559**	__
557h	If Yes: When did you last participate in a community-based action plan?	*Record DD/MM. If don't know 99/99. If know month but Not day record 99/MM*	____/____
558h	Who organized the community-based action plan?	1=Kebele health team<br>2=Health extension worker<br>3=Community health worker<br>8=other (specify)<br>9=don't know	__
559h	Have you attended any meetings in your community (outside your home) about health issues in the past 12 months?	1= Yes<br>2=No **SKIP TO 575**	__
560h	If Yes: When was the last meeting you attended outside your home?	*Record DD/MM. If don't know 99/99. If know month but Not day record 99/MM*	____/____
561h	Who organised the last meeting?	1=Kebele health team<br>2=Health extension worker<br>3=Community health worker<br>8=other (specify)<br>9=don't know	__
Do Not read out list, prompt, 'anything else' record 1=Yes for all that apply			
562h	What was discussed at the last community meeting you attended outside your home? **Immunisation**	1= Yes<br>2=No	__
563h	What was discussed at the last community meeting you attended outside your home? **Child nutrition**	1= Yes<br>2=No	__
564h	What was discussed at the last community meeting you attended outside your home? **Family planning**	1= Yes<br>2=No	__
565h	What was discussed at the last community meeting you attended outside your home?	1= Yes<br>2=No	__

	**Pregnancy care**		
566h	What was discussed at the last community meeting you attended outside your home? **Delivery care**	1= Yes<br>2=No	__
567h	What was discussed at the last community meeting you attended outside your home? **Newborn care / postnatal care**	1= Yes<br>2=No	__
568h	What was discussed at the last community meeting you attended outside your home? **Information about HIV/AIDS**	1= Yes<br>2=No	__
569h	What was discussed at the last community meeting you attended outside your home? **Information on hygiene**	1= Yes<br>2=No	__
570h	What was discussed at the last community meeting you attended outside your home? **Diarrhea treatment**	1= Yes<br>2=No	__
571h	What was discussed at the last community meeting you attended outside your home? **Malaria treatment**	1= Yes<br>2=No	__
572h	What was discussed at the last community meeting you attended outside your home? **Pneumonia treatment**	1= Yes<br>2=No	__
573h	What was discussed at the last community meeting you attended outside your home? **Promotion of latrine use**	1= Yes<br>2=No	__
574h	What was discussed at the last community meeting you attended outside your home? **Promotion of safe water use**	1= Yes<br>2=No	__

Section 3.4: Identification of child & general questions			
The following questions are to be asked for all under 5 years old. Start with the youngest			
575h	***Interviewer***: What is the name of the youngest child**?**	Check household listing	_______________
576h	***Interviewer***: select ID of the child from household listing		____
577h	Did you ever breastfeed [name]? OR Was [name] breastfed?	1=Yes<br>2=No<br>9=Don't know	__
578h	Are you still breastfeeding [name]? OR Is [name] still being breastfed?	1=Yes<br>2=No<br>9=Don't know	__
579h	For how many months did you breastfeed? OR For how many months was [name] breastfed?	*Record number of months. If don't know record 98*	__
580h	Did [name] receive a vitamin A dose (like this) during the last 6 months?	Show ampule/capsule/syrup<br>1=Yes<br>2=No<br>9=Don't know	__

Section 3.5: Immunizations			
581h	Do you have a card where [name's] vaccinations are written down? If Yes, may I see it?	1=Yes seen **SKIP TO 583**<br>2=Yes Not seen **SKIP TO 599**<br>3=No<br>9=Don't know	__
582h	If No card, did you ever have a vaccination card?	1=Yes<br>2=No<br>9=Don't know	__<br>If ALL answered **SKIP TO 599**
Record 44 in the DAY column if card shows that a vaccination was given but No date is recorded.) Record DD/MM/YYYY.			
583h	Copy vaccination date for **BCG** from the card	____/____/________	
584h	Copy vaccination date for **Polio 0 (Polio given at birth)** from the card	____/____/________	
585h	Copy vaccination date for **Polio 1** from the card	____/____/________	
586h	Copy vaccination date for **Polio 2** from the card	____/____/________	
587h	Copy vaccination date for **Polio 3** from the card	____/____/________	
588h	Copy vaccination date for **Pentavalent (DPT, HIV, Hep B)** from the card	____/____/________	
589h	Copy vaccination date for **Pentavalent 2** from the card	____/____/________	
590h	Copy vaccination date for **Pentavalent 3** from the card	____/____/________	
591h	Copy vaccination date for **PCV 1** from the card	____/____/________	

592h	Copy vaccination date for **PCV 2** from the card	____/____/________	
593	Copy vaccination date for **Rota 1** from the card	____/____/________	
594	Copy vaccination date for **Rota 2** from the card	____/____/________	
595	Copy vaccination date for **Rota 3** from the card	____/____/________	
596	Copy vaccination date for **Measles** from the card	____/____/________	
597	Copy vaccination date for **Vitamin A** from the card	____/____/________	
598	Copy vaccination date for **Deworming** from the card	____/____/________	
Ask and fill this question, only If the vaccination status in the card is empty (583-598)			
599h	Did [name] receive any vaccinations that are not on this card, including vaccinations received in a national immunization campaign?	1=Yes<br>2=No<br>9=Don't know	*Record Yes only if respondent mentions BCG, Polio 0-3, Pentavalent, and/or measles vaccine(s)*
600h	Did [name] receive **BCG**?	1=Yes<br>2=No<br>9=Don't know	__
601h	Did [name] receive **Polio 0 (Polio given at birth)?**	1=Yes<br>2=No<br>9=Don't know	__
602h	Did [name] receive **Polio 1**?	1=Yes<br>2=No<br>9=Don't know	__
603h	Did [name] receive **Polio 2?**	1=Yes<br>2=No<br>9=Don't know	__
604h	Did [name] receive **Polio 3**?	1=Yes<br>2=No<br>9=Don't know	__
605h	Did [name] receive **Pentavalent (DPT, HIV, Hep B)**?	1=Yes<br>2=No<br>9=Don't know	__
606h	Did [name] receive **Pentavalent 2**?	1=Yes<br>2=No<br>9=Don't know	__
607h	Did [name] receive **Pentavalent 3?**	1=Yes<br>2=No<br>9=Don't know	__
608h	Did [name] receive **PCV 1**?	1=Yes<br>2=No<br>9=Don't know	__
609h	Did [name] receive **PCV 2**?	1=Yes<br>2=No<br>9=Don't know	__
610h	Did [name] receive **Rota 1**?	1=Yes<br>2=No<br>9=Don't know	__

Tariku A, *et al. BMJ Open* 2022; 12:e058055. doi: 10.1136/bmjopen-2021-058055

611h	Copy vaccination date for **Rota 2**?	1=Yes<br>2=No<br>9=Don't know	__
612h	Did [name] receive **Rota 3**?	1=Yes<br>2=No<br>9=Don't know	__
613h	Did [name] receive **Measles**?	1=Yes<br>2=No<br>9=Don't know	__
614h	Did [name] receive **Vitamin A**?	1=Yes<br>2=No<br>9=Don't know	__
615h	Did [name] receive **Deworming**?	1=Yes<br>2=No<br>9=Don't know	__
616h	Did [name] ever receive any vaccinations to prevent him/her getting diseases, including vaccinations received in a national immunization day campaign?	1=Yes<br>2=No<br>9=Don't know	__
Please tell me if [name] received any of the following vaccinations:			
617h	A BCG vaccination against tuberculosis, that is an injection in the arm or shoulder that usually cases a scar?	1=Yes<br>2=No<br>9=Don't know	__
618h	A POLIO vaccination, that is drops in the mouth?	1=Yes<br>2=No<br>9=Don't know	__
619h	When was the first polio vaccine received, just after birth or later?	1=just after birth<br>2=later	__
620h	How many times was the polio vaccine received?	Record number.<br>9=don't know	__
621h	A PENTAVALENT vaccination, which is an injection given in the thigh or buttocks, sometimes at the same time as the polio drops?	1=Yes<br>2=No<br>9=Don't know	__
622h	How many times was pentavalent received?	*Record number.*<br>*9=don't know*	__
623h	An injection to prevent MEASLES, usually in the upper left arm?	1=Yes<br>2=No<br>9=Don't know	__
624h	Has [name] had any illness at any time in the last 2 weeks?	1=Yes<br>2=No **Go to718**<br>9=Don't know **Go to 718**	__

Tariku A, *et al. BMJ Open* 2022; 12:e058055. doi: 10.1136/bmjopen-2021-058055

Section 3.6: Two-Week Morbidity Module (symptoms, care seeking and drugs)			
FILL IN THE MODULE BELOW IF THE CHILD WAS ILL IN THE PREVIOUS TWO WEEKS. IF THE CHILD HAD MORE THAN ONE EPISODE, CONSIDER ONLY THE MOST RECENT ONE.			
625h	For how many days was [name] ill?	*If less than one day record 00*<br>*99 if don't know*	__
626h	Is [name] ill Now?	1=Yes<br>2=No	__
**Symptoms**			
627h	Did [name] have any of the following at any time in the last 2 weeks? **Convulsions**	1=Yes<br>2=No	__
628h	Did [name] have any of the following at any time in the last 2 weeks? **Very sleepy**	1=Yes<br>2=No	__
629h	Did [name] have any of the following at any time in the last 2 weeks? **Vomiting everything**	1=Yes<br>2=No	__
630h	Did [name] have any of the following at any time in the last 2 weeks? **Drinking poorly/Not able to drink or breastfeed**	1=Yes<br>2=No	__
631h	Did [name] have any of the following at any time in the last 2 weeks? **Fever**	1=Yes<br>2=No	__
632h	Did [name] have any of the following at any time in the last 2 weeks? **Cough**	1=Yes<br>2=No	__
633h	Did [name] have any of the following at any time in the last 2 weeks? **Difficult breathing**	1=Yes<br>2=No	__
634h	Did [name] have any of the following at any time in the last 2 weeks? **Diarrhea**	1=Yes<br>2=No	__
635h	Did [name] have any of the following at any time in the last 2 weeks? **Ear pain**	1=Yes<br>2=No	__
636h	Did [name] have any of the following at any time in the last 2 weeks? **Loss of appetite**	1=Yes<br>2=No	__
637h	Did [name] have any of the following at any time in the last 2 weeks? **Blocked or runny Nose**	1=Yes<br>2=No	__
638h	Did [name] have any of the following at any time in the last 2 weeks? **Fast breathing**	1=Yes<br>2=No	__
639h	Did [name] have any of the following at any time in the last 2 weeks? **Eye problems**	1=Yes<br>2=No	__
640h	Did [name] have any of the following at any time in the last 2 weeks? **Generalized rash**	1=Yes<br>2=No	__
641h	Did [name] have any of the following at any time in the last 2 weeks? **Other signs/symptoms**	1=Yes<br>2=No	__
642h	How many days ago did this start	*If less than one day record 00*<br>*99 if don't know*	____
If symptoms Fast Breathing or Difficult Breathing were selected ask the following question:			
643h	Were symptoms of fast or difficult breathing due to problem in the chest or to a blocked Nose?	1=Chest<br>2=Nose<br>3=Both	__

		8= Other<br>9=Don't know	
If Diarrhea was selected ask the following questions:			
644h	When [name] was sick with diarrhea, how many watery stools did [name] have?	If less than one day record 00<br>99 if don't know	_____
645h	During the diarrhea, did [name] have?<br>**Repeated vomiting**	1=Yes<br>2=No	__
646h	During the diarrhea, did [name] have?<br>**Marked thirst**	1=Yes<br>2=No	__
647h	During the diarrhea, did [name] have?<br>**Not eating/drinking well**	1=Yes<br>2=No	__
648h	During the diarrhea, did [name] have?<br>**Blood in the stool**	1=Yes<br>2=No	__
649h	During the diarrhea, did [name] have?<br>**Not getting better/getting sicker**	1=Yes<br>2=No	__
650h	Now I would like to know how much [name] was offered to drink during the diarrhoea. Was he/she given less to drink than usual, more than usual, or about the same amount?<br>If less, probe: was he/she given *much less than usual to drink or somewhat less.*	1=Much less<br>2=Somewhat less<br>3=About the same<br>4=More<br>5=Nothing to drink<br>9=Don't know	__
651h	When [name] had diarrhea, was he/she given less to eat than usual, more than usual, or about the same amount?<br>*If less, probe: was he/she given much less than usual to eat or somewhat less.*	1=Much less<br>2=Somewhat less<br>3=About the same<br>4=More<br>5=Nothing to drink<br>9=Don't know	__
**Care seeking**			
652h	Did you seek advice or treatment for [name]'s illness from any source?	1= Yes **SKIP TO 660**<br>2= No<br>9=Don't know **SKIP TO 664**	__
653h	Why did you Not seek advice or treatment from any source? **Health facility too far**	1= Yes<br>2= No **SKIP TO** 718	__
654h	Why did you Not seek advice or treatment from any source? **Had no time**	1= Yes<br>2= No **SKIP TO** 718	__
655h	Why did you Not seek advice or treatment from any source? **Had no money**	1= Yes<br>2= No **SKIP TO** 718	__
656h	Why did you Not seek advice or treatment from any source? **Did Not want to attend a health facility**	1= Yes<br>2= No **SKIP TO** 718	__
657h	Why did you Not seek advice or treatment from any source? **Could manage at home**	1= Yes<br>2= No **SKIP TO** 718	__
658h	Why did you Not seek advice or treatment from any source? **Spouse did Not allow**	1= Yes<br>2= No **SKIP TO** 718	__
659h	Why did you Not seek advice or treatment from any source? **Other**	1= Yes<br>2= No **SKIP TO** 718	__
660h	Where did you seek advice or treatment? Anywhere else? **Health Post**	1= Yes<br>2= No	__
661h	Where did you seek advice or treatment? Anywhere else? **Health Centre**	1= Yes<br>2= No	__

662h	Where did you seek advice or treatment? Anywhere else? **Hospital**	1= Yes<br>2= No	__
663h	Where did you seek advice or treatment? Anywhere else? **Other**	Specify	___________
664h	***Interviewer:*** Check was treatment sought at two or more places?	1=Yes<br>2=No **SKIP TO 665**	__
665h	Where did you first seek treatment?	1=Health Post<br>2=Health Centre<br>3=Hospital<br>8=Other	__
666h	How many days after the illness began did you first seek treatment for [name]?	*If same day record 00. If don't know record 99.*	____
667h	Did the health worker ask you to bring [name] back to be seen again?	1=Yes<br>2=No<br>9=Don't know **SKIP TO 670**	__
668h	When did he/she say that [name] should be brought back?	1=Before today's date<br>2=After today's date **SKIP TO 670**<br>9=Don't know **SKIP TO 670**	__
669h	Did you take [name] back?	1=Yes<br>2=No<br>9=Don't know	__
670h	Has [name] been hospitalized for this illness?	1=Yes<br>2=No **SKIP TO 672**<br>9=Don't know **SKIP TO 672**	__
671h	How many nights has [name] been hospitalized during this illness?	*Record number of nights. If don't know record 99.*	____
672h	Has [name] been hospitalized at any time in the last 3 months?	1=Yes<br>2=No<br>9=Don't know	__
673h	How many nights has [name] been hospitalized in the last three months?	Record number of nights. If don't know record 99.	____
674h	At any time during this illness, did [name] have blood taken from his/her finger or heel for testing?	1=Yes<br>2=No **SKIP TO 678**<br>9=Don't know **SKIP TO 678**	__
675h	Which test was done?	1=Microscopy<br>2=RDT<br>9=Don't know	__
676h	Where was the test done?	1=Health Post<br>2=Health Centre<br>3=Hospital<br>8=Other	__
677h	Was the test positive for malaria?	1=Yes<br>2=No<br>9=Don't know	__
678h	In addition, did you seek advice from any of the following? **Traditional birth attendants**	1=Yes<br>2=No	__
679h	In addition, did you seek advice from any of the following? **Religious leader**	1=Yes<br>2=No	__
680h	In addition, did you seek advice from any of the following? **Pharmacy**	1=Yes<br>2=No	__
681h	In addition, did you seek advice from any of	1=Yes<br>2=No	__

	the following? **Traditional drug seller**		
682h	In addition, did you seek advice from any of the following? **Relative or friend**	1=Yes<br>2=No	__
683h	In addition, did you seek advice from any of the following? **Other**	**Specify**	___________

**Drugs**			
684h	At any time during the illness, was [name] prescribed any drugs for the illness?	1=Yes<br>2=No **SKIP TO 718**<br>9=Don't know **SKIP TO 718**	__
685h_a	What drugs did [name] take? **Antimalarial tablet (Co-artem, Chloroquine, etc)**	1= Yes<br>2 = No	__
685h_b	How long after the illness started did [name] first take **Antimalarial tablet**?	Write number of days. If child did Not take drug write 0 If 7 days or more write 7.<br>If don't know write 9	__
685h_c	**Did [name] complete taking the Antimalarial tablet?**	1= Yes<br>2 = No	__
685h_d	**Did you have [Antimalarial tablet] at home or did you get it from somewhere else.**<br><br>*If somewhere else, probe for the source.*	1=Home<br>2=Public health facility<br>3=Private health facility<br>4=HEW<br>5=Shop<br>8=Other Specify______<br>9=Don't know	__
694h_a	What drugs did [name] take?<br>**Amoxycillin**	1= Yes<br>2 = No	__
694h_b	How long after the illness started did [name] first take **Amoxycillin?**	Write number of days. If child did Not take drug write 0. If 7 days or more write 7.<br>If don't know write 9	__
694h_c	Did [name] complete taking the **Amoxycillin?**	1= Yes<br>2 = No	__
694h_d	Did you have **Amoxycillin** at home or did you get it from somewhere else.<br><br>*If somewhere else, probe for the source.*	1=Home<br>2=Public health facility<br>3=Private health facility<br>4=HEW<br>5=Shop<br>8=Other Specify______<br>9=Don't know	__
695h_a	What drugs did [name] take?<br>**Erythromycin**	1= Yes<br>2 = No	__
695h_b	How long after the illness started did [name] first take **Erythromycin?**	Write number of days. If child did Not take drug write 0. If 7 days or more write 7. If don't know write 9	__
695h_c	Did [name] complete taking the **Erythromycin?**	1= Yes<br>2 = No	__
695h_d	Did you have **Erythromycin** at home or did you get it from somewhere else?<br><br>*If somewhere else, probe for the source.*	1=Home<br>2=Public health facility<br>3=Private health facility<br>4=HEW<br>5=Shop<br>8=Other Specify______	__

		9=Don't know	
696h_a	What drugs did [name] take?<br>**Azithromycin**	1= Yes<br>2 = No	__
696h_b	How long after the illness started did [name] first take **Azithromycin?**	Write number of days. If child did Not take drug write 0. If 7 days or more write 7.<br>If don't know write 9	__
696h_c	Did [name] complete taking the **Azithromycin?**	1= Yes<br>2 = No	__
696h_d	Did you have **Azithromycin** at home or did you get it from somewhere else.<br><br>*If somewhere else, probe for the source.*	1=Home<br>2=Public health facility<br>3=Private health facility<br>4=HEW<br>5=Shop<br>8=Other Specify______<br>9=Don't know	__
697h_a	What drugs did [name] take?<br>**Cotrimoxazole**	1= Yes<br>2 = No	__
697h_b	How long after the illness started did [name] first take **Cotrimoxazole?**	Write number of days. If child did Not take drug write 0. If 7 days or more write 7.<br>If don't know write 9	__
697h_c	**Did [name] complete taking the Cotrimoxazole?**	1= Yes<br>2 = No	__
697h_d	Did you have **Cotrimoxazole** at home or did you get it from somewhere else.<br><br>*If somewhere else, probe for the source.*	1=Home<br>2=Public health facility<br>3=Private health facility<br>4=HEW<br>5=Shop<br>8=Other Specify______<br>9=Don't know	__
698h_a	What drugs did [name] take?<br>**Other antibiotic (pill or syrup)**	1= Yes<br>2 = No	__
698h_b	How long after the illness started did [name] first take **other antibiotic (pill or syrup)?**	Write number of days. If child did Not take drug write 0. If 7 days or more write 7.<br>If don't know write 9	__
698h_c	Did [name] complete taking **other antibiotic (pill or syrup)?**	1= Yes<br>2 = No	__
698h_d	Did you have **other antibiotic (pill or syrup)** at home or did you get it from somewhere else.<br><br>*If somewhere else, probe for the source.*	1=Home<br>2=Public health facility<br>3=Private health facility<br>4=HEW<br>5=Shop<br>8=Other Specify______<br>9=Don't know	__
699h_a	What drugs did [name] take?<br>**Antibiotic injection**	1= Yes<br>2 = No	__
699h_b	How long after the illness started did [name] first take **antibiotic injection?**	Write number of days. If child did Not take drug write 0. If 7 days or more write 7.<br>If don't know write 9	__
699h_c	Did [name] complete taking **antibiotic injection?**	1= Yes<br>2 = No	__
699h_d	Did you have **antibiotic injection** at home or did you get it from somewhere else**.**	1=Home<br>2=Public health facility<br>3=Private health facility	

	If somewhere else, probe for the source.	4=HEW<br>5=Shop<br>8=Other Specify______<br>9=Don't know	__
700h_a	What drugs did [name] take?<br>**Zinc**	1= Yes<br>2 = No	__
700h_b	How long after the illness started did [name] first take **Zinc?**	Write number of days. If child did Not take drug write 0. If 7 days or more write 7. If don't know write 9	__
700h_c	Did [name] complete taking the **Zinc?**	1= Yes<br>2 = No	__
700h_d	Did you have **Zinc** at home or did you get it from somewhere else.<br>*If somewhere else, probe for the source.*	Write number of days. If child did Not take drug write 0 If 7 days or more write 7. If don't know write 9	__
701h_a	What drugs did [name] take?<br>**Fluid made from special packet called ORS**	1= Yes<br>2 = No	__
701h_b	How long after the illness started did [name] first take f**luid made from special packet called ORS**?	Write number of days. If child did Not take drug write 0. If 7 days or more write 7. If don't know write 9	__
702h_a	What drugs did [name] take?<br>**ORS-Zinc combined**	1= Yes<br>2 = No	__
702h_b	How long after the illness started did [name] first take **ORS-Zinc combined**?	Write number of days. If child did Not take drug write 0. If 7 days or more write 7. If don't know write 9	__
704h_a	What drugs did [name] take?<br>**Gov. recommended home mad fluid**	1= Yes<br>2 = No	__
704h_b	How long after the illness started did [name] first take **Gov. recommended home mad fluid?**	Write number of days. If child did Not take drug write 0. If 7 days or more write 7. If don't know write 9	__
705h_a	What drugs did [name] take?<br>**Pill or Syrup**	1= Yes<br>2 = No	__
705h_b	How long after the illness started did [name] first take **Pill or Syrup?**	Write number of days. If child did Not take drug write 0. If 7 days or more write 7. If don't know write 9	__
706h_a	What drugs did [name] take?<br>**Injection**	1= Yes<br>2 = No	__
706h_b	How long after the illness started did [name] first take **Injection**?	Write number of days. If child did Not take drug write 0. If 7 days or more write 7. If don't know write 9	__
707h_a	What drugs did [name] take?<br>**IV intravenous**	1= Yes<br>2 = No	__
707h_b	How long after the illness started did [name] first take **IV intravenous?**	Write number of days. If child did Not take drug write 0. If 7 days or more write 7. If don't know write 9	__
708h_a	What drugs did [name] take?<br>**Home remedies/herbal medicine**	1= Yes<br>2 = No	__
708h_b	How long after the illness started did [name] first take **home remedies/herbal medicine?**	Write number of days. If child did Not take drug write 0. If 7 days or more write 7. If don't know write 9	__
709h_a	What drugs did [name] take?<br>**Paracetamol**	1= Yes<br>2 = No	__
709h_b	How long after the illness started did [name]	Write number of days. If child did Not take drug	__

	first take **Paracetamol?**	write 0. If 7 days or more write 7.<br>If don't know write 9	
710h_a	What drugs did [name] take?<br>**Panadol**	1= Yes<br>2 = No	__
710h_b	How long after the illness started did [name] first take **Panadol?**	Write number of days. If child did Not take drug write 0. If 7 days or more write 7.<br>If don't know write 9	__
711h_a	What drugs did [name] take?<br>**Aspirin**	1= Yes<br>2 = No	__
711h_b	How long after the illness started did [name] first take **Aspirin?**	Write number of days. If child did Not take drug write 0. If 7 days or more write 7.<br>If don't know write 9	__
712h_a	What drugs did [name] take?<br>**Ibuprofen**	1= Yes<br>2 = No	__
712h_b	How long after the illness started did [name] first take **Ibuprofen**?	Write number of days. If child did Not take drug write 0. If 7 days or more write 7.<br>If don't know write 9	__
713h_a	What **other** drugs did [name] take?<br>**Other**	Specify	__
713h_b	How long after the illness started did [name] first take **other drug?**	Write number of days. If child did Not take drug write 0. If 7 days or more write 7.<br>If don't know write 9	__
714h	What **other** drugs did [name] take?<br>**Don't know**	1= Yes<br>2 = No	__
**Section 3.7: MUAC**			
718h	Mid-upper arm circumference	*In centimeters*<br>*9994=If Not present*<br>*9995=Refused*<br>*9996=Other*	____|.__
