# Supplementary material for: Health postservice readiness and use of preventive and curative services for suspected childhood pneumonia in Ethiopia: a cross-sectional study
Source: BMJ Open. 2022 Apr 26;12(4):e058055. doi: 10.1136/bmjopen-2021-058055 (PMC9047705; doi:10.1136/bmjopen-2021-058055)
Supplement: Supplementary data [file bmjopen-2021-058055supp002.pdf]

ANNEX C: INSTRUMENTS FOR HEALTH PROVIDER SURVEY

Dagu Baseline Facility Questionnaire: Health Post

MODULE 1: BACKGROUND INFORMATION OF THE HEALTH POST

100	**Date (Gregorian Calendar)**	|___|___| / |___|___| / |___|___| dd / mm / yy	
101	**Region Code**	|__|__|__|__|__|__|	
102	**Zone Code**	|__|__|__|__|__|__||__|__|__|__|	
103	**Woreda Name**	|__|__|__|__|__|__||__|__|__|__|	
104	**Woreda code**	|__|__|__|__|__|__||__|__|__|__|	
105	**PHCU/Health Centre name**	|__|__|__|__|__|__||__|__|__|__|	
106	**Kebele name**	|__|__|__|__|__|__||__|__|__|__|	
107	**Gote name**	|__|__|__|__|__|__||__|__|__|__|	
108	**Cluster code**	|___|___|	
109	**GPS Latitude**<br>**Take coordinates of health post**	|___|___| : |___|___|___|___|___|	
110	**GPS Longitude**<br>**Take coordinates of health post**	|___|___| : |___|___|___|___|___|	
111	**Interviewer Initials**	|___|___|	
112	**Did you read the HEW the consent form?**	1 = Yes<br>2 = No	|___|
113	**Did the HEW agree to be interviewed?**	1= Yes – GO TO MODULE 2<br>2 = No	|___|
114	**If not, why not?**	|___________________________|	

MODULE 2: FACILITY, EQUIPMENT, MEDCINE AND JOB AIDS

INTERVIEWER: *I would now like to ask you questions about the facility, equipment, medicine and job aids at this health post.*

WALK AROUND THE FACILITY WITH THE HEW AND PERSONALLY CHECK THE AVAILABILITY OF EQUIPMENT, AND MEDICINE IN STOCK.

Does the facility have the following essential support services?

115	**What is the main source of drinking water?**	1 = Piped connection into health post<br>2 = Piped connection into yard<br>3 = Public standpipes<br>4 = Boreholes<br>5 = Protected dug wells<br>6 = Protected springs<br>7 = Rainwater collection<br>8 = Surface water<br>9 = Open dug wells<br>10 = Unprotected springs<br>11 = Vendor provided water<br>12 = Bottled water<br>13 = Tanker	|___|
116	**Water supply available on day of survey**	1 = Yes<br>2 = No	|___|
117	**Electricity connection or other power**	1 = Yes	|___|

	**sources (example, gas/solar generator)**	2 = No-**SKIP 119**		
118	**Electricity supply on day of survey?**	1 = Yes<br>2 = No		|___|
119	**Functional sterilizer that works on the day of the survey?**	1 = Yes<br>2 = No		|___|
120	**Functional fridge that works on the day of the survey?**	1 = Yes<br>2 = No		|___|
121	**Toilets accessible to facility users?**	1 = Yes<br>2 = No		|___|
122	**Generally is there a cell phone signal at this health post?**	1 = Yes<br>2 = No		|___|
123	**Is it all functional today?**<br>*INTERVIEWER -check your phone if there is signal on day of survey*	1 = Yes<br>2 = No		|___|
	**Which means of communication do you have to speak to the health facility?**<br><br>**Check all that apply**		For each:1 = Yes 2 = No	
		124	Facility landline/mobile phone	|___|
		125	Staff member mobile phone	|___|
		126	Phone outside the facility	|___|
		127	In person communication	|___|
128	**During the last sick newborn referral from the health post to health center, did you speak to the health facility directly?**	1 = Yes<br>2 = No – GO TO 130<br>3 =Don't know - GO TO 130		|___|
129	**IF YES, Which means of communication did you use?**	1 = Facility landline/mobile phone<br>2 = Staff member mobile phone;<br>3 = Phone outside the facility<br>4 = Radio<br>5 = In person communication;		|___|
130	**When referring from this health post to the health center for further care, do you use referral forms?**	1 = Yes (NOTE: check to see if an official referral form)<br>2 = No		|___|
131	**Do you receive any back referral forms on cases you have referred?**	1 = Yes<br>2 = No		|___|

**Walk around the facility with the respondent and personally check the availability of medicine**					
	**Does the facility have the following medicines today?**<br><br>*For each:*<br>*1 = Yes*<br>*2 = Not available*<br>*3= Expired*<br>*4 = Never available*			**If not available or expired, what was the duration of stock out /expiry for?**<br><br>*Check for the last three months and what was the longest number of consecutive days without that item (in days)*	
132	Vitamin k 1 mg	|___|	133	Vitamin k 1 mg	|__|__|__|
132A	Vitamin k 10 mg	|___|	132B	Vitamin k 10 mg	|__|__|__|
134	Vitamin A 200,000 IU	|___|	135	Vitamin A 200,000 IU	|__|__|__|
136	Vitamin A 100,000 IU	|___|	137	Vitamin A 100,000 IU	|__|__|__|
138	TTC eye ointment	|___|	139	TTC eye ointment	|__|__|__|
140	Chlorohexidine	|___|	141	Chlorohexidine	|__|__|__|

142	Gentamycin 20 mg/2ml, box of 50 amp	|___|	143	Gentamycin 20 mg/2ml, box of 50 amp	|__|__|__|
144	Gentamycin 80mg/2ml		145	Gentamycin 80mg/2ml	|__|__|__|
146	Amoxicillin suspension (125 mg/5 ml)	|___|	147	Amoxicillin suspension (125 mg/5 ml)	|__|__|__|
148	Amoxicillin tab 250 (dispersible)	|___|	149	Amoxicillin tab 250 (dispersible)	|__|__|__|
150	Amoxicillin tab 125 mg (dispersible)	|___|	151	Amoxicillin tab 125 mg (dispersible)	|__|__|__|
152	Ampicillin powder for inj, 500 mg	|___|	153	Ampicillin powder for inj, 500 mg	|__|__|__|
154	Paracetamol	|___|	155	Paracetamol	|__|__|__|
156	Iron	|___|	157	Iron	|__|__|__|
158	Folate	|___|	159	Folate	|__|__|__|
160	BCG	|___|	161	BCG	|__|__|__|
162	Polio vaccine	|___|	163	Polio vaccine	|__|__|__|
164	ORS	|___|	165	ORS	|__|__|__|
166	Zinc	|___|	167	Zinc	|__|__|__|
168	Zinc-ORS combined	|___|	169	Zinc-ORS combined	|__|__|__|
170	Malaria RDT	|___|	171	Malaria RDT	|__|__|__|
172	Coartem (Artemether/lumefantrine)	|___|	173	Coartem (Artemether/lumefantrine)	|__|__|__|
174	Chloroquine syrup	|___|	175	Chloroquine syrup	|__|__|__|
176	Artesunate suppository	|___|	177	Artesunate suppository	|__|__|__|
178	HIV diagnostic tests	|___|	179	HIV diagnostic tests	|__|__|__|
180	Cotrimoazole	|___|	181	Cotrimoazole	|__|__|__|
182	Plumpy nut	|___|	183	Plumpy nut	|__|__|__|
184	BP100	|___|	185	BP100	|__|__|__|

	**Does the facility have the following functional equipment today?**		*For each 1=yes, 2=no*	
		186	Ambu bag / face mask (full size 0 and 1)	|___|
		187	Any Thermometer	|___|
		188	Infant scale	|___|
		189	Weighing sling	|___|
		190	Blood pressure cuff	|___|
		191	Stethoscope	|___|
		192	Watch or clock	|___|
		193	Tape measure	|___|
		194	Examination couch	|___|
		195	Drape	|___|
		196	Washable mackintosh	|___|
		197	Dustbin	|___|

		198	Cups/drinking water	|___|
		199	Sharps container	|___|
		200	Chlorine bleach	|___|
		201	Bucket for decontamination solution	|___|
		202	Contaminated waste container	|___|
		203	Soap and towel or handrub	|___|
		204	Alcohol-based hand rub	|___|
		205	Clean glove	|___|
		206	Syringe with needle for Gentamycin injection	|___|
		207	MUAC tape measure	|___|
	**Does the facility have the following job aids and forms today?**		*For each 1=yes, 2=no*	
		208	Pregnant woman registration book	|___|
		209	ANC Register	|___|
		210	Delivery Register	|___|
		211	PNC Register	|___|
		212	ICCM registration book for 0- under 2 months	|___|
		213	ICCM registration book 2 -59 months	|___|
		214	Family health cards	|___|
		215	Vaccination cards	|___|
		216	Family folder	|___|
		217	Stock card/bin card	|___|
		218	HMIS forms (monthly and quarterly reporting)	|___|
		219	Request and re-supply form	|___|
		220	Chart booklet	|___|

**MODULE 3: HEALTH POST REGISTER REVIEW BY THE DATA COLLECTOR**		
PLEASE LOOK AT THE SPECIFIED HEW REGISTERS DETAILED BELOW FOR THE DIFFERENT DATA ELEMENTS.<br>WRITE 9999, 999 OR 99 IF NOT AVAILABLE.<br>*I would now like to take a look at your registers to abstract information about the community in this kebele and the services provided them. I will ask about the population profile for the last 12 months and services provided by you for the last three months from _________ to ________.*		
	**Obtain data on population FOR THE YEAR from the day of survey from Health Post wall records**	
221	Number of people in the kebele	|__|__|__|__|
222	Number of households in the kebele	|__|__|__|__|
223	Total number of women of reproductive age	|__|__|__|__|
224	Total number of under 5 children in the kebele	|__|__|__|__|
	**Obtain data on expected number of pregnancies and births from the PAST QUARTER from Health Post wall records**	
225	Expected number pregnancies	|__|__|__|
226	Expected number of births	|__|__|__|

227	Expected number of facility births	|__|__|__|
	**Obtain data from ANC registers for the PAST QUARTER**	
228	Number of women receiving 1 visit	|__|__|
229	Number of women receiving 2 visits	|__|__|
230	Number of women receiving 3 visits	|__|__|
231	Number of women receiving 4 visits	|__|__|
	**Obtain data from delivery registers for the PAST QUARTER**	
232	Number of births attended by the HEW	|__|__|
233	Number of total births (home, health post, health center, hospital)	|__|__|__|
234	Number of live births	|__|__|
235	Number of newborn deaths (28 days or less)	|__|__|
	**PNC data**	
236	Is there a Post Natal Care register (standard or otherwise) in this health post 1= Yes, 2=No	|__|
	**If Post Natal Care register is not available but family folder is available, obtain the information from the family folder given for the PAST QUARTER. Ask the HEWs to separate those.**	
237	Number receiving 1 visit	|__|__|
238	Number receiving 2 visits	|__|__|
239	Number receiving 3 visits	|__|__|
	**If information is not available from register books on the following, enquire from the HEW where to obtain the following information for the PAST QUARTER**	
240	Number of newborns treated for asphyxia, initial stimulation, or resuscitation by the HEW	|__|__|
241	Number of newborns given chlorohexidine cord care by the HEW	|__|__|
	**Obtain data from the ICCM 2-59 month register for the PAST QUARTER**	
242	Number of initial consultations with children	|__|__|__|
243	Number of sick children who were referred	|__|__|__|
244	Number of children classified as having pneumonia	|__|__|__|
245	Number of children classified as having diarrhea	|__|__|__|
246	Number of children classified as having malaria	|__|__|__|
247	Number of children who received antibiotic for pneumonia	|__|__|__|
248	Number of children who received ORS for diarrhea	|__|__|__|
249	Number of children who received zinc for diarrhea	|__|__|__|
250	Number of children who received zinc-ORS combined for diarrhea	|__|__|__|
251	Number of children who received RDT for malaria	|__|__|__|
252	Number of children who received ACT for malaria	|__|__|__|

Obtain data from iCCM 0-2 month registration book for information below for the PAST QUARTER
For each of the newborn less than 2 months old seen at the health center (recorded above) complete a separate record review.

Record 1

253A	How many 0-2 months were seen in the last quarter?		|___|___|___|
253	**Name of child**	______________ First name<br>______________ Last name	
254	**Address of child**	______________ Gote name<br>______________ Keble name	
255	**Date Seen**	*Gregorian calendar (DD/MM/YY)*	|__|__|/|__|__|/|__|__|
256	**Age of baby at the time of consultation in weeks**	*Record age of baby in weeks ranging from 1-8 weeks.*<br>*If unknown 9*	|___| weeks
257	**Gender of baby**	*1 = Male*<br>*2 = Female*	|___|
258	**Weight on the day of consultation in grams**	*If weight is given in KGs record in grams e.g 3.5 KG = 3500 grams.*<br>*If unknown 9999*	|___|___|___|___| grams
259	**Birth Weight**<br>**(Written for those less than 7 days)**	*1= < 1,500 grams*<br>*2= 1,500 - < 2,500 grams*<br>*3= >/= 2,500 grams*<br>*9= Unknown*	|___|
260	**Gestational Age (in weeks)**	*1= < 32 weeks*<br>*2= 32 – 36 weeks*<br>*3= >/= 37 weeks*<br>*9= Unknown*	|___|
261	**Temperature on the day of consultation in degree Celsius**	*Record temperature to one decimal place (e.g. 34.3 ℃)*<br>*If unknown 99.9*	|___|___|.|___|℃
262	**Respiratory Rate per minute on the day of consultation**	*If unknown 999*	|___|___|___|

	**Signs and symptoms of the newborn at the time of consultation?**<br><br>**Record all that apply**		For each:1 = Yes 2 = No	
		263	Reduced feeding/unable to feed	|___|
		264	Convulsion	|___|
		265	Severe Chest in-drawing	|___|
		266	Vomiting	|___|
		267	Fever	|___|
		268	Diarrhea	|___|
		269	Fast breathing	|___|
		270	Coughing	|___|
		271	Grunting	|___|
		272	Skin pustules	|___|
		273	Yellow palms and soles	|___|
		274	Yellow eyes and skin	|___|
		275	Red umbilicus or draining pus	|___|
		276	Movement only when stimulated or no movement even when stimulated	|___|
		277	Movement only when stimulated or no movement even when stimulated	|___|

		278	Bulging fontanelle	|___|
		279	Restless/Irritable	|___|
		280	Sunken eyes	|___|
		281	Skin pinch goes back slowly	|___|
		282	Skin pinch goes back very slowly	|___|
		283	Diarrhea lasting 14 days or more	|___|
		284	Blood in the stool	|___|
		285	Not suckling well	|___|
		286	Less than 8 breast feeds in 24 hours	|___|
		287	Switching to another breast before one is emptied	|___|
		288	Not breast feeding more frequently and longer during sickness	|___|
		289	Poor positioning during breast feeding	|___|
		290	Not well attached during breast feeding	|___|
		291	Receives other foods or drinks (even water)	|___|
		292	Low weight for age	|___|
		293	Thrush (ulcers or white patches in mouth)	|___|
		294	Signs and symptoms not given	|___|
		295	Other – GO TO 297	|___|
		296	Specify _______________	
	**Disease classification of the newborn**<br>**Record all that apply**		For each:1 = Yes 2 = No	
		297	Very Preterm and/or very low birth weight	|___|
		298	Preterm and/or low birth weight	|___|
		299	VSD	|___|
		300	Local bacterial infection	|___|
		301	Severe Dehydration	|___|
		302	Some Dehydration	|___|
		303	No Dehydration	|___|
		304	Severe Persistent Diarrhea	|___|
		305	Dysentery	|___|
		306	Jaundice	|___|
		307	Severe Jaundice	|___|
		308	Malaria	|___|
		309	Feeding problem or low weight	|___|
		310	Classification not given	|___|
		311	Other Go to 313	|___|
		312	Specify _______________	
	**Treatment given to the newborn**<br>**Record all that apply**		For each:1 = Yes 2 = No	
		313	Gentamycin IM first dose	|___|
		314	Gentamycin IM for seven days	|___|
		315	Amoxicillin suspension/dispersible first dose	|___|
		316	Amoxicillin suspension/dispersible for 7 days	|___|
		317	Amoxicillin suspension/dispersible for 5 days	|___|
		318	ORS (Plan B) – Facility treatment	|___|
		319	ORS (Plan A) – Home treatment	|___|
		320	Zinc for 10 days	|___|
		321	Zinc-ORS combined	|___|
		322	Oral chloroquine (Anti-malarial)	|___|
		323	Oral quinine (Anti-malarial)	|___|
		324	Oral coartem (Anti-malarial)	|___|
		325	Rectal Artesunate (Anti-malarial)	|___|
		326	IV Quinine (Anti-malarial)	|___|
		327	Other Antimalarial (specify)	|___|

		328	TTC (Tetracycline) eye ointment	|___|
		329	GV paint (Gentian Violet)	|___|
		330	Nutritional Counseling	|___|
		331	Exposing to sunshine 20– 30 minutes everyday	|___|
		332	Other treatment GO TO 334	|___|
		333	Specify ________________	
334	**Was newborn referred to a higher facility?**		1 = Yes – GO TO 337<br>2 = No	|___|
335	**If newborn had VSD and was treated at health post was gentamycin treatment completed?**		1 = Yes<br>2 = No<br>3 = Not VSD case	|___|
	**Outcome of the newborn treatment**		For each:1 = Yes 2 = No	
		336	Health improved/healed	|___|
		337	Died	|___|
		338	Worsened	|___|
		339	Same	|___|
		340	Unknown	|___|

Obtain data from iCCM 2-59 registration book for information below for the PAST QUARTER
For the past 10 children seen at the health post (recorded above) complete a separate record review.

Record 1

341A	**How many 2-59 months were seen in the last quarter?**			|___|___|___|
341	**Name of child**	________________ First name<br>________________ Last name		
342	**Address of child**	________________ Gote name<br>________________ Keble name		
343	**Date Seen**	*Gregorian calendar (DD/MM/YY)*		|__|__|/|__|__|/|__|__|
344	**Age of child at the time of consultation**	*Record age of child in months.*<br>*If unknown 99*		|___|___| months
345	**Gender**	*1 = Male*<br>*2 = Female*		|___|
346	**Weight on the day of consultation in grams**	*If weight is given in KGs record in grams*<br>*e.g 3.5 KG = 3500 grams.*<br>*If unknown 9999*		|___|___|___|___| grams
347	**Temperature on the day of consultation in degree Celsius**	*Record temperature to one decimal place (e.g. 34.3 ℃)*<br>*If unknown 99.9*		|___|___|.|___|℃
348	**Respiratory Rate per minute on the day of consultation**	*If unknown 999*		|___|___|___|
	**Signs and symptoms of the child at the time of consultation?**<br><br>**Record all that apply**		For each:1 = Yes 2 = No	
		349	Reduced feeding/unable to feed	|___|
		350	Vomiting	|___|
		351	Convulsion	|___|
		352	Movement only when stimulated or no movement even when stimulated	|___|
		353	Movement only when stimulated or no movement even when stimulated	|___|
		354	Restless/Irritable	|___|
		355	Difficult breathing	|___|
		356	Fast breathing	|___|
		357	Coughing	|___|
		358	Severe Chest in-drawing	|___|
		359	Stridor	|___|
		360	Diarrhea	|___|
		361	Diarrhea lasting 14 days or more	|___|
		362	Blood in the stool	|___|
		363	Skin pinch goes back slowly	|___|
		364	Skin pinch goes back very slowly	|___|
		365	Fever	|___|
		366	Bulging fontanelle	|___|
		367	Rash	|___|
		368	Mouth ulcers	|___|
		369	Pus or clouding of cornea	|___|
		370	Perform malaria RDT	|___|
		371	Discharge / pus in the ear	|___|
		372	Swelling	|___|
		373	Visible severe wasting	|___|

		374	Palmor pallor	|___|
		375	Signs and symptoms not given	|___|
		376	Other – GO TO 377	|___|
		377	Specify ________________	
	**Disease classification of the child**<br>**Record all that apply**		For each:1 = Yes 2 = No	
		378	One or more danger signs (unable to drink or breastfeed, vomits everything, convulsions, movement only when stimulated or no movement even when stimulated	|___|
		379	Severe pneumonia/very severe disease	|___|
		380	Pneumonia	|___|
		381	Severe dehydration	|___|
		382	Some dehydration	|___|
		383	Severe persistent diarrhoea	|___|
		384	Persistent diarrhoea	|___|
		385	Dysentery	|___|
		386	Very severe febrile disease	|___|
		387	Malaria	|___|
		388	Fever, malaria unlikely	|___|
		389	Fever, no malaria	|___|
		390	Severe complicated measles	|___|
		391	Measles with eye/mouth complications	|___|
		392	Measles	|___|
		393	Acute ear infection	|___|
		394	Chronic ear infection	|___|
		395	Severe malnutrition	|___|
		396	Moderate malnutrition	|___|
		397	Severe anaemia	|___|
		398	Anaemia	|___|
		399	Vaccination status not up-to-date	|___|
		400	Vaccine(s) needed (specify)	|___|
		401	Vitamin A status not up-to-date	|___|
		402	Classification not given	|___|
		403	Other Go to 404	|___|
		404	Specify ________________	
	**Treatment given to the child**<br>**Record all that apply**		For each:1 = Yes 2 = No	
		405	ORS (Plan A) – Home treatment	|___|
		406	ORS (Plan B) – Facility treatment	|___|
		407	Zinc for ten days	|___|
		408	Oral coartem (Anti-malarial)	|___|
		409	Oral chloroquine (Anti-malarial)	|___|
		410	Oral quinine (Anti-malarial)	|___|
		411	Rectal Artesunate (Anti-malarial)	|___|
		412	IV Quinine (Anti-malarial)	|___|
		413	Other Antimalarial (specify)	|___|
		414	Cotrimoxazole	|___|
		415	Vitamin A	|___|
		416	Paracetamol	|___|
		417	Gentamycin IM first dose	|___|
		418	Gentamycin IM for seven days	|___|
		419	Amoxicillin suspension/dispersible first dose	|___|
		420	Amoxicillin suspension/dispersible for seven days	|___|
		421	Amoxicillin suspension/dispersible for five days	|___|

		422	TTC (Tetracycline) eye ointment	|___|
		423	Plumpy nut	|___|
		424	BP100	|___|
		425	Nutritional Counseling	|___|
		426	Other treatment GO TO 427	|___|
		427	Specify ________________	
428	**Was the child referred to a higher facility?**		1 = Yes<br>2 = No	|___|
	**Outcome of the child treatment**		For each:1 = Yes 2 = No	
		429	Health improved/healed	|___|
		430	Died	|___|
		431	Worsened	|___|
		432	Same	|___|
		433	Unknown	|___|
