# Supplementary material for: Health postservice readiness and use of preventive and curative services for suspected childhood pneumonia in Ethiopia: a cross-sectional study
Source: BMJ Open. 2022 Apr 26;12(4):e058055. doi: 10.1136/bmjopen-2021-058055 (PMC9047705; doi:10.1136/bmjopen-2021-058055)
Supplement: Supplementary data [file bmjopen-2021-058055supp003.pdf]

OHEP HEW Questionnaire

Unique ID-----------

100	**Date (dd/mm/yyyy) Gregorian Calendar**	|___|___| / |___|___| / |___|___| dd / mm / yy	
101	**Region Name**	|__|__|__|__|__|__||__|__|__|__|	
102	**Zone Name**	|__|__|__|__|__|__||__|__|__|__|	
103	**Woreda Name**	|__|__|__|__|__|__||__|__|__|__|	
104	**PHCU/Health Center Name**	|__|__|__|__|__|__||__|__|__|__|	
104A	**Health post code**	|__|	
104B	**HEW code**	|__|	
105	**Kebele**	|__|__|__|__|__|__||__|__|__|__|	
106	**Gote**	|__|__|__|__|__|__||__|__|__|__|	
107	**Cluster Code**	|___|___|	
108	**GPS Latitude**	|___|___| : |___|___|___|___|___|	
109	**GPS Longitude**	|___|___| : |___|___|___|___|___|	
ELEVATION	**Elevation**		
110	**Interviewer Initials**	**|___|___|**	
111	**Did you read the HEW the consent form?**	1 = Yes<br>2 = No	|___|
112	**Did the HEW agree to be interviewed?**	1= Yes – GO TO MODULE 2<br>2 = No	|___|
113	**If not, why not?**	_________________________________ **END**	

Tariku A, *et al. BMJ Open* 2022; 12:e058055. doi: 10.1136/bmjopen-2021-058055

Module: 2

114	**What is your name?**		
115 Y	**What is your date of birth?**	yyyy |___|__|___|__| <br>Ethiopian Calendar	
115 M	**What is your date of birth?**	mm|___|__| Ethiopian Calendar	
115 D	**What is your date of birth?**	dd |___|__| Ethiopian Calendar	
116	**What is the number of years you attended school?**	Write number of years	|___||___| Years
117	**As an HEW, what is your level?**	1 = Level 1<br>2 = Level 2<br>3 = Level 3<br>4 = Level 4	|___|
118	**Do you have any specific qualification in addition to HEW training?**	1 = Yes<br>2 = No – GO TO 120	|___|
119	**If yes, specify**	_________________________	
120 YY	**For how long have you worked as an HEW (including work at other kebeles)?**	Write number of years.	|___||___| Years
120MM	If less than one year, enter number of months only.		|___||___| Months
121YY	**For how long have you worked as an HEW in this Health post?**	Write number of years and months.	|___||___| Years<br>|___||___| Months
121MM	If less than one year, enter 00 years and number of months		
122	**Do you reside in this kebele?**	1 = Yes<br>2 = No Skip to 124	|___|
123	**Was a home provided to you by the kebele?**	1 = Yes<br>2 = No	|___|
124	**How many HEWs work in this health post?**	Enter number, including the person being interviewed	|___|
125	**In this health post who is the HEW in charge (senior HEW)?**	1 = Myself<br>2 = Other HEW<br>3 = Neither of us	|___|

126	**How many days a week is the health post facility functionally open by at least 1 HEW?**	Enter number of days		|___|
127	**Do you post the health post functional days for clients to see?**	1 = Yes<br>2 = No		|___|
128	**Do you post the health posts hours of operation for clients to see?**	1 = Yes<br>2 = No		|___|
**During the weekend and public holidays- where do the residents of the kebele seek medical care?**<br><br>Select all that apply			For each: 1 = Yes 2 = No	
		129	**Health center**	|___|
		130	**Health Post**	|___|
		131	**With HEW (at her house or elsewhere)**	|___|
		132	**Pharmacy**	|___|
		133	**Traditional Healers**	
		134	**Other –**	|___|
		135	Specify ______________________________	

Instruction: From Question HEW_132-HEW_207
Don’t prompt. Select all mentioned

**What are the main components of immediate newborn care?**<br><br>Do not prompt<br>Select all mentioned.	**For each: 1 = Yes 2 = No**		
	136	Deliver baby onto mother’s abdomen	|___|
	137	Dry and wrap baby	|___|
	138	Assess breathing	|___|
	139	Delay cord clamping for three minutes	|___|
	140	Tie and cut cord appropriately	|___|
	141	Skin to skin contact	|___|
	142	Initiate breastfeeding	|___|
	143	Apply TTC eye ointment	|___|
	144	Apply chlorohexidine on cord	|___|
	145	Give Vitamin K	|___|
	146	Weight baby	|___|
	999	None mentioned from the list	
**What are the main components of the <u>first PNC visit</u> for newborn?**	**For each: 1 = Yes 2 = No**		
	147	Advice washing hands before touching baby	|___|

Do not prompt<br>Select all mentioned	148	Check for danger sings	|___|
	149	Check for congenital abnormalities	|___|
	150	Measure temp	|___|
	151	Measure weight	|___|
	152	Apply TTC eye ointment	|___|
	153	Encourage exclusive breast feeding for baby	|___|
	154	Advice to delay bathing of baby for 24 hrs	|___|
	155	Encourage skin to skin contact	|___|
	156	Provide cord care (Chlorohexidine)	|___|
	157	Education on appropriate cord care (Chlorohexidine)	|___|
	158	Vaccinate for polio and BCG	|___|
	159	Teach mother on how to recognize newborn danger signs using family health card.	|___|
	999	None mentioned from the list	
**What are the main components of <u>subsequent (3rd and 7th day and 6th week) PNC visits</u> for newborn**?<br><br>Do not prompt<br>Select all mentioned	**For each: 1 = Yes 2 = No**		
	160	Check for newborn danger signs	|___|
	161	Advice to keep cord clean	|___|
	162	Assess breastfeeding	|___|
	163	Advise on breastfeeding	|___|
	164	Ensure baby is kept warm	|___|
	165	Check baby's weight	|___|
	166	Vaccination	|___|
	999	None mentioned from the list	
**When a newborn weighs less than 1.5 kgs or has a gestational age of less than 32 weeks, what special care do you provide?**<br><br>Do not prompt<br>Select all mentioned	**For each: 1 = Yes 2 = No**		
	167	Continue feeding with expressed breast milk	|___|
	168	Monitor ability to breastfeed	|___|
	169	Cover baby well including head	|___|
	170	Hold close to mother	|___|
	171	Refer urgently with mother to health center or hospital	|___|
	999	None mentioned from the list	
**When a newborn weighs between1.5 - 2.5 kgs or has a gestational age of 32-<37 weeks, what special care do you provide?**<br><br>Do not prompt<br>Select all mentioned	**For each: 1 = Yes 2 = No**		
	172	Make sure the baby is warm	|___|
	173	Educate on optimal breastfeeding	|___|
	174	Monitor ability to breastfeed	|___|
	175	Monitor baby for the first 24 hours	|___|
	176	Educate on infection prevention	|___|
	999	None mentioned from the list	
**What are the main signs for good attachment**	**For each: 1 = Yes 2 = No**		

**during breast feeding?**<br><br>Do not prompt<br>Select all mentioned	177	Chin touching breast	|___|
	178	Mouth open wide	|___|
	179	Lower lip turned out	|___|
	180	More areola showing above	|___|
	999	None mentioned from the list	
**How do you determine feeding problems in a newborn?**<br><br>Do not prompt<br>Select all mentioned	**For each: 1 = Yes 2 = No**		
	181	Not well-attached to breast	|___|
	182	Not suckling effectively	|___|
	183	Less than 8 breastfeeds in 24 hours	|___|
	184	Switching to another breast before one is emptied	|___|
	185	Receives other foods or drinks (even water)	|___|
	186	Underweight for age	|___|
	187	Thrush (ulcers or white patches in mouth)	|___|
	999	None mentioned from the list	
**When a newborn shows signs of feeding problems or is underweight, what initial steps do you take?**<br><br>Do not prompt<br>Select all mentioned	**For each: 1 = Yes 2 = No**		
	188	Advise mother to breastfeed as often and as long as infant wants in 24 hours	|___|
	189	Teach mother correct positioning and attachment	|___|
	190	Educate on exclusive breastfeeding	|___|
	191	Teach the mother to treat thrush at home	|___|
	192	Follow-up any feeding problem	|___|
	193	Follow-up any thrush in two days	|___|
	194	Follow-up underweight for age in 14 days	|___|
	999	None mentioned from the list	
**What are the main signs for very severe disease in newborns?**<br><br>Do not prompt<br>Select all mentioned	**For each: 1 = Yes 2 = No**		
	195	Convulsions	|___|
	196	Stopped feeding or significantly reduced feeding	|___|
	197	Severe chest in drawing	|___|
	198	Fast breathing	|___|
	199	Temperature with 37.5 or more (warm)<br>*(Note: if high temperature only mentioned ask for clarification to what extent)*	|___|
	200	Temperature less than 35.5 (cold)<br>(*Note: if low temperature only mentioned ask for clarification to what extent*)	|___|
	201	No or very limited movement on stimulation	|___|

	999	None mentioned from the list	
**When the newborn presents sign of very severe disease, what initial steps do you take?**<br><br>Do not prompt<br>Select all mentioned	**For each: 1 = Yes 2 = No**		
	202	Continue to breastfeed or if unable to suck give breast milk that has been expressed	|___|
	203	Begin a dose of amoxicillin (pre-referral)	|___|
	204	Begin a dose of gentamycin antibiotics (pre-referral)	|___|
	205	Refer URGENTLY	|___|
	206	When referral is not possible treat with/prescribe amoxicillin for 7 days	|___|
	207	When referral is not possible treat with gentamycin daily for 7 days	|___|
	999	None mentioned from the list	
**What are the main signs for local bacterial infection in newborns?**<br><br>Do not prompt<br>Select all mentioned	**For each: 1 = Yes 2 = No**		
	208	Umbilicus red	|___|
	209	Umbilicus draining pus	|___|
	210	Skin pustules	|___|
	999	None mentioned from the list	
**When the newborn presents signs of local bacterial infection, what initial steps do you take?**<br><br>Do not prompt<br>Select all mentioned	**For each: 1 = Yes 2 = No**		
	211	Give amoxicillin syrup for 5 days	|___|
	212	Follow up care on 2nd day from initial visit	|___|
	213	Advice mother when to return	|___|
	214	Breastfeed more frequently	|___|
	215	Advice mother to give breast milk more frequently	|___|
	216	Advice mother to keep baby warm	|___|
	999	None mentioned from the list	
217	Are there any possible side effects of using injectable gentamicin for neonatal illness?	**1= Yes –**<br>**2 = No – Skip to 228**	|___|
	**For each: 1 = Yes 2 = No**		
218	What are the possible side effects of using inject able gentamicin for neonatal illness?		
**What are the possible side effects of using injectable gentamicin for neonatal illness?**	218	Kidney damage (nephropathy)	|___|
	219	Nerve damage (neuropathy especially hearing or visual damage)	|___|
	220	Hearing loss	|___|
	221	Lethargy	|___|
	222	Nausea/vomiting	|___|

	223	General anaphylactic reaction	|___|
	224	Fever	|___|
	225	Poor appetite	|___|
	226	Weight loss	|___|
	227	Skin rash	|___|
228	Is there any contraindication of using injectable gentamicin for the neonatal illness?	1= Yes<br>2 = No – 232	
**What are those possible contraindications of using injectable gentamicin for the neonatal illness?**		**For each: 1 = Yes 2 = No**	
	229	History of general body reaction or shock to injectable gentamicin (Anaphylactic reaction	|___|
	230	History of kidney/urine problem	|___|
	231	History of skin reaction to gentamicin	|___|
232	**Are there any possible side effects of using amoxicillin for the neonatal illness?**	**1= Yes**<br>**2 = No – 234**	|___|
		**For each: 1 = Yes 2 = No**	
233	**What are the possible side effects of using amoxicillin for the neonatal illness?**	1 = General anaphylactic reaction (penicillin hypersensitivity)<br>9.Not mentioned	|___|
234	**Is there any contraindication of using amoxicillin for the neonatal illness?**	**1= Yes**<br>**2 = No – GO TO 236**	|___|
		**For each: 1 = Yes 2 = No**	
235	**What are those possible contraindications of using amoxicillin for the neonatal illness**	1 = History of General body reaction or shock to amoxicillin (penicillin hypersensitivity)<br>9.Not mentioned	|___|
236	**Are there any possible additional side effects of using antibiotics (injectable gentamicin or amoxicillin) for non-severe neonatal illness?**	**1= Yes**<br>**2 = No – 238**	|___|
		**For each: 1 = Yes 2 = No**	
237	**What are those additional side effects of using antibiotics (injectable gentamicin or amoxicillin) for non-severe neonatal illness?**	1 = Drug resistance<br>9.Not mentioned	|___|
**What are the main signs for jaundice in newborns?**<br><br>Do not prompt<br>Select all mentioned		**For each: 1 = Yes 2 = No**	
	238	Yellow skin	|___|
	239	Yellow eyes	|___|
	999	Not mentioned	
**When the newborn presents signs of jaundice, what initial steps do you take?**<br><br>Do not prompt<br>Select all mentioned		**For each: 1 = Yes 2 = No**	
	240	Breastfeed more frequently	|___|
	241	Advise mother to keep young infant warm	|___|
	242	Expose to sunshine 20 to 30 minutes every day	|___|

	243	Advise mother to return immediately if sign & symptoms of jaundice aggravates	|___|
	244	Follow-up in 2 days	|___|
**What are the main symptoms/signs for severe jaundice in newborns?**<br><br>Do not prompt<br>Select all mentioned	**For each: 1 = Yes 2 = No**		
	245	Jaundice in newborns of Age 14 days or more	|___|
	246	Jaundice in newborns of Age less than 24 hours	|___|
	247	Palms yellow	|___|
	248	Soles yellow	|___|
	999	Not mentioned from the list	
**When the newborn presents symptoms /signs of severe jaundice what initial steps do you take?**<br><br>Do not prompt<br>Select all mentioned	**For each: 1 = Yes 2 = No**		
	249	Breastfeed more frequently	|___|
	250	Refer URGENTLY to health center /hospital	|___|
	251	Keep the baby warm	|___|
**What are the main signs for some dehydration caused by diarrhea in newborns?**<br><br>Do not prompt<br>Select all mentioned	**For each: 1 = Yes 2 = No**		
	252	Restless and irritable	|___|
	253	Sunken eyes	|___|
	254	Skin pinch goes back slowly	|___|
	999	None mentioned from the list	
**When the newborn presents signs of some dehydration caused by diarrhea what initial steps do you take?**<br><br>Do not prompt<br>Select all mentioned	**For each: 1 = Yes 2 = No**		
	255	Give ORS fluids	|___|
	256	Give zinc treatment for 10 days	|___|
	257	Advise mother to breastfeed more frequently and longer	|___|
	258	Keep the infant warm	|___|
	259	Advise mother when to return	|___|
	260	Follow up in 2 days	|___|
	999	None mentioned from the list	
**What are the main symptoms/signs for severe dehydration caused by diarrhea in newborns?**<br><br>Do not prompt<br>Select all mentioned	**For each: 1 = Yes 2 = No**		
	261	Limited or No movement even when stimulated	|___|
	262	Sunken eyes	|___|
	263	Skin pinch goes back VERY slowly	|___|
	999	None mentioned from the list	
**When the newborn presents signs of severe dehydration caused by diarrhea what initial steps do you take?**<br><br>Do not prompt<br>Select all mentioned			
	264	Give first dose of amoxicillin syrup	|___|
	265	Give first dose of IM Gentamycin	|___|
	266	Refer URGENTLY to health center/hospital	|___|
	267	Ensure mother gives child ORS on the way to health center/hospital	|___|
	268	Advise mother to breastfeed more	|___|

		frequently and longer	
	269	Advice mother to keep young infant warm	|___|
**What are the main signs and symptoms of pneumonia in a child aged 2-59 months?**<br><br>Do not prompt<br>Select all mentioned	**For each: 1 = Yes 2 = No**		
	270	Cough	|___|
	271	Difficult or fast breathing	|___|
	272	Chest indrawing	|___|
	273	Stridor	|___|
	999		
**When the child presents signs and symptoms of pneumonia what initial steps do you take?**<br><br>Do not prompt<br>Select all mentioned	**For each: 1 = Yes 2 = No**		
	274	Give antibiotics	|___|
	275	Advise on when/how to administer	|___|
	276	Keep the child warm	|___|
	277	Advise mother when to return	|___|
	999	None mentioned from the list	
**What are the main signs and symptoms of diarrhea in a child aged 2-59 months?**<br><br>Do not prompt<br>Select all mentioned	**For each: 1 = Yes 2 = No**		
	278	Diarrhea	
	279	Blood in stool	
	280	Restless or irritable	
	281	Sunken eyes	|___|
	282	Skin pinch goes back slowly	|___|
	999	None mentioned from the list	
**When the child presents signs and symptoms of diarrhea what initial steps do you take?**<br><br>Do not prompt<br>Select all mentioned	**For each: 1 = Yes 2 = No**		
	283	Give ORS fluids	|___|
	284	Give zinc treatment for 10 days	|___|
	285	Advise on when/how to administer	
	286	Advise mother to breastfeed / feed more frequently and longer	|___|
	287	Keep the child warm	|___|
	288	Advise mother when to return	|___|
	289	Follow up in 2 days	
	999	None mentioned from the list	
**What are the main signs and symptoms of malaria in a child aged 2-59 months?**<br><br>Do not prompt<br>Select all mentioned	**For each: 1 = Yes 2 = No**		
	290	Fever	|___|
	291	Chillis	|___|
**When the child presents signs and symptoms of malaria what initial steps do you take?**<br><br>Do not prompt<br>Select all mentioned	**For each: 1 = Yes 2 = No**		
	292	Use RDT to test for malaria	|___|
	293	Treat malaria with Coartem / ACT	|___|
	294	Advise on when/how to administer Coartem	
	295	Advise mother when to return	|___|
	999	None mentioned from the list	

**What are the main signs and symptoms of acute malnutrition in a child aged 2-59 months?**<br><br>Do not prompt<br>Select all mentioned	**For each: 1 = Yes 2 = No**		
	296	Pitting edema of both feet	|___|
	297	Visible severe wasting	|___|
	298	MUAC measurement <11cm (if 6 months or older)	|___|
	999	None mentioned from the list	
**When the child presents signs and symptoms of acute malnutrition what initial steps do you take?**<br><br>Do not prompt<br>Select all mentioned	**For each: 1 = Yes 2 = No**		
	299	Appetite test if 6 months or older	|___|
	300	Give with RUTF (Plumpy nut or BP 100)	|___|
	301	Advise on when/how to take RUTF	|___|
	302	Advise mother when to return	|___|
	999	None mentioned from the list	
**What are general danger signs in child aged 2-59 months?**<br><br>Do not prompt<br>Select all mentioned	**For each: 1 = Yes 2 = No**		
	303	Unable to drink or breastfeed	|___|
	304	Convulsions	|___|
	305	Movement only when stimulated or no movement even when stimulated	|___|
	999	None mentioned from the list	
**When the child presents with general danger signs and symptoms what initial steps do you take?**<br><br>Do not prompt<br>Select all mentioned	**For each: 1 = Yes 2 = No**		
	306	Refer to health centre	|___|
	307	Giver pre-refferal dose and refer to health center	|___|
	308	Give ORS	|___|
	999	None mentioned from the list	

MODULE 4: TRAINING OF THE HEW

Interviewer*: I would now like to ask you some questions on your training*.

INTERVIEWER: PLEASE STATE THE START AND END MONTHS THAT CAPTURE THE PAST 12 MONTHS FROM THE DATE OF THE INTERVIEW AND INSERT IT INTO THE QUESTION BELOW.

Have you received training or training update from HC, Woreda health office or NGO in the last 12 months between _____________ (start month) and _____________ (end month) in?
[READ TOPIC]
FOR EACH QUESTION IF NO ASK THE FOLLOWING: *Did you receive a training or training update more than 12 months ago?*
REPEAT BOTH QUESTIONS FOR EACH TOPIC

		Yes, within past 12 months	Yes, over past 12 months ago	No in-service training or update	
309	CBNC	1	2	3	|____|

310	Providing antenatal services	**1**	**2**	**3**	|____|
311	PMTCT	**1**	**2**	**3**	|____|
312	Misoprostol use	**1**	**2**	**3**	|____|
313	Providing post-natal care to mother	**1**	**2**	**3**	|____|
314	Providing postnatal care to newborn	**1**	**2**	**3**	|____|
315	Clean cord care	**1**	**2**	**3**	|____|
316	Managing newborns with very severe disease	**1**	**2**	**3**	|____|
317	Managing newborn with local bacterial infection	**1**	**2**	**3**	|____|
318	Managing newborn neonates with jaundice/severe jaundice	**1**	**2**	**3**	|____|
319	Managing neonates with diarrhea	**1**	**2**	**3**	|____|
320	Managing neonates with feeding problem or who are underweight	**1**	**2**	**3**	|____|
321	iCCM	**1**	**2**	**3**	|____|
322	Using referral forms for VSD	**1**	**2**	**3**	|____|
323	Using/filling family folder	**1**	**2**	**3**	|____|
324	Integrated Refresher training on MNCH services	**1**	**2**	**3**	|____|
325	EPI	**1**	**2**	**3**	|____|
326	Can you tell us whether or not you were satisfied with the quality of training received for managing sick neonate?<br><br>**Do not give options to the respondent**	**1 = Yes was satisfied**<br>**2 = No was not satisfied – GO TO 328**<br>**3 = Neither satisfied nor dissatisfied – GO TO 329**<br>**4= No training in the last 12 months –GO TO 335**			|____|
327	**IF YES,** then what was the level of satisfaction<br><br>**Give both options to the respondent**	**1. Fully satisfied – GO TO 329**<br>**2. Somewhat satisfied – GO TO 329**			|____|
328	**IF NO,** then what was the level of dissatisfaction<br><br>**Give both options to the respondent**	**1. Fully dissatisfied**<br>**2. Somewhat dissatisfied**			|____|

How can the quality of the training be further improved<br>**Read list.**<br>**Select all that apply.**	**For each: 1 = Yes 2 = No**		
	329	More training	|____|
	330	More practice sessions	|____|
	331	More training aids	|____|
	332	More post training supervision	|____|
	333	Other – **GO TO 334**	|____|
	334	Specify_________________________	

MODULE 5: SUPPORTIVE SUPERVISION

INTERVIEWER:
I would now like to ask some questions about <u>supportive supervision</u> you have received. By supportive supervision I mean being visited by individuals from the region, zone, woredaand/or health center to discuss, review and give feedback on your <u>TECHNICAL</u> or <u>PROFEESIONAL</u> work.
INTERVIEWER: PLEASE STATE THE START AND END MONTHS THAT CAPTURE THE PAST 6 MONTHS FROM THE DATE OF THE INTERVIEW AND INSERT IT INTO THE QUESTION BELOW.

<u>LAST 6 MONTHS: STATE THE START & END MONTHS</u>
START MONTH ________________
END MONTH ________________

335	Have you received a supportive supervisory visit in the last 6 months? .	**1 = Yes**<br>**2 = No – GO TO 366**		|____|
**IF YES**: Who from?<br><br>**Select all that apply**		**For each: 1 = Yes 2 = No**		
		336	Woreda health office	|____|
		337	PHCU/health centre	|____|
		338	NGO	|____|
339	**IF YES:** How many times did you receive this visit in the last 6 months?	**Enter total number of times**<br>**IF 0 skip to 342**		|____|
340	How many of these visits were in last 3 months?	**Enter total number of times**<br>**IF 0 skip to 342**		|____|
341	How many of these visits were in last 1 month?	**Enter total number of times**		|____|
342	Who provided the most recent supervisory visit?<br><br>**Select one**	**1 = Woreda Health Office**<br>**2 = Health Centre**<br>**3 = NGO**<br>**4 = Woreda Health Office and Health center**<br>**5 = Woreda Health Office and NGO**<br>**6 = Health Center and NGO**<br>**7 = All three together (Woreda, Health Center, NGO)**		|____|
**If YES to 335**: Did that visit include any of the following?<br><br>**Read all the following**		**For each: 1 = Yes 2 = No**		
		343	Discussing early identification of pregnancy	|____|
		344	Discussing provision focused ANC	|____|
		345	Discussing promotion of institutional delivery	|____|
		346	Discussing safe and clean delivery	|____|
		347	Discussing immediate newborn care including cord care (chlorohexidine)	|____|
		348	Discussing recognition of asphyxia, initial stimulation, and resuscitation of newborn babies	|____|
		349	Discussing prevention and management of hypothermia	|____|
		350	Discussing management of pre-term and/or low	|____|

			birth weight neonates	
		351	Discussing management of very severe disease in newborns	|____|
		352	Discussing diagnosis or treatment of suspected pneumonia	|___|
		353	Discussing diagnosis or treatment of diarrheoa	|___|
		354	Discussing diagnosis or treatment of malaria	|___|
		355	Discussing diagnosis or treatment of acute malnutrition	|____|
		356	Discussing HEW activities with WDA	|____|
		357	Observing record keeping	|____|
		358	Checking the register for consistency and completeness	|____|
		359	Checking supplies including training manuals, job aides, request forms	|____|
		360	Delivering supplies including /training manuals, job aides, request forms	|____|
		361	Observing client Consultation with HEW	|____|
		362	Conducted postnatal household visits together to observe HEWs skill on checking general danger signs	|____|
		363	Checking if they visited a sick neonate under treatment or that has been treated,	|____|
		364	Providing WRTTTEN feedback to you on your work	|____|
		365	WRTTTEN feedback: copy of the last visit available and checked by the interviewer	|____|
366	Did you receive a follow up visit within 6 weeks of CBNC training to assess and support your **CBNC work**?	**1 = Yes**<br>**2 = No – GO TO 371**<br>**3 = No CBNC training –GO TO 371**		|____|
If received CBNC post-training visit who conducted 6 weeks follow up visit?<br><br>**Select all that apply**		**For each: 1 = Yes 2 = No**		
		367	Zone	|____|
		368	Woreda	|____|
		369	Health Center	|____|
		370	NGO	|___|

371	Can you tell us whether or not you were satisfied with the QUALITY of supportive supervision received in last six months?<br><br>**Do not read options**	**1 = Yes was satisfied<br>2 = No was not satisfied – GO TO 373<br>3 = Neither satisfied nor dissatisfied – GO TO 374<br>4 = no supportive supervision in the last 6 months GO TO 379**		|___|
372	**IF YES,** then what was the level of satisfaction?<br><br>**Read options**	**1. Fully satisfied -GO TO 379<br>2. Somewhat satisfied -GO TO 374**		|___|
373	**IF NO,** then what was the level of dissatisfaction?<br><br>**Read options**	**1. Fully dissatisfied<br>2. Somewhat dissatisfied**		|___|
How can the quality of the supervision be further improved:<br><br>**Read list<br>Select all that apply**		**For each: 1 = Yes 2 = No**		
		374	More visits	|___|
		375	More crash trainings during supervision	|___|
		376	More technical supervision	|___|
		377	Other **can the quality of the supervision be further improved**	|___|
		378	Specify __________________	|___|

Interviewer:

I would now like to ask you some questions about Performance Review and Clinical Mentoring Meeting (PRCMM) By this I mean when NGO, health center and health post staff meet together to review records, discuss performance, and ways to improve your TECHNICAL skills and achieve targets for CBNC/ICCM?

INTERVIEWER: PLEASE STATE THE START AND END MONTHS THAT CAPTURE THE PAST 6 MONTHS FROM THE DATE OF THE INTERVIEW AND INSERT IT INTO THE QUESTION BELOW.

LAST 6 MONTHS: STATE THE START & END MONTHS

START MONTH ________________

END MONTH _______________

**379**	In the past 6 months, did you participate in a PRCMM meeting, where the health center, health post and/or NGO staff met together to discuss performance, targets, and ways to improve HEWs' skills and achieve targets for CBNC and/or ICCM?	**1 = Yes**<br>**2 = No**<br>**3 = No CBNC and/or ICCM training**	|____|
**380**	Since training of CBNC and/or ICCM, have you participated in any PRCM meeting conducted in your Health center catchment area?	**1 = Yes**<br>**2 = No – GO TO MODULE 6**	|____|
**381**	Did the meeting extract data from the HEW's 0-2 month (newborn) registers?	**1 = Yes**<br>**2 = No**	|____|
**382**	At that meeting, did your health center staff get a chance to offer mentoring on how to improve your newborn illness management skills?	**1 = Yes**<br>**2 = No**	|____|

Did that meeting cover discussions on performance and targets on the following?<br><br>**Read all the following**		**For each: 1 = Yes 2 = No**	
	383	Early identification of pregnancy	|____|
	384	Focused ANC	|____|
	385	Promotion of institutional delivery	|____|
	386	Safe and clean delivery	|____|
	387	Immediate newborn care including cord care (chlorohexidine)	|____|
	388	Recognition of asphyxia, initial stimulation and resuscitation of newborn babies	|____|
	389	Management of diarrhea among neonate	|____|
	390	Breast feeding among neonate	|____|
	391	Immunization among neonate	|____|
	392	Management of hypothermia	|____|
	393	Management of pre-term and/or low birth weight neonates	|____|
	394	Management of neonatal/very severe disease	|____|
	395	Register review	|____|
	396	Community level observation	|____|

MODULE 6: HEWS SERVICES PROVIDED IN THE LAST 3 MONTHS

Interviewer: ***I would now like to ask you about the services you provided in the last 3 months.***

REFER TO HEW'S RECORD BOOKS (AT THE HEALTH POST REGISTERS) TO COMPLETE THE FOLLOWING; ONLY COUNT EVENTS ATTENDED BY THE SPECIFIC HEW BEING INTERVIEWED:

INTERVIEWER: PLEASE STATE THE START AND END MONTHS THAT CAPTURE THE PAST 3 MONTHS FROM THE DATE OF THE INTERVIEW AND INSERT IT INTO THE QUESTION BELOW.
LAST 3 MONTHS: STATE THE START & END MONTHS
START MONTH ________________
END MONTH ________________

397	In the last three months did you use community forums and meeting to deliver maternal newborn and child health education?	**1 = Yes**<br>**2 = No – GO TO 406**	|___|

In the last three months did you meet any of the following to deliver maternal newborn and child health education?<br><br>**Read list. Choose all that apply.**	**For each: 1 = Yes 2 = No**		
	398	Religious leaders	|___|
	399	Edir (Traditional community organization whose members assist each other during the mourning process)	|___|
	400	Women's savings group	|___|
	401	Command post	|___|
	402	Traditional birth attendants	|___|
	403	Other	|___|
	404	Other Specify________________________	

405	When was the last time you used community forum and meeting to deliver maternal newborn and child health education?	|__||__|/|__||__|/|__||__| dd / mm / yy	
406	Did you conduct pregnant women conference in the community in the last 3 months?	**1 = Yes**<br>**2 = No – GO TO 411**	|___|
407	When was the last time you conducted a pregnant women's conference?	|__||__|/|__||__|/|__||__| dd / mm / yy	
408	How regularly do you conduct the pregnant women's conference?	**1 = Once a week**<br>**2 = Every two weeks**<br>**3 = Once a month**<br>**4 = Every other month**<br>**5 = Once every three months**	|___|

409	In the last pregnant women conference, how many women in your catchment area were pregnant?	**Enter number**<br>**Enter 999 if don't know**	|__|__|__|
410	Among them, how many of them attended the pregnant women's conference?<br>(The number should be $\leq$ numbers inserted in Q 408)	**Enter number**	|___|___|
411	Did you provide ANC to any women in the last three months?	**1 = Yes**<br>**2 = No – GO TO 412**	|___|
412	**IF YES:** how many?	**Enter number**	|___||___|
413	Did you refer any pregnant women from this health post to a health center or hospital in the last three months?	**1 = Yes**<br>**2 = No – GO TO 414**	|___|
414	**IF YES:** how many?	**Enter number**	|___||___|
415	Did you see any women to provide postpartum care in the last three months?	**1 = Yes**<br>**2 = No – GO TO 416**	|___|
416	**IF YES:** How many women did you see for postpartum care in the last three months?	**Enter number**	|___||___|
417	Did you refer any post-partum women from this health post to a health center or hospital in the past three months?	**1 = Yes**<br>**2 = No – GO TO 418**	|___|
418	**IF YES:** How many?	**Enter number**	|___||___|
419	Did you see any newborns to provide a postnatal check for in the last three months?	**1 = Yes**<br>**2 = No – GO TO 420**	|___|
420	**IF YES:** How many newborns did you provide a postnatal check for in the last three months?	**Enter number**	|___||___|
421	Did you refer any newborns from this health post to a health center or hospital in the past three months?	**1 = Yes**<br>**2 = No – GO TO 422**	|___|
422	**IF YES:** How many?	**Enter number**	|___||___|
423	Did you give care for prevention of hypothermia in the last three months?	**1 = Yes**<br>**2 = No – GO TO 424**	|___|
424	**IF YES**: How many?	**Enter number**	|___||___|
425	Did you give care for management of hypothermia in the last three months?	**1 = Yes**<br>**2 = No – GO TO 426**	|___|
426	**IF YES:** How many?	**Enter number**	|___||___|
427	Did you provide care for pre-term and/or low birth weight neonates in the last three months?	**1 = Yes**<br>**2 = No – GO TO 428**	|___|
428	**IF YES**: How many?	**Enter number**	|___|
429	Did you identify newborns with suspected very severe disease in the	**1 = Yes**<br>**2 = No – GO TO 430**	|___|

	last three months?		
430	**IF YES**: How many?	**Enter number**	|____|
431	Did you treat newborns with suspected very severe disease in the last three months?	**1 = Yes**<br>**2 = No – GO TO 433**<br>**3 = Antibiotics not available – GO TO 433**	|____|
432	**IF YES**: how many?	**Enter number**	|____|
433	**IF YES**: how many completed the treatment at the health post?	**Enter number**	|____|
434	Did you refer any newborns from this health post to a health center or hospital for very severe disease in the past three months?	**1 = Yes**<br>**2 = No – GO TO 435**	|____|
435	**IF YES**: How many?	**Enter number**	|____|
436	Did you see any newborns with diarrhea in the last three months?	**1 = Yes**<br>**2 = No – GO TO 437**	|____|
437	**IF YES**, how many?	**Enter number**	|____||____|
438	Did you see any newborns with jaundice in the last three months?	**1 = Yes**<br>**2 = No – GO TO 439**	|____|
439	**IF YES**, how many?	**Enter number**	|____||____|
440	Are the maternity record books completely up to date until the day before survey?(observe)	**1 = Yes**<br>**2 = No GO TO 442**	|____|
441	What is number of maternity cases maintained in the last three months in the maternity register?	**Enter number**	|____||____|
442	Did you see any children (2-59 months) for suspected pneumonia in the last three months?	**1 = Yes**<br>**2 = No – GO TO 443**	|____|
443	**IF YES**, how many?	**Enter number**	|____||____|
444	Did you see any children (2-59 months) for diarrhea in the last three months?	**1 = Yes**<br>**2 = No – GO TO 445**	|____|
445	**IF YES**, how many?	**Enter number**	|____||____|
446	Did you see any children (2-59 months) for malaria in the last three months?	**1 = Yes**<br>**2 = No – GO TO 447**	|____|
447	**IF YES**, how many?	**Enter number**	|____||____|
448	Did you see any children (2-59 months) for acute malnutrition in the last three months?	**1 = Yes**<br>**2 = No – GO TO 449**	|____|
449	**IF YES**, how many?	**Enter number**	|____||____|
450	Did you refer any children (2-59 months) from this health post to a health center or hospital for very severe disease in the past three months?	**1 = Yes**<br>**2 = No – Exit interview**	|____|
451	**IF YES**: How many?	**Enter number**	|____||____|

Tariku A, *et al. BMJ Open* 2022; 12:e058055. doi: 10.1136/bmjopen-2021-058055
