# Supplementary material for: Health postservice readiness and use of preventive and curative services for suspected childhood pneumonia in Ethiopia: a cross-sectional study
Source: BMJ Open. 2022 Apr 26;12(4):e058055. doi: 10.1136/bmjopen-2021-058055 (PMC9047705; doi:10.1136/bmjopen-2021-058055)
Supplement: Author's manuscript [file bmjopen-2021-058055.draft_revisions.pdf]

BMJ Open is committed to open peer review. As part of this commitment we make the peer review history of every article we publish publicly available.

When an article is published we post the peer reviewers' comments and the authors' responses online. We also post the versions of the paper that were used during peer review. These are the versions that the peer review comments apply to.

The versions of the paper that follow are the versions that were submitted during the peer review process. They are not the versions of record or the final published versions. They should not be cited or distributed as the published version of this manuscript.

BMJ Open is an open access journal and the full, final, typeset and author-corrected version of record of the manuscript is available on our site with no access controls, subscription charges or pay-per-view fees (http://bmjopen.bmj.com).

If you have any questions on BMJ Open's open peer review process please email info.bmjopen@bmj.com

BMJ Open

Health post service readiness and use of preventive and curative services for suspected childhood pneumonia in Ethiopia: a cross-sectional study

Journal:	*BMJ Open*
Manuscript ID	bmjopen-2021-058055
Article Type:	Original research
Date Submitted by the Author:	05-Oct-2021

Complete List of Authors:	Woldemariam, Amare; University of Gondar, Human Nutrition;<br>Berhane, Yemane; Addis Continental Institute of Public Health,<br>Worku, Alemayehu; Addis Ababa University, School of Public Health<br>Biks, Gashaw ; University of Gondar, Public Health ; NA<br>Persson, Lars; London School of Hygiene and Tropical Medicine,<br>Okwaraji, Yemisrach; London School of Hygiene & Tropical Medicine, Epidemiology and Population Health; 74B Beaconfield Road,

Keywords:	Public health < INFECTIOUS DISEASES, Community child health < PAEDIATRICS, Respiratory infections < THORACIC MEDICINE, Health policy < HEALTH SERVICES ADMINISTRATION & MANAGEMENT

SCHOLARONE™
Manuscripts

I, the Submitting Author has the right to grant and does grant on behalf of all authors of the Work (as defined in the below author licence), an exclusive licence and/or a non-exclusive licence for contributions from authors who are: i) UK Crown employees; ii) where BMJ has agreed a CC-BY licence shall apply, and/or iii) in accordance with the terms applicable for US Federal Government officers or employees acting as part of their official duties; on a worldwide, perpetual, irrevocable, royalty-free basis to BMJ Publishing Group Ltd ("BMJ") its licensees and where the relevant Journal is co-owned by BMJ to the co-owners of the Journal, to publish the Work in this journal and any other BMJ products and to exploit all rights, as set out in our licence.

The Submitting Author accepts and understands that any supply made under these terms is made by BMJ to the Submitting Author unless you are acting as an employee on behalf of your employer or a postgraduate student of an affiliated institution which is paying any applicable article publishing charge ("APC") for Open Access articles. Where the Submitting Author wishes to make the Work available on an Open Access basis (and intends to pay the relevant APC), the terms of reuse of such Open Access shall be governed by a Creative Commons licence – details of these licences and which Creative Commons licence will apply to this Work are set out in our licence referred to above.

Other than as permitted in any relevant BMJ Author's Self Archiving Policies, I confirm this Work has not been accepted for publication elsewhere, is not being considered for publication elsewhere and does not duplicate material already published. I confirm all authors consent to publication of this Work and authorise the granting of this licence.

For peer review only

Health post service readiness and use of preventive and curative services for suspected childhood pneumonia in Ethiopia: a cross-sectional study

Amare Tariku[1,2*], Yemane Berhane[2], Alemayehu Worku[2,3], Gashaw Andargie Biks[4], Lars Åke Persson[5,6], Yemisrach B. Okwaraji[5,6]

[1]Department of Human Nutrition, Institute of Public Health, College of Medicine and Health Sciences, University of Gondar, Gondar, Ethiopia

[2]Department of Epidemiology and Biostatistics and Department of Reproductive Health and Population, Addis Continental Institute of Public Health, Addis Ababa, Ethiopia

[3]School of Public Health, Addis Ababa University, Addis Ababa, Ethiopia

[4]Department of Health System and Policy, Institute of Public Health, College of Medicine and Health Sciences, University of Gondar, Gondar, Ethiopia

[5]London School of Hygiene & Tropical Medicine, London, UK

[6]Ethiopian Public Health Institute, Addis Ababa, Ethiopia

Co-authors;

Yemane Berhane, MD, PhD: yemaneberhane@addiscontinental.edu.et

Alemayehu Worku, PhD: alemayehuwy@yahoo.com

Gashaw Andargie Biks, PhD: gashawab@gmail.com

Lars Åke Persson, MD, PhD: Lars.Persson@lshtm.ac.uk

Yemisrach B. Okwaraji, PhD: yemisrach.okwaraji@lshtm.ac.uk

[*]Corresponding author:

Amare Tariku, MSc; amaretariku15@yahoo.com

For peer review only

For peer review only - http://bmjopen.bmj.com/site/about/guidelines.xhtml

ABSTRACT

Objective: Pneumonia is the single leading cause of infectious disease deaths in children under-five. Despite this challenge, the utilization of preventive and curative child health services remains low in Ethiopia. We investigated the association between health post service readiness and caregivers' awareness of pneumonia services, care-seeking, and utilisation of pneumonia-relevant immunisation in four Ethiopian regions.

Design and setting: This cross-sectional study was conducted from December 2018 to February 2019. The readiness of health posts for sick child care was assessed using the World Health Organization Health Service Availability and Readiness Assessment tool. A multilevel analysis was employed to examine the association between health post readiness and household level awareness and utilisation of services.

Participants: We included 165 health posts, 274 health extension workers (community health workers), and 4,729 caregivers with 5,787 children 2-59 months.

Outcome measures: Awareness of pneumonia treatment, care-seeking behaviour, and pentavalent-3 immunisation.

Results: Only 62.8% of health posts were ready to provide sick child care services. Approximately one-quarter of caregivers were aware of pneumonia services, and 56.8% sought an appropriate care provider for suspected pneumonia. Nearly half (49.3%) of children 12-23 months had received three doses of pentavalent vaccine. General health post readiness was not associated with the caregiver's awareness of pneumonia treatment [AOR= 0.9, 95% CI 0.7 to 1.1] and utilization of pentavalent-3 immunisation [AOR= 1.2, 95% CI 0.8 to 1.6], but negatively associated with care-seeking for childhood illnesses [AOR= 0.6, 95% CI 0.4 to 0.8].

Conclusion: We found no association between facility readiness and awareness or utilisation of child health services. There were significant deficiencies in health post preparedness for services. Caregivers had low awareness and utilisation of preventive and curative pneumonia-related services. The results underline the importance of enhancing facility preparedness, providing high-quality care, and intensifying demand generation efforts to prevent and treat pneumonia.

Keywords: Childhood pneumonia; Health post preparedness; Health care utilisation; Ethiopia

For peer review only - http://bmjopen.bmj.com/site/about/guidelines.xhtml

Strengths and limitations of this study

- This is the first study, which assessed the association between first-level healthcare facilities and caregiver's awareness and utilisation of pneumonia relevant preventive and curative services in four of the most populous Ethiopian regions.
- Health post preparedness was assessed using the standard World Health Organization Health Service Availability and Readiness Assessment tool.
- We ascertained information on the utilisation of pneumonia-related preventive and curative services with methods used by Demographic and Health Surveys, despite this assessment of care-seeking behaviour and pentavalent immunisation by caregivers' recall might be influenced by bias.
- The study was conducted in 52 districts of four Ethiopian regions and assessed facility readiness at the health post level. Hence, the findings may not be generalized to other geographic areas and health system levels.

INTRODUCTION

Worldwide, pneumonia is responsible for one-fifth of all under-five deaths [1]. Nearly half (49%) of these deaths occur in four low- and middle-income countries, including Ethiopia [2 3]. There are effective preventive measures, including immunisation and antibiotics that can prevent or treat most cases of pneumonia. Despite these resources, children in low-income countries continue to suffer and die from pneumonia due to lack of access to or availability of these services [4].

The World Health Organization and UNICEF introduced the integrated Community Case Management (iCCM) strategy in 2004 to increase access and quality of services for childhood pneumonia, diarrhea, and malaria. Effective implementation of this strategy requires uninterrupted stock of drugs and supplies, trained community health workers, and community awareness of these services [5 6]. Nevertheless, studies in low-income countries reveal that less than a fifth of sick children were brought to relevant health services for suspected pneumonia [7 8]. This low level of care-seeking has, among other things, been attributed to the poor quality of health services [9]. A majority of the facilities in these settings have reportedly not had essential drugs, supplies and trained community health workers [10-13]. The general service readiness index for sick child care has varied between 19% and 69% in reports from Sub-Saharan African countries [11 13]. The readiness has been lower in rural areas and at the lowest primary healthcare level [14-17]. In Ethiopia, earlier reports have shown a lack of iCCM drugs and supplies at health posts [9 18].

For peer review only - http://bmjopen.bmj.com/site/about/guidelines.xhtml

Inadequate preparedness and low service quality at the primary healthcare level reduce parents thrust and utilisation of health services [11 19-21]. In Ethiopia, low utilisation of iCCM services was also attributed to absent supervision and refresher training of health extension workers [22-27]. We have earlier shown that a substantial proportion of caregivers were not aware of pneumonia-related health services and, therefore, less likely to seek care when their children got sick or get their children immunised [28 29]. The low utilisation of iCCM services has also been attributed to the lack of readiness of health posts to care for sick children [19 30 31]. Thus, there are reasons to investigate the primary-level health facility preparedness to provide child health services and whether this is associated with the coverage of pneumonia-related preventive and treatment services. In Ethiopia, under-five mortality was reported to be 55/1000 live births in the 2019 Demographic and Health Survey. Although reduced in recent decades, the persistently high level and continued pneumonia deaths call for intensified efforts to reduce these preventable deaths [28 32]. The Ethiopian Ministry of Health in collaboration with non-governmental organisations implemented the complex community-based intervention labelled Optimizing the Health Extension Program (OHEP) intervention in four of the most populous regions. The OHEP aimed to contribute to reductions in under-five child mortality through enhancing utilisation of iCCM and community-based newborn care (CBNC) services [33]. This study was part of the evaluation of OHEP intervention. We aimed to examine the association between the health post readiness to serve and caregiver's awareness of pneumonia-related services, care seeking for sick children, and whether their 12-23 months old children had got three pentavalent vaccinations.

METHODS

Study setting and design

The Ethiopian primary healthcare system typically consists of a primary hospital, a health centre and five satellite health posts. A health post is the lowest service delivery point staffed by two health extension workers serving around 5,000 rural residents. Since 2003, Ethiopia has implemented the health extension program to achieve universal health coverage in rural areas. The integrated community case management (iCCM) of childhood illnesses is one of the components of this national programme. As part of this programme, the health extension workers examine, classify, and treat pneumonia. The OHEP encompassed three intervention strategies, i.e., community engagement activities, capacity building of health extension workers and women's development group leaders, and strengthening of the district health services' ownership and accountability of the primary newborn and child health services. The intervention was conducted in 26 intervention districts with 26 comparison districts spread in four regions

For peer review only - http://bmjopen.bmj.com/site/about/guidelines.xhtml

of Ethiopia, namely Tigray, Amhara, Oromia and Southern Nations, Nationalities and Peoples Regions. The intervention started in 2016 and was completed in 2018. For the evaluation, baseline and end line surveys were performed. This paper reports a secondary analysis of end line cross-sectional data [33].

Participants

This study included all caregivers and children aged 2 to 59 months, who resided in 52 study districts. It also includes all health posts with one or two health extension workers per health post serving these families.

Sampling

Sample size for the end line survey was estimated to measure changes in care-seeking and appropriate treatment for childhood illnesses following the implementation of the OHEP intervention. Assumptions considered for the sample size calculation for the main OHEP evaluation [34] were 80% power to detect differences of 15 and 20 percentage points, respectively, in the reported level of care seeking (55%) and appropriate treatment for childhood illnesses (47%) at the baseline, design effect of 1.001 and 90% completeness. Accordingly, a sample size of 12,000 households was obtained. With this number of households, 6,532 children below the age of five years were expected to be reached by the survey, of whom 368 were assumed to have any illnesses and 308 to have suspected pneumonia within two weeks before the survey.

The parent study used a sampling frame generated based on the 2007 Ethiopian Central Statistical Agency housing and population survey. Two hundred enumeration areas, i.e., clusters, were selected from 52 study districts with probability proportional to size. A two-staged cluster sampling followed by systematic sampling to select 60 representative households from each study cluster. All caregivers of children aged 2-59 months living in the selected households were interviewed. Moreover, all health posts and one health extension worker from each health post serving the population of the study clusters were included.

Data collection

Data were collected using structured and pre-tested interviewer-administered questionnaires through an electronic data collection software (CSpro survey software). The questionnaires were translated into local languages (Amharic, Tigrigna, and Oromiffa) and back-translated into English. They comprised of three main modules; household, healthcare provider and health facility modules. Data collectors and

For peer review only - http://bmjopen.bmj.com/site/about/guidelines.xhtml

For peer review only

supervisors, who had bachelor's degree or above, were trained for two weeks on data collection techniques, procedures, quality assurance, and ethical considerations of the study. Further detailed information about data collection and quality control is available in the published study protocol [33].

Outcomes

The outcomes of this study are caregiver's awareness of pneumonia treatment, care-seeking behaviour and coverage of pentavalent-3 immunisation as defined in the Demographic and Health Surveys [28]. The awareness of availability of pneumonia treatment was calculated as the proportion of caregivers who had heard messages regarding pneumonia treatment. Suspected pneumonia was ascertained by asking the caregiver if the child had cough combined with either fast or difficult breathing due to chest problems within two weeks before the survey. Care-seeking was defined as children with suspected pneumonia for whom advice or treatment was sought from an appropriate care provider, i.e., either government or private providers. The vaccination status of children aged 12-23 months was primarily assessed by reviewing immunisation cards. When cards were not available at home, the caregivers were requested to report the type of vaccines their children had received. Hence, coverage of pentavalent vaccination was estimated as the proportion of children 12-23 months who had received three doses of pentavalent vaccine.

Health post readiness

The readiness of health posts for sick child care was assessed using the World Health Organization Service Availability and Readiness Assessment tool [35]. Using 23 tracer items, the preparedness of facilities was shown in five domains or indices, i.e., basic amenities, basic equipment, standard precaution equipment for infection prevention, diagnostic capacity, and essential medicines. The mean availability of items across the four domains of readiness was estimated by assigning equal weight for each of the items, and was expressed as a proportion. Health post's diagnostic capacity was shown as the proportion of facilities having rapid diagnostic test for malaria. The general service readiness was calculated as the average of percentages depicting mean availability of tracer items in five indices [35]. The availability of vaccination at the health posts was also estimated. The number of health extension workers working in the health post and the percentage of these workers trained for iCCM and who had received supportive supervision during six months before the survey were also calculated. The health post demand generation activities were recorded as the proportion of health post showing opening days or that used community forums to deliver maternal and child health education.

For peer review only - http://bmjopen.bmj.com/site/about/guidelines.xhtml

Analyses

The household socio-economic status was constructed through principal component analysis of household assets, ownership of house, livestock, agricultural land, and access to utilities and infrastructures. The factor scores were summed and ranked into quintiles from the poorest to the least poor.

The study linked the household, health facility and care provider information. Our analysis was based on linked samples for outcome variables, i.e., caregivers' awareness of pneumonia treatment (n=4,934), care seeking when the child was sick (n=613) and vaccination of 12-23 months old children with third dose of pentavalent immunisation (n=860). Care-seeking was assessed for all childhood illness episodes, including symptoms of suspected pneumonia as reported by caregiver for the two weeks prior to the survey. The effect of clustering on three of the study outcomes was examined by estimating Intra Cluster Correlation Coefficient (ICC). A multilevel binary logistic regression model was fitted to examine the association between heath post readiness and household level awareness, care-seeking and utilisation of three doses of pentavalent vaccinations. We checked for potential household-level confounders. The fitness of the model was checked through Likelihood Ratio Test. Data were analysed using Stata version-14.

Patient and public involvement

Patients or the public were not involved in the design or conduct or reporting or dissemination plans of this research.

RESULTS

Characteristics of caregivers and children

A total of 10,785 households from 181 study clusters, 165 health posts and 274 health extension workers were included in the survey. A total of 4,729 eligible caregivers with 5,787 children aged 2-59 months were included in the analyses. A majority of the caregivers had no schooling (59.4%) and were currently married (88.6%). About two-thirds (64.0%) of caregivers were able to access the nearest health facility within 30 minutes of walk from their home (Table 1).

For peer review only - http://bmjopen.bmj.com/site/about/guidelines.xhtml

Table 1 Socio-demographic characteristics of caregivers and children aged 2-59 months in four regions of Ethiopia, December 2018 to February 2019

Characteristics	Frequency	Percentage
Caregiver's characteristics (n=4,729)		
Age		
<25	885	18.7
25-29	1,281	27.1
30-34	1,038	22.0
35-39	867	18.3
>=40	658	13.9
Marital status		
Currently married	4,067	88.6
Unmarried	521	11.4
Education		
No schooling	2,810	59.4
Schooling	1,919	40.6
Number of children under five		
1	3,487	73.7
2	1,148	24.3
3+	94	2.0
Socio-economic quintiles		
Q1 (poorest)	1,024	21.7
Q2	982	20.8
Q3	874	18.5
Q3	895	18.9
Q5 (least poor)	954	20.1
Walking distance from household to nearest health facility (n=3,918)		
<=30 minutes	2,507	64.0
>30 minute	1,411	36.0
Child (2-59 months) characteristics (n=5,787)		
Sex		
Male	2,961	51.2
Female	2,826	49.8
Age		
2-11 Months	959	16.6
12-23 Months	992	17.1
24-35 Months	1114	19.3
36-59 Months	2722	47.0

Characteristics of health posts and health extension workers

The median number of households served by the health post was 1,181. The majority (85.1%) of the health posts were functionally open for five days or more per week. More than a tenth (12.7%) of the health posts were served by only one health extension worker. Most (78.8%) of the health extension workers were trained in the integrated community Case Management of childhood illnesses. More than three-fourth (78.8%) had received supportive supervision within six months preceding the survey. Three-

For peer review only - http://bmjopen.bmj.com/site/about/guidelines.xhtml

quarters used community forums or meetings to deliver maternal and child health education within three months prior to the survey (Table 2).

Table 2 Characteristics of health post in four regions of Ethiopia, December 2018 to February 2019

Characteristics	Frequency	Percentage
Health posts catchment area population (n=165)		
Number of households, median (Inter-quartile range)	1,181 (841, 1572)	
Number of children under five years, median (Inter-quartile range)	780 (493, 1065)	
Health extension workers' characteristics (274)		
Number of health extension workers per health post		
One	35	12.7
Two	142	51.8
Three and above	97	35.4
Health post opening days per week		
1 to 4 days	41	14.9
5 to 7 days	233	85.1
Posted health post opening days	54	19.7
Trained for integrated community case management of childhood illnesses	216	78.8
Received supportive supervision in the last six months	216	78.8
Participated in Performance Review and Clinical Mentorship meetings	126	46.0
Used community forums to deliver maternal and child health education	205	74.8

Health post preparedness to deliver sick child care services

The general service readiness of health posts (n=165) to deliver sick child health services was estimated at 62.8%. Half of the health posts had Rapid Diagnostic Test for malaria, and the mean availability of essential medicine was 66.9%. Relatively improved mean availability (80.1%) of tracer items was shown for basic equipment, while the lowest (48.7%) was for availability of standard precaution items. Very few health posts had all essential medicines and standard precaution equipment. Most of the health posts (84.2%) had vaccination cards (Figure 1).

Figure 1 Percentage of health posts (n=165) with drugs and supplies to deliver pneumonia-related and other sick child care services in four regions of Ethiopia, December 2018 to February 2019

Awareness of treatment, actual care-seeking and utilisation of preventive immunisation

During the two weeks before the survey 704 (12.3%) of the children had any illnesses. Of these, 44 children had symptoms and signs of suspected pneumonia. Only one in five (23.9%) caregivers were aware of the

For peer review only - http://bmjopen.bmj.com/site/about/guidelines.xhtml

availability of pneumonia treatment services. Health care was sought for one-third (36.1%) of children with any illnesses and for 56.8% of children with suspected pneumonia. Almost half (49.3%) of children aged 12-23 months had received three doses of pentavalent vaccinations (Table 3).

Table 3 Childhood illness and care utilisation by child demographic characteristics in four regions of Ethiopia, December 2018 to February 2019

Service utilisation	Frequency	Percentage
Children with any illnesses		
Yes	704	12.3
No	5,021	87.7
Child illnesses by sex (n=704)		
Male	362	51.4
Female	342	48.6
Childhood illnesses by age (n=704)		
2-11 Months	121	17.2
12-23 Months	147	20.9
24-35 Months	152	21.6
36-59 Months	284	40.3
Child with suspected pneumonia		
Yes	44	0.8
No	5,743	99.2
Child with suspected pneumonia treated with antibiotics (n=44)		
Yes	17	38.6
No	27	61.4
Full pentavalent vaccination by sex (n=489)		
Male	258	52.8
Female	231	47.2

Association of health post readiness with awareness and utilisation of pneumonia care

The Intra Cluster Correlation Coefficient (ICC) indicated that the study outcomes, i.e., awareness of pneumonia treatment (ICC=0.29), care-seeking behaviour (ICC=0.16) and pentavalent vaccination (ICC=0.28) significantly varied with level of clusters. While checking for the household-level confounders, we found that none of the household, caregiver and child characteristics were associated with the outcomes and main exposure variables (P-value of<0.2), i.e., the five health post readiness indices. But regardless of the statistical screening criteria (P-value of<0.2), analyses were adjusted for maternal age, education and intervention-comparison variables.

For peer review only - http://bmjopen.bmj.com/site/about/guidelines.xhtml

Analyses tested the association of general health post readiness index with study outcomes were adjusted for all independent variables (shown in Table 5) in addition to mother's age, education and intervention-comparison variables. The adjusted multilevel analyses revealed that general health post readiness was not associated with mother's awareness of availability of pneumonia treatment [AOR= 0.9, 95% CI 0.7 to 1.1] and utilisation of pentavalent-3 immunisation [AOR= 1.2, 95% CI 0.8 to 1.6]. The general health post readiness was negatively associated with care-seeking for childhood illnesses [AOR= 0.6, 95% CI 0.4 to 0.8], Table 4.

Table 4 General health post readiness and caregiver's awareness, care-seeking and utilisation of pentavalent-3 immunisation in four regions of Ethiopia, December 2018 to February 2019

**Awareness and utilisation**	**General readiness**	
	**Crude Odds Ratio (95% CI)**	**Adjusted Odds Ratio (95% CI)**
Awareness of treatment service (N= 4,934)	0.9 (0.7,1.1)	0.9 (0.7,1.1)
Care seeking (N=613)	0.7 (0.5,0.9)	0.6 (0.4,0.8)
Pentavalent-3 (N=860)	1.2 (0.9,1.6)	1.2 (0.8,1.6)

As illustrated in Table-5, none of the health post readiness indices were associated with mother's awareness of availability of pneumonia treatment and care seeking for childhood illnesses. Only availability of standard precaution equipment for infection prevention was positively associated with utilisation of pentavalent-3 immunisation [AOR= 4.5, 95% CI 1.6 to 12.8]. On the other hand, our analysis revealed that home visiting by the health extension workers was associated with higher odds of [AOR= 2.9, 95% CI 2.3 to 3.6] mother's awareness of availability of pneumonia treatment.

For peer review only - http://bmjopen.bmj.com/site/about/guidelines.xhtml

Table 5 Health post preparedness indices associated with caregiver's awareness, care-seeking and utilisation of three doses of pentavalent vaccines in four regions of Ethiopia, December 2018 to February 2019

Characteristics	Awareness of treatment service (N= 4,934)				Care seeking (N=613)				Pentavalent-3 (N=860)			
	Aware	Unaware	Crude Odds Ratio (95% CI)	Adjusted Odds Ratio (95% CI)	Sought care	Didn't seek care	Crude Odds Ratio (95% CI)	Adjusted Odds Ratio (95% CI)	Vaccinated	Not vaccinated	Crude Odds Ratio (95% CI)	Adjusted Odds Ratio (95% CI)
Basic amenities												
All not available	923	2,867	1.0	1.0	161	270	1.0	1.0	325	337	1.0	1.0
All available	256	888	0.9 (0.6,1.5)	0.9 (0.6,1.5)	49	133	0.7 (0.4,1.1)	0.7 (0.4,1.2)	98	100	0.9 (0.6,1.8)	0.9 (0.6,1.8)
Basic equipment												
All not available	590	2,021	1.0	1.0	117	202	1.0	1.0	219	224	1.0	1.0
All available	589	1,734	1.3 (0.9,1.9)	1.2 (0.8,1.9)	93	201	0.8 (0.5,1.3)	0.9 (0.5,1.4)	204	213	0.9 (0.6,1.6)	0.8 (0.5,1.4)
Standard precaution equipment for infection prevention												
All not available	1,091	3,555	1.0	1.0	202	379	1.0	1.0	386	414	1.0	**1.0**
All available	88	200	1.9 (0.9,3.9)	1.9 (0.9,4.2)	8	24	0.6 (0.2,1.7)	0.8 (0.3,2.1)	37	23	2.3 (0.9,5.6)	**4.5 (1.6,12.8)**
Rapid diagnostic test for malaria												
No available	597	1,858	1.0	1.0	NA	NA	NA	NA	NA	NA	NA	NA
Available	582	1,897	0.8 (0.5,1.2)	0.7 (0.4,1.1)	NA	NA	NA	NA	NA	NA	NA	NA
Essential medicine												
First tercile	354	1025	1.0	1.0	58	98	1	1	108	126	1.0	1.0
Second tercile	396	1355	0.9 (0.5,1.4)	0.9 (0.5,1.4)	76	142	0.8 (0.4,1.4)	0.8 (0.4,1.5)	129	145	0.9 (0.5,1.8)	0.8 (0.4,1.6)
Third tercile	429	1375	0.8 (0.5,1.3)	0.8 (0.5,1.4)	76	163	0.7 (0.4,1.3)	0.7 (0.4,1.4)	186	166	1.3 (0.7,2.3)	1.1 (0.6,2.1)
Number of health extension workers per health post												
One	226	784	1.0	1.0	47	71	1.0	1.0	73	68	1.0	1.0
Two and above	953	2,971	1.2 (0.7,1.9)	1.2 (0.7,1.9)	163	332	0.8 (0.4,1.4)	0.8 (0.5,1.5)	350	369	0.9 (0.5,1.6)	0.7 (0.4,1.4)
Number of health post opening days												
Less than five days	209	582	1.0	1.0	43	71	1.0	1.0	61	67	1.0	1.0

For peer review only - http://bmjopen.bmj.com/site/about/guidelines.xhtml

Five days and above	970	3,173	0.8 (0.5,1.3)	0.7 (0.4,1.2)	167	332	0.9 (0.5,1.5)	0.7 (0.4,1.4)	362	370	1.3 (0.7,2.5)	1.4 (0.7,2.8)
Health extension workers used community forum												
No	297	1,003	1.0	1.0	70	139	1.0	1.0	113	131	1.0	1.0
Yes	882	2,752	1.2 (0.8,1.8)	1.3 (0.8,1.9)	140	264	1.0 (0.6,1.6)	0.9 (0.6,1.5)	310	306	1.3 (0.8,2.2)	1.3 (0.7,2.2)
Health extension workers received supervision												
No	NA	NA	NA	NA	44	105	1.0	1.0	60	68	1.0	1.0
Yes	NA	NA	NA	NA	166	298	1.3 (0.8,2.3)	1.5 (0.8,2.6)	363	369	1.4 (0.7,2.5)	1.1 (0.5,2.2)
Health extension workers received iCCM training												
Did not received training	NA	NA	NA	NA	40	74	1.0	1.0	NA	NA	NA	NA
Received training	NA	NA	NA	NA	170	329	0.9 (0.5,1.6)	0.9 (0.5,1.7)	NA	NA	NA	NA
Health extension workers participated at Performance Review and Clinical Mentorship meeting												
No	NA	NA	NA	NA	123	252	1.0	1.0	232	252	1.0	1.0
Yes	NA	NA	NA	NA	87	151	1.2 (0.8,1.9)	1.2 (0.7,2.0)	191	185	1.2 (0.7,1.9)	0.9 (0.5,1.5)
Home vising												
No	934	3,445	1	**1**	175	357	1	1	372	386	1.0	1.0
Yes	244	309	2.9 (2.4,3.7)	**2.9 (2.3,3.6)**	35	46	1.6 (0.9,2.6)	1.5 (0.9,2.5)	51	38	1.6 (0.9,2.7)	1.6 (0.9,2.7)

For peer review only - http://bmjopen.bmj.com/site/about/guidelines.xhtml

DISCUSSION

Overall, this study showed insufficient health post service preparedness and low household awareness and utilisation of preventive and curative services for childhood suspected pneumonia. There was no consistent association between facility preparedness and utilisation of preventive and curative services.

Strengths and limitations

With an attempt of narrowing the existing scarcity of evidence, our study examined the association between health post readiness and household level awareness and utilisation of pneumonia relevant health services in a wider geographic area. Readiness of health posts was measured using the standard WHO Health Service Availability and Readiness Assessment tool [35]. When vaccination cards were not available at home, children's pentavalent immunisation status was assessed through interviewing the caregivers. Likewise, ascertainment of childhood suspected pneumonia and other illnesses was based on the caregiver's two weeks reported symptoms prior to the survey. These ascertainment techniques have been used by the Demographic and Health Surveys, but might be influenced by recall bias [28]. Furthermore, we assessed health post service readiness and pneumonia service utilisation in selected, but representative, districts of four Ethiopian regions, therefore the findings may not be generalized to other geographic areas and health system levels.

Health post preparedness

This study showed that two-thirds of the health posts were ready to provide sick child care, suggesting that the remaining significant proportion of facilities were not able to deliver such services. More or less similar level of structural preparedness of health posts or primary health care facilities for pneumonia and other sick child care were previously reported in Ethiopia and other low- and middle-income countries [10 36 37]. Furthermore, health posts or health centres of Ethiopia and other low-income countries were found with low readiness to provide quality care to sick children [9]. According to the Ethiopia health system, a health centre is structured to support and strengthen five health posts with their catchment areas, hence insufficient preparedness of the surveyed health posts could be explained by scarcity of supplies at health centres or poor linkages between health posts and health centres or the health system at large [29 38].

Inadequate readiness of health posts in the study setting and other low-income countries indicates a serious challenge to community case management of pneumonia, particularly in the rural or unreached communities, where a majority of preventable deaths occur [6 28]. Most importantly, only a few of the

For peer review only - http://bmjopen.bmj.com/site/about/guidelines.xhtml

surveyed health posts had all essential medicines and just half had diagnostic equipment, clearly indicating their limitations in providing effective pneumonia or sick child treatment [5 6]. Scarcity of essential medicines in Ethiopia and other Sub-Saharan African countries results in missed pneumonia treatments at facility level [13 17 29]. Unavailability of rapid diagnostic tests impairs community health worker's ability to differentiate suspected pneumonia from malaria in case of symptom overlap, a common clinical problem in African children [39-41].

Awareness and utilisation of pneumonia-related health services

Our study revealed low health care utilisation for pneumonia-specific preventive and curative services. These levels of service utilisation were lower compared to the reported regional pentavalent-3 immunisation coverage (80%) and care seeking behaviour (85%) for childhood suspected pneumonia and other illnesses in Sub-Saharan African Countries [42 43]. Community awareness of illness and sick child care is a pre-requisite to seek timely health services [44]. We found that less than a fifth of caregivers were aware of the availability of pneumonia treatment services, and this might partly explain the observed poor utilisation of pneumonia-related health services in the study setting [45 46]. The reported low care utilisation could also be explained by inadequate readiness or service quality of the primary health care facilities for pneumonia-related preventive and treatment services [36 47]. The OHEP evaluation studies have revealed a low quality of sick child care services provided at the primary health care facilities, and caregivers of children have also mentioned this as a key barrier to seek care at health posts [48-50]. A study in 22 African countries noted a low level of community trust in public health facilities as an important reason to the low coverage of child vaccination [51].

Association between health post preparedness and utilisation of preventive immunisation and care seeking behaviour

Earlier studies have shown a positive association between health facility readiness and utilisation of first-level sick child care [11 21]. We did not find any consistent pattern of relationship between facility readiness indices and utilisation of services. There was a positive association between the availability of standard precaution equipment for infection prevention and utilisation of pentavalent-3 immunisation. However, the general health post readiness had no association with awareness and coverage of pentavalent-3 immunisation, but a negative association with care-seeking for childhood suspected pneumonia and other illnesses. Another national study also showed absence of association between readiness of primary health care facilities for sick child care and caregivers' satisfaction to the respective services [52]. The lack of

For peer review only - http://bmjopen.bmj.com/site/about/guidelines.xhtml

consistent positive association between service readiness and use of services may be linked to the dominating low level of facility preparedness. In spite of this, equipping health care facilities with relevant equipment is a prerequisite to enhance the quality of iCCM services [6]. Hence, the reported lack of association of health post readiness with awareness and utilisation of pneumonia treatment services suggests that simply equipping facilities with necessary supplies is not a guarantee to reach the intended level of community awareness and utilisation of health services. Health facility strengthening efforts, therefore, should go along with implementation of awareness creation and demand generation interventions to increase the community awareness, trust and utilisation of pneumonia-related and other child health services [44]. In line with this notion, our study revealed that home visiting by the health extension workers was positively associated with caregivers' awareness of pneumonia treatment. Home visiting by the community health workers is one of the vital strategies to promote child health and enhance awareness and utilisation of health services [44 53]. A sub-study of the same project also showed that awareness creation and delivery of preventive child health interventions (such as immunisations) were the main components of outreach services delivered by the health extension workers [48].

Relation of findings with already published OHEP evaluation studies

This study was part of the end line evaluation of the OHEP intervention. The findings showed sizable gaps in structural readiness of health posts for sick child care. Results of our published baseline study also illustrated the scarcity of essential drugs and other supplies at the health posts [29]. Sub-studies of the same project investigated process aspects of quality of sick child care, and results showed low clinical performance of the health extension workers to identify and treat childhood suspected pneumonia and other illnesses [49 50]. A qualitative study noted a lack of caregiver's trust in the health extension workers' clinical competency to manage sick children and a low availability of essential drugs, diagnostic and other supplies at health posts. The low quality of sick child care at health posts was a barrier to utilise the services [48].

CONCLUSIONS

This study has shown a low health post readiness for services, and low household awareness and utilisation of pneumonia-relevant preventive and curative services. Parent's awareness and utilisation of pneumonia-specific preventive and curative services were not consistently associated with the health post readiness. These findings underline the pivotal role of strengthening the availability and quality of pneumonia and sick child care services at the health posts. There is a need to simultaneously equip

For peer review only - http://bmjopen.bmj.com/site/about/guidelines.xhtml

facilities and intensify the community awareness creation and demand generation efforts to improve utilisation of essential child health services.

References

1. Liu L, Johnson HL, Cousens S, et al. Global, regional, and national causes of child mortality: an updated systematic analysis for 2010 with time trends since 2000. The Lancet 2012;**379**(9832):2151-61.
2. United Nations Inter-agency Group for Child Mortality Estimation (UN IGME), 'Levels & Trends in Child Mortality: Report 2020, Estimates developed by the United Nations Inter-agency Group for Child Mortality Estimation', United Nations Children's Fund, New York, 2020.
3. McAllister DA, Liu L, Shi T, et al. Global, regional, and national estimates of pneumonia morbidity and mortality in children younger than 5 years between 2000 and 2015: a systematic analysis. The Lancet Global Health 2019;**7**(1):e47-e57.
4. Bhutta ZA, Das JK, Walker N, et al. Interventions to address deaths from childhood pneumonia and diarrhoea equitably: what works and at what cost? The Lancet 2013;**381**(9875):1417-29.
5. World Health Organization. *Management of pneumonia in community settings*: World Health Organization, Department of Child and Adolescent Health and …, 2004.
6. World Health Organization. WHO/UNICEF joint statement integrated community case management (iCCM): an equity-focused strategy to improve access to essential treatment services for children. Geneva and New York 2012.
7. Geldsetzer P, Williams TC, Kirolos A, et al. The recognition of and care seeking behaviour for childhood illness in developing countries: a systematic review. PloS one 2014;**9**(4):e93427.
8. Adedokun ST, Yaya S. Factors influencing mothers' health care seeking behaviour for their children: evidence from 31 countries in sub-Saharan Africa. BMC Health Services Research 2020;**20:842**.
9. Macarayan EK, Gage AD, Doubova SV, et al. Assessment of quality of primary care with facility surveys: a descriptive analysis in ten low-income and middle-income countries. The Lancet Global Health 2018;**6**(11):e1176-e85.
10. Acharya K B, J, and Tuladhar S,. Client Satisfaction and Quality of Curative Services for Sick Children in Nepal. DHS Further Analysis Reports No. 114. Rockville, Maryland, USA: ICF. 2018.
11. Liu L, Leslie HH, Joshua M, et al. Exploring the association between sick child healthcare utilisation and health facility quality in Malawi: a cross-sectional study. BMJ open 2019;**9**(7):e029631.
12. Shawon MSR, Adhikary G, Ali MW, et al. General service and child immunization-specific readiness assessment of healthcare facilities in two selected divisions in Bangladesh. BMC health services research 2018;**18**(1):39.
13. Taylor C, Linn A, Wang W, et al. Examination of malaria service utilization and service provision: an analysis of DHS and SPA data from Malawi, Senegal, and Tanzania. Malaria journal 2019;**18**(1):258.
14. Blanas DA, Ndiaye Y, Nichols K, et al. Barriers to community case management of malaria in Saraya, Senegal: training, and supply-chains. Malaria journal 2013;**12**(1):95.
15. Masters SH, Burstein R, DeCenso B, et al. Pharmaceutical availability across levels of care: evidence from facility surveys in Ghana, Kenya, and Uganda. PloS one 2014;**9**(12):e114762.
16. Ssempiira J, Kasirye I, Kissa J, et al. Measuring health facility readiness and its efects on severe malaria outcomes in Uganda. Scientific Reports 2018.
17. Bagonza J, Rutebemberwa E, Eckmanns T, et al. What influences availability of medicines for the community management of childhood illnesses in central Uganda? Implications for scaling up the integrated community case management programme. BMC Public Health 2015;**15**(1):1-8.
18. International EPHIEFMoHI. Ethiopia Service Provision Assessment Plus Survey. 2014.

For peer review only - http://bmjopen.bmj.com/site/about/guidelines.xhtml

19. Shaw B, Amouzou A, Miller NP, et al. Determinants of utilization of health extension workers in the context of scale-up of integrated community case management of childhood illnesses in Ethiopia. The American journal of tropical medicine and hygiene 2015;**93**(3):636-47.
20. Anselmi L, Lagarde M, Hanson K. Health service availability and health seeking behaviour in resource poor settings: evidence from Mozambique. Health Economics Review (2015) 5:26
21. Kuwawenaruwa A, Wyss K, Wiedenmayer K, et al. The effects of medicines availability and stock-outs on household's utilization of healthcare services in Dodoma region, Tanzania. Health policy and planning 2020;**35**(3):323-33.
22. Kok MC, Kea AZ, Datiko DG, et al. A qualitative assessment of health extension workers' relationships with the community and health sector in Ethiopia: opportunities for enhancing maternal health performance. Human resources for health 2015;**13**(1):80.
23. Befekadu A, Yitayal M. Knowledge and practice of health extension workers on drug provision for childhood illness in west Gojjam, Amhara, Northwest Ethiopia. BMC Public Health 2020;**20**:1-10.
24. Miller NP, Amouzou A, Tafesse M, et al. Integrated community case management of childhood illness in Ethiopia: implementation strength and quality of care. The American journal of tropical medicine and hygiene 2014;**91**(2):424-34.
25. Tilahun H, Fekadu B, Abdisa H, et al. Ethiopia's health extension workers use of work time on duty: time and motion study. Health policy and planning 2017;**32**(3):320-28.
26. Datiko DG, Bunte EM, Birrie GB, et al. Community participation and maternal health service utilization: lessons from the health extension programme in rural southern Ethiopia. Journal of Global Health Reports 2019;**3**.
27. Mangham-Jefferies L, Mathewos B, Russell J, et al. How do health extension workers in Ethiopia allocate their time? Human resources for health 2014;**12**(1):61.
28. Central Statistical Agency [Ethiopia] and ICF International. Ethiopia Demographic and Health Survey 2016. Addis Ababa, Ethiopia and Calverton, Maryland, USA: Central Statistical Agency and ICF International. 2017.
29. Tariku A, Okwaraji YB, Worku A, et al. Prevention and treatment of suspected pneumonia in Ethiopian under-five children from household to primary care. Acta Paediatrica 2020.
30. Berhanu A, Alemayehu M, Kassa Daka WB, et al. Utilization of Integrated Community Case Management of Childhood Illnesses at Health Posts in Southern Ethiopia. Pediatric Health, Medicine and Therapeutics 2020;**11**:459.
31. Ethiopian Public Health Institute: World Health Organization. Ethiopia Service Availability and Readiness Assessment 2016 Summary Report. 2016.
32. Deribew A, Tessema GA, Deribe K, et al. Trends, causes, and risk factors of mortality among children under 5 in Ethiopia, 1990–2013: findings from the Global Burden of Disease Study 2013. Population health metrics 2016;**14**(1):42.
33. Berhanu D, Okwaraji YB, Belayneh AB, et al. Protocol for the evaluation of a complex intervention aiming at increased utilisation of primary child health services in Ethiopia: a before and after study in intervention and comparison areas. BMC Health Services Research 2020;**20**:1-12.
34. Berhanu D, Okwaraji YB, Defar A, et al. Does a complex intervention targeting communities, health facilities and district health managers increase the utilisation of community-based child health services? A before and after study in intervention and comparison areas of Ethiopia. BMJ open 2020;**10**(9):e040868.
35. Organization WH. Service availability and readiness assessment (SARA): an annual monitoring system for service delivery: reference manual: World Health Organization, 2013.
36. Hailemariam S, Gebeyehu Y, Loha E, et al. Inadequate management of pneumonia among children in South Ethiopia: findings from descriptive study. BMC health services research 2019;**19**(1):426.

For peer review only - http://bmjopen.bmj.com/site/about/guidelines.xhtml

37. Gage AD, Leslie HH, Bitton A, et al. Does quality influence utilization of primary health care? Evidence from Haiti. Globalization and health 2018;**14**(1):1-9.
38. Sousa Ad, Tiedje KE, Recht J, et al. Community case management of childhood illnesses: policy and implementation in Countdown to 2015 countries. Bull World Health Organ 2012;**90:183–190**.
39. Ukwaja KN, Aina OB, Talabi AA. Clinical overlap between malaria and pneumonia: can malaria rapid diagnostic test play a role? The Journal of Infection in Developing Countries 2011;**5**(03):199-203.
40. Kyabayinze DJ, Asiimwe C, Nakanjako D, et al. Use of RDTs to improve malaria diagnosis and fever case management at primary health care facilities in Uganda. Malaria journal 2010;**9**(1):1-10.
41. Yeboah-Antwi K, Pilingana P, Macleod WB, et al. Community case management of fever due to malaria and pneumonia in children under five in Zambia: a cluster randomized controlled trial. PLoS Med 2010;**7**(9):e1000340.
42. Ahinkorah BO, Budu E, Seidu A-A, et al. Barriers to healthcare access and healthcare seeking for childhood illnesses among childbearing women in sub-Saharan Africa: A multilevel modelling of Demographic and Health Surveys. PLoS ONE 16(2): e0244395 2021.
43. Fenta SM, Biresaw HB, Fentaw KD, et al. Determinants of full childhood immunization among children aged 12– 23 months in sub-Saharan Africa: a multilevel analysis using Demographic and Health Survey Data. Tropical Medicine and Health (2021) 49:29.
44. Miller NP, Bagheri Ardestani F, Wong H, et al. Barriers to the utilization of community-based child and newborn health services in Ethiopia: a scoping review. Health Policy and Planning 2021.
45. Sadruddin S, Khan IuH, Bari A, et al. Effect of community mobilization on appropriate care seeking for pneumonia in Haripur, Pakistan. Journal of Global Health 2015;**5 (1)**.
46. Noordam AC, Sharkey AB, Hinssen P, et al. Association between caregivers' knowledge and care seeking behaviour for children with symptoms of pneumonia in six subSaharan African Countries. BMC Health Services Research (2017) 17:107
47. Tadesse T, Gelaw B, Haile Y, et al. Immunization service availability and readiness in primary health care in pastoral and semi-pastoral CGPP Ethiopia implementation districts. Ethiopian Journal of Health Development 2019;**33**.
48. Defar A, Alemu K, Tigabu Z, et al. Caregivers' and Health Extension Workers' Perceptions and Experiences of Outreach Management of Childhood Illnesses in Ethiopia: A Qualitative Study. International journal of environmental research and public health 2021;**18**(7):3816.
49. Daka DW, Wordofa MA, Woldie M, et al. Quality of clinical assessment and management of sick children by Health Extension Workers in four regions of Ethiopia: A cross-sectional survey. PLoS ONE 2020;**15(9): e0239361**.
50. Getachew T, Mekonnen S, Yitayal M, et al. Health Extension Workers' diagnostic accurac y for common childhood illnesses in four regions of Ethiopia: a cross-sectional study. Acta Pædiatrica 2019.
51. Stoop N, Hirvonen K, Maystadt J-F. Institutional mistrust and child vaccination coverage in Africa. BMJ Global Health 2021;6:e004595 doi:101136/bmjgh-2020-004595.
52. Getachew T, Abebe SM, Yitayal M, et al. Assessing the quality of care in sick child services at health facilities in Ethiopia. BMC Health Services Research 2020;**20**(1):1-12.
53. Tripathi A, Kabra S, Sachdev H, et al. Home visits by community health workers to improve identification of serious illness and care seeking in newborns and young infants from low-and middle-income countries. Journal of Perinatology 2016;**36**(1):S74-S82.

For peer review only

For peer review only - http://bmjopen.bmj.com/site/about/guidelines.xhtml

Acknowledgements

We would like to forward our deepest gratitude to the study participants. Our special thanks to field assistants involved in the data collection process.

Contributors

AT, LÅP, YB, YO, AW and GAB contributed to the conceptualization of the study. AT analysed and interpreted the data and drafted the manuscript. LÅP, YB, YO, AW and GAB contributed to analysis and writing of the paper. All authors have read and approved the final manuscript.

Funding

The study was funded by a grant from the Bill and Melinda Gates Foundation (grant INV-009691) to the London School of Hygiene & Tropical Medicine. The funder had no role in data collection, analysis, or interpretation of results.

Competing interests

The authors declare that they have no conflict of interest.

Patient consent for publication

Not applicable

Ethics approval

The original study was approved by the Ethical Review Boards of the Ethiopian Public Health Institute (protocol number SERO-012-8-2016), the London School of Hygiene & Tropical Medicine (protocol number 11235), and the University of Gondar (V/P/RCS/05/559/2019). A written informed consent was obtained from each household respondent, caregiver of the index child and the health workers.

Provenance and peer review

Not commissioned; externally peer reviewed.

Data availability statement

For peer review only - http://bmjopen.bmj.com/site/about/guidelines.xhtml

For peer review only

The data for this manuscript were primarily collected by the Ethiopian Public Health Institute and London School of Hygiene & Tropical Medicine. Interested researchers may contact the focal person, Dr Yemisrach B. Okwaraji through email: Yemisrach.Okwaraji@lshtm.ac.uk. All requests will be reviewed by this committee and if granted, data will be shared without any identifiers.

Figure legend

Figure 1 Percentage of health posts (n=165) with drugs and supplies to deliver pneumonia-related and other sick child care services in four regions of Ethiopia, December 2018 to February 2019

For peer review only

For peer review only - http://bmjopen.bmj.com/site/about/guidelines.xhtml

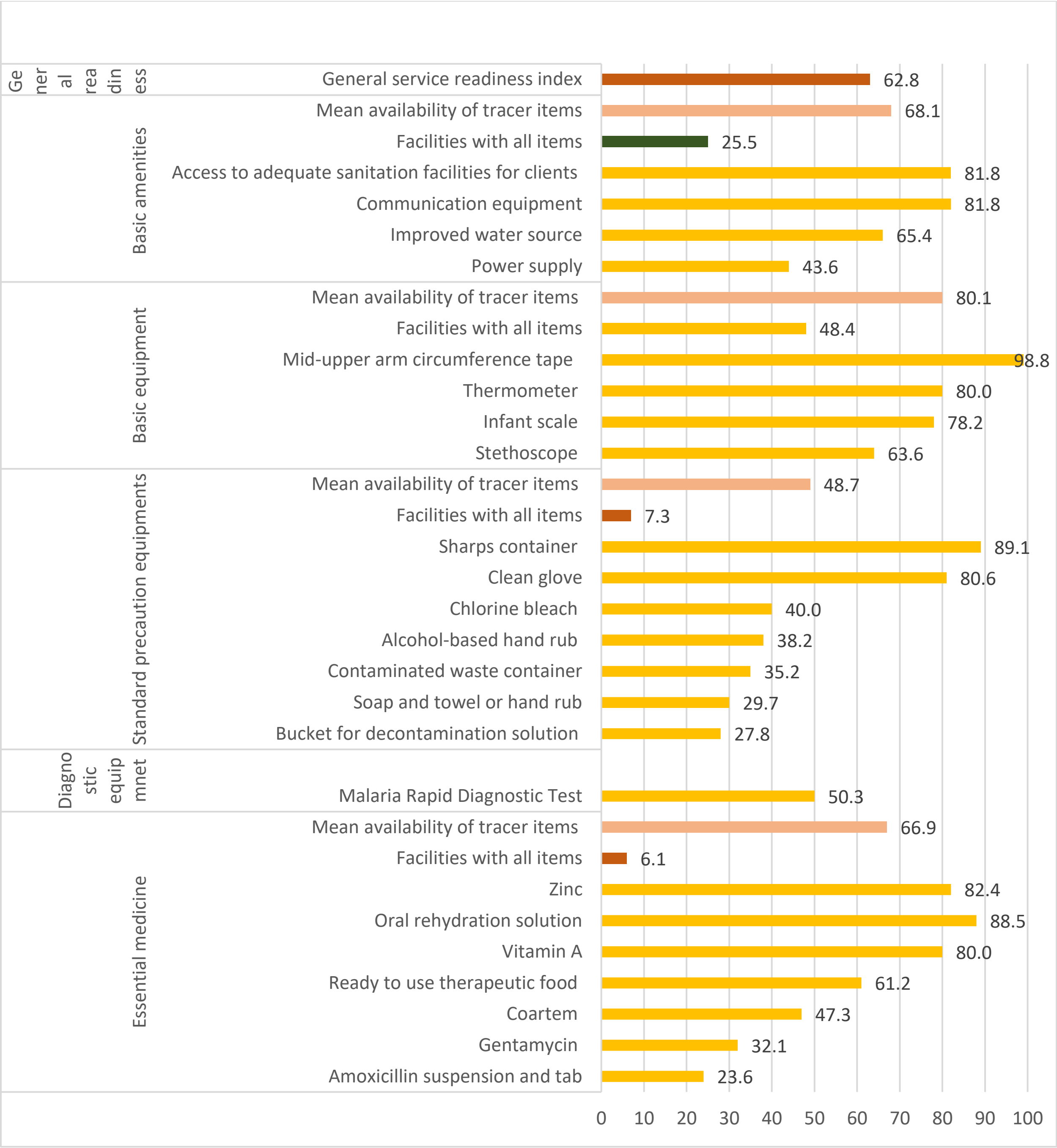

Figure 1 Percentage of health posts (n=165) with drugs and supplies to deliver pneumonia-related and other sick child care services in four regions of Ethiopia, December 2018 to February 2019

STROBE 2007 (v4) Statement—Checklist of items that should be included in reports of *cross-sectional studies*

Section/Topic	Item #	Recommendation	Reported on page #
**Title and abstract**	1	(*a*) Indicate the study's design with a commonly used term in the title or the abstract	1
		(*b*) Provide in the abstract an informative and balanced summary of what was done and what was found	2
**Introduction**			
Background/rationale	2	Explain the scientific background and rationale for the investigation being reported	3-4
Objectives	3	State specific objectives, including any prespecified hypotheses	4
**Methods**			
Study design	4	Present key elements of study design early in the paper	5
Setting	5	Describe the setting, locations, and relevant dates, including periods of recruitment, exposure, follow-up, and data collection	4-5
Participants	6	(*a*) Give the eligibility criteria, and the sources and methods of selection of participants	5
Variables	7	Clearly define all outcomes, exposures, predictors, potential confounders, and effect modifiers. Give diagnostic criteria, if applicable	6-7
Data sources/ measurement	8*	For each variable of interest, give sources of data and details of methods of assessment (measurement). Describe comparability of assessment methods if there is more than one group	6-7
Bias	9	Describe any efforts to address potential sources of bias	6-7
Study size	10	Explain how the study size was arrived at	5
Quantitative variables	11	Explain how quantitative variables were handled in the analyses. If applicable, describe which groupings were chosen and why	6–7
Statistical methods	12	(*a*) Describe all statistical methods, including those used to control for confounding	7
		(*b*) Describe any methods used to examine subgroups and interactions	7
		(*c*) Explain how missing data were addressed	7
		(*d*) If applicable, describe analytical methods taking account of sampling strategy	7
		(*e*) Describe any sensitivity analyses	-
**Results**			

For peer review only

Participants	13*	(a) Report numbers of individuals at each stage of study—eg numbers potentially eligible, examined for eligibility, confirmed eligible, included in the study, completing follow-up, and analysed	8
		(b) Give reasons for non-participation at each stage	-
		(c) Consider use of a flow diagram	-
Descriptive data	14*	(a) Give characteristics of study participants (eg demographic, clinical, social) and information on exposures and potential confounders	8-9
		(b) Indicate number of participants with missing data for each variable of interest	8, 11, 13, 14
Outcome data	15*	Report numbers of outcome events or summary measures	11
Main results	16	(*a*) Give unadjusted estimates and, if applicable, confounder-adjusted estimates and their precision (eg, 95% confidence interval). Make clear which confounders were adjusted for and why they were included	13-14
		(*b*) Report category boundaries when continuous variables were categorized	9
		(*c*) If relevant, consider translating estimates of relative risk into absolute risk for a meaningful time period	N/A
Other analyses	17	Report other analyses done—eg analyses of subgroups and interactions, and sensitivity analyses	12
**Discussion**			
Key results	18	Summarise key results with reference to study objectives	15
Limitations	19	Discuss limitations of the study, taking into account sources of potential bias or imprecision. Discuss both direction and magnitude of any potential bias	15
Interpretation	20	Give a cautious overall interpretation of results considering objectives, limitations, multiplicity of analyses, results from similar studies, and other relevant evidence	15 - 17
Generalisability	21	Discuss the generalisability (external validity) of the study results	15
**Other information**			
Funding	22	Give the source of funding and the role of the funders for the present study and, if applicable, for the original study on which the present article is based	18

*Give information separately for cases and controls in case-control studies and, if applicable, for exposed and unexposed groups in cohort and cross-sectional studies.

Note: An Explanation and Elaboration article discusses each checklist item and gives methodological background and published examples of transparent reporting. The STROBE checklist is best used in conjunction with this article (freely available on the Web sites of PLoS Medicine at http://www.plosmedicine.org/, Annals of Internal Medicine at http://www.annals.org/, and Epidemiology at http://www.epidem.com/). Information on the STROBE Initiative is available at www.strobe-statement.org.

For peer review only

Health post service readiness and use of preventive and curative services for suspected childhood pneumonia in Ethiopia: a cross-sectional study

Journal:	*BMJ Open*
Manuscript ID	bmjopen-2021-058055.R1
Article Type:	Original research
Date Submitted by the Author:	10-Mar-2022
Complete List of Authors:	Tariku, Amare; University of Gondar, Human Nutrition;<br>Berhane, Yemane; Addis Continental Institute of Public Health,<br>Worku, Alemayehu; Addis Ababa University, School of Public Health<br>Biks, Gashaw ; University of Gondar, Public Health ; University of Gondar College of Medicine and Health Sciences,<br>Persson, Lars; London School of Hygiene and Tropical Medicine,<br>Okwaraji, Yemisrach; London School of Hygiene & Tropical Medicine, Epidemiology and Population Health; 74B Beaconfield Road,
**Primary Subject Heading**:	Health services research
Secondary Subject Heading:	Infectious diseases
Keywords:	Public health < INFECTIOUS DISEASES, Community child health < PAEDIATRICS, Respiratory infections < THORACIC MEDICINE, Health policy < HEALTH SERVICES ADMINISTRATION & MANAGEMENT

SCHOLARONE™
Manuscripts

I, the Submitting Author has the right to grant and does grant on behalf of all authors of the Work (as defined in the below author licence), an exclusive licence and/or a non-exclusive licence for contributions from authors who are: i) UK Crown employees; ii) where BMJ has agreed a CC-BY licence shall apply, and/or iii) in accordance with the terms applicable for US Federal Government officers or employees acting as part of their official duties; on a worldwide, perpetual, irrevocable, royalty-free basis to BMJ Publishing Group Ltd ("BMJ") its licensees and where the relevant Journal is co-owned by BMJ to the co-owners of the Journal, to publish the Work in this journal and any other BMJ products and to exploit all rights, as set out in our licence.

The Submitting Author accepts and understands that any supply made under these terms is made by BMJ to the Submitting Author unless you are acting as an employee on behalf of your employer or a postgraduate student of an affiliated institution which is paying any applicable article publishing charge ("APC") for Open Access articles. Where the Submitting Author wishes to make the Work available on an Open Access basis (and intends to pay the relevant APC), the terms of reuse of such Open Access shall be governed by a Creative Commons licence – details of these licences and which Creative Commons licence will apply to this Work are set out in our licence referred to above.

Other than as permitted in any relevant BMJ Author's Self Archiving Policies, I confirm this Work has not been accepted for publication elsewhere, is not being considered for publication elsewhere and does not duplicate material already published. I confirm all authors consent to publication of this Work and authorise the granting of this licence.

For peer review only

Health post service readiness and use of preventive and curative services for suspected childhood pneumonia in Ethiopia: a cross-sectional study

Amare Tariku[1,2*], Yemane Berhane[2], Alemayehu Worku[2,3], Gashaw Andargie Biks[4], Lars Åke Persson[5,6], Yemisrach B. Okwaraji[5,6]

[1]Department of Human Nutrition, Institute of Public Health, College of Medicine and Health Sciences, University of Gondar, Gondar, Ethiopia

[2]Department of Epidemiology and Biostatistics and Department of Reproductive Health and Population, Addis Continental Institute of Public Health, Addis Ababa, Ethiopia

[3]School of Public Health, Addis Ababa University, Addis Ababa, Ethiopia

[4]Department of Health System and Policy, Institute of Public Health, College of Medicine and Health Sciences, University of Gondar, Gondar, Ethiopia

[5]London School of Hygiene & Tropical Medicine, London, UK

[6]Ethiopian Public Health Institute, Addis Ababa, Ethiopia

Co-authors;

Yemane Berhane, MD, PhD: yemaneberhane@addiscontinental.edu.et

Alemayehu Worku, PhD: alemayehuwy@yahoo.com

Gashaw Andargie Biks, PhD: gashawab@gmail.com

Lars Åke Persson, MD, PhD: Lars.Persson@lshtm.ac.uk

Yemisrach B. Okwaraji, PhD: yemisrach.okwaraji@lshtm.ac.uk

[*]Corresponding author:

Amare Tariku, MSc; amaretariku15@yahoo.com

For peer review only - http://bmjopen.bmj.com/site/about/guidelines.xhtml

ABSTRACT

Objective: Pneumonia is the single leading cause of infectious disease deaths in children under-five. Despite this challenge, the utilization of preventive and curative child health services remains low in Ethiopia. We investigated the association between health post service readiness and caregivers' awareness of pneumonia services, care-seeking, and utilisation of pneumonia-relevant immunisation in four Ethiopian regions.

Design and setting: This cross-sectional study was conducted in 52 districts of four regions of Ethiopia from December 2018 to February 2019. The health posts preparedness for sick child care was assessed using the World Health Organization Health Service Availability and Readiness Assessment tool. Multilevel analyses were employed to examine the associations between health post readiness and household level awareness and utilisation of services.

Participants: We included 165 health posts, 274 health extension workers (community health workers), and 4,729 caregivers with 5,787 children 2-59 months.

Outcome measures: Awareness of pneumonia treatment, care-seeking behaviour, and coverage of pentavalent-3 immunisation.

Results: Only 62.8% of health posts were ready to provide sick child care services. One-quarter of caregivers were aware of pneumonia services, and 56.8% sought an appropriate care provider for suspected pneumonia. Nearly half (49.3%) of children (12-23 months) had received pentavalent-3 immunisation. General health post readiness was not associated with caregivers' awareness of pneumonia treatment [AOR= 0.9, 95% CI 0.7 to 1.1] and utilisation of pentavalent-3 immunisation [AOR= 1.2, 95% CI 0.8 to 1.6], but negatively associated with care-seeking for childhood illnesses [AOR= 0.6, 95% CI 0.4 to 0.8].

Conclusion: We found no association between facility readiness and awareness or utilisation of child health services. There were significant deficiencies in health post preparedness for services. Caregivers had low awareness and utilisation of pneumonia-related services. The results underline the importance of enhancing facility preparedness, providing high-quality care, and intensifying demand generation efforts to prevent and treat pneumonia.

Keywords: Childhood pneumonia; Health post preparedness; Health care utilisation; Ethiopia

For peer review only - http://bmjopen.bmj.com/site/about/guidelines.xhtml

Strengths and limitations of this study

- This is the first study, which assessed the association between first-level healthcare facility readiness for services and caregivers' awareness and utilisation of pneumonia-relevant preventive and curative services in four of the most populous Ethiopian regions.
- Facility preparedness was assessed using the World Health Organization Health Service Availability and Readiness Assessment tool to generate objective and reliable information that is comparable across or within countries.
- We ascertained information on the utilisation of pneumonia-related preventive and curative services with Demographic and Health Surveys methods, assumed to reduce recall bias.
- The study covered pneumonia services and service readiness of health posts in 52 districts of four Ethiopian regions and findings may not be representative of other geographic areas and health system levels.

INTRODUCTION

Worldwide, pneumonia is responsible for one-fifth of all under-five deaths [1]. Nearly half (49%) of these deaths occur in four low- and middle-income countries, including Ethiopia [2 3]. There are effective preventive measures, including immunisation and antibiotics that can prevent or treat most cases of pneumonia. Despite these resources, children in low-income countries continue to suffer and die from pneumonia due to lack of access to or availability of these services [4].

The World Health Organization and UNICEF introduced the integrated Community Case Management (iCCM) strategy in 2004 to increase access and quality of services for childhood pneumonia, diarrhea, and malaria. Effective implementation of this strategy requires uninterrupted stock of drugs and supplies, trained community health workers, and community awareness of these services [5 6]. Nevertheless, studies in low-income countries reveal that less than a fifth of sick children were brought to relevant health services for suspected pneumonia [7 8]. This low level of care-seeking has, among other things, been attributed to the poor quality of health services [9]. A majority of facilities in these settings have reportedly not had essential drugs, supplies and trained community health workers [10-13]. The general service readiness index for sick child care has varied between 19% and 69% in reports from Sub-Saharan African countries [11 13]. The readiness has been lower in rural areas and at the lowest primary healthcare level [14-17]. In Ethiopia, earlier reports have shown a lack of iCCM drugs and supplies at health posts [9 18].

For peer review only - http://bmjopen.bmj.com/site/about/guidelines.xhtml

Inadequate preparedness and low service quality at the primary healthcare level reduce parents' trust and utilisation of health services [11 19-21]. In Ethiopia, the low utilisation of iCCM services was also attributed to absent supervision and refresher training of health extension workers [22-27]. We have earlier shown that a substantial proportion of caregivers were not aware of pneumonia-related health services and, therefore, less likely to seek care when their children got sick or get their children immunised [28 29]. The low utilisation of iCCM services has also been attributed to the lack of readiness of health posts to care for sick children [19 30 31]. Thus, there are reasons to investigate the primary-level health facility preparedness to provide child health services and whether this is associated with the coverage of pneumonia-related preventive and treatment services. In Ethiopia, under-five mortality was reported to be 55 per 1000 live births in the 2019 Demographic and Health Survey. Although reduced in recent decades, the persistently high level and continued pneumonia deaths call for intensified efforts to prevent these preventable deaths [28 32]. The Ethiopian Ministry of Health in collaboration with non-governmental organisations implemented a complex community-based intervention labelled Optimizing the Health Extension Program (OHEP) in four of the most populous regions. The OHEP aimed to contribute to reductions in under-five child mortality through enhancing utilisation of iCCM and community-based newborn care (CBNC) services [33]. This study was part of the evaluation of OHEP intervention. We aimed to examine the association between the health post readiness to serve and caregivers' awareness of pneumonia-related services, care-seeking for sick children, and whether their 12-23 months old children had got three pentavalent vaccinations.

METHODS

Study setting and design

The Ethiopian primary healthcare system typically consists of a primary hospital, a health centre and five satellite health posts. A health post is the lowest service delivery point staffed by two health extension workers serving around 5,000 rural residents. Since 2003, Ethiopia has implemented the health extension program to achieve universal coverage of primary health care for the rural population. This national program is implemented by health extension workers, and they provide basic promotive, preventive and curative services through outreach and health post-based approaches. In 2010, after a change in policy that allowed the health extension workers to treat child pneumonia, the Ethiopian Ministry of Health and partners initiated the implementation of iCCM of childhood illnesses as part of the health extension program. Under the iCCM program, the health extension workers examine, classify, and treat pneumonia[34 35].

For peer review only - http://bmjopen.bmj.com/site/about/guidelines.xhtml

The OHEP intervention had three components, i.e., community engagement activities, capacity building of health extension workers and women's development group leaders, and strengthening of the district health services' ownership and accountability of the primary newborn and child health services. The intervention was conducted in 26 intervention districts with 26 comparison districts spread in four regions of Ethiopia, namely Tigray, Amhara, Oromia and Southern Nations, Nationalities and Peoples Regions. The intervention started in 2016 and was completed in 2018. For the evaluation, baseline and end line surveys were performed. This paper reports a secondary analysis of end line cross-sectional data [33].

Participants

This study included all caregivers and children aged 2 to 59 months, who resided in 52 study districts. It also includes all health posts with one or two health extension workers per health post serving these families.

Sampling

This study was based on secondary analysis of data from the endline survey that was part of the evaluation of the OHEP intervention. Sample size for the end line survey was estimated to measure changes in care-seeking and appropriate treatment for childhood illnesses between intervention and comparison areas at baseline and endline. Assumptions considered for the sample size calculation for the main OHEP evaluation [36] were 80% power to detect differences of 15% for the reported level of care-seeking (55%) and 20% for appropriate treatment for childhood illnesses (47%) at the baseline, design effect of 1.001 and 90% completeness. Accordingly, a sample size of 12,000 households was obtained. With this number of households, 6,532 children below the age of five years were expected to be reached by the survey, of whom 368 were assumed to have any illnesses and 308 to have suspected pneumonia within two weeks before the survey.

The parent study used a sampling frame generated based on the 2007 Ethiopian Central Statistical Agency housing and population survey. Two hundred enumeration areas, i.e., clusters, were selected from 52 study districts with probability proportional to size. A two-staged cluster sampling followed by systematic sampling to select 60 representative households from each study cluster. All caregivers of children aged 2-59 months living in the selected households were interviewed. Moreover, all health posts and one or

For peer review only - http://bmjopen.bmj.com/site/about/guidelines.xhtml

two health extension workers from each health post serving the population of the study clusters were included [37].

Data collection

Data were collected using structured and pre-tested interviewer-administered questionnaires through an electronic data collection software (CSpro survey software). The questionnaires were translated into local languages (Amharic, Tigrigna, and Oromiffa) and back-translated into English. They comprised of three main modules; household, healthcare provider and health facility modules (see supplementary files 1, 2 & 3). Data collectors and supervisors, who had bachelor's degree or above, were trained for two weeks on data collection techniques, procedures, quality assurance, and ethical considerations of the study. Further detailed information about data collection and quality control is available in the published study protocol [33].

Outcomes

The outcomes of this study are caregivers' awareness of pneumonia treatment, care-seeking behaviour and coverage of pentavalent-3 immunisation as defined in the Demographic and Health Surveys [28]. The awareness of availability of pneumonia treatment was calculated as the proportion of caregivers who had heard messages regarding pneumonia treatment. Suspected pneumonia was ascertained by asking the caregiver if the child had cough combined with either fast or difficult breathing due to chest problems within two weeks before the survey. Care-seeking was defined as children with suspected pneumonia for whom advice or treatment was sought from an appropriate care provider, i.e., either government or private providers. The vaccination status of children aged 12-23 months was primarily assessed by reviewing immunisation cards. When cards were not available at home, the caregivers were requested to report the type of vaccines their children had received. Hence, coverage of pentavalent vaccination was estimated as the proportion of children 12-23 months who had received three doses of pentavalent vaccine.

Health post readiness

The readiness of health posts for sick child care was assessed using the World Health Organization Service Availability and Readiness Assessment tool [38]. Using 23 tracer items, the preparedness of facilities was shown in five domains or indices, i.e., basic amenities, basic equipment, standard precaution equipment for infection prevention, diagnostic capacity, and essential medicines. The mean availability of items

For peer review only - http://bmjopen.bmj.com/site/about/guidelines.xhtml

across the four domains of readiness was estimated by assigning equal weight to each of the items, and was expressed as a proportion. Health posts' diagnostic capacity was shown as the proportion of facilities having rapid diagnostic test for malaria. The general service readiness was calculated as the average of percentages depicting mean availability of tracer items in five indices [38]. The availability of vaccination card at the health posts was also estimated. The number of health extension workers working at the health post and the percentage of these workers trained in iCCM and who had received supportive supervision during six months before the survey were also calculated. The health post demand generation activities were recorded as the proportion of health posts showing opening days or that used community forums to deliver maternal and child health education.

Analyses

The household socio-economic status was constructed through principal component analysis of household assets, ownership of house, livestock, agricultural land, and access to utilities and infrastructures. The factor scores were summed and ranked into quintiles from the poorest to the least poor.

The study linked the household, health facility and care provider information. Our analysis was based on linked samples for outcome variables, i.e., caregivers' awareness of pneumonia treatment (n=4,934), care-seeking when the child was sick (n=613) and vaccination of 12-23 months old children with a third dose of pentavalent immunisation (n=860). Care-seeking was assessed for all childhood illness episodes, including symptoms of suspected pneumonia as reported by caregivers for the two weeks prior to the survey. The effect of clustering on three of the study outcomes was examined by estimating Intra Cluster Correlation Coefficients (ICC). A multilevel binary logistic regression model was fitted to examine the association between health post readiness and household level awareness, care-seeking and utilisation of three doses of pentavalent vaccinations. We checked for potential household-level confounders. The fitness of the model was checked through Likelihood Ratio Test. Data were analysed using Stata version 14.

Patient and public involvement

Patients or the public were not involved in the design or conduct or reporting or dissemination plans of this research.

RESULTS

For peer review only - http://bmjopen.bmj.com/site/about/guidelines.xhtml

Characteristics of caregivers and children

A total of 10,785 households from 181 study clusters, 165 health posts and 274 health extension workers were included in the survey. A total of 4,729 eligible caregivers with 5,787 children aged 2-59 months were included in the analyses. A majority of the caregivers had no schooling (59.4%) and were currently married (88.6%). About two-thirds (64.0%) of caregivers were able to access the nearest health facility within 30 minutes of walk from their home (Table 1).

Table 1 Socio-demographic characteristics of caregivers and children aged 2-59 months in four regions of Ethiopia, December 2018 to February 2019

For peer review only

Characteristics	Frequency	Percentage
Caregivers' characteristics (n=4,729)		
Age		
<25	885	18.7
25-29	1,281	27.1
30-34	1,038	22.0
35-39	867	18.3
>=40	658	13.9
Marital status		
Currently married	4,067	88.6
Unmarried	521	11.4
Education		
No schooling	2,810	59.4
Schooling	1,919	40.6
Number of children under five		
1	3,487	73.7
2	1,148	24.3
3+	94	2.0
Socio-economic quintiles		
Q1 (poorest)	1,024	21.7
Q2	982	20.8
Q3	874	18.5
Q3	895	18.9
Q5 (least poor)	954	20.1
Walking distance from household to nearest health facility (n=3,918)		
<=30 minutes	2,507	64.0
>30 minute	1,411	36.0
Child (2-59 months) characteristics (n=5,787)		
Sex		
Male	2,961	51.2
Female	2,826	48.8
Age		
2-11 Months	959	16.6
12-23 Months	992	17.1
24-35 Months	1114	19.3
36-59 Months	2722	47.0

For peer review only - http://bmjopen.bmj.com/site/about/guidelines.xhtml

Characteristics of health posts and health extension workers

The median number of households served by the health post was 1,181. The majority (85.1%) of the health posts were functionally open for five days or more per week. More than a tenth (12.7%) of the health posts were served by only one health extension worker. Most (78.8%) of the health extension workers were trained in the iCCM of childhood illnesses. More than three-fourth (78.8%) had received supportive supervision within six months preceding the survey. Three-quarters used community forums or meetings to deliver maternal and child health education within three months prior to the survey (Table 2).

Table 2 Characteristics of health posts in four regions of Ethiopia, December 2018 to February 2019

Characteristics	Frequency	Percentage
Health posts catchment area population (n=165)		
Number of households, median (Inter-quartile range)	1,181 (841, 1572)	
Number of children under five years, median (Inter-quartile range)	780 (493, 1065)	
Health extension workers' characteristics (274)		
Number of health extension workers per health post		
One	35	12.7
Two	142	51.8
Three and above	97	35.4
Health post opening days per week		
1 to 4 days	41	14.9
5 to 7 days	233	85.1
Posted health post opening days	54	19.7
Trained for iCCM* of childhood illnesses	216	78.8
Received supportive supervision in the last six months	216	78.8
Participated in Performance Review and Clinical Mentorship meetings	126	46.0
Used community forums to deliver maternal and child health education	205	74.8

* Integrated community case management

Health post preparedness to deliver sick child care services

The general service readiness of health posts (n=165) to deliver sick child health services was estimated at 62.8%. Half of the health posts had Rapid Diagnostic Test for malaria, and the mean availability of essential medicines was 66.9%. Relatively higher mean availability (80.1%) of tracer items was shown for basic equipment, while the lowest (48.7%) was for availability of standard precaution items. Very few health posts had all essential medicines and standard precaution equipment. Most of the health posts (84.2%) had vaccination cards (Figure 1).

For peer review only

For peer review only - http://bmjopen.bmj.com/site/about/guidelines.xhtml

229 Figure 1 Percentage of health posts (n=165) with drugs and supplies to deliver pneumonia-related and
230 other sick child care services in four regions of Ethiopia, December 2018 to February 2019

231 **Awareness of treatment, actual care-seeking and utilisation of preventive immunisation**

232 During the two weeks before the survey 704 (12.3%) of the children had any illnesses. Of these, 44 children
233 had symptoms and signs of suspected pneumonia. Only one in five (23.9%) caregivers were aware of the
234 availability of pneumonia treatment services. Health care was sought for one-third (36.1%) of children
235 with any illnesses and for 56.8% of children with suspected pneumonia. Almost half (49.3%) of children
236 aged 12-23 months had received three doses of pentavalent vaccinations (Table 3).

237 Table 3 Childhood illness and care utilisation by child demographic characteristics in four regions of
238 Ethiopia, December 2018 to February 2019

Service utilisation	Frequency	Percentage
Children with any illnesses		
Yes	704	12.3
No	5,021	87.7
Childhood illnesses by sex (n=704)		
Male	362	51.4
Female	342	48.6
Childhood illnesses by age (n=704)		
2-11 Months	121	17.2
12-23 Months	147	20.9
24-35 Months	152	21.6
36-59 Months	284	40.3
Child with suspected pneumonia		
Yes	44	0.8
No	5,743	99.2
Child with suspected pneumonia treated with antibiotics (n=44)		
Yes	17	38.6
No	27	61.4
Full pentavalent vaccination by sex (n=489)		
Male	258	52.8
Female	231	47.2

239

240 **Association between health post readiness and awareness and utilisation of pneumonia care**

241 The Intra Cluster Correlation Coefficient (ICC) indicated that the study outcomes, i.e., awareness of
242 pneumonia treatment (ICC=0.29, 95% CI: 0.24, 0.36), care-seeking behaviour (ICC=0.16, 95% CI: 0.10, 0.27)
243 and pentavalent vaccination (ICC=0.28, 95% CI: 0.20, 0.38) significantly varied with level of clusters. While

For peer review only - http://bmjopen.bmj.com/site/about/guidelines.xhtml

checking for the household-level confounders, we found that none of the household, caregiver and child characteristics were associated with the outcomes and main exposure variables, i.e., the five health post readiness indices. But regardless of the statistical screening criteria (P-value <0.2), analyses were adjusted for maternal age, education and area of residence (intervention-comparison area) variables.

Analyses tested the association of general health post readiness index with study outcomes were adjusted for health extension workers' iCCM training, supportive supervision, participation at performance review and clinical mentorship meeting, home vising, use of community forums, opening days and number of staff at health post, mothers' age, education and area of residence (intervention-comparison area) variables. The adjusted multilevel analyses revealed that general health post readiness was not associated with caregivers' awareness of availability of pneumonia treatment [AOR= 0.9, 95% CI 0.7 to 1.1] or utilisation of pentavalent-3 immunisation [AOR= 1.2, 95% CI 0.8 to 1.6]. The general health post readiness was negatively associated with care-seeking for childhood illnesses [AOR= 0.6, 95% CI 0.4 to 0.8], Table 4.

Table 4 General health post readiness and caregivers' awareness, care-seeking and utilisation of pentavalent-3 immunisation in four regions of Ethiopia, December 2018 to February 2019

**Awareness and utilisation**	**General readiness**	
	**Crude Odds Ratio (95% CI)**	**Adjusted Odds Ratio (95% CI)**
Awareness of treatment service (N= 4,934) *	0.9 (0.7,1.1)	0.9 (0.7,1.1)
Care-seeking (N=613) *	0.7 (0.5,0.9)	0.6 (0.4,0.8)
Pentavalent-3 (N=860) *	1.2 (0.9,1.6)	1.2 (0.8,1.6)

* Analyses adjusted for health extension workers' iCCM training, supportive supervision, participation at performance review and clinical mentorship meeting, home vising, use of community forums, opening days and number of staff at health post, mothers' age, education and area of residence.

As illustrated in Table-5, none of the health post readiness indices were associated with caregivers' awareness of availability of pneumonia treatment and care-seeking for childhood illnesses. Only availability of standard precaution equipment for infection prevention was positively associated with utilisation of pentavalent-3 immunisation [AOR= 4.5, 95% CI 1.6 to 12.8]. Home visiting by the health extension workers was associated with higher odds for caregivers' awareness of availability of pneumonia treatment [AOR= 2.9, 95% CI 2.3 to 3.6].

For peer review only - http://bmjopen.bmj.com/site/about/guidelines.xhtml

Table 5 Health post preparedness indices associated with caregivers' awareness, care-seeking and utilisation of three doses of pentavalent vaccines in four regions of Ethiopia, December 2018 to February 2019

Characteristics	Awareness of treatment service (N= 4,934)				Care-seeking (N=613)				Pentavalent-3 (N=860)			
	Aware	Unaware	Crude Odds Ratio (95% CI)	Adjusted Odds Ratio (95% CI)	Sought care	Didn't seek care	Crude Odds Ratio (95% CI)	Adjusted Odds Ratio (95% CI)	Vaccinated	Not vaccinated	Crude Odds Ratio (95% CI)	Adjusted Odds Ratio (95% CI)
Basic amenities												
All not available	923	2,867	1.0	1.0	161	270	1.0	1.0	325	337	1.0	1.0
All available	256	888	0.9 (0.6,1.5)	0.9 (0.6,1.5)	49	133	0.7 (0.4,1.1)	0.7 (0.4,1.2)	98	100	0.9 (0.6,1.8)	0.9 (0.6,1.8)
Basic equipment												
All not available	590	2,021	1.0	1.0	117	202	1.0	1.0	219	224	1.0	1.0
All available	589	1,734	1.3 (0.9,1.9)	1.2 (0.8,1.9)	93	201	0.8 (0.5,1.3)	0.9 (0.5,1.4)	204	213	0.9 (0.6,1.6)	0.8 (0.5,1.4)
Standard precaution equipment for infection prevention												
All not available	1,091	3,555	1.0	1.0	202	379	1.0	1.0	386	414	1.0	1.0
All available	88	200	1.9 (0.9,3.9)	1.9 (0.9,4.2)	8	24	0.6 (0.2,1.7)	0.8 (0.3,2.1)	37	23	2.3 (0.9,5.6)	4.5 (1.6,12.8)
Rapid diagnostic test for malaria												
No available	597	1,858	1.0	1.0	NA	NA	NA	NA	NA	NA	NA	NA
Available	582	1,897	0.8 (0.5,1.2)	0.7 (0.4,1.1)	NA	NA	NA	NA	NA	NA	NA	NA
Essential medicine												
First tercile	354	1025	1.0	1.0	58	98	1	1	108	126	1.0	1.0
Second tercile	396	1355	0.9 (0.5,1.4)	0.9 (0.5,1.4)	76	142	0.8 (0.4,1.4)	0.8 (0.4,1.5)	129	145	0.9 (0.5,1.8)	0.8 (0.4,1.6)
Third tercile	429	1375	0.8 (0.5,1.3)	0.8 (0.5,1.4)	76	163	0.7 (0.4,1.3)	0.7 (0.4,1.4)	186	166	1.3 (0.7,2.3)	1.1 (0.6,2.1)
Number of health extension workers per health post												
One	226	784	1.0	1.0	47	71	1.0	1.0	73	68	1.0	1.0
Two and above	953	2,971	1.2 (0.7,1.9)	1.2 (0.7,1.9)	163	332	0.8 (0.4,1.4)	0.8 (0.5,1.5)	350	369	0.9 (0.5,1.6)	0.7 (0.4,1.4)
Number of health post opening days												
Less than five days	209	582	1.0	1.0	43	71	1.0	1.0	61	67	1.0	1.0

For peer review only - http://bmjopen.bmj.com/site/about/guidelines.xhtml

Five days and above	970	3,173	0.8 (0.5,1.3)	0.7 (0.4,1.2)	167	332	0.9 (0.5,1.5)	0.7 (0.4,1.4)	362	370	1.3 (0.7,2.5)	1.4 (0.7,2.8)
Health extension workers used community forum												
No	297	1,003	1.0	1.0	70	139	1.0	1.0	113	131	1.0	1.0
Yes	882	2,752	1.2 (0.8,1.8)	1.3 (0.8,1.9)	140	264	1.0 (0.6,1.6)	0.9 (0.6,1.5)	310	306	1.3 (0.8,2.2)	1.3 (0.7,2.2)
Health extension workers received supervision												
No	NA	NA	NA	NA	44	105	1.0	1.0	60	68	1.0	1.0
Yes	NA	NA	NA	NA	166	298	1.3 (0.8,2.3)	1.5 (0.8,2.6)	363	369	1.4 (0.7,2.5)	1.1 (0.5,2.2)
Health extension workers received iCCM training												
Did not received training	NA	NA	NA	NA	40	74	1.0	1.0	NA	NA	NA	NA
Received training	NA	NA	NA	NA	170	329	0.9 (0.5,1.6)	0.9 (0.5,1.7)	NA	NA	NA	NA
Health extension workers participated at Performance Review and Clinical Mentorship meeting												
No	NA	NA	NA	NA	123	252	1.0	1.0	232	252	1.0	1.0
Yes	NA	NA	NA	NA	87	151	1.2 (0.8,1.9)	1.2 (0.7,2.0)	191	185	1.2 (0.7,1.9)	0.9 (0.5,1.5)
Home vising												
No	934	3,445	1	1	175	357	1	1	372	386	1.0	1.0
Yes	244	309	2.9 (2.4,3.7)	2.9 (2.3,3.6)	35	46	1.6 (0.9,2.6)	1.5 (0.9,2.5)	51	38	1.6 (0.9,2.7)	1.6 (0.9,2.7)

For peer review only - http://bmjopen.bmj.com/site/about/guidelines.xhtml

DISCUSSION

Overall, this study showed insufficient health post service preparedness and low household awareness and utilisation of preventive and curative services for childhood suspected pneumonia. There was no consistent association between facility preparedness and utilisation of preventive and curative services.

Strengths and limitations

With an attempt of narrowing the existing scarcity of evidence, our study examined the association between health post readiness and household-level awareness and utilisation of pneumonia relevant health services in a wider geographic area. Readiness of health posts was measured using the standard WHO Health Service Availability and Readiness Assessment tool. This tool is presumed to generate objective and reliable information that is comparable across or within countries. We pretested and adopted the tool to the local context and to level of care provided at health posts [38]. When vaccination cards were not available at home, children's pentavalent immunisation status was assessed through interviewing the caregivers. Ascertainment of childhood suspected pneumonia and other illnesses was based on the caregivers' two weeks reported symptoms prior to the survey. These ascertainment techniques have been used by the Demographic and Health Surveys, but might anyhow be influenced by recall bias [28]. Furthermore, we assessed health post service readiness and pneumonia service utilisation in selected districts of four Ethiopian regions. The findings may not be generalizable to other geographic areas and health system levels.

Health post preparedness

This study showed that two-thirds of the health posts were ready to provide sick child care, suggesting that the remaining facilities were not able to deliver such services. More or less similar level of structural preparedness of health posts or primary health care facilities for pneumonia and other sick child care were previously reported in Ethiopia and from other low- and middle-income countries [10 39 40]. Furthermore, health posts or health centres of Ethiopia and other low-income countries were found with low readiness to provide quality care to sick children [9]. According to the Ethiopia health system, a health centre is structured to support and strengthen five health posts within their catchment areas, hence insufficient preparedness of the surveyed health posts could be explained by scarcity of supplies at health centres[29 41]. The weak linkage and inadequate support from the health centres or the health system could further cause scarcity of drugs and supplies and unpreparedness of staff to serve at health posts[22 42]. The lack of

For peer review only - http://bmjopen.bmj.com/site/about/guidelines.xhtml

readiness at health posts could also be related to the donor-dependent nature of supplies and the health extension workers' lack of accountability and capacity in supply-chain management[34 43 44].

Inadequate readiness of health posts in the study setting and other low-income countries indicates a serious challenge to community case management of pneumonia, particularly in the rural or unreached communities, where a majority of preventable deaths occur [6 28]. Most importantly, only a few of the surveyed health posts had all essential medicines and just half had diagnostic equipment, clearly indicating their limitations in providing effective pneumonia or sick child treatment [5 6]. Scarcity of essential medicines in Ethiopia and other Sub-Saharan African countries results in missed pneumonia treatments at facility level [13 17 29]. Unavailability of rapid diagnostic tests impairs community health workers' ability to differentiate suspected pneumonia from malaria in case of symptom overlap, a common clinical problem in African children [45-47].

Awareness and utilisation of pneumonia-related health services

Our study revealed low health care utilisation for pneumonia-specific preventive and curative services. These levels of service utilisation were lower compared to the reported regional pentavalent-3 immunisation coverage (80%) and care-seeking behaviour (85%) for childhood suspected pneumonia and other illnesses in Sub-Saharan African Countries [48 49]. Community awareness of illness and sick child care is a pre-requisite for timely utilisation of health services [50]. We found that less than a fifth of caregivers were aware of the availability of pneumonia treatment services, and this might partly explain the observed poor utilisation of pneumonia-related health services in the study setting [51 52]. The reported low care utilisation could also be explained by inadequate readiness or service quality of the primary health care facilities for pneumonia-related preventive and treatment services [39 53]. The OHEP evaluation studies have revealed a low quality of sick child care services provided at the primary health care facilities, and caregivers of children have also mentioned this as a key barrier to seek care at health posts [54-56]. A study in 22 African countries noted a low level of community trust in public health facilities as an important reason to the low coverage of child vaccination [57].

Association between health post preparedness and utilisation of preventive immunisation and care-seeking behaviour

Earlier studies have shown a positive association between health facility readiness and utilisation of first-level sick child care [11 21]. We did not find any consistent pattern of relationship between facility readiness indices and utilisation of services. There was a positive association between the availability of standard

For peer review only - http://bmjopen.bmj.com/site/about/guidelines.xhtml

precaution equipment for infection prevention and utilisation of pentavalent-3 immunisation. However, the general health post readiness had no association with awareness and coverage of pentavalent-3 immunisation, but a negative association with care-seeking for childhood suspected pneumonia and other illnesses. Studies in Haiti and Ethiopia have shown absence of association between readiness of primary health care facilities for sick child care and caregivers' utilization and satisfaction to the respective services [40 58]. The lack of consistent positive association may be linked to the dominating low level of health post preparedness. The community's value to quality of healthcare service is a key driver of their decision to seek care, and this may subserve the lack of consistent association between facility readiness and utilization of pneumonia services. Irrespective of the readiness of health posts, caregivers' preconceived lack of trust to quality of primary child health services could motivate the use of other facilities with perceived higher service quality[54]. Equipping health care facilities with relevant equipment is a prerequisite to enhance the quality of iCCM services [6]. Hence, the reported lack of association of health post readiness with awareness and utilisation of pneumonia treatment services suggests that simply equipping facilities with necessary supplies is not a guarantee to reach the intended level of community awareness and utilisation of health services. Health facility strengthening efforts should go along with implementation of awareness creation and demand generation interventions to increase the community awareness, trust and utilisation of pneumonia-related and other child health services [50]. Our previous study showed higher parents' care-seeking for childhood suspected pneumonia among those with improved awareness of treatment service[59]. The current study also illustrated that parents' awareness of pneumonia treatment was higher when health extension workers had visited at home. Home visiting by the community health workers is a vital strategy to promote child health and enhance awareness and utilisation of health services [50 60]. A sub-study of the same project also showed that awareness creation and delivery of preventive child health interventions (such as immunisations) were the main components of outreach services delivered by the health extension workers [54].

Relation of findings with already published OHEP evaluation studies

This study was part of the end line evaluation of the OHEP intervention. The findings showed sizable gaps in structural readiness of health posts for sick child care. Results of our published baseline study also illustrated the scarcity of essential drugs and other supplies at the health posts [29]. Sub-studies of the same project investigated quality of sick child care, showing low clinical performance of the health extension workers to identify and treat childhood suspected pneumonia and other illnesses [55 56]. A qualitative study noted lack of caregivers' trust in the health extension workers' clinical competency to manage sick

For peer review only - http://bmjopen.bmj.com/site/about/guidelines.xhtml

children and a low availability of essential drugs, diagnostics and other supplies at health posts. The low quality of sick child care at health posts is a barrier to utilise these services [54].

CONCLUSIONS

This study has shown a low health post readiness for services, and low household awareness and utilisation of pneumonia-relevant preventive and curative services. Parents' awareness and utilisation of pneumonia-specific preventive and curative services were not consistently associated with the health post readiness. The results underline the critical importance of intensifying the health extension workers' awareness creation and demand generation efforts in each kebele (the lowest administrative unit in Ethiopia). Enhancing the coverage of home visiting and other awareness creation activities are crucial to boost community awareness and utilization of pneumonia and other sick child care services. Our findings also underline the pivotal role of improving the availability and quality of pneumonia and other sick child care services to ensure optimal uptake of the services. It is imperative that the district health offices strengthen the linkages within the primary healthcare units to increase the availability of essential medicines and readiness of the health posts for sick child care. The office should also optimize the availability of essential medicines and supplies at health centers that are the suppliers to the satellite health posts. Improving the coverage of regular supportive supervision, performance reviews and clinical mentorship could also help to timely identify and solve gaps in the availability of drug and other supplies at health posts. Community awareness creation and demand generation efforts should simultaneously be accompanied with health facility strengthening strategies.

REFERENCES

1. Liu L, Johnson HL, Cousens S, et al. Global, regional, and national causes of child mortality: an updated systematic analysis for 2010 with time trends since 2000. The Lancet 2012;**379**(9832):2151-61.
2. United Nations Inter-agency Group for Child Mortality Estimation (UN IGME), 'Levels & Trends in Child Mortality: Report 2020, Estimates developed by the United Nations Inter-agency Group for Child Mortality Estimation', United Nations Children's Fund, New York, 2020.
3. McAllister DA, Liu L, Shi T, et al. Global, regional, and national estimates of pneumonia morbidity and mortality in children younger than 5 years between 2000 and 2015: a systematic analysis. The Lancet Global Health 2019;**7**(1):e47-e57.
4. Bhutta ZA, Das JK, Walker N, et al. Interventions to address deaths from childhood pneumonia and diarrhoea equitably: what works and at what cost? The Lancet 2013;**381**(9875):1417-29.
5. World Health Organization. *Management of pneumonia in community settings*: World Health Organization, Department of Child and Adolescent Health and …, 2004.
6. World Health Organization. WHO/UNICEF joint statement integrated community case management (iCCM): an equity-focused strategy to improve access to essential treatment services for children. Geneva and New York 2012.

For peer review only - http://bmjopen.bmj.com/site/about/guidelines.xhtml

7. Geldsetzer P, Williams TC, Kirolos A, et al. The recognition of and care seeking behaviour for childhood illness in developing countries: a systematic review. PloS one 2014;**9**(4):e93427.
8. Adedokun ST, Yaya S. Factors influencing mothers' health care seeking behaviour for their children: evidence from 31 countries in sub-Saharan Africa. BMC Health Services Research 2020;**20:842**.
9. Macarayan EK, Gage AD, Doubova SV, et al. Assessment of quality of primary care with facility surveys: a descriptive analysis in ten low-income and middle-income countries. The Lancet Global Health 2018;**6**(11):e1176-e85.
10. Acharya K B, J, and Tuladhar S,. Client Satisfaction and Quality of Curative Services for Sick Children in Nepal. DHS Further Analysis Reports No. 114. Rockville, Maryland, USA: ICF. 2018.
11. Liu L, Leslie HH, Joshua M, et al. Exploring the association between sick child healthcare utilisation and health facility quality in Malawi: a cross-sectional study. BMJ open 2019;**9**(7):e029631.
12. Shawon MSR, Adhikary G, Ali MW, et al. General service and child immunization-specific readiness assessment of healthcare facilities in two selected divisions in Bangladesh. BMC health services research 2018;**18**(1):39.
13. Taylor C, Linn A, Wang W, et al. Examination of malaria service utilization and service provision: an analysis of DHS and SPA data from Malawi, Senegal, and Tanzania. Malaria journal 2019;**18**(1):258.
14. Blanas DA, Ndiaye Y, Nichols K, et al. Barriers to community case management of malaria in Saraya, Senegal: training, and supply-chains. Malaria journal 2013;**12**(1):95.
15. Masters SH, Burstein R, DeCenso B, et al. Pharmaceutical availability across levels of care: evidence from facility surveys in Ghana, Kenya, and Uganda. PloS one 2014;**9**(12):e114762.
16. Ssempiira J, Kasirye I, Kissa J, et al. Measuring health facility readiness and its efects on severe malaria outcomes in Uganda. Scientific Reports 2018.
17. Bagonza J, Rutebemberwa E, Eckmanns T, et al. What influences availability of medicines for the community management of childhood illnesses in central Uganda? Implications for scaling up the integrated community case management programme. BMC Public Health 2015;**15**(1):1-8.
18. International EPHIEFMoHI. Ethiopia Service Provision Assessment Plus Survey. 2014.
19. Shaw B, Amouzou A, Miller NP, et al. Determinants of utilization of health extension workers in the context of scale-up of integrated community case management of childhood illnesses in Ethiopia. The American journal of tropical medicine and hygiene 2015;**93**(3):636-47.
20. Anselmi L, Lagarde M, Hanson K. Health service availability and health seeking behaviour in resource poor settings: evidence from Mozambique. Health Economics Review (2015) 5:26
21. Kuwawenaruwa A, Wyss K, Wiedenmayer K, et al. The effects of medicines availability and stock-outs on household's utilization of healthcare services in Dodoma region, Tanzania. Health policy and planning 2020;**35**(3):323-33.
22. Kok MC, Kea AZ, Datiko DG, et al. A qualitative assessment of health extension workers' relationships with the community and health sector in Ethiopia: opportunities for enhancing maternal health performance. Human resources for health 2015;**13**(1):80.
23. Befekadu A, Yitayal M. Knowledge and practice of health extension workers on drug provision for childhood illness in west Gojjam, Amhara, Northwest Ethiopia. BMC Public Health 2020;**20**:1-10.
24. Miller NP, Amouzou A, Tafesse M, et al. Integrated community case management of childhood illness in Ethiopia: implementation strength and quality of care. The American journal of tropical medicine and hygiene 2014;**91**(2):424-34.
25. Tilahun H, Fekadu B, Abdisa H, et al. Ethiopia's health extension workers use of work time on duty: time and motion study. Health policy and planning 2017;**32**(3):320-28.
26. Datiko DG, Bunte EM, Birrie GB, et al. Community participation and maternal health service utilization: lessons from the health extension programme in rural southern Ethiopia. Journal of Global Health Reports 2019;**3**.

For peer review only - http://bmjopen.bmj.com/site/about/guidelines.xhtml

27. Mangham-Jefferies L, Mathewos B, Russell J, et al. How do health extension workers in Ethiopia allocate their time? Human resources for health 2014;**12**(1):61.

28. Central Statistical Agency [Ethiopia] and ICF International. Ethiopia Demographic and Health Survey 2016. Addis Ababa, Ethiopia and Calverton, Maryland, USA: Central Statistical Agency and ICF International. 2017.

29. Tariku A, Okwaraji YB, Worku A, et al. Prevention and treatment of suspected pneumonia in Ethiopian under-five children from household to primary care. Acta Paediatrica 2020.

30. Berhanu A, Alemayehu M, Kassa Daka WB, et al. Utilization of Integrated Community Case Management of Childhood Illnesses at Health Posts in Southern Ethiopia. Pediatric Health, Medicine and Therapeutics 2020;**11**:459.

31. Ethiopian Public Health Institute: World Health Organization. Ethiopia Service Availability and Readiness Assessment 2016 Summary Report. 2016.

32. Deribew A, Tessema GA, Deribe K, et al. Trends, causes, and risk factors of mortality among children under 5 in Ethiopia, 1990–2013: findings from the Global Burden of Disease Study 2013. Population health metrics 2016;**14**(1):42.

33. Berhanu D, Okwaraji YB, Belayneh AB, et al. Protocol for the evaluation of a complex intervention aiming at increased utilisation of primary child health services in Ethiopia: a before and after study in intervention and comparison areas. BMC Health Services Research 2020;**20**:1-12.

34. Ministry of Health (2020). Realizing Universal Health Coverage through Primary Health Care: A Roadmap for Optimizing the Ethiopian Health Extension Program 2020 – 2035. Addis Ababa, Ethiopia: Ministry of Health.

35. Wang, Huihui, Roman Tesfaye, Gandham N. V. Ramana, Chala Tesfaye Chekagn. 2016. Ethiopia Health Externsion Program: An Institutionalized Community Approach for Universal Health Coverage. World Bank Studies. Washington, DC: World Bank.doi: 10.1596/978-1-4648-0815-9. License: Creative Commons Attribution CC BY3.0 IGO.

36. Berhanu D, Okwaraji YB, Defar A, et al. Does a complex intervention targeting communities, health facilities and district health managers increase the utilisation of community-based child health services? A before and after study in intervention and comparison areas of Ethiopia. BMJ open 2020;**10**(9):e040868.

37. Schellenberg J, Persson LÅ, Defar A, et al. Endline evaluation of improved integrated community case management and community-based newborn care in Ethiopia: Ethiopian Public Health Institute & London School of Hygien and Tropical Medicine 2018.

38. Organization WH. Service availability and readiness assessment (SARA): an annual monitoring system for service delivery: reference manual: World Health Organization, 2013.

39. Hailemariam S, Gebeyehu Y, Loha E, et al. Inadequate management of pneumonia among children in South Ethiopia: findings from descriptive study. BMC health services research 2019;**19**(1):426.

40. Gage AD, Leslie HH, Bitton A, et al. Does quality influence utilization of primary health care? Evidence from Haiti. Globalization and health 2018;**14**(1):1-9.

41. Sousa Ad, Tiedje KE, Recht J, et al. Community case management of childhood illnesses: policy and implementation in Countdown to 2015 countries. Bull World Health Organ 2012;**90:183–190**.

42. Assefa Y, Gelaw YA, Hill PS, et al. Community health extension program of Ethiopia, 2003–2018: successes and challenges toward universal coverage for primary healthcare services. Globalization and health 2019;**15**(1):1-11.

43. Chandani Y, Noel M, Pomeroy A, et al. Factors affecting availability of essential medicines among community health workers in Ethiopia, Malawi, and Rwanda: solving the last mile puzzle. The American journal of tropical medicine and hygiene 2012;**87**(5 Suppl):120.

44. Ademe BW, Tebeje B, Molla A. Availability and utilization of medical devices in Jimma zone hospitals, Southwest Ethiopia: a case study. BMC health services research 2016;**16**(1):1-10.

For peer review only

For peer review only - http://bmjopen.bmj.com/site/about/guidelines.xhtml

45. Ukwaja KN, Aina OB, Talabi AA. Clinical overlap between malaria and pneumonia: can malaria rapid diagnostic test play a role? The Journal of Infection in Developing Countries 2011;**5**(03):199-203.
46. Kyabayinze DJ, Asiimwe C, Nakanjako D, et al. Use of RDTs to improve malaria diagnosis and fever case management at primary health care facilities in Uganda. Malaria journal 2010;**9**(1):1-10.
47. Yeboah-Antwi K, Pilingana P, Macleod WB, et al. Community case management of fever due to malaria and pneumonia in children under five in Zambia: a cluster randomized controlled trial. PLoS Med 2010;**7**(9):e1000340.
48. Ahinkorah BO, Budu E, Seidu A-A, et al. Barriers to healthcare access and healthcare seeking for childhood illnesses among childbearing women in sub-Saharan Africa: A multilevel modelling of Demographic and Health Surveys. PLoS ONE 16(2): e0244395 2021.
49. Fenta SM, Biresaw HB, Fentaw KD, et al. Determinants of full childhood immunization among children aged 12– 23 months in sub-Saharan Africa: a multilevel analysis using Demographic and Health Survey Data. Tropical Medicine and Health (2021) 49:29.
50. Miller NP, Bagheri Ardestani F, Wong H, et al. Barriers to the utilization of community-based child and newborn health services in Ethiopia: a scoping review. Health Policy and Planning 2021.
51. Sadruddin S, Khan IuH, Bari A, et al. Effect of community mobilization on appropriate care seeking for pneumonia in Haripur, Pakistan. Journal of Global Health 2015;**5 (1)**.
52. Noordam AC, Sharkey AB, Hinssen P, et al. Association between caregivers' knowledge and care seeking behaviour for children with symptoms of pneumonia in six subSaharan African Countries. BMC Health Services Research (2017) 17:107
53. Tadesse T, Gelaw B, Haile Y, et al. Immunization service availability and readiness in primary health care in pastoral and semi-pastoral CGPP Ethiopia implementation districts. Ethiopian Journal of Health Development 2019;**33**.
54. Defar A, Alemu K, Tigabu Z, et al. Caregivers' and Health Extension Workers' Perceptions and Experiences of Outreach Management of Childhood Illnesses in Ethiopia: A Qualitative Study. International journal of environmental research and public health 2021;**18**(7):3816.
55. Daka DW, Wordofa MA, Woldie M, et al. Quality of clinical assessment and management of sick children by Health Extension Workers in four regions of Ethiopia: A cross-sectional survey. PLoS ONE 2020;**15(9): e0239361**.
56. Getachew T, Mekonnen S, Yitayal M, et al. Health Extension Workers' diagnostic accurac y for common childhood illnesses in four regions of Ethiopia: a cross-sectional study. Acta Pædiatrica 2019.
57. Stoop N, Hirvonen K, Maystadt J-F. Institutional mistrust and child vaccination coverage in Africa. BMJ Global Health 2021;6:e004595 doi:101136/bmjgh-2020-004595.
58. Getachew T, Abebe SM, Yitayal M, et al. Assessing the quality of care in sick child services at health facilities in Ethiopia. BMC Health Services Research 2020;**20**(1):1-12.
59. Tariku A, Berhane Y, Worku A, et al. Household wealth and utilization of preventive and treatment services for suspected childhood pneumonia in Ethiopia. *Submitted for publication at PLOS ONE* 2021.
60. Tripathi A, Kabra S, Sachdev H, et al. Home visits by community health workers to improve identification of serious illness and care seeking in newborns and young infants from low-and middle-income countries. Journal of Perinatology 2016;**36**(1):S74-S82.

For peer review only - http://bmjopen.bmj.com/site/about/guidelines.xhtml

Acknowledgements

We would like to forward our deepest gratitude to the study participants. Our special thanks to field assistants involved in the data collection process.

Contributors

AT, LÅP, YB, YO, AW and GAB contributed to the conceptualization of the study. AT analysed and interpreted the data and drafted the manuscript. LÅP, YB, YO, AW and GAB contributed to analysis and writing of the paper. All authors have read and approved the final manuscript.

Funding

The study was funded by a grant from the Bill and Melinda Gates Foundation (grant INV-009691) to the London School of Hygiene & Tropical Medicine. The funder had no role in data collection, analysis, or interpretation of results.

Competing interests

The authors declare that they have no conflict of interest.

Patient consent for publication

Not applicable

Ethics approval

The original study was approved by the Ethical Review Boards of the Ethiopian Public Health Institute (protocol number SERO-012-8-2016), the London School of Hygiene & Tropical Medicine (protocol number 11235), and the University of Gondar (V/P/RCS/05/559/2019). A written informed consent was obtained from each household respondent, caregiver of the index child and the health workers.

Provenance and peer review

Not commissioned; externally peer reviewed.

Data availability statement

For peer review only - http://bmjopen.bmj.com/site/about/guidelines.xhtml

The data for this manuscript were primarily collected by the Ethiopian Public Health Institute and London School of Hygiene & Tropical Medicine. Interested researchers may contact the focal person, Dr Yemisrach B. Okwaraji through email: Yemisrach.Okwaraji@lshtm.ac.uk. All requests will be reviewed by this committee and if granted, data will be shared without any identifiers.

Figure legend

Figure 1 Percentage of health posts (n=165) with drugs and supplies to deliver pneumonia-related and other sick child care services in four regions of Ethiopia, December 2018 to February 2019

Supplementary files

Supplementary file 1 Household survey questionnaire

Supplementary file 2 Health post survey questionnaire

Supplementary file 3 Health extension worker survey questionnaire

For peer review only

For peer review only - http://bmjopen.bmj.com/site/about/guidelines.xhtml

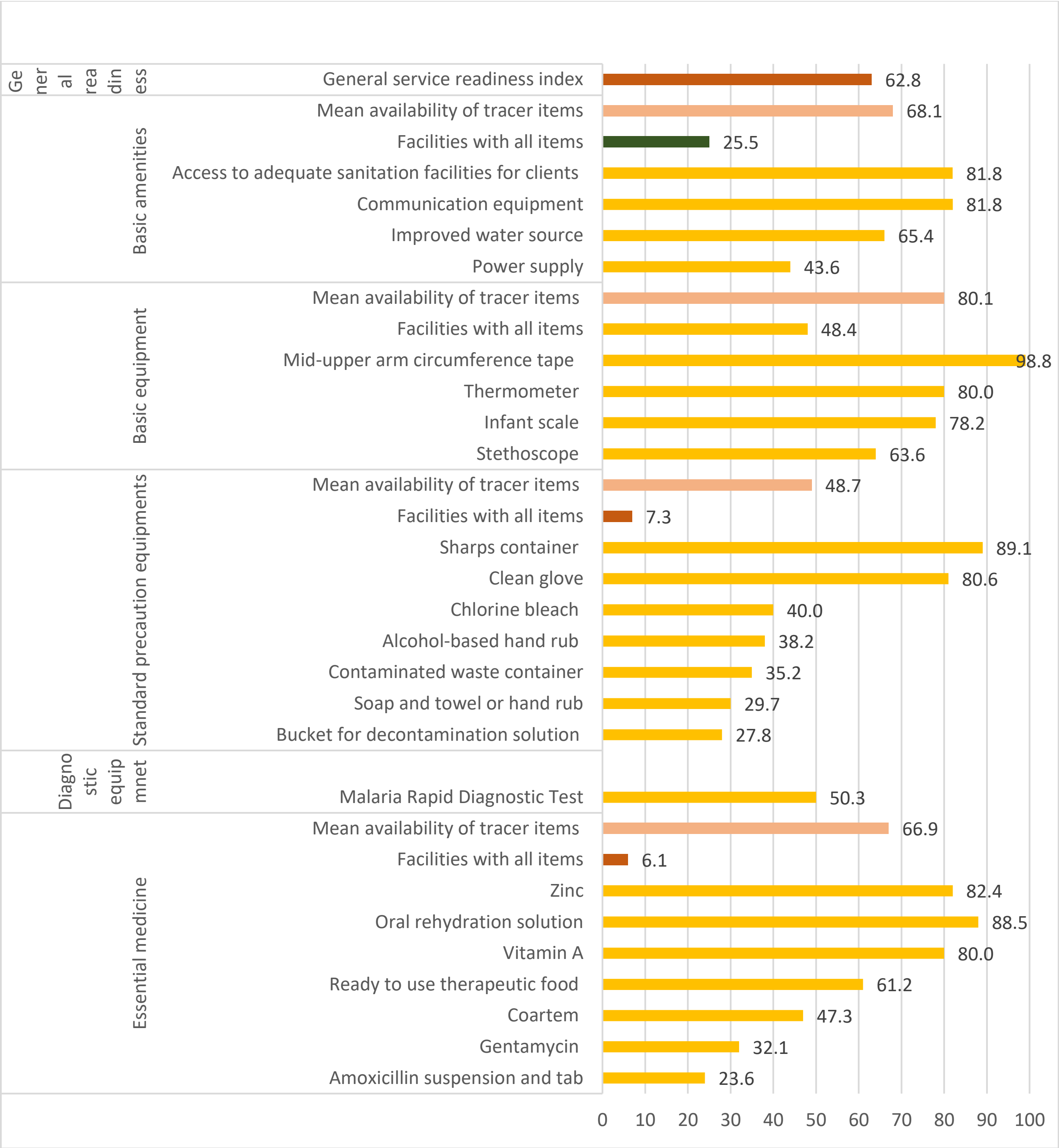

Figure 1 Percentage of health posts (n=165) with drugs and supplies to deliver pneumonia-related and other sick child care services in four regions of Ethiopia, December 2018 to February 2019

Dagu End line Household Survey 2018

Module 1 – Household

Section 1.1: Household identifiers				
100h	**Date** (dd/mm/yyyy)	_ _ / _ _ / _ _		
101h	Region	________________		
102h	Zone	________________		
103h	Woreda	_________________		
105h	Kebele	__________________		
106h	Gote	__________________		
107h	Cluster Code	_____		
108h	Household Code	__ __		
109h	Unique household ID<br>(cluster / household code)	__ __ __ / __ __		
110h	GPS Latitude	____ DEG ____ MIN ____ SEC		
111h	GPS Longitude	____ DEG ____ MIN ____ SEC		
113h	Elevation	__ __ __ __		
114h	Interviewer initials	__ __		
115h	***Interviewer***: Have you read him/her the consent form?	1=Yes<br>2=No-one is available		If the answer is No, fill the revisit form
116h	***Interviewer***: Does the respondent agree?	1=Yes<br>2=No. END INTERVIEW		If No Why? State the reason.
112h	Name of household head	______________________		
117h	What is the ethnic group of the household head?	01=Agew<br>02=Amhara<br>03=Konso<br>04=Burji<br>05=Kore<br>06=Gedeo<br>08=Derashe<br>09=Dawuro	11=Konta<br>12=Ale<br>13=Oromo<br>15=Tigray<br>17=Other, Specify	_____

For peer review only

Household Listing with Household Head: Now I would like to ask you about people who are residents of this house. By resident, I mean everyone who has been present for the last 3 months and who normally eat together. *Start with head of the household and older people, then children from oldest to youngest.*

No. of Family members: ***Including yourself how many family members do you have?*** ___________

118h_1 Unique ID	118h_2 NAME	118h_3 RELATIONSHIP	118h_4 SEX	118h_5 AGE	118h_E Eligible	118h_6 DATE OF BIRTH	118h_7 MARITAL STATUS	118h_8 RELIGION	118h_9 EDUCATION	118h_10-118h_15 ELIGIBILITY		
		**What is the relationship of [Name] to the household head?**<br><br>01=Head<br>02=Wife or husband<br>03=Son or daughter<br>04=In-law<br>05=Grandchild<br>06=Parent<br>08=Brother or sister<br>09=Niece or nephew<br>10=Adopted/stepchild<br>11=Other relative<br>12=Not related<br>99=Don't know	**Is [Name] male or female?**<br><br>1=Male<br>2=Female	**How old is [Name]?**<br><br>If less than 1 year write in 0		**What is [Name] date of birth?**<br><br>dd/mm/yyyy	***For all born after 1966:*** **Is [name] married?**<br><br>1=Currently married<br>2= In a union<br>3=Not married or in a union<br>4=Divorced<br>5=Widowed<br>6=N/A	***For all:*** **What religion is [name]?**<br><br>1=Orthodox<br>3=Protestant<br>4=Muslim<br>8=Other	**How many years of formal education have they completed?**<br><br>*Enter number of formal years of education completed*	***CHECK: Is the person a woman 13-49 years?***<br>From 1969 to 2005 (Eth: 1961-1997)<br>1=Yes<br>2=No	***CHECK*****: Is the person a child under 5 years?**<br><br>1=Yes<br>2=No	***CHECK*****: If the household has a child under 5 years, is this person the primary caregiver?**<br><br>1=Yes<br>2=No
01	___	___	___	___		__ __/__ __/__ __	__	__	___	__	__	__
02	___	___	___	___		__ __/__ __/__ __	__	__	___	__	__	__
03	___	___	___	___		__ __/__ __/__ __	__	__	___	__	__	__
04	___	___	___	___		__ __/__ __/__ __	__	__	___	__	__	__
05	___	___	___	___		__ __/__ __/__ __	__	__	___	__	__	__
06	___	___	___	___		__ __/__ __/__ __	__	__	___	__	__	__
07	___	___	___	___		__ __/__ __/__ __	__	__	___	__	__	__
08	___	___	___	___		__ __/__ __/__ __	__	__	___	__	__	__
09	___	___	___	___		__ __/__ __/__ __	__	__	___	__	__	__

For peer review only

For peer review only - http://bmjopen.bmj.com/site/about/guidelines.xhtml

Section 1.2: Characteristics of house and assets			
119h	What is the main material of the roof? *Select one*	1 = Thatch/grass or leaves<br>2 = Iron sheets<br>8 = Other…specify	__
120h	What is the main material of the walls? *Select one*	2 = Natural materials cane, wood, mud<br>4 = Stone with mud<br>5 = Stone with cement/bricks<br>8 = Other	__
121h	What is the main floor material? *Select one*	1 = Natural floor (earth/sand/dung)<br>2 = Rudimentary floor (wood/palm/bamboo)<br>3 = Finished floor (polished wood, cement, carpet)<br>8 = Other	__
122h	What kind of toilet facilities does your household have?	1= No facility/bush/field<br>2= Pit toilet/latrine<br>3= Ventilated improved pit latrine<br>8=Other	__
123h	What is the main source of drinking water for members of your household?<br><br>*Do Not read list*	1=Piped water into dwelling<br>2=Piped water into compound<br>3=Public tap<br>4=Borehole in compound<br>6=Water from protected spring<br>8=Surface water (river/dam/lake etc)<br>9=Hand pump<br>10=Unprotected spring<br>88=Other	__
124h	Do you do anything to the water to make it safer to drink?	1 = Yes<br>2 = No (SKIP TO 126)<br>3 = Don't know (SKIP TO 126)	__
125h	If Yes, what is the main thing you do?<br><br>*Do Not read list*	1 = Let it stand and settle<br>2 = Strain through a cloth<br>3 = Use water filter (ceramic/sand/composite/etc)<br>4 = Boil<br>5 = Solar disinfection<br>6 = Add bleach/chlorine<br>8 = Other	__
126h	What type of fuel does your household mainly use for cooking? *Select one*	1=Electricity<br>4=Charcoal<br>5=Firewood/straw<br>6=Dung<br>8=Other	__
127h	Is the house connected to electricity?	1=Yes<br>2=No	__
**In total, how many the following items owned by residents of this household?** *Enter number of items (zero if None)*			
128h	How many **Wrist watch**?		____
129h	How many **Gold (in grams)?**		____
130h	How many **kerosene lamp/pressure lamps?**		____

For peer review only - http://bmjopen.bmj.com/site/about/guidelines.xhtml

131h	How many **Solar lamp?**		____
132h	How many **beds?**		____
134h	How many **Mobile phone?**		____
137h	How many **Radio?**		____
138h	How many **TV**?		____
142h	Do you own this house?	1 = Yes<br>2 = No	__
143h	Does any member of the household own any agricultural land?	1 = Yes<br>2 = No **SKIP TO 145**	
144h	How many hectares of agricultural land do members of this household own?	*Enter total number of hectares*<br>*(If less than 1, Enter in decimals (example 0.5)*<br>*Enter 9999 if hectares are Not known*	____.____
145h	Does this household own any livestock, herds, other farm animals, or poultry?	1 = Yes<br>2 = No **SKIP TO 155**	__
	For each: Enter number. If None, enter 000		
	**How many of the following animals does this household own?**		
146h	How many **chickens?**		________
147h	How many **goats?**		___
148h	How many **sheep?**		___
149h	How many **donkeys?**		___
150h	How many **horses?**		___
153h	How many **milk cows?**		___
154h	How many **ox (bulls)?**		___
**155h**	At any time in the past 12 months, has anyone come into your house to spray the interior walls of your dwelling against mosquitoes?	1=Yes<br>2=No **SKIP TO 158**<br>9=Don't know **SKIP TO 158**	__
156h	What type of residual spray was done? *(Probe if indoor residual spraying (IRS)*	1=IRS<br>8=Other<br>9=Don't know	__
157h	How many months ago was the house last sprayed?	If less than one month write 00. If don't know write 99	__
158h	How many mosquito nets does your household have?	Write total number Count those in use plus those Not in use. If don't know write 9 If "0" SKIP TO MODULE 2	__

For peer review only - http://bmjopen.bmj.com/site/about/guidelines.xhtml

Section 1.3: Household protection from malaria

Write total number of count those in use plus those Not in use (If don't know write 9)

			Net #1	Net #2	Net #3
159h	*Interviewer*: Is the Net 1 observed?	1=Yes<br>2=No	___	___	___
160h	How many months ago did your household obtain the mosquito Net1?	Estimate the number of months ago each net was obtained. If don't know 99	___	___	___
161h	Where did your household get the mosquito Net1 from?	1=Gov. hospital or health centre<br>2=Private hospital or health centre<br>3=HEW/HDA<br>5=Bought from shop/open market<br>7=Mass campaign<br>8=Other<br>9=Don't know	___	___	___
162h	What kind of Net 1 is it?	1= Long-lasting net **SKIP TO 166**<br>2=Factory net<br>8=Other<br>9=Don't know	___	___	___
163h	When you got Net 1 was it already treated with an insecticide to kill or repel mosquitos?	1=Yes<br>2=No<br>9=Don't know	___	___	___
164h	Did anyone sleep under the mosquito Net 1 last night?	1=Yes<br>2=No **If 2 or 9 then, SKIP TO 166**<br>9=Don't know	___	___	___
165h	Who slept under any of the nets last night?	*(record the Person ID from household listing)*	*Enter Person ID*<br>____<br>____<br>____	____<br>____<br>____	____<br>____<br>____
159h 2	***Interviewer***: Is the Net 2observed?	1=Yes<br>2=No	___	___	___
160h 2	How many months ago did your household obtain the mosquito Net2	*Estimate the number of months ago each net was obtained. If don't know 99*	____	____	____
161h 2	Where did your household get the mosquito Net2 from?	1=Gov. hospital or health centre<br>2=Private hospital or health centre<br>3=HEW/HDA<br>5=Bought from shop/open market<br>7=Mass campaign<br>8=Other<br>9=Don't know	___	___	___
162 h2	What kind of Net 2 is it?	1= Long-lasting net **SKIP TO 166**<br>2=Factory net<br>8=Other<br>9=Don't know	___	___	___
163h 2	When you got Net 2 was it already treated with an insecticide to kill or repel mosquitos?	1=Yes<br>2=No<br>9=Don't know	___	___	___
164h 2	Did anyone sleep	1=Yes	___	___	___

For peer review only - http://bmjopen.bmj.com/site/about/guidelines.xhtml

	under the mosquito Net 2 last night?	2=No **If 2 or 9 then, SKIP TO 166**<br>9=Don't know			
165h 2	Who slept under any of nets	*(Record the Person ID from household listing)*	*Enter Person ID*<br>____<br>____<br>____	____<br>____<br>____	____<br>____<br>____
159h 3	***Interviewer***: Is the Net 3 observed	1=Yes<br>2=No	__	__	__
160h 3	How many months ago did your household obtain the mosquito Net3?	Estimate the number of months ago each net was obtained. If don't know 99	__	__	__
161h 3	Where did your household get the mosquito Net3 from?	1=Gov. hospital or health centre<br>2=Private hospital or health centre<br>3=HEW/HDA<br>5=Bought from shop/open market<br>7=Mass campaign<br>8=Other<br>9=Don't know	__	__	__
162h 3	What kind of Net 3 is it?	1= Long-lasting net (**SKIP TO 166)**<br>2=Factory net<br>8=Other<br>9=Don't know	__	__	__
163h 3	When you got Net 3 was it already treated with an insecticide to kill or repel mosquitos?	1=Yes<br>2=No<br>9=Don't know	__	__	__
164h 3	Did anyone sleep under the mosquito Net 3 last night?	1=Yes<br>2=No **If 2 or 9 then, SKIP TO 166**<br>9=Don't know	__	__	__
165h 3	Who slept under this mosquito Net 3 last night	*(record the Person ID from household listing)*	*Enter Person ID*<br>____<br>____<br>____	____<br>____<br>____	____<br>____<br>____

For peer review only

Module 2 – Woman aged 13-49 years

**Section 2.1: Identification and consent of eligible women**			
166h	*Name of women*		________________
167h	*Woman ID*		____
168h	Is it possible to interview the woman?	1=Yes **SKIP TO 170**<br>2=No	__
169h	If No, why Not possible to interview the woman?	1=Travelled away<br>2=Sick<br>3=Other<br>4=Currently Not present	__
170h	*Interviewer*: Have you read her the consent form?	1=Yes<br>2= No	__
171h	Does she give her consent to be interviewed?	1=Yes<br>2=No **SKIP TO 179**	__
Thank you for agreeing to participate in this interview. I would like to first ask you about your involvement in the women development army (WDA)			
172h	Do you participate in WDA?	1=Yes<br>2=No **SKIP TO 175**	__
173h	Are you a 1 to 5 WDA leader?	1=Yes<br>2=No	__
174h	Are you a 1 to 30 WDA team leader?	1=Yes<br>2=No	__

**Section 2.2: Pregnancy history**			
175h	Are you pregnant Now?	1=Yes<br>2=No<br>8=Unsure	__
176h	Have you ever been pregnant before?	1 = Yes<br>2 = No **(End Interview)**	__
177h	Have you ever given birth to a child?	1 = Yes<br>2 = No	__
178h	How many children have you given birth to in total?	Enter number	__
I understand that it is not easy to talk about children who have died, or pregnancies that did not end up not in live birth, but it is important that you tell us about all of them, so that the government can develop programs to improve children's health.			
179h	Have you ever had a pregnancy that's lost before full term?	1=Yes<br>2=No **SKIP TO 181**	__
180h	How many pregnancies have you had that did Not end in a live birth?	Enter number	__
Now would like to ask about the information regarding pregnancies beginning of 2009 (Ethiopian calendar).			
181h	Were you pregnant at the time of 2009 or any time afterwards?	1 = Yes<br>2 = No **(End interview)**	__

For peer review only - http://bmjopen.bmj.com/site/about/guidelines.xhtml

Now I would like to record your births since 2009 (Ethiopian Calendar), whether still alive or not, starting with THE MOST RECENT ONE. *Record twins and triplets on separate lines.*

No_of_Preg. How many times were you pregnant at the time of 2009 or any time afterwards?

Is your younger child 2 or under2 years of age?

167h **Woman ID**	181h_a **Pregnancy ID**	181h_b **Outcome of pregnancy**	181h_c **Baby's name**	181h_d **Date of birth/ Pregnancy ended**	181h_e **Born as a twin?**	181h_f **Sex**	181h_g **Still alive?**	181h_h **If still alive, how old in months?**	181h_i **If the child died, when did s/he die?**
		1=Live birth<br>2=Baby born dead, **Skip to 181**<br>3=Lost before full-term **Skip to 181**	*Enter "Not given" if Not given a name*	*Enter 01 for day if Not known. Probe for months/ years if Not known*	1 = Yes<br>2 = No<br>3=Don't know	1=Male<br>2=Female<br>3=Don't know	1=Yes<br>2=No	*If less than 28 days enter number of days*	*Enter 01 for day if Not known. Probe for months/years if Not known*
	1	__	__	DD/MM/ YYYY	__	__	__	DD/MM	DD/MM/ YYYY
	2	__	__	DD/MM/ YYYY	__	__	__	DD/MM	DD/MM/ YYYY
	3	__	__	DD/MM/ YYYY	__	__	__	DD/MM	DD/MM/ YYYY
	4	__	__	DD/MM/ YYYY	__	__	__	DD/MM	DD/MM/ YYYY
	5	__	__	DD/MM/ YYYY	__	__	__	DD/MM	DD/MM/ YYYY
	6	__	__	DD/MM/ YYYY	__	__	__	DD/MM	DD/MM/ YYYY

For peer review only

Section 2.3: Pregnancy care for last birth (in the last two years.			
Now I want to talk to you about the last birth you had that ended in [DATE}, with the birth of [NAME}			
182h	Do you have a family health card with information about that pregnancy and birth?	1=Yes<br>2=No **SKIP TO 184**	__
183h	May I see your family health card?	1=Yes<br>2=No because it isn't at home<br>3=No, refused permission	__
184h	When pregnant with [NAME], did you receive any care during pregnancy? *(Probe: by care I mean any care from HEW, HW, nurses, midwives or doctors, clinics)*	1=Yes<br>2=No **SKIP TO 236**	__
185h_a	Where did you go for pregnancy care?<br>**At home**	1=Yes<br>2=No	__
185h_b	Where did you go for pregnancy care?<br>**At Health Post**	1=Yes<br>2=No	__
185h_c	Where did you go for pregnancy care?<br>**At Health Centre**	1=Yes<br>2=No	__
185h_d	Where did you go for pregnancy care?<br>**At Hospital**	1=Yes<br>2=No	__
185h_e	Where did you go for pregnancy care?<br>**Other**	1=Yes<br>2=No	__
For women who received pregnancy care at a hospital, health center or health post			
186h	How many times did you attend the hospital for pregnancy (antenatal) care for that pregnancy?	Enter the number of times<br>If the answer is "0" Skip to 190	__
188h	How old was your pregnancy at the first visit?	*Record No. of weeks. If don't know 99. Record from FHC/ANC appointment card if available*	__
190h	How many times did you attend the health center for pregnancy (antenatal) care for that pregnancy?	Enter the number of times<br>If the answer is "0" Skip to 194	__
192h	How old was your pregnancy at the first visit?	*Record No. of weeks. If don't know 99. Record from FHC/ANC appointment card if available*	__
194h	How many times did you attend the health post for pregnancy (antenatal) care for that pregnancy?	Enter the number of times<br>If the answer is "0" Skip to 199	__
196h	How old was your pregnancy at the first visit?	*Record No. of weeks. If don't know 99. Record from FHC/ANC appointment card if available*	__
For women who receive pregnancy care at home			
199h	How many times <u>did the HEW visit you</u> at home for pregnancy care that pregnancy?	Enter number of times<br>If answer is "0" Skip to 201	__
200h	How old was your pregnancy at the first HEW visit?	Record No. of weeks. If don't know 99. Record from FHC/ANC appointment card if available	__
Now I want to ask you about the pregnancy care you received (Verify with family health card if available)			
201h	Was your weight measured?	1=Yes<br>2=No **SKIP TO 203**	__
202h	In which location was this service first provided?	1=Home by HEW<br>2=Health Post<br>3=Health Centre<br>4=Hospital<br>8=Other	__
203h	Was your height measured?	1=Yes<br>2=No **SKIP TO 205**	__
204h	In which location was this service first provided?	1=Home by HEW<br>2=Health Post<br>3=Health Centre	__

For peer review only - http://bmjopen.bmj.com/site/about/guidelines.xhtml

		4=Hospital<br>8=Other	__
205h	Did you receive information about breastfeeding?	1=Yes<br>2=No **SKIP TO 207**	__
206h	In which location was this service first provided?	1=Home by WDA<br>2=Home by HEW<br>3=Health post<br>4=Health center<br>5=Hospital<br>8=Other	__
207h	Was your blood pressure measured?<br>*(Probe: when a strap was put around your upper arm and a measure taken).*	1=Yes<br>2=No **SKIP TO 209**	__
208h	In which location was this service first provided?	1=Home by HEW<br>2=Health post<br>3=Health center<br>4=hospital<br>8=other	__
209h	Did you give a urine sample for a test?	1=Yes<br>2=No **SKIP TO 211**	__
210h	In which location was this service provided?	1=Home by HEW<br>2=Health post<br>3=Health center<br>4=Hospital<br>8=other	__
211h	Did you give blood for any test for syphilis?	1=Yes<br>2=No **SKIP TO 213**	__
212h	In which location was this service provided?	1=Home by HEW<br>2=Health Post<br>3=Health Centre<br>4=Hospital<br>8=Other	__
213h	Did you receive iron folate tablets or iron syrup?	1=Yes<br>2=No **SKIP TO 216**	__
214h	In which location was this service provided?	1=Home by HEW<br>2=Health post<br>3=Health Centre<br>4=Hospital<br>8=other	__
215h	For how many days did you take the tablets or syrup?	Enter number of days<br>Enter 999 if Not known	__
216h	Were you given an injection in the arm to prevent the baby from getting tetanus, that is, convulsions after birth?	1=Yes<br>2=No **SKIP TO 222**	__
217h	If Yes: How many times did you get a tetanus injection?	Write number of times	__
218h	If less than 2 times: **At any time before this pregnancy did you receive any tetanus injections?**	1=Yes<br>2=No **SKIP TO 221**	__
221h	In which health facility was tetanus injection given?	1=Home by HEW<br>2=Health post<br>3=Health Centre<br>4=Hospital<br>8=Other	__
222h	Did you receive HIV information?	1=Yes<br>2=No **SKIP TO 224**	__

For peer review only

For peer review only - http://bmjopen.bmj.com/site/about/guidelines.xhtml

223h	In which location was this service first provided?	1=Home by WDA<br>2=Home by HEW<br>3=Health post<br>4=Health Centre<br>5=Hospital<br>8=other	__
224h	Did you receive STI testing?	1=Yes<br>2=No **SKIP TO 226**	__
225h	In which location was this service provided?	1=Health Centre<br>2=Hospital<br>8=Other	__
226h	Did you receive any STI treatment?	1=Yes<br>2=No **SKIP TO 228**	__
227h	In which location was this service provided?	1=Health center<br>2=Hospital<br>8=Other	__
228h	Did you receive information on nutrition?	1=Yes<br>2=No **SKIP TO 230**	__
229h	In which location was this service first provided?	1=Home by WDA<br>2=Home by HEW<br>3=Health post<br>4=Health Centre<br>5=Hospital<br>8=Other	__
230h	Did you receive information on pregnancy danger signs?	1=Yes<br>2=No **SKIP TO 232**	__
231h	In which location was this service first provided?	1=Home by WDA<br>2=Home by HEW<br>3=Health post<br>4=Health Centre<br>5=Hospital<br>8=Other	__
232h	Were you advised on birth preparedness and complication readiness? *Probe: for finances, for help during delivery, for transport, for emergencies?*	1=Yes<br>2=No **SKIP TO 236**	__
233h	In which location was this service first provided?	1=Home by WDA<br>2=Home by HEW<br>3=Health post<br>4=Health Centre<br>5=Hospital<br>8=Other	__
234h	**Was your birth preparedness and complication readiness plan recorded?**	1=Yes<br>2=No **SKIP TO 236**	__
235h	**In which location was this service provided?**	1=Home by WDA<br>2=Home by HEW<br>3=Health post<br>4=Health Centre<br>5=Hospital<br>8=Other	__
Do Not read out list, prompt, 'anything else' record 1=Yes for all that apply			
236h	Can you tell me what are problems in pregnancy might need medical treatment? **Severe headache**	1=Yes<br>2= No	__
237h	Can you tell me what are problems in pregnancy might need	1=Yes	__

For peer review only - http://bmjopen.bmj.com/site/about/guidelines.xhtml

	medical treatment? **Blurry vision**	2= No	
238h	Can you tell me what are problems in pregnancy might need medical treatment?<br>**Reduced or absent fetal movement**	1=Yes<br>2= No	__
239h	Can you tell me what are problems in pregnancy might need medical treatment? **High blood pressure**	1=Yes<br>2= No	__
240h	Can you tell me what are problems in pregnancy might need medical treatment?<br>**Edema of the face/hands (i.e. swelling)**	1=Yes<br>2= No	__
241h	Can you tell me what are problems in pregnancy might need medical treatment? **Convulsions**	1=Yes<br>2= No	__
242h	Can you tell me what are problems in pregnancy might need medical treatment?<br>**Excessive vaginal bleeding**	1=Yes<br>2= No	__
243h	Can you tell me what are problems in pregnancy might need medical treatment?<br>**Severe lower abdominal pain**	1=Yes<br>2= No	__
244h	Can you tell me what are problems in pregnancy might need medical treatment? **Fever**	1=Yes<br>2= No	__
245h	Can you tell me what are problems in pregnancy might need medical treatment? **Anemia**	1=Yes<br>2= No	__
246h	Can you tell me what are problems in pregnancy might need medical treatment? **Other**	Specify	__
247h	Were you told where to go if you had any complications?	1=Yes<br>2= No **SKIP TO 249**	__
248h	If Yes, where were you told to go?	1= Health post<br>2= Health Centre<br>3= Hospital<br>8=Other (specify)<br>9=Don't know	__
249h	During your last pregnancy did you make any preparations for your delivery? *Probe: for finances, for help during delivery, transport, emergencies?*	1=Yes<br>2= No **SKIP TO 260**	__
Do Not read out list, prompt, 'anything else' record 1=Yes for all that apply			
250h	What preparations did you make for the delivery?<br>**Financial**	1=Yes<br>2= No	__
251h	What preparations did you make for the delivery?<br>**Transport**	1=Yes<br>2= No	__
252h	What preparations did you make for the delivery?<br>**Food**	1=Yes<br>2= No	__
253h	What preparations did you make for the delivery?<br>**Identification of birth attendant**	1=Yes<br>2= No	__
254h	What preparations did you make for the delivery?<br>**Identification of facility**	1=Yes<br>2= No	__
255h	What preparations did you make for the delivery?<br>**Clean clothes**	1=Yes<br>2= No	__
256h	What preparations did you make for the delivery?	1=Yes<br>2= No	__

For peer review only

For peer review only - http://bmjopen.bmj.com/site/about/guidelines.xhtml

	**Cover to deliver on**		
257h	What preparations did you make for the delivery?<br>**Gloves**	1=Yes<br>2= No	__
258h	What preparations did you make for the delivery?<br>**Cotton gauze**	1=Yes<br>2= No	__
259h	What preparations did you make for the delivery?<br>**Other**	Specify	__

Section 2.4 Delivery for last birth

260h	Who assisted with the delivery?	1=Doctor<br>2=Nurse/Midwife<br>3=Health Extension Worker<br>4=Traditional Birth Attendant<br>5=Relative/friend<br>6=No-one **SKIP TO 263**<br>8= Other (specify)	__
261h	When you gave birth, did the person assisting you wear gloves during delivery?	1=Yes<br>2= No<br>9=Don't know	__
262h	When you gave birth, did the person assisting you wash her hands before the delivery?	1=Yes<br>2= No<br>9=Don't know	__
263h	Where did you give birth?	1=Home **SKIP TO 266**<br>2=Health post<br>3=Health Centre<br>4=Hospital<br>8=Other	__
264h	If health post/health centre/hospital: After giving birth, for how many days did you stay at the health post/health Centre/hospital in total?	Enter number of days, enter 0 if she left on the same day as delivery	__
265h	Was [NAME] delivered by caesarean, that is, did they cut your belly open to take the baby out?	1=Yes<br>2= No	__
	Read out the list		
266h	During the delivery of [NAME] did you experience any of the following? **Heavy bleeding**	1=Yes<br>2= No	__
267h	During the delivery of [NAME] did you experience any of the following? **Labour more than 12 hours**	1=Yes<br>2= No	__
268h	During the delivery of [NAME] did you experience any of the following? **Loss of consciousness**	1=Yes<br>2= No	__
269h	During the delivery of [NAME] did you experience any of the following? **Premature labour**	1=Yes<br>2= No	__
270h	During the delivery of [NAME] did you experience any of the following? **Foul discharge**	1=Yes<br>2= No	__
271h	During the delivery of [NAME] did you experience any of the following? **Baby in abnormal position**	1=Yes<br>2= No	__
272h	During delivery were you advised to go to a different facility for specialist care (if home birth to a health post/health center/hospital, if health post/facility to a hospital/different	1=Yes<br>2= No **SKIP TO 275**	__

For peer review only

	facility)?		
273h	If Yes: Did you go to that different facility to get the specialist care (referral)?	1=Yes<br>2= No **SKIP TO 275**	__
274h	If Yes: What transport did you take to get there?	1=Own transport<br>2=Public transport<br>3=Hired transport<br>4=District/ambulance<br>5=Traditional ambulance (Qareza/cart)<br>6=Motor Bike/Bajaj<br>7=Walked<br>8=Other (specify)	__
275h	Did anyone check on your health after the delivery?	1=Yes<br>2= No **SKIP TO 288**	__
276h	How many times did anyone check on your health in the first month after delivery?	Write number. If don't know 99.	__
277h	How long after delivery did the first check take place?	Record number of days if same day as delivery enter 0. If don't know 99.	__
278h	Who checked on your health for the first time after you gave birth to [NAME]? *(Probe for most qualified person)*	1=Doctor/Health Officer<br>2=Nurse/Midwife/Health Officer<br>3=Health Extension Worker<br>4=Traditional birth attendant<br>8=Other (specify)	__
279h	Where did this check take place?	1=Home<br>2=Health post<br>3=Health center<br>4=Hospital<br>8=Other (specify)	__
280h	*If her health was checked at least twice (see Q above):* How long after delivery did the second check take place?	Record number of days. If don't know 99.	__
Do Not read out list, prompt, 'anything else' record 1=Yes for all that apply			
281h	During any of the health checks what was done to check on your health? **Examined body**	1=Yes<br>2= No	__
282h	During any of the health checks what was done to check on your health? **Checked breasts**	1=Yes<br>2= No	__
283h	During any of the health checks what was done to check on your health? **Checked for heavy bleeding**	1=Yes<br>2= No	__
284h	During any of the health checks what was done to check on your health? **Counselled on danger signs**	1=Yes<br>2= No	__
285h	During any of the health checks what was done to check on your health? **Counselled on family planning**	1=Yes<br>2= No	__
286h	During any of the health checks what was done to check on your health? **Counselled on nutrition**	1=Yes<br>2= No	__
287h	During any of the health checks what was done to check on your health? **Referred to a health facility**	1=Yes<br>2= No	__
287h_n	During any of the health checks what was done to check on your health? **None from the list**	1=Yes<br>2= No	__

For peer review only - http://bmjopen.bmj.com/site/about/guidelines.xhtml

Section 2.5: Newborn Care			
Now I have some questions about what happened to [NAME] at the birth and immediately after.			
288h	Can I see a card recording information about the birth? (*use the card to verify all information if possible*)	1=Yes<br>2=No	__
289h	Was [NAME] weighed at birth?	1=Yes<br>2=No **SKIP TO 291**	__
290h	If Yes, how much did [NAME] weigh at birth? (*use the weight recorded on the card if possible)*	*Weight in grams e.g. if the weight was 1.9 kilograms enter 1900, If don't know 9999.*	__
291h	Did [NAME] have any difficulty breathing/crying at birth?	1=Yes<br>2=No **SKIP TO 293**	
292h_a	Did anyone do **Rubbing** to [NAME] immediately after birth?	1= Yes<br>2= No<br>9 = Don't know	__
292h_b	Did anyone do **Stimulating** to [NAME] immediately after birth?	1= Yes<br>2= No<br>9 = Don't know	__
292h_c	Did anyone do **Mouth-to-mouth** to [NAME] immediately after birth?	1= Yes<br>2= No<br>9= Don't know	__
292h_d	Did anyone do **Resuscitation** to [NAME] immediately after birth?	1= Yes<br>2= No<br>99 = Don't know	__
292h_e	Did anyone do **None of these** to [NAME] immediately at birth?	1= Yes<br>2= No<br>9= Don't know	__
293h	Where was [NAME] placed immediately after delivery?	1=Alone/on the floor<br>2=On the mother's belly/chest<br>3=Beside the mother<br>4=With someone else<br>8=Other (specify)<br>9= Don't know	__
294h	When [NAME] was born, was she/he dried/wiped?	1=Yes<br>2=No **If 2 or 9 Skip to 296**<br>9=Don't know	__
295h	How long after [NAME] was born was she/he dried/wiped?	*Enter in minutes, check for time after the baby was born (Not time after the placenta came out). If don't know 99*	__
296h	When [NAME] was born, was she/he wrapped with a cloth?	1=Yes<br>2= No **If 2 or 9 SKIP TO 298**<br>9=Don't know	__
297h	How long after [NAME] was born was she/he wrapped with a cloth?	*Enter in minutes, check for time after the baby was born (Not time after the placenta came out). If don't know 99*	__
298h	What was used to tie the cord?	1=New string/thread<br>2=Boiled string/thread<br>3=Any string/thread<br>4=Nothing<br>8=Other (specify)<br>9=Don't know	__
299h	What was used to cut the cord?	1=New razor blade<br>2=Any razor blade<br>3=Scissors<br>8=Other (specify)<br>9=Don't know	__
300h	Was anything applied to the cord after cutting and tying?	1=Yes<br>2= No **SKIP TO 308**	__

For peer review only - http://bmjopen.bmj.com/site/about/guidelines.xhtml

		9=Don't know **SKIP TO 308**	
Do Not read out list, prompt, 'anything else' record 1=Yes for all that apply			
301h	What was applied to the cord just after cutting the cord? **Butter**	1= Yes<br>2= No	__
302h	What was applied to the cord just after cutting the cord? **Ash**	1= Yes<br>2= No	__
303h	What was applied to the cord just after cutting the cord? **Ointment**	1= Yes<br>2= No	__
304h	What was applied to the cord just after cutting the cord? **Animal dung**	1= Yes<br>2= No	__
305h	What was applied to the cord just after cutting the cord? **Oil**	2= No<br>1= Yes	__
306h	What was applied to the cord just after cutting the cord? **Cold water**	1= Yes<br>2= No	__
307h	What was applied to the cord just after cutting the cord? **Other**	Specify________	__
308h	When [NAME] was born, how soon was [NAME] bathed?	1=In the first hour<br>2=After one-hour **SKIP TO 310**<br>3=After one day **SKIP TO 311**	__
309h	If in the first hour: After how many minutes would you say?	Write number of minutes.<br>**SKIP TO 310**	__
310h	If after one hour: After how many hours would you say?	*Write number of hours. If response is 'after one hour' enter 1. If response is 'after one and a half hours' enter 1.*<br>**SKIP TO 312**	__
311h	If after one day: After how many days would you say?	*Write number of days. If response is 'after one day' enter 1. If response is 'after one and a half days' enter 1.*	__
312h	In the first week of life, did you hold [NAME] skin to skin against your breasts during the daytime and night time?	1=Yes always<br>2=Yes very often<br>3=Yes sometime<br>4=Never<br>9=Don't know	__
313h	In the first week of life, did you sleep with [NAME] against you at night, or did you lay him/her alone on the bed or elsewhere?	1=Slept with mother<br>2=Baby slept alone<br>3=Baby slept with another person	__
314h	Did you ever breastfeed [NAME]?	1=Yes<br>2= No **SKIP TO 319**	__
315h	How long after birth did you first put [NAME] to the breast?	1=In the first hour<br>2=After one-hour **SKIP TO 317**<br>3=After one day **SKIP TO 318**	__
316h	If in the first hour: After how many minutes would you say?	Write number of minutes.<br>**SKIP TO 319**	__
317h	If after one hour: After how many hours would you say?	*Write number of hours. If response is 'after one hour' enter 1. If response is 'after one and a half hours' enter 1.*<br>**SKIP TO 319**	__
318h	How soon after the baby born did you bath him/ her?	*Write number of days. If response is 'after one day' enter 1. If response is 'after one and a half days' enter 1.*	__
319h	Did [NAME] receive vitamin K injection after delivery?	1 = Yes<br>2 = No<br>9= Don't know	__

For peer review only - http://bmjopen.bmj.com/site/about/guidelines.xhtml

320h	Did [NAME] receive TTC eye ointment after delivery?	1 = Yes<br>2 = No<br>9= Don't know	__
321h	Did [NAME] receive oral polio vaccine-after delivery?	1 = Yes<br>2 = No<br>9= Don't know	__
322h	Did you squeeze out and throw away the first milk?	1=Yes<br>2= No	__
323h	In the first three days after delivery, was [NAME] given anything to drink other than breast milk?	1=Yes<br>2= No	__
Now about care for [name] in the first month of his/her life			
324h	In the month after [NAME] was born, did any health care provider or a traditional birth attendant check on his/her health? *Probe for checks done at the place of birth on the same day as birth, and checks after*	1=Yes<br>2= No **SKIP TO 340**	__
325h	If Yes: In the month after [NAME] was born, how many times did a health care provider or a traditional birth attendant check on his/her health?	Write number of times.	__
326h	If Yes: How long after delivery did the first check take place?	*Record number of days if same day as delivery enter 0*	__
327h	If more than one time: How long after delivery did the second check take place?	*Record number of days*	__
328h	Who checked on [NAME] health the first time? (Probe for most qualified person)	1=Doctor/Health Officer<br>2=Nurse/Midwife<br>3=Health Extension Worker<br>4=Traditional birth attendant<br>8=Other (specify)	__
329h	Where did the first check on [NAME] take place?	1=Home<br>2=Health post<br>3=Health centre<br>4=Hospital<br>8=Other (specify)	__
330h	At any of the health checks in the first month, what was done to check the health of baby? **Generally examined/ looked at baby's body**	1=Yes<br>2= No	__
331h	At any of the health checks in the first month, what was done to check the health of baby? **Weighed baby**	1=Yes<br>2= No	__
332h	At any of the health checks in the first month, what was done to check the health of baby? **Checked cord**	1=Yes<br>2= No	__
333h	At any of the health checks in the first month, what was done to check the health of baby? **Counselled on breastfeeding**	1=Yes<br>2= No	__
334h	At any of the health checks in the first month, what was done to check the health of baby? **Observed breastfeeding**	1=Yes<br>2= No	__
335h	At any of the health checks in the first month, what was done to check the health of baby? **Counselled on skin-to-skin contact/warmth**	1=Yes<br>2= No	__
336h	At any of the health checks in the first month, what was done to check the health of baby?	1=Yes<br>2= No	__

For peer review only - http://bmjopen.bmj.com/site/about/guidelines.xhtml

	**Checked baby for danger signs**		
337h	At any of the health checks in the first month, what was done to check the health of baby? **Counselled on danger signs**	1=Yes<br>2= No	__
338h	At any of the health checks in the first month, what was done to check the health of baby? **Referred to a health facility**	1=Yes<br>2= No	__
339h	At any of the health checks in the first month, what was done to check the health of baby? **Nothing**	1=Yes<br>2= No	__
	Now I want to talk to you about any sickness your child experienced in the ***first month of life.***		
341h	Was [NAME] sick in the first month?	1=Yes<br>2= No **SKIP TO 396**	__
342h	Can I just check, did [NAME] have any of the following symptoms? **Stopped feeding well**	1=Yes<br>2= No	__
343h	Can I just check, did [NAME] have any of the following symptoms? **Difficult or fast breathing**	1=Yes<br>2= No	__
344h	Can I just check, did [NAME] have any of the following symptoms? **Chest in-drawing**	1=Yes<br>2= No	__
345h	Can I just check, did [NAME] have any of the following symptoms? **Unusually hot or cold**	1=Yes<br>2= No	__
346h	Can I just check, did [NAME] have any of the following symptoms? **Baby less active than usual**	1=Yes<br>2= No	__
347h	Can I just check, did [NAME] have any of the following symptoms? **Body became yellow**	1=Yes<br>2= No	__
348h	Can I just check, did [NAME] have any of the following symptoms? **Other**	**Specify**	__
349h	If any sickness/symptom reported: How old was [NAME] when sick for the first time?	*Record number of days of age when [NAME] was first sick if on first day of life enter 0*	__
	Check all the following symptoms: (Read all)		
350h	When [NAME] was sick that first time what was the problem? **Fever**	1=Yes<br>2= No	__
351h	When [NAME] was sick that first time what was the problem? **Unable to suckle/feed**	1=Yes<br>2= No	__
352h	When [NAME] was sick that first time what was the problem? **Difficult/fast breathing**	1=Yes<br>2= No	__
353h	When [NAME] was sick that first time what was the problem? **Severe chest in-drawing**	1=Yes<br>2= No	__
354h	When [NAME] was sick that first time what was the problem? **Diarrhea**	1=Yes<br>2= No	__
355h	When [NAME] was sick that first time what was the problem? **Convulsions**	1=Yes<br>2= No	__
356h	When [NAME] was sick that first time what was the problem? **Persistent vomiting**	1=Yes<br>2= No	__
357h	When [NAME] was sick that first time what was the problem? **Yellow palms/soles/eyes**	1=Yes<br>2= No	__

For peer review only - http://bmjopen.bmj.com/site/about/guidelines.xhtml

358h	When [NAME] was sick that first time what was the problem? **Lethargy**	1=Yes<br>2= No	__
359h	When [NAME] was sick that first time what was the problem? **Unconscious**	1=Yes<br>2= No	__
360h	When [NAME] was sick that first time what was the problem? **Red/discharge eyes**	*1=Yes*<br>*2=No*	__
361h	When [NAME] was sick that first time what was the problem? **Skin pustules**	*1=Yes*<br>*2=No*	__
362h	When [NAME] was sick that first time what was the problem? **Skin around cord was red**	*1=Yes*<br>*2=No*	__
363h	When [NAME] was sick that first time what was the problem? **Pus from cord**	*1=Yes*<br>*2=No*	__
364h	When [NAME] was sick that first time what was the problem? **Other**	Specify	______
Select all that apply for the time before the baby was taken for extra care (do Not read out list, prompt, 'anything else' record 1=Yes for all that apply)			
365h	How was [NAME] treated for this illness at home? **Giving drugs**	*1=Yes*<br>*2=No*	__
366h	How was [NAME] treated for this illness at home? **Giving herbs**	*1=Yes*<br>*2=No*	__
367h	How was [NAME] treated for this illness at home? **Contacting a provider for advice**	*1=Yes*<br>*2=No*	__
368h	How was [NAME] treated for this illness at home? **Contacting someone else for advice**	*1=Yes*<br>*2=No*	__
369h	How was [NAME] treated for this illness at home? **No treatment given at home**	*1=Yes*<br>*2=No*	__
370h	How was [NAME] treated for this illness at home? **Don't remember**	*1=Yes*<br>*2=No*	__
371h	Did you seek care for [NAME] outside the home at that time?	1=Yes **SKIP TO 373**<br>2= No	__
372h_a	If No care sought at that time: Why didn't you seek care for [NAME] outside the home during that first illness? **Expected him/her to get better**	*1=Yes*<br>*2=No*	__
372h_b	If No care sought at that time: Why didn't you seek care for [NAME] outside the home during that first illness? **Health facility too far**	*1=Yes*<br>*2=No*	__
372h_c	If No care sought at that time: Why didn't you seek care for [NAME] outside the home during that first illness? **Cost of treatment too expensive**	*1=Yes*<br>*2=No*	__
372h_d	If No care sought at that time: Why didn't you seek care for [NAME] outside the home during that first illness? **Don't trust the facility**	*1=Yes*<br>*2=No*	__
372h_e	If No care sought at that time: Why didn't you seek care for [NAME] outside the home during that first illness? **Family member didn't allow**	*1=Yes*<br>*2=No*	__
372h_f	If No care sought at that time: Why didn't you seek care for [NAME] outside the home during that first illness? **Community advisor/TBA advised against it**	*1=Yes*<br>*2=No*	__
372h_g	If No care sought at that time: Why didn't you seek care for [NAME] outside the home during that first illness? **Other**	Specify	__

For peer review only - http://bmjopen.bmj.com/site/about/guidelines.xhtml

373h	If sought care: How many times did you seek care for that illness?	Write number of times	__
374h	Where outside the home did you seek care from the first time?	1=Health post<br>2=Health centre<br>3=Hospital<br>4=Pharmacy/shop<br>8=Other (specify)	__
375h	Do you have any medical record from when you went for health care outside the home the first time?	1=Yes<br>2= No **SKIP TO 377**	__
376h	If Yes, can I see it?	1=Yes<br>2= No	__
377h	After how many days did you seek care the first time?	*Write number of days from the onset of illness, if first day of illness write 0. If possible use the medical record to confirm*	__
378h	If Yes to any of the symptoms (342-348): At any time during the illness, did [NAME] take any drugs for the illness?	*If the information is available from the card use it*<br>1=Yes<br>2= No ***(END OF MODULE)***	__
Select all mentioned (do Not read out list, prompt, 'anything else' record 1=Yes for all that apply)			
379h	What drugs did [NAME] take? **Antimalarial tablet**	1=Yes<br>2= No	__
382h	What drugs did [NAME] take? **Antibiotic: Gentamicin injection**	1=Yes<br>2= No	__
383h	What drugs did [NAME] take? **Antibiotic: Amoxicillin syrup or tablet**	1=Yes<br>2= No	__
384h	What drugs did [NAME] take? **Antibiotic: Cotrimoxazole syrup**	1=Yes<br>2= No	__
385h	What drugs did [NAME] take? **Antibiotic: Other /Not known**	1=Yes<br>2= No	__
386h	What drugs did [NAME] take? **Tetracycline eye ointment**	1=Yes<br>2= No	__
387h	What drugs did [NAME] take? **Zinc**	1=Yes<br>2= No	__
388h	What drugs did [NAME] take? **ORS**	1=Yes<br>2= No	__
389h	What drugs did [NAME] take? **Combined Zinc-ORS**	1=Yes<br>2= No	__
390h	What drugs did [NAME] take? **Vitamin A**	1=Yes<br>2= No	__
391h	What drugs did [NAME] take? **Paracetamol**	1=Yes<br>2= No	__
392h	What drugs did [NAME] take? **Traditional remedies herbs/local remedy**	1=Yes<br>2= No	__
393h	What drugs did [NAME] take? **Other**	Specify	________
394h	If Yes to gentamicin injection: Did [NAME] receive **7 consecutive days of gentamycin injection?**	1=Yes<br>2= No<br>9 = Don't know	__
395h	If Yes to amoxicillin: Did [NAME] receive **7 consecutive days of amoxicillin?**	1=Yes<br>2= No<br>9= Don't know	__

End – thank the participant. Check to see if the participant needs to answer the child module. If so, continue to Module 3. Otherwise check whether there is another woman aged 13-49 in the house.

For peer review only

Module 3 – Child Health

Section 3.1: Identification of and consent from caregivers		
396h	***Interviewer***: How many children under 5 years of age live in this household? Check household listing.	____
397h	***Interviewer***: select name of caregiver from household listing	____
398h	***Interviewer***: select ID of the caregiver from household listing	____
399h	Is it possible to interview the caregiver?	1=Yes **SKIP TO 401**<br>2=No
400h	If No, why Not possible to interview the caregiver?	1=Travelled away<br>2=Sick<br>3=Currently Not present<br>8=Other reason
401h	***Interviewer***: Have you read her the consent form?	1=Yes<br>2= No-one is available (add reason)
402h	***Interviewer***: Does he/she give her consent to be interviewed??	1=Yes<br>2=No END INTERVIEW.

Section 3.2: Knowledge of malaria, diarrhea & pneumonia		
Do Not read out list, prompt, 'anything else' record 1=Yes for all that apply		
403h	Why do you sleep under a mosquito net?<br>**Protects from malaria**	1=Yes<br>2=No
404h	Why do you sleep under a mosquito net?<br>**Protects from mosquitoes**	1=Yes<br>2=No
405h	Why do you sleep under a mosquito net?<br>**Given free**	1=Yes<br>2=No
406h	Why do you sleep under a mosquito net?<br>**Bought cheaply**	1=Yes<br>2=No
407h	Why do you sleep under a mosquito net?<br>**Look attractive**	1=Yes<br>2=No
408h	Why do you sleep under a mosquito net? Health worker said to use	1=Yes<br>2=No
409h	Why do you sleep under a mosquito net?<br>**Someone told me to use it**	1=Yes<br>2=No
410h	Why do you sleep under a mosquito net?<br>**Neighbor uses it**	1=Yes<br>2=No
411h	Why do you sleep under a mosquito net?<br>**Other**	Specify
412h	Why do you sleep under a mosquito net?<br>**Don't know**	1=Yes<br>2=No

For peer review only - http://bmjopen.bmj.com/site/about/guidelines.xhtml

Do Not read out list, prompt, 'anything else' record 1=Yes for all that apply		
413h	If you don't want to get sick from malaria, how can you protect yourself? **Use a Bednet**	1=Yes<br>2=No
414h	If you don't want to get sick from malaria, how can you protect yourself? **Use repellent or spray**	1=Yes<br>2=No
415h	If you don't want to get sick from malaria, how can you protect yourself? **Avoid mosquitoes/bites**	1=Yes<br>2=No
416h	If you don't want to get sick from malaria, how can you protect yourself? **Take medication for**	1=Yes<br>2=No
417h	If you don't want to get sick from malaria, how can you protect yourself? **prevention Keep surroundings clean**	1=Yes<br>2=No
418h	If you don't want to get sick from malaria, how can you protect yourself? **Eat well**	1=Yes<br>2=No
419h	If you don't want to get sick from malaria, how can you protect yourself? **Drink boiled water**	1=Yes<br>2=No
420h	If you don't want to get sick from malaria, how can you protect yourself? **Avoid getting wet from rain**	1=Yes<br>2=No
421h	If you don't want to get sick from malaria, how can you protect yourself? **Other**	Specify
422h	If you don't want to get sick from malaria, how can you protect yourself? **Don't know**	1=Yes<br>2=No
**423h**	Have you seen or hear any messages on:<br>**Bed nets**	1=Yes<br>2=No
	Multiple options are possible. (do Not read out list, prompt, 'anything else' record 1=Yes for all that apply)	
423h_1	Where did you hear the message about **Bed nets** from?<br>**Friend**	1=Yes<br>2=No
423h_2	Where did you hear the message about **Bed nets** from?<br>**At Health Centre**	1=Yes<br>2=No
423h_3	Where did you hear the message about **Bed nets** from?<br>**HEW**	1=Yes<br>2=No
423h_4	Where did you hear the message about **Bed nets** from?<br>**WDA**	1=Yes<br>2=No
423h_5	Where did you hear the message about **Bed nets** from?<br>**Newspaper**	1=Yes<br>2=No
423h_6	Where did you hear the message about **Bed nets** from?<br>**TV**	1=Yes<br>2=No
423h_7	Where did you hear the message about **Bed nets** from?<br>**Radio**	1=Yes<br>2=No
423h_8	Where did you hear the message about **Bed nets** from?<br>**Government official**	1=Yes<br>2=No
423h_9	Where did you hear the message about **Bed nets** from?<br>**Church/Mosque**	1=Yes<br>2=No
423h_10	Where did you hear the message about **Bed nets** from?<br>**School**	1=Yes<br>2=No
423h_11	Where did you hear the message about **Bed nets** from?<br>**Poster/flier**	1=Yes<br>2=No

For peer review only - http://bmjopen.bmj.com/site/about/guidelines.xhtml

423h_12	Where did you hear the message about **Bed nets** from?<br>**Meeting**	1=Yes<br>2=No
423h_13	Where did you hear the message about **Bed nets** from?<br>**Street announcement**	1=Yes<br>2=No
423h_14	Where did you hear the message about **Bed nets** from?<br>**Other**	Specify
**424h**	Have you seen or hear any messages on:<br>**Malaria treatment using ACT**	1=Yes<br>2=No
Multiple options are possible. (do Not read out list, prompt, 'anything else' for all that apply)		
424h_1	Where did you hear the message about **ACT** from?<br>**Friend**	1=Yes<br>2=No
424h_2	Where did you hear the message about **ACT** from?<br>**At Health Centre**	1=Yes<br>2=No
424h_3	Where did you hear the message about **ACT** from?<br>**HEW**	1=Yes<br>2=No
424h_4	Where did you hear the message about **ACT** from?<br>**WDA**	1=Yes<br>2=No
424h_5	Where did you hear the message about **ACT** from?<br>**Newspaper**	1=Yes<br>2=No
424h_6	Where did you hear the message about **ACT** from?<br>**TV**	1=Yes<br>2=No
424h_7	Where did you hear the message about **ACT** from?<br>**Radio**	1=Yes<br>2=No
424h_8	Where did you hear the message about **ACT** from?<br>**Government official**	1=Yes<br>2=No
424h_9	Where did you hear the message about **ACT** from?<br>**Church/Mosque**	1=Yes<br>2=No
424h_10	Where did you hear the message about **ACT** from?<br>**School**	1=Yes<br>2=No
424h_11	Where did you hear the message about **ACT** from?<br>**Poster/flier**	1=Yes<br>2=No
424h_12	Where did you hear the message about **ACT** from?<br>**Meeting**	1=Yes<br>2=No
424h_13	Where did you hear the message about **ACT** from?<br>**Street announcement**	1=Yes<br>2=No
424h_14	Where did you hear the message about **ACT** from?<br>**Other**	Specify
**425h**	Have you seen or hear any messages on:<br>**Rapid diagnostic tests (RDT) for malaria**	1=Yes<br>2=No
Multiple options are possible. (do Not read out list, prompt, 'anything else' for all that apply)		
425h_1	Where did you hear the message about **RDT** from?<br>**Friend**	1=Yes<br>2=No
425h_2	Where did you hear the message about **RDT** from?<br>**At Health Centre**	1=Yes<br>2=No
425h_3	Where did you hear the message about **RDT** from?<br>**HEW**	1=Yes<br>2=No

For peer review only

425h_4	Where did you hear the message about **RDT** from? **WDA**	1=Yes<br>2=No
425h_5	Where did you hear the message about **RDT** from? **Newspaper**	1=Yes<br>2=No
425h_6	Where did you hear the message about **RDT** from? **TV**	1=Yes<br>2=No
425h_7	Where did you hear the message about **RDT** from? **Radio**	1=Yes<br>2=No
425h_8	Where did you hear the message about **RDT** from? **Government official**	1=Yes<br>2=No
425h_9	Where did you hear the message about **RDT** from? **Church/Mosque**	1=Yes<br>2=No
425h_10	Where did you hear the message about **RDT** from? **School**	1=Yes<br>2=No
425h_11	Where did you hear the message about **RDT** from? **Poster/flier**	1=Yes<br>2=No
425h_12	Where did you hear the message about **RDT** from? **Meeting**	1=Yes<br>2=No
425h_13	Where did you hear the message about **RDT** from? **Street announcement**	1=Yes<br>2=No
425h_14	Where did you hear the message about **RDT** from? **Other**	Specify
**426h**	Have you seen or hear any messages on: **Rectal artesunate**	1=Yes<br>2=No
Multiple options are possible. (do Not read out list, prompt, 'anything else' for all that apply)		
426h_1	Where did you hear the message about **Rectal artesunate** from? **Friend**	1=Yes<br>2=No
426h_2	Where did you hear the message about **Rectal artesunate** from? **At Health Centre**	1=Yes<br>2=No
426h_3	Where did you hear the message about **Rectal artesunate** from? **HEW**	1=Yes<br>2=No
426h_4	Where did you hear the message about **Rectal artesunate** from? **WDA**	1=Yes<br>2=No
426h_5	Where did you hear the message about **Rectal artesunate** from? **Newspaper**	1=Yes<br>2=No
426h_6	Where did you hear the message about **Rectal artesunate** from? **TV**	1=Yes<br>2=No
426h_7	Where did you hear the message about **Rectal artesunate** from? **Radio**	1=Yes<br>2=No
426h_8	Where did you hear the message about **Rectal artesunate** from? **Government official**	1=Yes<br>2=No
426h_9	Where did you hear the message about **Rectal artesunate** from? **Church/Mosque**	1=Yes<br>2=No
426h_10	Where did you hear the message about **Rectal artesunate** from? **School**	1=Yes<br>2=No
426h_11	Where did you hear the message about **Rectal artesunate** from? **Poster/flier**	1=Yes<br>2=No

For peer review only - http://bmjopen.bmj.com/site/about/guidelines.xhtml

426h_12	Where did you hear the message about **Rectal artesunate** from? **Meeting**	1=Yes<br>2=No
426h_13	Where did you hear the message about **Rectal artesunate** from? **Street announcement**	1=Yes<br>2=No
426h_14	Where did you hear the message about **Rectal artesunate** from? **Other**	Specify
**427h**	Have you seen or hear any messages on: **Diarrhea treatment**	1=Yes<br>2=No
Multiple options are possible. (do Not read out list, prompt, 'anything else' for all that apply)		
427h_1	Where did you hear the message about **Diarrhea treatment** from? **Friend**	1=Yes<br>2=No
427h_2	Where did you hear the message about **Diarrhea treatment** from? **At Health Centre**	1=Yes<br>2=No
427h_3	Where did you hear the message about **Diarrhea treatment** from? **HEW**	1=Yes<br>2=No
427h_4	Where did you hear the message about **Diarrhea treatment** from? **WDA**	1=Yes<br>2=No
427h_5	Where did you hear the message about **Diarrhea treatment** from? **Newspaper**	1=Yes<br>2=No
427h_6	Where did you hear the message about **Diarrhea treatment** from? **TV**	1=Yes<br>2=No
427h_7	Where did you hear the message about **Diarrhea treatment** from? **Radio**	1=Yes<br>2=No
427h_8	Where did you hear the message about **Diarrhea treatment** from? **Government official**	1=Yes<br>2=No
427h_9	Where did you hear the message about **Diarrhea treatment** from? **Church/Mosque**	1=Yes<br>2=No
427h_10	Where did you hear the message about **Diarrhea treatment** from? **School**	1=Yes<br>2=No
427h_11	Where did you hear the message about **Diarrhea treatment** from? **Poster/flier**	1=Yes<br>2=No
427h_12	Where did you hear the message about **Diarrhea treatment** from? **Meeting**	1=Yes<br>2=No
427h_13	Where did you hear the message about **Diarrhea treatment** from? **Street announcement**	1=Yes<br>2=No
427h_14	Where did you hear the message about **Diarrhea treatment** from? **Other**	Specify
**428h**	Have you seen or hear any messages on: **Pneumonia treatment**	1=Yes<br>2=No
Multiple options are possible. (do Not read out list, prompt, 'anything else' for all that apply)		
428h_1	Where did you hear the message about **Pneumonia treatment** from? **Friend**	1=Yes<br>2=No
428h_2	Where did you hear the message about **Pneumonia treatment** from? **At Health Centre**	1=Yes<br>2=No
428h_3	Where did you hear the message about **Pneumonia treatment** from? **HEW**	1=Yes<br>2=No

For peer review only

428h_4	Where did you hear the message about **Pneumonia treatment** from? **WDA**	1=Yes<br>2=No
428h_5	Where did you hear the message about **Pneumonia treatment** from? **Newspaper**	1=Yes<br>2=No
428h_6	Where did you hear the message about **Pneumonia treatment** from? **TV**	1=Yes<br>2=No
428h_7	Where did you hear the message about **Pneumonia treatment** from? **Radio**	1=Yes<br>2=No
428h_8	Where did you hear the message about **Pneumonia treatment** from? **Government official**	1=Yes<br>2=No
428h_9	Where did you hear the message about **Pneumonia treatment** from? **Church/Mosque**	1=Yes<br>2=No
428h_10	Where did you hear the message about **Pneumonia treatment** from? **School**	1=Yes<br>2=No
428h_11	Where did you hear the message about **Pneumonia treatment** from? **Poster/flier**	1=Yes<br>2=No
428h_12	Where did you hear the message about **Pneumonia treatment** from? **Meeting**	1=Yes<br>2=No
428h_13	Where did you hear the message about **Pneumonia treatment** from? **Street announcement**	1=Yes<br>2=No
428h_14	Where did you hear the message about **Pneumonia treatment** from? **Other**	Specify
**429h**	Have you seen or hear any messages on:<br>**Treatment for sick newborns**	1=Yes<br>2=No
429h_1	Where did you hear the message about **Pneumonia treatment** from? **Friend**	1=Yes<br>2=No
Multiple options are possible. (do Not read out list, prompt, 'anything else' for all that apply)		
429h_2	Where did you hear the message **Treatment for sick newborns** from? **At Health Centre**	1=Yes<br>2=No
429h_3	Where did you hear the message about **Treatment for sick newborns** from? **HEW**	1=Yes<br>2=No
429h_4	Where did you hear the message about **Treatment for sick newborns** from? **WDA**	1=Yes<br>2=No
429h_5	Where did you hear the message about **Treatment for sick newborns** from? **Newspaper**	1=Yes<br>2=No
429h_6	Where did you hear the message about **Treatment for sick newborns** from? **TV**	1=Yes<br>2=No
429h_7	Where did you hear the message about **Treatment for sick newborns** from? **Radio**	1=Yes<br>2=No
429h_8	Where did you hear the message about **Treatment for sick newborns** from? **Government official**	1=Yes<br>2=No
429h_9	Where did you hear the message about **Treatment for sick newborns** from? **Church/Mosque**	1=Yes<br>2=No
429h_10	Where did you hear the message about **Treatment for sick newborns** from? **School**	1=Yes<br>2=No
429h_11	Where did you hear the message about **Treatment for sick newborns** from? **Poster/flier**	1=Yes<br>2=No

For peer review only - http://bmjopen.bmj.com/site/about/guidelines.xhtml

429h_12	Where did you hear the message about **Treatment for sick newborns** from? **Meeting**	1=Yes<br>2=No
429h_13	Where did you hear the message about **Treatment for sick newborns** from? **Street announcement**	1=Yes<br>2=No
429h_14	Where did you hear the message about **Treatment for sick newborns** from? **Other**	Specify

---

Do Not read out list, prompt, 'anything else' record 1=Yes for all that apply)			
444h	If your child is sick with **a fever** what should you do? **Ignore it will go away**	1=Yes<br>2=No	__
445h	If your child is sick with **a fever** what should you do? **Go to health centre**	1=Yes<br>2=No	__
446h	If your child is sick with **a fever** what should you do? **Go to HEW**	1=Yes<br>2=No	__
447h	If your child is sick with **a fever** what should you do? **Go to WDA**	1=Yes<br>2=No	__
448h	If your child is sick with **a fever** what should you do? **Go to traditional healer**	1=Yes<br>2=No	__
449h	If your child is sick with **a fever** what should you do? **Ask to be tested for malaria**	1=Yes<br>2=No	__
450h	If your child is sick with **a fever** what should you do? **Treat with antimalarial / ACT**	1=Yes<br>2=No	__
451h	If your child is sick with **a fever** what should you do? **Treat with paracetamol**	1=Yes<br>2=No	__
452h	If your child is sick with **a fever** what should you do? **Treat with antibiotic**	1=Yes<br>2=No	__
453h	If your child is sick with **a fever** what should you do? **Treat with other medicine**	1=Yes<br>2=No	__
454h	If your child is sick with **a fever** what should you do? **Other**	Specify	__
Do Not read out list, prompt, 'anything else' record 1=Yes for all that apply)			
455h	If your child is sick with **a diarrhea** what should you do? **Ignore it will go away**	1=Yes<br>2=No	__
456h	If your child is sick with **a diarrhea** what should you do? **Go to health centre**	1=Yes<br>2=No	__
457h	If your child is sick with **a diarrhea** what should you do? **Go to HEW**	1=Yes<br>2=No	__
458h	If your child is sick with **a diarrhea** what should you do? **Go to WDA**	1=Yes<br>2=No	__
459h	If your child is sick with **a diarrhea** what should you do? **Go to traditional healer**	1=Yes<br>2=No	__
460h	If your child is sick with **a diarrhea** what should you do? **Fluid made from a special**	1=Yes<br>2=No	__

For peer review only - http://bmjopen.bmj.com/site/about/guidelines.xhtml

	**packet / ordinary rehydration salts (ORS)**		
461h	If your child is sick with **a diarrhea** what should you do? **A gov-recommended home-made fluid for diarrhoea**	1=Yes<br>2=No	__
462h	If your child is sick with **a diarrhea** what should you do? **Treat with paracetamol**	1=Yes<br>2=No	__
463h	If your child is sick with **a diarrhea** what should you do? **Treat with antibiotic**	1=Yes<br>2=No	__
464h	If your child is sick with **a diarrhea** what should you do? **Treat with other medicine**	1=Yes<br>2=No	__
465h	If your child is sick with a diarrhea what should you do? **Other**	Specify	__
Do Not read out list, prompt, 'anything else' record 1=Yes for all that apply)			
466h	If your child is sick with **a cough/ respiratory** infection what should you do? **Ignore it will go away**	1=Yes<br>2=No	__
467h	If your child is sick with **a cough/ respiratory infection** what should you do? **Go to health centre**	1=Yes<br>2=No	__
468h	If your child is sick with **a cough/ respiratory infection** what should you do? **Go to HEW**	1=Yes<br>2=No	__
469h	If your child is sick with **a cough/ respiratory infection** what should you do? **Go to WDA**	1=Yes<br>2=No	__
470h	If your child is sick with **a cough/ respiratory infection** what should you do? **Go to traditional healer**	1=Yes<br>2=No	__
471h	If your child is sick with **a cough/ respiratory infection** what should you do? **Treat with antibiotic**	1=Yes<br>2=No	__
472h	If your child is sick with **a cough/ respiratory infection** what should you do? **Treat with paracetamol**	1=Yes<br>2=No	__
473h	If your child is sick with **a cough/ respiratory infection** what should you do? **Treat with other medicine**	1=Yes<br>2=No	__
474h	If your child is sick with **a cough/ respiratory infection** what should you do? **Other action**	Specify	__
Do Not read out list, prompt, 'anything else' record 1=Yes for all that apply)			
475h	What are the signs of illness for a newborn baby? **Reduced feeding**	1=Yes<br>2=No	__
476h	What are the signs of illness for a newborn baby? **Difficult or fast breathing**	1=Yes<br>2=No	__
477h	What are the signs of illness for a newborn baby? **Movement only when stimulated or No movement even when stimulated**	1=Yes<br>2=No	__

For peer review only - http://bmjopen.bmj.com/site/about/guidelines.xhtml

478h	What are the signs of illness for a newborn baby? **Unusually hot or cold**	1=Yes<br>2=No	__
479h	What are the signs of illness for a newborn baby? **Convulsions**	1=Yes<br>2=No	__
480h	What are the signs of illness for a newborn baby? **Severe chest in-drawing**	1=Yes<br>2=No	__
481h	What are the signs of illness for a newborn baby? **Yellow palms/soles/eyes**	1=Yes<br>2=No	__
482h	What are the signs of illness for a newborn baby? **Diarrhea**	1=Yes<br>2=No	__
483h	What are the signs of illness for a newborn baby? **Skin pustules**	1=Yes<br>2=No	__
484h	What are the signs of illness for a newborn baby? **Cord red or draining puss**	1=Yes<br>2=No	__
485h	What are the signs of illness for a newborn baby? **Small infant (weight <2000 grams)**	1=Yes<br>2=No	__
Symptoms are: reduced feeding, difficult or fast breathing, Movement only when stimulated or No movement even when stimulated, unusually hot or cold, convulsions, severe chest in-drawing			
486h	If your newborn child **has symptoms of neonatal sepsis** what should you do?<br>**Ignore it will go away**	1=Yes<br>2=No	__
487h	If your newborn child has **symptoms of neonatal sepsis** what should you do?<br>**Go to health centre**	1=Yes<br>2=No	__
488h	If your newborn child has **symptoms of neonatal sepsis** what should you do?<br>**Go to HEW**	1=Yes<br>2=No	__
489h	If your newborn child has **symptoms of neonatal sepsis** what should you do?<br>**Go to WDA**	1=Yes<br>2=No	__
490h	If your newborn child has **symptoms of neonatal sepsis** what should you do?<br>**Go to traditional healer**	1=Yes<br>2=No	__
491h	If your newborn child has **symptoms of neonatal sepsis** what should you do?<br>**Treat with antibiotic**	1=Yes<br>2=No	__
492h	If your newborn child has **symptoms of neonatal sepsis** what should you do?<br>**Treat with paracetamol**	1=Yes<br>2=No	__
493h	If your newborn child has **symptoms of neonatal sepsis** what should you do?<br>**Treat with other medicine**	1=Yes<br>2=No	__
494h	If your newborn child has **symptoms of neonatal sepsis** what should you do?<br>**Other action**	Specify	__

For peer review only - http://bmjopen.bmj.com/site/about/guidelines.xhtml

Section 3.3: Health care available in kebele and recent interactions			
495h	Is there a health post in your kebele?	1= Yes<br>2=No **SKIP TO 506**	__
496h	How long does it take you to walk to the nearest health post?	*Record the distance in minutes.*<br>*If don't know, record 99*	__
497h	How many times have you visited the health post in the last six months?	*Record the number of times*<br>*If any visits last 6 months,*<br>***SKIP TO 504***	__
Do Not read out list, prompt, 'anything else' record 1=Yes for all that apply then SKIP TO 504			
498h	If never in the last 6 months: What are the reasons why you have Not visited the health post in the last six months? **No illness in the family/No births**	1=Yes<br>2=No	__
499h	If never in the last 6 months: What are the reasons why you have Not visited the health post in the last six months? **Health post is too far away**	1=Yes<br>2=No	__
500h	If never in the last 6 months: What are the reasons why you have Not visited the health post in the last six months? **health post Costs too much to go to**	1=Yes<br>2=No	__
501h	If never in the last 6 months: What are the reasons why you have Not visited the health post in the last six months? **Not enough time to visit**	1=Yes<br>2=No	__
502h	If never in the last 6 months: What are the reasons why you have Not visited the health post in the last six months? **Poor services available at the health post**	1=Yes<br>2=No	__
503h	If never in the last 6 months: What are the reasons why you have Not visited the health post in the last six months? **Other**	Specify	________________
504h	When was the last time you visited that health post?	*Record DD/MM. If don't know 99/99. If know month but Not day record 99/MM*	____/____
505h	The last time you visited the health post, what was the primary reason?	01=Family planning<br>02=Child immunisation<br>03=Antenatal care<br>04=Delivery care<br>05=Postnatal care<br>06=Neonatal care<br>07=Health education<br>08=Growth monitoring<br>09=Referral of sick child<br>10=Diarrhea treatment<br>11=Malaria treatment<br>13=Pneumonia treatment<br>14=other treatment of sickness<br>15=receive mosquito nets<br>88=other (specify)	____
506h	Is there another health facility in your kebele?	1= Yes<br>2=No **SKIP TO 518**	__
507h	What kind of a health facility	1=Health Centre<br>2=Hospital	__

For peer review only - http://bmjopen.bmj.com/site/about/guidelines.xhtml

		3=Private clinic<br>8=Other	
508h	How long does it take you to walk to the nearest health facility?	*Record the distance in minutes. If don't know, record 99*	____
509h	How many times have you visited the health facility in the last 6 months?	*Record the number of times*<br>*If any visits last 6 months,*<br>***SKIP TO 516***	__
*Do Not read out list, prompt, 'anything else' record 1=Yes for all that apply Then **SKIP TO 516***			
510h	If never in the last 6 months: What are the reasons why you have Not visited the health facility in the last six months?<br>**No illness in the family/No births**	1=Yes<br>2=No	__
511h	If never in the last 6 months: What are the reasons why you have Not visited the health facility in the last six months?<br>**Health facility is too far away**	1=Yes<br>2=No	__
512h	If never in the last 6 months: What are the reasons why you have Not visited the health facility in the last six months?<br>**Costs too much to go to health post**	1=Yes<br>2=No	__
513h	If never in the last 6 months: What are the reasons why you have Not visited the health facility in the last six months?<br>**Not enough time to visit**	1=Yes<br>2=No	__
514h	If never in the last 6 months: What are the reasons why you have Not visited the health facility in the last six months? **Poor services available at the health facility**	1=Yes<br>2=No	__
515h	If never in the last 6 months: What are the reasons why you have Not visited the health facility in the last six months? **Other**	Specify	________________
516h	When was the last time you visited that health facility?	*Record DD/MM. If don't know 99/99. If know month but Not day record 99/MM*	____/___
517h	The last time you visited the health facility, what was the primary reason?	01=Family planning<br>02=Child immunisation<br>03=Antenatal care<br>04=Delivery care<br>05=Postnatal care<br>06=Neonatal care<br>07=Health education<br>08=Growth monitoring<br>09=Referral of sick child<br>10=Diarrhoea treatment<br>11=Malaria treatment<br>13=Pneumonia treatment<br>14=Other treatment of sickness<br>15=Receive mosquito nets<br>88=Other (specify)	____
518h	Have you been visited at home during the past 6 months by a Health Extension Worker to talk about health related issues?	1= Yes<br>2=No<br>**SKIP TO 537**	__
519h	When was the last time the HEW visited you at home?	*Record DD/MM. If don't know 99/99. If know month but Not day record 99/MM*	____/____
Do Not read out list, prompt, 'anything else' record 1=Yes for all that apply			

For peer review only - http://bmjopen.bmj.com/site/about/guidelines.xhtml

520h	Who did the HEW talk to the last time she visited you at home? **Myself**	1=Yes<br>2=No	__
521h	Who did the HEW talk to the last time she visited you at home? **Other adult woman**	1=Yes<br>2=No	__
522h	Who did the HEW talk to the last time she visited you at home? **Head of household**	1=Yes<br>2=No	__
523h	Who did the HEW talk to the last time she visited you at home? **Other adult male**	1=Yes<br>2=No	__
Do Not read out list, prompt, 'anything else' record 1=Yes for all that apply			
524h	What was discussed the last time the HEW visited you at home? **Immunisation**	1= Yes<br>2=No	__
525h	What was discussed the last time the HEW visited you at home? **Child nutrition**	1=Yes<br>2=No	__
526h	What was discussed the last time the HEW visited you at home? **Family planning**	1=Yes<br>2=No	__
527h	What was discussed the last time the HEW visited you at home? **Pregnancy care**	1=Yes<br>2=No	__
528h	What was discussed the last time the HEW visited you at home? **Delivery care**	1=Yes<br>2=No	__
529h	What was discussed the last time the HEW visited you at home? **Newborn care / postnatal care**	1=Yes<br>2=No	__
530h	What was discussed the last time the HEW visited you at home? **Information about HIV/AIDS**	1=Yes<br>2=No	__
531h	What was discussed the last time the HEW visited you at home? **Information on hygiene**	1=Yes<br>2=No	__
532h	What was discussed the last time the HEW visited you at home? **Diarrhea treatment**	1=Yes<br>2=No	__
533h	What was discussed the last time the HEW visited you at home? **Malaria treatment**	1=Yes<br>2=No	__
534h	What was discussed the last time the HEW visited you at home? **Pneumonia treatment**	1=Yes<br>2=No	__
535h	What was discussed the last time the HEW visited you at home? **Promotion of latrine use**	1=Yes<br>2=No	__
536h	What was discussed the last time the HEW visited you at home? **Promotion of safe water use**	1=Yes<br>2=No	__
537h	Are you aware of women development army who visit people at home to talk about health issues?	1= Yes<br>2=No **SKIP TO 556**	__
538h	If Yes When was the last time the WDA visited you at home?	*Record DD/MM. If don't know 99/99. If know month but Not day record 99/MM*	____
Do Not read out list, prompt, 'anything else' record 1=Yes for all that apply			
539h	Who did the WDA talk to at that last visited to your home? **Myself**	1=Yes<br>2=No	__
540h	Who did the WDA talk to at that last visited to your home? **Other adult woman**	1=Yes<br>2=No	__
541h	Who did the WDA talk to at that last visited to your home? **Head of household**	1=Yes<br>2=No	__
542h	Who did the WDA talk to at that last visited to your home? **Other adult male**	1=Yes<br>2=No	__
Do Not read out list, prompt, 'anything else' record 1=Yes for all that apply			
543h	What was discussed the last time the WDA visited you at home? **Immunisation**	1=Yes<br>2=No	
544h	What was discussed the last time the WDA visited you at home? **Child nutrition**	1=Yes<br>2=No	__

For peer review only - http://bmjopen.bmj.com/site/about/guidelines.xhtml

545h	What was discussed the last time the WDA visited you at home? **Family planning**	1=Yes<br>2=No	__
546h	What was discussed the last time the WDA visited you at home? **Pregnancy care**	1=Yes<br>2=No	__
547h	What was discussed the last time the WDA visited you at home? **Delivery care**	1=Yes<br>2=No	__
548h	What was discussed the last time the WDA visited you at home? **Newborn care / postnatal care**	1=Yes<br>2=No	__
549h	What was discussed the last time the WDA visited you at home? **Information about HIV/AIDS**	1=Yes<br>2=No	__
550h	What was discussed the last time the WDA visited you at home? **Information on hygiene**	1=Yes<br>2=No	__
551h	What was discussed the last time the WDA visited you at home? **Diarrhoea treatment**	1=Yes<br>2=No	__
552h	What was discussed the last time the WDA visited you at home? **Malaria treatment**	1=Yes<br>2=No	__
553h	What was discussed the last time the WDA visited you at home? **Pneumonia treatment**	1=Yes<br>2=No	__
554h	What was discussed the last time the WDA visited you at home? **Promotion of latrine use**	1=Yes<br>2=No	__
555h	What was discussed the last time the WDA visited you at home? **Promotion of safe water use**	1=Yes<br>2=No	__
556h	Have you participated in developing local community-based action plan addressing maternal newborn and child health issues about health issues in the past 12 months?	1= Yes<br>2=No **SKIP TO 559**	__
557h	If Yes: When did you last participate in a community-based action plan?	*Record DD/MM. If don't know 99/99. If know month but Not day record 99/MM*	____/____
558h	Who organized the community-based action plan?	1=Kebele health team<br>2=Health extension worker<br>3=Community health worker<br>8=other (specify)<br>9=don't know	__
559h	Have you attended any meetings in your community (outside your home) about health issues in the past 12 months?	1= Yes<br>2=No **SKIP TO 575**	__
560h	If Yes: When was the last meeting you attended outside your home?	*Record DD/MM. If don't know 99/99. If know month but Not day record 99/MM*	____/____
561h	Who organised the last meeting?	1=Kebele health team<br>2=Health extension worker<br>3=Community health worker<br>8=other (specify)<br>9=don't know	__
Do Not read out list, prompt, 'anything else' record 1=Yes for all that apply			
562h	What was discussed at the last community meeting you attended outside your home? **Immunisation**	1= Yes<br>2=No	__
563h	What was discussed at the last community meeting you attended outside your home? **Child nutrition**	1= Yes<br>2=No	__
564h	What was discussed at the last community meeting you attended outside your home? **Family planning**	1= Yes<br>2=No	__
565h	What was discussed at the last community meeting you attended outside your home?	1= Yes<br>2=No	__

For peer review only - http://bmjopen.bmj.com/site/about/guidelines.xhtml

	**Pregnancy care**		
566h	What was discussed at the last community meeting you attended outside your home? **Delivery care**	1= Yes<br>2=No	__
567h	What was discussed at the last community meeting you attended outside your home? **Newborn care / postnatal care**	1= Yes<br>2=No	__
568h	What was discussed at the last community meeting you attended outside your home? **Information about HIV/AIDS**	1= Yes<br>2=No	__
569h	What was discussed at the last community meeting you attended outside your home? **Information on hygiene**	1= Yes<br>2=No	__
570h	What was discussed at the last community meeting you attended outside your home? **Diarrhea treatment**	1= Yes<br>2=No	__
571h	What was discussed at the last community meeting you attended outside your home? **Malaria treatment**	1= Yes<br>2=No	__
572h	What was discussed at the last community meeting you attended outside your home? **Pneumonia treatment**	1= Yes<br>2=No	__
573h	What was discussed at the last community meeting you attended outside your home? **Promotion of latrine use**	1= Yes<br>2=No	__
574h	What was discussed at the last community meeting you attended outside your home? **Promotion of safe water use**	1= Yes<br>2=No	__

For peer review only

For peer review only - http://bmjopen.bmj.com/site/about/guidelines.xhtml

Section 3.4: Identification of child & general questions			
The following questions are to be asked for all under 5 years old. Start with the youngest			
575h	***Interviewer***: What is the name of the youngest child**?**	Check household listing	___________
576h	***Interviewer***: select ID of the child from household listing		___
577h	Did you ever breastfeed [name]? OR Was [name] breastfed?	1=Yes<br>2=No<br>9=Don't know	__
578h	Are you still breastfeeding [name]? OR Is [name] still being breastfed?	1=Yes<br>2=No<br>9=Don't know	__
579h	For how many months did you breastfeed? OR For how many months was [name] breastfed?	*Record number of months. If don't know record 98*	__
580h	Did [name] receive a vitamin A dose (like this) during the last 6 months?	Show ampule/capsule/syrup<br>1=Yes<br>2=No<br>9=Don't know	__

Section 3.5: Immunizations			
581h	Do you have a card where [name's] vaccinations are written down? If Yes, may I see it?	1=Yes seen **SKIP TO 583**<br>2=Yes Not seen **SKIP TO 599**<br>3=No<br>9=Don't know	__
582h	If No card, did you ever have a vaccination card?	1=Yes<br>2=No<br>9=Don't know	__<br>If ALL answered **SKIP TO 599**
Record 44 in the DAY column if card shows that a vaccination was given but No date is recorded.) Record DD/MM/YYYY.			
583h	Copy vaccination date for **BCG** from the card	___/___/______	
584h	Copy vaccination date for **Polio 0 (Polio given at birth)** from the card	___/___/______	
585h	Copy vaccination date for **Polio 1** from the card	___/___/______	
586h	Copy vaccination date for **Polio 2** from the card	___/___/______	
587h	Copy vaccination date for **Polio 3** from the card	___/___/______	
588h	Copy vaccination date for **Pentavalent (DPT, HIV, Hep B)** from the card	___/___/______	
589h	Copy vaccination date for **Pentavalent 2** from the card	___/___/______	
590h	Copy vaccination date for **Pentavalent 3** from the card	___/___/______	
591h	Copy vaccination date for **PCV 1** from the card	___/___/______	

For peer review only - http://bmjopen.bmj.com/site/about/guidelines.xhtml

592h	Copy vaccination date for **PCV 2** from the card	____/____/________	
593	Copy vaccination date for **Rota 1** from the card	____/____/________	
594	Copy vaccination date for **Rota 2** from the card	____/____/________	
595	Copy vaccination date for **Rota 3** from the card	____/____/________	
596	Copy vaccination date for **Measles** from the card	____/____/________	
597	Copy vaccination date for **Vitamin A** from the card	____/____/________	
598	Copy vaccination date for **Deworming** from the card	____/____/________	
Ask and fill this question, only If the vaccination status in the card is empty (583-598)			
599h	Did [name] receive any vaccinations that are not on this card, including vaccinations received in a national immunization campaign?	1=Yes<br>2=No<br>9=Don't know	*Record Yes only if respondent mentions BCG, Polio 0-3, Pentavalent, and/or measles vaccine(s)*
600h	Did [name] receive **BCG**?	1=Yes<br>2=No<br>9=Don't know	__
601h	Did [name] receive **Polio 0 (Polio given at birth)?**	1=Yes<br>2=No<br>9=Don't know	__
602h	Did [name] receive **Polio 1**?	1=Yes<br>2=No<br>9=Don't know	__
603h	Did [name] receive **Polio 2?**	1=Yes<br>2=No<br>9=Don't know	__
604h	Did [name] receive **Polio 3**?	1=Yes<br>2=No<br>9=Don't know	__
605h	Did [name] receive **Pentavalent (DPT, HIV, Hep B)**?	1=Yes<br>2=No<br>9=Don't know	__
606h	Did [name] receive **Pentavalent 2**?	1=Yes<br>2=No<br>9=Don't know	__
607h	Did [name] receive **Pentavalent 3?**	1=Yes<br>2=No<br>9=Don't know	__
608h	Did [name] receive **PCV 1**?	1=Yes<br>2=No<br>9=Don't know	__
609h	Did [name] receive **PCV 2**?	1=Yes<br>2=No<br>9=Don't know	__
610h	Did [name] receive **Rota 1**?	1=Yes<br>2=No<br>9=Don't know	__

For peer review only

611h	Copy vaccination date for **Rota 2**?	1=Yes<br>2=No<br>9=Don't know	__
612h	Did [name] receive **Rota 3**?	1=Yes<br>2=No<br>9=Don't know	__
613h	Did [name] receive **Measles**?	1=Yes<br>2=No<br>9=Don't know	__
614h	Did [name] receive **Vitamin A**?	1=Yes<br>2=No<br>9=Don't know	__
615h	Did [name] receive **Deworming**?	1=Yes<br>2=No<br>9=Don't know	__
616h	Did [name] ever receive any vaccinations to prevent him/her getting diseases, including vaccinations received in a national immunization day campaign?	1=Yes<br>2=No<br>9=Don't know	__
Please tell me if [name] received any of the following vaccinations:			
617h	A BCG vaccination against tuberculosis, that is an injection in the arm or shoulder that usually cases a scar?	1=Yes<br>2=No<br>9=Don't know	__
618h	A POLIO vaccination, that is drops in the mouth?	1=Yes<br>2=No<br>9=Don't know	__
619h	When was the first polio vaccine received, just after birth or later?	1=just after birth<br>2=later	__
620h	How many times was the polio vaccine received?	Record number.<br>9=don't know	__
621h	A PENTAVALENT vaccination, which is an injection given in the thigh or buttocks, sometimes at the same time as the polio drops?	1=Yes<br>2=No<br>9=Don't know	__
622h	How many times was pentavalent received?	*Record number.*<br>*9=don't know*	__
623h	An injection to prevent MEASLES, usually in the upper left arm?	1=Yes<br>2=No<br>9=Don't know	__
624h	Has [name] had any illness at any time in the last 2 weeks?	1=Yes<br>2=No **Go to718**<br>9=Don't know **Go to 718**	__

For peer review only

Section 3.6: Two-Week Morbidity Module (symptoms, care seeking and drugs)			
FILL IN THE MODULE BELOW IF THE CHILD WAS ILL IN THE PREVIOUS TWO WEEKS. IF THE CHILD HAD MORE THAN ONE EPISODE, CONSIDER ONLY THE MOST RECENT ONE.			
625h	For how many days was [name] ill?	*If less than one day record 00*<br>*99 if don't know*	__
626h	Is [name] ill Now?	1=Yes<br>2=No	__
**Symptoms**			
627h	Did [name] have any of the following at any time in the last 2 weeks? **Convulsions**	1=Yes<br>2=No	__
628h	Did [name] have any of the following at any time in the last 2 weeks? **Very sleepy**	1=Yes<br>2=No	__
629h	Did [name] have any of the following at any time in the last 2 weeks? **Vomiting everything**	1=Yes<br>2=No	__
630h	Did [name] have any of the following at any time in the last 2 weeks? **Drinking poorly/Not able to drink or breastfeed**	1=Yes<br>2=No	__
631h	Did [name] have any of the following at any time in the last 2 weeks? **Fever**	1=Yes<br>2=No	__
632h	Did [name] have any of the following at any time in the last 2 weeks? **Cough**	1=Yes<br>2=No	__
633h	Did [name] have any of the following at any time in the last 2 weeks? **Difficult breathing**	1=Yes<br>2=No	__
634h	Did [name] have any of the following at any time in the last 2 weeks? **Diarrhea**	1=Yes<br>2=No	__
635h	Did [name] have any of the following at any time in the last 2 weeks? **Ear pain**	1=Yes<br>2=No	__
636h	Did [name] have any of the following at any time in the last 2 weeks? **Loss of appetite**	1=Yes<br>2=No	__
637h	Did [name] have any of the following at any time in the last 2 weeks? **Blocked or runny Nose**	1=Yes<br>2=No	__
638h	Did [name] have any of the following at any time in the last 2 weeks? **Fast breathing**	1=Yes<br>2=No	__
639h	Did [name] have any of the following at any time in the last 2 weeks? **Eye problems**	1=Yes<br>2=No	__
640h	Did [name] have any of the following at any time in the last 2 weeks? **Generalized rash**	1=Yes<br>2=No	__
641h	Did [name] have any of the following at any time in the last 2 weeks? **Other signs/symptoms**	1=Yes<br>2=No	__
642h	How many days ago did this start	*If less than one day record 00*<br>*99 if don't know*	____
If symptoms Fast Breathing or Difficult Breathing were selected ask the following question:			
643h	Were symptoms of fast or difficult breathing due to problem in the chest or to a blocked Nose?	1=Chest<br>2=Nose<br>3=Both	__

For peer review only - http://bmjopen.bmj.com/site/about/guidelines.xhtml

		8= Other<br>9=Don't know	
If Diarrhea was selected ask the following questions:			
644h	When [name] was sick with diarrhea, how many watery stools did [name] have?	If less than one day record 00<br>99 if don't know	____
645h	During the diarrhea, did [name] have?<br>**Repeated vomiting**	1=Yes<br>2=No	__
646h	During the diarrhea, did [name] have?<br>**Marked thirst**	1=Yes<br>2=No	__
647h	During the diarrhea, did [name] have?<br>**Not eating/drinking well**	1=Yes<br>2=No	__
648h	During the diarrhea, did [name] have?<br>**Blood in the stool**	1=Yes<br>2=No	__
649h	During the diarrhea, did [name] have?<br>**Not getting better/getting sicker**	1=Yes<br>2=No	__
650h	Now I would like to know how much [name] was offered to drink during the diarrhoea. Was he/she given less to drink than usual, more than usual, or about the same amount?<br>If less, probe: was he/she given *much less than usual to drink or somewhat less.*	1=Much less<br>2=Somewhat less<br>3=About the same<br>4=More<br>5=Nothing to drink<br>9=Don't know	__
651h	When [name] had diarrhea, was he/she given less to eat than usual, more than usual, or about the same amount?<br>*If less, probe: was he/she given much less than usual to eat or somewhat less.*	1=Much less<br>2=Somewhat less<br>3=About the same<br>4=More<br>5=Nothing to drink<br>9=Don't know	__
**Care seeking**			
652h	Did you seek advice or treatment for [name]'s illness from any source?	1= Yes **SKIP TO 660**<br>2= No<br>9=Don't know **SKIP TO 664**	__
653h	Why did you Not seek advice or treatment from any source? **Health facility too far**	1= Yes<br>2= No **SKIP TO** 718	__
654h	Why did you Not seek advice or treatment from any source? **Had no time**	1= Yes<br>2= No **SKIP TO** 718	__
655h	Why did you Not seek advice or treatment from any source? **Had no money**	1= Yes<br>2= No **SKIP TO** 718	__
656h	Why did you Not seek advice or treatment from any source? **Did Not want to attend a health facility**	1= Yes<br>2= No **SKIP TO** 718	__
657h	Why did you Not seek advice or treatment from any source? **Could manage at home**	1= Yes<br>2= No **SKIP TO** 718	__
658h	Why did you Not seek advice or treatment from any source? **Spouse did Not allow**	1= Yes<br>2= No **SKIP TO** 718	__
659h	Why did you Not seek advice or treatment from any source? **Other**	1= Yes<br>2= No **SKIP TO** 718	__
660h	Where did you seek advice or treatment? Anywhere else? **Health Post**	1= Yes<br>2= No	__
661h	Where did you seek advice or treatment? Anywhere else? **Health Centre**	1= Yes<br>2= No	__

For peer review only - http://bmjopen.bmj.com/site/about/guidelines.xhtml

662h	Where did you seek advice or treatment? Anywhere else? **Hospital**	1= Yes<br>2= No	__
663h	Where did you seek advice or treatment? Anywhere else? **Other**	Specify	______________
664h	***Interviewer:*** Check was treatment sought at two or more places?	1=Yes<br>2=No **SKIP TO 665**	__
665h	Where did you first seek treatment?	1=Health Post<br>2=Health Centre<br>3=Hospital<br>8=Other	__
666h	How many days after the illness began did you first seek treatment for [name]?	*If same day record 00. If don't know record 99.*	____
667h	Did the health worker ask you to bring [name] back to be seen again?	1=Yes<br>2=No<br>9=Don't know **SKIP TO 670**	__
668h	When did he/she say that [name] should be brought back?	1=Before today's date<br>2=After today's date **SKIP TO 670**<br>9=Don't know **SKIP TO 670**	__
669h	Did you take [name] back?	1=Yes<br>2=No<br>9=Don't know	__
670h	Has [name] been hospitalized for this illness?	1=Yes<br>2=No **SKIP TO 672**<br>9=Don't know **SKIP TO 672**	__
671h	How many nights has [name] been hospitalized during this illness?	*Record number of nights. If don't know record 99.*	____
672h	Has [name] been hospitalized at any time in the last 3 months?	1=Yes<br>2=No<br>9=Don't know	__
673h	How many nights has [name] been hospitalized in the last three months?	Record number of nights. If don't know record 99.	____
674h	At any time during this illness, did [name] have blood taken from his/her finger or heel for testing?	1=Yes<br>2=No **SKIP TO 678**<br>9=Don't know **SKIP TO 678**	__
675h	Which test was done?	1=Microscopy<br>2=RDT<br>9=Don't know	__
676h	Where was the test done?	1=Health Post<br>2=Health Centre<br>3=Hospital<br>8=Other	__
677h	Was the test positive for malaria?	1=Yes<br>2=No<br>9=Don't know	__
678h	In addition, did you seek advice from any of the following? **Traditional birth attendants**	1=Yes<br>2=No	__
679h	In addition, did you seek advice from any of the following? **Religious leader**	1=Yes<br>2=No	__
680h	In addition, did you seek advice from any of the following? **Pharmacy**	1=Yes<br>2=No	__
681h	In addition, did you seek advice from any of	1=Yes<br>2=No	__

For peer review only - http://bmjopen.bmj.com/site/about/guidelines.xhtml

	the following? **Traditional drug seller**		
682h	In addition, did you seek advice from any of the following? **Relative or friend**	1=Yes<br>2=No	__
683h	In addition, did you seek advice from any of the following? **Other**	**Specify**	___________

**Drugs**			
684h	At any time during the illness, was [name] prescribed any drugs for the illness?	1=Yes<br>2=No **SKIP TO 718**<br>9=Don't know **SKIP TO 718**	__
685h_a	What drugs did [name] take? **Antimalarial tablet (Co-artem, Chloroquine, etc)**	1= Yes<br>2 = No	__
685h_b	How long after the illness started did [name] first take **Antimalarial tablet**?	Write number of days. If child did Not take drug write 0 If 7 days or more write 7.<br>If don't know write 9	__
685h_c	**Did [name] complete taking the Antimalarial tablet?**	1= Yes<br>2 = No	__
685h_d	**Did you have [Antimalarial tablet] at home or did you get it from somewhere else.**<br><br>*If somewhere else, probe for the source.*	1=Home<br>2=Public health facility<br>3=Private health facility<br>4=HEW<br>5=Shop<br>8=Other Specify______<br>9=Don't know	__
694h_a	What drugs did [name] take?<br>**Amoxycillin**	1= Yes<br>2 = No	__
694h_b	How long after the illness started did [name] first take **Amoxycillin?**	Write number of days. If child did Not take drug write 0. If 7 days or more write 7.<br>If don't know write 9	__
694h_c	Did [name] complete taking the **Amoxycillin?**	1= Yes<br>2 = No	__
694h_d	Did you have **Amoxycillin** at home or did you get it from somewhere else.<br><br>*If somewhere else, probe for the source.*	1=Home<br>2=Public health facility<br>3=Private health facility<br>4=HEW<br>5=Shop<br>8=Other Specify______<br>9=Don't know	__
695h_a	What drugs did [name] take?<br>**Erythromycin**	1= Yes<br>2 = No	__
695h_b	How long after the illness started did [name] first take **Erythromycin?**	Write number of days. If child did Not take drug write 0. If 7 days or more write 7. If don't know write 9	__
695h_c	Did [name] complete taking the **Erythromycin?**	1= Yes<br>2 = No	__
695h_d	Did you have **Erythromycin** at home or did you get it from somewhere else?<br><br>*If somewhere else, probe for the source.*	1=Home<br>2=Public health facility<br>3=Private health facility<br>4=HEW<br>5=Shop<br>8=Other Specify______	__

For peer review only - http://bmjopen.bmj.com/site/about/guidelines.xhtml

		9=Don't know	
696h_a	What drugs did [name] take?<br>**Azithromycin**	1= Yes<br>2 = No	__
696h_b	How long after the illness started did [name] first take **Azithromycin?**	Write number of days. If child did Not take drug write 0. If 7 days or more write 7.<br>If don't know write 9	__
696h_c	Did [name] complete taking the **Azithromycin?**	1= Yes<br>2 = No	__
696h_d	Did you have **Azithromycin** at home or did you get it from somewhere else.<br><br>*If somewhere else, probe for the source.*	1=Home<br>2=Public health facility<br>3=Private health facility<br>4=HEW<br>5=Shop<br>8=Other Specify______<br>9=Don't know	__
697h_a	What drugs did [name] take?<br>**Cotrimoxazole**	1= Yes<br>2 = No	__
697h_b	How long after the illness started did [name] first take **Cotrimoxazole?**	Write number of days. If child did Not take drug write 0. If 7 days or more write 7.<br>If don't know write 9	__
697h_c	**Did [name] complete taking the Cotrimoxazole?**	1= Yes<br>2 = No	__
697h_d	Did you have **Cotrimoxazole** at home or did you get it from somewhere else.<br><br>*If somewhere else, probe for the source.*	1=Home<br>2=Public health facility<br>3=Private health facility<br>4=HEW<br>5=Shop<br>8=Other Specify______<br>9=Don't know	__
698h_a	What drugs did [name] take?<br>**Other antibiotic (pill or syrup)**	1= Yes<br>2 = No	__
698h_b	How long after the illness started did [name] first take **other antibiotic (pill or syrup)?**	Write number of days. If child did Not take drug write 0. If 7 days or more write 7.<br>If don't know write 9	__
698h_c	Did [name] complete taking **other antibiotic (pill or syrup)?**	1= Yes<br>2 = No	__
698h_d	Did you have **other antibiotic (pill or syrup)** at home or did you get it from somewhere else.<br><br>*If somewhere else, probe for the source.*	1=Home<br>2=Public health facility<br>3=Private health facility<br>4=HEW<br>5=Shop<br>8=Other Specify______<br>9=Don't know	__
699h_a	What drugs did [name] take?<br>**Antibiotic injection**	1= Yes<br>2 = No	__
699h_b	How long after the illness started did [name] first take **antibiotic injection?**	Write number of days. If child did Not take drug write 0. If 7 days or more write 7.<br>If don't know write 9	__
699h_c	Did [name] complete taking **antibiotic injection?**	1= Yes<br>2 = No	__
699h_d	Did you have **antibiotic injection** at home or did you get it from somewhere else**.**	1=Home<br>2=Public health facility<br>3=Private health facility	

For peer review only - http://bmjopen.bmj.com/site/about/guidelines.xhtml

	If somewhere else, probe for the source.	4=HEW<br>5=Shop<br>8=Other Specify_______<br>9=Don't know	__
700h_a	What drugs did [name] take?<br>**Zinc**	1= Yes<br>2 = No	__
700h_b	How long after the illness started did [name] first take **Zinc?**	Write number of days. If child did Not take drug write 0. If 7 days or more write 7. If don't know write 9	__
700h_c	Did [name] complete taking the **Zinc?**	1= Yes<br>2 = No	__
700h_d	Did you have **Zinc** at home or did you get it from somewhere else.<br>*If somewhere else, probe for the source.*	Write number of days. If child did Not take drug write 0 If 7 days or more write 7. If don't know write 9	__
701h_a	What drugs did [name] take?<br>**Fluid made from special packet called ORS**	1= Yes<br>2 = No	__
701h_b	How long after the illness started did [name] first take **fluid made from special packet called ORS**?	Write number of days. If child did Not take drug write 0. If 7 days or more write 7. If don't know write 9	__
702h_a	What drugs did [name] take?<br>**ORS-Zinc combined**	1= Yes<br>2 = No	__
702h_b	How long after the illness started did [name] first take **ORS-Zinc combined**?	Write number of days. If child did Not take drug write 0. If 7 days or more write 7. If don't know write 9	__
704h_a	What drugs did [name] take?<br>**Gov. recommended home mad fluid**	1= Yes<br>2 = No	__
704h_b	How long after the illness started did [name] first take **Gov. recommended home mad fluid?**	Write number of days. If child did Not take drug write 0. If 7 days or more write 7. If don't know write 9	__
705h_a	What drugs did [name] take?<br>**Pill or Syrup**	1= Yes<br>2 = No	__
705h_b	How long after the illness started did [name] first take **Pill or Syrup?**	Write number of days. If child did Not take drug write 0. If 7 days or more write 7. If don't know write 9	__
706h_a	What drugs did [name] take?<br>**Injection**	1= Yes<br>2 = No	__
706h_b	How long after the illness started did [name] first take **Injection**?	Write number of days. If child did Not take drug write 0. If 7 days or more write 7. If don't know write 9	__
707h_a	What drugs did [name] take?<br>**IV intravenous**	1= Yes<br>2 = No	__
707h_b	How long after the illness started did [name] first take **IV intravenous?**	Write number of days. If child did Not take drug write 0. If 7 days or more write 7. If don't know write 9	__
708h_a	What drugs did [name] take?<br>**Home remedies/herbal medicine**	1= Yes<br>2 = No	__
708h_b	How long after the illness started did [name] first take **home remedies/herbal medicine?**	Write number of days. If child did Not take drug write 0. If 7 days or more write 7. If don't know write 9	__
709h_a	What drugs did [name] take?<br>**Paracetamol**	1= Yes<br>2 = No	__
709h_b	How long after the illness started did [name]	Write number of days. If child did Not take drug	__

For peer review only - http://bmjopen.bmj.com/site/about/guidelines.xhtml

	first take **Paracetamol?**	write 0. If 7 days or more write 7.<br>If don't know write 9	
710h_a	What drugs did [name] take?<br>**Panadol**	1= Yes<br>2 = No	__
710h_b	How long after the illness started did [name] first take **Panadol?**	Write number of days. If child did Not take drug write 0. If 7 days or more write 7.<br>If don't know write 9	__
711h_a	What drugs did [name] take?<br>**Aspirin**	1= Yes<br>2 = No	__
711h_b	How long after the illness started did [name] first take **Aspirin?**	Write number of days. If child did Not take drug write 0. If 7 days or more write 7.<br>If don't know write 9	__
712h_a	What drugs did [name] take?<br>**Ibuprofen**	1= Yes<br>2 = No	__
712h_b	How long after the illness started did [name] first take **Ibuprofen**?	Write number of days. If child did Not take drug write 0. If 7 days or more write 7.<br>If don't know write 9	__
713h_a	What **other** drugs did [name] take?<br>**Other**	Specify	__
713h_b	How long after the illness started did [name] first take **other drug?**	Write number of days. If child did Not take drug write 0. If 7 days or more write 7.<br>If don't know write 9	__
714h	What **other** drugs did [name] take?<br>**Don't know**	1= Yes<br>2 = No	__

Section 3.7: MUAC

718h	Mid-upper arm circumference	*In centimeters*<br>*9994=If Not present*<br>*9995=Refused*<br>*9996=Other*	____|.__

For peer review only - http://bmjopen.bmj.com/site/about/guidelines.xhtml

ANNEX C: INSTRUMENTS FOR HEALTH PROVIDER SURVEY

Dagu Baseline Facility Questionnaire: Health Post

MODULE 1: BACKGROUND INFORMATION OF THE HEALTH POST

100	**Date (Gregorian Calendar)**	|___|___| / |___|___| / |___|___| dd / mm / yy	
101	**Region Code**	|__|__|__|__|__|__|__|	
102	**Zone Code**	|__|__|__|__|__|__|__||__|__|__|__|	
103	**Woreda Name**	|__|__|__|__|__|__|__||__|__|__|__|	
104	**Woreda code**	|__|__|__|__|__|__|__||__|__|__|__|	
105	**PHCU/Health Centre name**	|__|__|__|__|__|__|__||__|__|__|__|	
106	**Kebele name**	|__|__|__|__|__|__|__||__|__|__|__|	
107	**Gote name**	|__|__|__|__|__|__|__||__|__|__|__|	
108	**Cluster code**	|___|___|	
109	**GPS Latitude**<br>**Take coordinates of health post**	|___|___| : |___|___|___|___|___|	
110	**GPS Longitude**<br>**Take coordinates of health post**	|___|___| : |___|___|___|___|___|	
111	**Interviewer Initials**	|___|___|	
112	**Did you read the HEW the consent form?**	1 = Yes<br>2 = No	|___|
113	**Did the HEW agree to be interviewed?**	1= Yes – GO TO MODULE 2<br>2 = No	|___|
114	**If not, why not?**	|_________________________|	

MODULE 2: FACILITY, EQUIPMENT, MEDCINE AND JOB AIDS

INTERVIEWER: *I would now like to ask you questions about the facility, equipment, medicine and job aids at this health post.*

WALK AROUND THE FACILITY WITH THE HEW AND PERSONALLY CHECK THE AVAILABILITY OF EQUIPMENT, AND MEDICINE IN STOCK.

Does the facility have the following essential support services?

115	**What is the main source of drinking water?**	1 = Piped connection into health post<br>2 = Piped connection into yard<br>3 = Public standpipes<br>4 = Boreholes<br>5 = Protected dug wells<br>6 = Protected springs<br>7 = Rainwater collection<br>8 = Surface water<br>9 = Open dug wells<br>10 = Unprotected springs<br>11 = Vendor provided water<br>12 = Bottled water<br>13 = Tanker	|___|
116	**Water supply available on day of survey**	1 = Yes<br>2 = No	|___|
117	**Electricity connection or other power**	1 = Yes	|___|

	**sources (example, gas/solar generator)**	2 = No-**SKIP 119**	
118	**Electricity supply on day of survey?**	1 = Yes<br>2 = No	|___|
119	**Functional sterilizer that works on the day of the survey?**	1 = Yes<br>2 = No	|___|
120	**Functional fridge that works on the day of the survey?**	1 = Yes<br>2 = No	|___|
121	**Toilets accessible to facility users?**	1 = Yes<br>2 = No	|___|
122	**Generally is there a cell phone signal at this health post?**	1 = Yes<br>2 = No	|___|
123	**Is it all functional today?**<br>*INTERVIEWER -check your phone if there is signal on day of survey*	1 = Yes<br>2 = No	|___|
	**Which means of communication do you have to speak to the health facility?**<br><br>**Check all that apply**	For each:1 = Yes 2 = No	
		124 Facility landline/mobile phone	|___|
		125 Staff member mobile phone	|___|
		126 Phone outside the facility	|___|
		127 In person communication	|___|
128	**During the last sick newborn referral from the health post to health center, did you speak to the health facility directly?**	1 = Yes<br>2 = No – GO TO 130<br>3 =Don't know - GO TO 130	|___|
129	**IF YES, Which means of communication did you use?**	1 = Facility landline/mobile phone<br>2 = Staff member mobile phone;<br>3 = Phone outside the facility<br>4 = Radio<br>5 = In person communication;	|___|
130	**When referring from this health post to the health center for further care, do you use referral forms?**	1 = Yes (NOTE: check to see if an official referral form)<br>2 = No	|___|
131	**Do you receive any back referral forms on cases you have referred?**	1 = Yes<br>2 = No	|___|

**Walk around the facility with the respondent and personally check the availability of medicine**					
	**Does the facility have the following medicines today?**<br><br>*For each:*<br>*1 = Yes*<br>*2 = Not available*<br>*3= Expired*<br>*4 = Never available*			**If not available or expired, what was the duration of stock out /expiry for?**<br><br>*Check for the last three months and what was the longest number of consecutive days without that item (in days)*	
132	Vitamin k 1 mg	|___|	133	Vitamin k 1 mg	|__|__|__|
132A	Vitamin k 10 mg	|___|	132B	Vitamin k 10 mg	|__|__|__|
134	Vitamin A 200,000 IU	|___|	135	Vitamin A 200,000 IU	|__|__|__|
136	Vitamin A 100,000 IU	|___|	137	Vitamin A 100,000 IU	|__|__|__|
138	TTC eye ointment	|___|	139	TTC eye ointment	|__|__|__|
140	Chlorohexidine	|___|	141	Chlorohexidine	|__|__|__|

For peer review only

142	Gentamycin 20 mg/2ml, box of 50 amp	|___|	143	Gentamycin 20 mg/2ml, box of 50 amp	|__|__|__|
144	Gentamycin 80mg/2ml		145	Gentamycin 80mg/2ml	|__|__|__|
146	Amoxicillin suspension (125 mg/5 ml)	|___|	147	Amoxicillin suspension (125 mg/5 ml)	|__|__|__|
148	Amoxicillin tab 250 (dispersible)	|___|	149	Amoxicillin tab 250 (dispersible)	|__|__|__|
150	Amoxicillin tab 125 mg (dispersible)	|___|	151	Amoxicillin tab 125 mg (dispersible)	|__|__|__|
152	Ampicillin powder for inj, 500 mg	|___|	153	Ampicillin powder for inj, 500 mg	|__|__|__|
154	Paracetamol	|___|	155	Paracetamol	|__|__|__|
156	Iron	|___|	157	Iron	|__|__|__|
158	Folate	|___|	159	Folate	|__|__|__|
160	BCG	|___|	161	BCG	|__|__|__|
162	Polio vaccine	|___|	163	Polio vaccine	|__|__|__|
164	ORS	|___|	165	ORS	|__|__|__|
166	Zinc	|___|	167	Zinc	|__|__|__|
168	Zinc-ORS combined	|___|	169	Zinc-ORS combined	|__|__|__|
170	Malaria RDT	|___|	171	Malaria RDT	|__|__|__|
172	Coartem (Artemether/lumefantrine)	|___|	173	Coartem (Artemether/lumefantrine)	|__|__|__|
174	Chloroquine syrup	|___|	175	Chloroquine syrup	|__|__|__|
176	Artesunate suppository	|___|	177	Artesunate suppository	|__|__|__|
178	HIV diagnostic tests	|___|	179	HIV diagnostic tests	|__|__|__|
180	Cotrimoazole	|___|	181	Cotrimoazole	|__|__|__|
182	Plumpy nut	|___|	183	Plumpy nut	|__|__|__|
184	BP100	|___|	185	BP100	|__|__|__|

	**Does the facility have the following functional equipment today?**		*For each 1=yes, 2=no*	
		186	Ambu bag / face mask (full size 0 and 1)	|___|
		187	Any Thermometer	|___|
		188	Infant scale	|___|
		189	Weighing sling	|___|
		190	Blood pressure cuff	|___|
		191	Stethoscope	|___|
		192	Watch or clock	|___|
		193	Tape measure	|___|
		194	Examination couch	|___|
		195	Drape	|___|
		196	Washable mackintosh	|___|
		197	Dustbin	|___|

For peer review only

		198	Cups/drinking water	|___|
		199	Sharps container	|___|
		200	Chlorine bleach	|___|
		201	Bucket for decontamination solution	|___|
		202	Contaminated waste container	|___|
		203	Soap and towel or handrub	|___|
		204	Alcohol-based hand rub	|___|
		205	Clean glove	|___|
		206	Syringe with needle for Gentamycin injection	|___|
		207	MUAC tape measure	|___|
	**Does the facility have the following job aids and forms today?**		*For each 1=yes, 2=no*	
		208	Pregnant woman registration book	|___|
		209	ANC Register	|___|
		210	Delivery Register	|___|
		211	PNC Register	|___|
		212	ICCM registration book for 0- under 2 months	|___|
		213	ICCM registration book 2 -59 months	|___|
		214	Family health cards	|___|
		215	Vaccination cards	|___|
		216	Family folder	|___|
		217	Stock card/bin card	|___|
		218	HMIS forms (monthly and quarterly reporting)	|___|
		219	Request and re-supply form	|___|
		220	Chart booklet	|___|

MODULE 3: HEALTH POST REGISTER REVIEW BY THE DATA COLLECTOR

PLEASE LOOK AT THE SPECIFIED HEW REGISTERS DETAILED BELOW FOR THE DIFFERENT DATA ELEMENTS.
WRITE 9999, 999 OR 99 IF NOT AVAILABLE.
I would now like to take a look at your registers to abstract information about the community in this kebele and the services provided them. I will ask about the population profile for the last 12 months and services provided by you for the last three months from _________ to ________.

	**Obtain data on population FOR THE YEAR from the day of survey from Health Post wall records**	
221	Number of people in the kebele	|__|__|__|__|
222	Number of households in the kebele	|__|__|__|__|
223	Total number of women of reproductive age	|__|__|__|__|
224	Total number of under 5 children in the kebele	|__|__|__|__|
	**Obtain data on expected number of pregnancies and births from the PAST QUARTER from Health Post wall records**	
225	Expected number pregnancies	|__|__|__|
226	Expected number of births	|__|__|__|

For peer review only

227	Expected number of facility births	|__|__|__|
	**Obtain data from ANC registers for the PAST QUARTER**	
228	Number of women receiving 1 visit	|__|__|
229	Number of women receiving 2 visits	|__|__|
230	Number of women receiving 3 visits	|__|__|
231	Number of women receiving 4 visits	|__|__|
	**Obtain data from delivery registers for the PAST QUARTER**	
232	Number of births attended by the HEW	|__|__|
233	Number of total births (home, health post, health center, hospital)	|__|__|__|
234	Number of live births	|__|__|
235	Number of newborn deaths (28 days or less)	|__|__|
	**PNC data**	
236	Is there a Post Natal Care register (standard or otherwise) in this health post 1= Yes, 2=No	|__|
	**If Post Natal Care register is not available but family folder is available, obtain the information from the family folder given for the PAST QUARTER. Ask the HEWs to separate those.**	
237	Number receiving 1 visit	|__|__|
238	Number receiving 2 visits	|__|__|
239	Number receiving 3 visits	|__|__|
	**If information is not available from register books on the following, enquire from the HEW where to obtain the following information for the PAST QUARTER**	
240	Number of newborns treated for asphyxia, initial stimulation, or resuscitation by the HEW	|__|__|
241	Number of newborns given chlorohexidine cord care by the HEW	|__|__|
	**Obtain data from the ICCM 2-59 month register for the PAST QUARTER**	
242	Number of initial consultations with children	|__|__|__|
243	Number of sick children who were referred	|__|__|__|
244	Number of children classified as having pneumonia	|__|__|__|
245	Number of children classified as having diarrhea	|__|__|__|
246	Number of children classified as having malaria	|__|__|__|
247	Number of children who received antibiotic for pneumonia	|__|__|__|
248	Number of children who received ORS for diarrhea	|__|__|__|
249	Number of children who received zinc for diarrhea	|__|__|__|
250	Number of children who received zinc-ORS combined for diarrhea	|__|__|__|
251	Number of children who received RDT for malaria	|__|__|__|
252	Number of children who received ACT for malaria	|__|__|__|

For peer review only

Obtain data from iCCM 0-2 month registration book for information below for the PAST QUARTER
For each of the newborn less than 2 months old seen at the health center (recorded above) complete a separate record review.

Record 1

253A	How many 0-2 months were seen in the last quarter?		|___|___|___|
253	**Name of child**	________________ First name<br>________________ Last name	
254	**Address of child**	________________ Gote name<br>________________ Keble name	
255	**Date Seen**	*Gregorian calendar (DD/MM/YY)*	|__|__|/|__|__|/|__|__|
256	**Age of baby at the time of consultation in weeks**	*Record age of baby in weeks ranging from 1-8 weeks.*<br>*If unknown 9*	|___| weeks
257	**Gender of baby**	*1 = Male*<br>*2 = Female*	|___|
258	**Weight on the day of consultation in grams**	*If weight is given in KGs record in grams e.g 3.5 KG = 3500 grams.*<br>*If unknown 9999*	|___|___|___|___| grams
259	**Birth Weight**<br>**(Written for those less than 7 days)**	*1= < 1,500 grams*<br>*2= 1,500 - < 2,500 grams*<br>*3= >/= 2,500 grams*<br>*9= Unknown*	|___|
260	**Gestational Age (in weeks)**	*1= < 32 weeks*<br>*2= 32 – 36 weeks*<br>*3= >/= 37 weeks*<br>*9= Unknown*	|___|
261	**Temperature on the day of consultation in degree Celsius**	*Record temperature to one decimal place (e.g. 34.3 ℃)*<br>*If unknown 99.9*	|___|___|.|___|℃
262	**Respiratory Rate per minute on the day of consultation**	*If unknown 999*	|___|___|___|

	**Signs and symptoms of the newborn at the time of consultation?**<br><br>**Record all that apply**		For each:1 = Yes 2 = No	
		263	Reduced feeding/unable to feed	|___|
		264	Convulsion	|___|
		265	Severe Chest in-drawing	|___|
		266	Vomiting	|___|
		267	Fever	|___|
		268	Diarrhea	|___|
		269	Fast breathing	|___|
		270	Coughing	|___|
		271	Grunting	|___|
		272	Skin pustules	|___|
		273	Yellow palms and soles	|___|
		274	Yellow eyes and skin	|___|
		275	Red umbilicus or draining pus	|___|
		276	Movement only when stimulated or no movement even when stimulated	|___|
		277	Movement only when stimulated or no movement even when stimulated	|___|

For peer review only

		278	Bulging fontanelle	|___|
		279	Restless/Irritable	|___|
		280	Sunken eyes	|___|
		281	Skin pinch goes back slowly	|___|
		282	Skin pinch goes back very slowly	|___|
		283	Diarrhea lasting 14 days or more	|___|
		284	Blood in the stool	|___|
		285	Not suckling well	|___|
		286	Less than 8 breast feeds in 24 hours	|___|
		287	Switching to another breast before one is emptied	|___|
		288	Not breast feeding more frequently and longer during sickness	|___|
		289	Poor positioning during breast feeding	|___|
		290	Not well attached during breast feeding	|___|
		291	Receives other foods or drinks (even water)	|___|
		292	Low weight for age	|___|
		293	Thrush (ulcers or white patches in mouth)	|___|
		294	Signs and symptoms not given	|___|
		295	Other – GO TO 297	|___|
		296	Specify _______________	
	**Disease classification of the newborn** **Record all that apply**		For each:1 = Yes 2 = No	
		297	Very Preterm and/or very low birth weight	|___|
		298	Preterm and/or low birth weight	|___|
		299	VSD	|___|
		300	Local bacterial infection	|___|
		301	Severe Dehydration	|___|
		302	Some Dehydration	|___|
		303	No Dehydration	|___|
		304	Severe Persistent Diarrhea	|___|
		305	Dysentery	|___|
		306	Jaundice	|___|
		307	Severe Jaundice	|___|
		308	Malaria	|___|
		309	Feeding problem or low weight	|___|
		310	Classification not given	|___|
		311	Other Go to 313	|___|
		312	Specify _______________	
	**Treatment given to the newborn** **Record all that apply**		For each:1 = Yes 2 = No	
		313	Gentamycin IM first dose	|___|
		314	Gentamycin IM for seven days	|___|
		315	Amoxicillin suspension/dispersible first dose	|___|
		316	Amoxicillin suspension/dispersible for 7 days	|___|
		317	Amoxicillin suspension/dispersible for 5 days	|___|
		318	ORS (Plan B) – Facility treatment	|___|
		319	ORS (Plan A) – Home treatment	|___|
		320	Zinc for 10 days	|___|
		321	Zinc-ORS combined	|___|
		322	Oral chloroquine (Anti-malarial)	|___|
		323	Oral quinine (Anti-malarial)	|___|
		324	Oral coartem (Anti-malarial)	|___|
		325	Rectal Artesunate (Anti-malarial)	|___|
		326	IV Quinine (Anti-malarial)	|___|
		327	Other Antimalarial (specify)	|___|

For peer review only

		328	TTC (Tetracycline) eye ointment	|___|
		329	GV paint (Gentian Violet)	|___|
		330	Nutritional Counseling	|___|
		331	Exposing to sunshine 20– 30 minutes everyday	|___|
		332	Other treatment GO TO 334	|___|
		333	Specify ______________	
334	**Was newborn referred to a higher facility?**		1 = Yes – GO TO 337<br>2 = No	|___|
335	**If newborn had VSD and was treated at health post was gentamycin treatment completed?**		1 = Yes<br>2 = No<br>3 = Not VSD case	|___|
	**Outcome of the newborn treatment**		For each:1 = Yes 2 = No	
		336	Health improved/healed	|___|
		337	Died	|___|
		338	Worsened	|___|
		339	Same	|___|
		340	Unknown	|___|

For peer review only

Obtain data from iCCM 2-59 registration book for information below for the PAST QUARTER
For the past 10 children seen at the health post (recorded above) complete a separate record review.

Record 1

341A	**How many 2-59 months were seen in the last quarter?**		|___|___|___|
341	**Name of child**	________________ First name<br>________________ Last name	
342	**Address of child**	________________ Gote name<br>________________ Keble name	
343	**Date Seen**	*Gregorian calendar (DD/MM/YY)*	|__|__|/|__|__|/|__|__|
344	**Age of child at the time of consultation**	*Record age of child in months.*<br>*If unknown 99*	|___|___| months
345	**Gender**	*1 = Male*<br>*2 = Female*	|___|
346	**Weight on the day of consultation in grams**	*If weight is given in KGs record in grams*<br>*e.g 3.5 KG = 3500 grams.*<br>*If unknown 9999*	|___|___|___|___| grams
347	**Temperature on the day of consultation in degree Celsius**	*Record temperature to one decimal place (e.g. 34.3 ℃)*<br>*If unknown 99.9*	|___|___|.|___|℃
348	**Respiratory Rate per minute on the day of consultation**	*If unknown 999*	|___|___|___|

	**Signs and symptoms of the child at the time of consultation?**<br><br>**Record all that apply**		For each:1 = Yes 2 = No	
		349	Reduced feeding/unable to feed	|___|
		350	Vomiting	|___|
		351	Convulsion	|___|
		352	Movement only when stimulated or no movement even when stimulated	|___|
		353	Movement only when stimulated or no movement even when stimulated	|___|
		354	Restless/Irritable	|___|
		355	Difficult breathing	|___|
		356	Fast breathing	|___|
		357	Coughing	|___|
		358	Severe Chest in-drawing	|___|
		359	Stridor	|___|
		360	Diarrhea	|___|
		361	Diarrhea lasting 14 days or more	|___|
		362	Blood in the stool	|___|
		363	Skin pinch goes back slowly	|___|
		364	Skin pinch goes back very slowly	|___|
		365	Fever	|___|
		366	Bulging fontanelle	|___|
		367	Rash	|___|
		368	Mouth ulcers	|___|
		369	Pus or clouding of cornea	|___|
		370	Perform malaria RDT	|___|
		371	Discharge / pus in the ear	|___|
		372	Swelling	|___|
		373	Visible severe wasting	|___|

For peer review only

		374	Palmor pallor	|___|
		375	Signs and symptoms not given	|___|
		376	Other – GO TO 377	|___|
		377	Specify _______________	
	**Disease classification of the child**<br>**Record all that apply**		For each:1 = Yes 2 = No	
		378	One or more danger signs (unable to drink or breastfeed, vomits everything, convulsions, movement only when stimulated or no movement even when stimulated	|___|
		379	Severe pneumonia/very severe disease	|___|
		380	Pneumonia	|___|
		381	Severe dehydration	|___|
		382	Some dehydration	|___|
		383	Severe persistent diarrhoea	|___|
		384	Persistent diarrhoea	|___|
		385	Dysentery	|___|
		386	Very severe febrile disease	|___|
		387	Malaria	|___|
		388	Fever, malaria unlikely	|___|
		389	Fever, no malaria	|___|
		390	Severe complicated measles	|___|
		391	Measles with eye/mouth complications	|___|
		392	Measles	|___|
		393	Acute ear infection	|___|
		394	Chronic ear infection	|___|
		395	Severe malnutrition	|___|
		396	Moderate malnutrition	|___|
		397	Severe anaemia	|___|
		398	Anaemia	|___|
		399	Vaccination status not up-to-date	|___|
		400	Vaccine(s) needed (specify)	|___|
		401	Vitamin A status not up-to-date	|___|
		402	Classification not given	|___|
		403	Other Go to 404	|___|
		404	Specify _______________	
	**Treatment given to the child**<br>**Record all that apply**		For each:1 = Yes 2 = No	
		405	ORS (Plan A) – Home treatment	|___|
		406	ORS (Plan B) – Facility treatment	|___|
		407	Zinc for ten days	|___|
		408	Oral coartem (Anti-malarial)	|___|
		409	Oral chloroquine (Anti-malarial)	|___|
		410	Oral quinine (Anti-malarial)	|___|
		411	Rectal Artesunate (Anti-malarial)	|___|
		412	IV Quinine (Anti-malarial)	|___|
		413	Other Antimalarial (specify)	|___|
		414	Cotrimoxazole	|___|
		415	Vitamin A	|___|
		416	Paracetamol	|___|
		417	Gentamycin IM first dose	|___|
		418	Gentamycin IM for seven days	|___|
		419	Amoxicillin suspension/dispersible first dose	|___|
		420	Amoxicillin suspension/dispersible for seven days	|___|
		421	Amoxicillin suspension/dispersible for five days	|___|

For peer review only

		422	TTC (Tetracycline) eye ointment	|___|
		423	Plumpy nut	|___|
		424	BP100	|___|
		425	Nutritional Counseling	|___|
		426	Other treatment GO TO 427	|___|
		427	Specify _______________	
428	**Was the child referred to a higher facility?**		1 = Yes<br>2 = No	|___|
	**Outcome of the child treatment**		For each:1 = Yes 2 = No	
		429	Health improved/healed	|___|
		430	Died	|___|
		431	Worsened	|___|
		432	Same	|___|
		433	Unknown	|___|

For peer review only

OHEP HEW Questionnaire

Unique ID-----------

100	**Date (dd/mm/yyyy)**<br>**Gregorian Calendar**	|___|___| / |___|___| / |___|___| dd / mm / yy	
101	**Region Name**	|__|__|__|__|__|__||__|__|__|__|	
102	**Zone Name**	|__|__|__|__|__|__||__|__|__|__|	
103	**Woreda Name**	|__|__|__|__|__|__||__|__|__|__|	
104	**PHCU/Health Center Name**	|__|__|__|__|__|__||__|__|__|__|	
104A	**Health post code**	|__|	
104B	**HEW code**	|__|	
105	**Kebele**	|__|__|__|__|__|__||__|__|__|__|	
106	**Gote**	|__|__|__|__|__|__||__|__|__|__|	
107	**Cluster Code**	|___|___|	
108	**GPS Latitude**	|___|___| : |___|___|___|___|___|	
109	**GPS Longitude**	|___|___| : |___|___|___|___|___|	
ELEVATION	**Elevation**		
110	**Interviewer Initials**	**|___|___|**	
111	**Did you read the HEW the consent form?**	1 = Yes<br>2 = No	|___|
112	**Did the HEW agree to be interviewed?**	1= Yes – GO TO MODULE 2<br>2 = No	|___|
113	**If not, why not?**	______________________________ **END**	

For peer review only - http://bmjopen.bmj.com/site/about/guidelines.xhtml

Module: 2

114	**What is your name?**		
115 Y	**What is your date of birth?**	yyyy |___|__|___|__| <br>Ethiopian Calendar	
115 M	**What is your date of birth?**	mm|___|__| Ethiopian Calendar	
115 D	**What is your date of birth?**	dd |___|__| Ethiopian Calendar	
116	**What is the number of years you attended school?**	Write number of years	|___||___| Years
117	**As an HEW, what is your level?**	1 = Level 1<br>2 = Level 2<br>3 = Level 3<br>4 = Level 4	|___|
118	**Do you have any specific qualification in addition to HEW training?**	1 = Yes<br>2 = No – GO TO 120	|___|
119	**If yes, specify**	___________________________	
120 YY	**For how long have you worked as an HEW (including work at other kebeles)?**	Write number of years.	|___||___| Years
120MM	If less than one year, enter number of months only.		|___||___| Months
121YY	**For how long have you worked as an HEW in this Health post?**	Write number of years and months.	|___||___| Years |___||___| Months
121MM	If less than one year, enter 00 years and number of months		
122	**Do you reside in this kebele?**	1 = Yes<br>2 = No Skip to 124	|___|
123	**Was a home provided to you by the kebele?**	1 = Yes<br>2 = No	|___|
124	**How many HEWs work in this health post?**	Enter number, including the person being interviewed	|___|
125	**In this health post who is the HEW in charge (senior HEW)?**	1 = Myself<br>2 = Other HEW<br>3 = Neither of us	|___|

For peer review only - http://bmjopen.bmj.com/site/about/guidelines.xhtml

126	**How many days a week is the health post facility functionally open by at least 1 HEW?**	Enter number of days	|___|
127	**Do you post the health post functional days for clients to see?**	1 = Yes<br>2 = No	|___|
128	**Do you post the health posts hours of operation for clients to see?**	1 = Yes<br>2 = No	|___|

**During the weekend and public holidays- where do the residents of the kebele seek medical care?**<br><br>Select all that apply		For each: 1 = Yes 2 = No	
	129	**Health center**	|___|
	130	**Health Post**	|___|
	131	**With HEW (at her house or elsewhere)**	|___|
	132	**Pharmacy**	|___|
	133	**Traditional Healers**	
	134	**Other –**	|___|
	135	Specify ______________________________	

Instruction: From Question HEW_132-HEW_207
Don’t prompt. Select all mentioned

**What are the main components of immediate newborn care?**<br><br>Do not prompt<br>Select all mentioned.	**For each: 1 = Yes 2 = No**		
	136	Deliver baby onto mother’s abdomen	|___|
	137	Dry and wrap baby	|___|
	138	Assess breathing	|___|
	139	Delay cord clamping for three minutes	|___|
	140	Tie and cut cord appropriately	|___|
	141	Skin to skin contact	|___|
	142	Initiate breastfeeding	|___|
	143	Apply TTC eye ointment	|___|
	144	Apply chlorohexidine on cord	|___|
	145	Give Vitamin K	|___|
	146	Weight baby	|___|
	999	None mentioned from the list	
**What are the main components of the first PNC visit for newborn?**	**For each: 1 = Yes 2 = No**		
	147	Advice washing hands before touching baby	|___|

For peer review only - http://bmjopen.bmj.com/site/about/guidelines.xhtml

Do not prompt<br>Select all mentioned	148	Check for danger sings	|___|
	149	Check for congenital abnormalities	|___|
	150	Measure temp	|___|
	151	Measure weight	|___|
	152	Apply TTC eye ointment	|___|
	153	Encourage exclusive breast feeding for baby	|___|
	154	Advice to delay bathing of baby for 24 hrs	|___|
	155	Encourage skin to skin contact	|___|
	156	Provide cord care (Chlorohexidine)	|___|
	157	Education on appropriate cord care (Chlorohexidine)	|___|
	158	Vaccinate for polio and BCG	|___|
	159	Teach mother on how to recognize newborn danger signs using family health card.	|___|
	999	None mentioned from the list	
**What are the main components of <u>subsequent (3rd and 7th day and 6th week) PNC visits</u> for newborn?**<br><br>Do not prompt<br>Select all mentioned	**For each: 1 = Yes 2 = No**		
	160	Check for newborn danger signs	|___|
	161	Advice to keep cord clean	|___|
	162	Assess breastfeeding	|___|
	163	Advise on breastfeeding	|___|
	164	Ensure baby is kept warm	|___|
	165	Check baby's weight	|___|
	166	Vaccination	|___|
	999	None mentioned from the list	
**When a newborn weighs less than 1.5 kgs or has a gestational age of less than 32 weeks, what special care do you provide?**<br><br>Do not prompt<br>Select all mentioned	**For each: 1 = Yes 2 = No**		
	167	Continue feeding with expressed breast milk	|___|
	168	Monitor ability to breastfeed	|___|
	169	Cover baby well including head	|___|
	170	Hold close to mother	|___|
	171	Refer urgently with mother to health center or hospital	|___|
	999	None mentioned from the list	
**When a newborn weighs between1.5 - 2.5 kgs or has a gestational age of 32-<37 weeks, what special care do you provide?**<br><br>Do not prompt<br>Select all mentioned	**For each: 1 = Yes 2 = No**		
	172	Make sure the baby is warm	|___|
	173	Educate on optimal breastfeeding	|___|
	174	Monitor ability to breastfeed	|___|
	175	Monitor baby for the first 24 hours	|___|
	176	Educate on infection prevention	|___|
	999	None mentioned from the list	
**What are the main signs for good attachment**	**For each: 1 = Yes 2 = No**		

For peer review only

**during breast feeding?**<br><br>Do not prompt<br>Select all mentioned	177	Chin touching breast	|___|
	178	Mouth open wide	|___|
	179	Lower lip turned out	|___|
	180	More areola showing above	|___|
	999	None mentioned from the list	
**How do you determine feeding problems in a newborn?**<br><br>Do not prompt<br>Select all mentioned	**For each: 1 = Yes 2 = No**		
	181	Not well-attached to breast	|___|
	182	Not suckling effectively	|___|
	183	Less than 8 breastfeeds in 24 hours	|___|
	184	Switching to another breast before one is emptied	|___|
	185	Receives other foods or drinks (even water)	|___|
	186	Underweight for age	|___|
	187	Thrush (ulcers or white patches in mouth)	|___|
	999	None mentioned from the list	
**When a newborn shows signs of feeding problems or is underweight, what initial steps do you take?**<br><br>Do not prompt<br>Select all mentioned	**For each: 1 = Yes 2 = No**		
	188	Advise mother to breastfeed as often and as long as infant wants in 24 hours	|___|
	189	Teach mother correct positioning and attachment	|___|
	190	Educate on exclusive breastfeeding	|___|
	191	Teach the mother to treat thrush at home	|___|
	192	Follow-up any feeding problem	|___|
	193	Follow-up any thrush in two days	|___|
	194	Follow-up underweight for age in 14 days	|___|
	999	None mentioned from the list	
**What are the main signs for very severe disease in newborns?**<br><br>Do not prompt<br>Select all mentioned	**For each: 1 = Yes 2 = No**		
	195	Convulsions	|___|
	196	Stopped feeding or significantly reduced feeding	|___|
	197	Severe chest in drawing	|___|
	198	Fast breathing	|___|
	199	Temperature with 37.5 or more (warm)<br>*(Note: if high temperature only mentioned ask for clarification to what extent)*	|___|
	200	Temperature less than 35.5 (cold)<br>(*Note: if low temperature only mentioned ask for clarification to what extent*)	|___|
	201	No or very limited movement on stimulation	|___|

For peer review only
For peer review only - http://bmjopen.bmj.com/site/about/guidelines.xhtml

	999	None mentioned from the list	
**When the newborn presents sign of very severe disease, what initial steps do you take?**<br><br>Do not prompt<br>Select all mentioned	**For each: 1 = Yes 2 = No**		
	202	Continue to breastfeed or if unable to suck give breast milk that has been expressed	|___|
	203	Begin a dose of amoxicillin (pre-referral)	|___|
	204	Begin a dose of gentamycin antibiotics (pre-referral)	|___|
	205	Refer URGENTLY	|___|
	206	When referral is not possible treat with/prescribe amoxicillin for 7 days	|___|
	207	When referral is not possible treat with gentamycin daily for 7 days	|___|
	999	None mentioned from the list	
**What are the main signs for local bacterial infection in newborns?**<br><br>Do not prompt<br>Select all mentioned	**For each: 1 = Yes 2 = No**		
	208	Umbilicus red	|___|
	209	Umbilicus draining pus	|___|
	210	Skin pustules	|___|
	999	None mentioned from the list	
**When the newborn presents signs of local bacterial infection, what initial steps do you take?**<br><br>Do not prompt<br>Select all mentioned	**For each: 1 = Yes 2 = No**		
	211	Give amoxicillin syrup for 5 days	|___|
	212	Follow up care on 2nd day from initial visit	|___|
	213	Advice mother when to return	|___|
	214	Breastfeed more frequently	|___|
	215	Advice mother to give breast milk more frequently	|___|
	216	Advice mother to keep baby warm	|___|
	999	None mentioned from the list	
217	Are there any possible side effects of using injectable gentamicin for neonatal illness?	**1= Yes –**<br>**2 = No – Skip to 228**	|___|
	**For each: 1 = Yes 2 = No**		
218	What are the possible side effects of using inject able gentamicin for neonatal illness?		
**What are the possible side effects of using injectable gentamicin for neonatal illness?**	218	Kidney damage (nephropathy)	|___|
	219	Nerve damage (neuropathy especially hearing or visual damage)	|___|
	220	Hearing loss	|___|
	221	Lethargy	|___|
	222	Nausea/vomiting	|___|

For peer review only - http://bmjopen.bmj.com/site/about/guidelines.xhtml

		223	General anaphylactic reaction	|___|
		224	Fever	|___|
		225	Poor appetite	|___|
		226	Weight loss	|___|
		227	Skin rash	|___|
228	Is there any contraindication of using injectable gentamicin for the neonatal illness?		**1= Yes**<br>**2 = No – 232**	
	**What are those possible contraindications of using injectable gentamicin for the neonatal illness?**	**For each: 1 = Yes 2 = No**		
		229	History of general body reaction or shock to injectable gentamicin (Anaphylactic reaction	|___|
		230	History of kidney/urine problem	|___|
		231	History of skin reaction to gentamicin	|___|
232	**Are there any possible side effects of using amoxicillin for the neonatal illness?**		**1= Yes**<br>**2 = No – 234**	|___|
			**For each: 1 = Yes 2 = No**	
233	**What are the possible side effects of using amoxicillin for the neonatal illness?**		1 = General anaphylactic reaction (penicillin hypersensitivity)<br>9.Not mentioned	|___|
234	**Is there any contraindication of using amoxicillin for the neonatal illness?**		**1= Yes**<br>**2 = No – GO TO 236**	|___|
			**For each: 1 = Yes 2 = No**	
235	**What are those possible contraindications of using amoxicillin for the neonatal illness**		1 = History of General body reaction or shock to amoxicillin (penicillin hypersensitivity)<br>9.Not mentioned	|___|
236	**Are there any possible additional side effects of using antibiotics (injectable gentamicin or amoxicillin) for non-severe neonatal illness?**		**1= Yes**<br>**2 = No – 238**	|___|
			**For each: 1 = Yes 2 = No**	
237	**What are those additional side effects of using antibiotics (injectable gentamicin or amoxicillin) for non-severe neonatal illness?**		1 = Drug resistance<br>9.Not mentioned	|___|
	**What are the main signs for jaundice in newborns?**<br><br>Do not prompt<br>Select all mentioned	**For each: 1 = Yes 2 = No**		
		238	Yellow skin	|___|
		239	Yellow eyes	|___|
		999	Not mentioned	
	**When the newborn presents signs of jaundice, what initial steps do you take?**<br><br>Do not prompt<br>Select all mentioned	**For each: 1 = Yes 2 = No**		
		240	Breastfeed more frequently	|___|
		241	Advise mother to keep young infant warm	|___|
		242	Expose to sunshine 20 to 30 minutes every day	|___|

For peer review only

	243	Advise mother to return immediately if sign & symptoms of jaundice aggravates	|___|
	244	Follow-up in 2 days	|___|
**What are the main symptoms/signs for severe jaundice in newborns?**<br><br>Do not prompt<br>Select all mentioned	**For each: 1 = Yes 2 = No**		
	245	Jaundice in newborns of Age 14 days or more	|___|
	246	Jaundice in newborns of Age less than 24 hours	|___|
	247	Palms yellow	|___|
	248	Soles yellow	|___|
	999	Not mentioned from the list	
**When the newborn presents symptoms /signs of severe jaundice what initial steps do you take?**<br><br>Do not prompt<br>Select all mentioned	**For each: 1 = Yes 2 = No**		
	249	Breastfeed more frequently	|___|
	250	Refer URGENTLY to health center /hospital	|___|
	251	Keep the baby warm	|___|
**What are the main signs for some dehydration caused by diarrhea in newborns?**<br><br>Do not prompt<br>Select all mentioned	**For each: 1 = Yes 2 = No**		
	252	Restless and irritable	|___|
	253	Sunken eyes	|___|
	254	Skin pinch goes back slowly	|___|
	999	None mentioned from the list	
**When the newborn presents signs of some dehydration caused by diarrhea what initial steps do you take?**<br><br>Do not prompt<br>Select all mentioned	**For each: 1 = Yes 2 = No**		
	255	Give ORS fluids	|___|
	256	Give zinc treatment for 10 days	|___|
	257	Advise mother to breastfeed more frequently and longer	|___|
	258	Keep the infant warm	|___|
	259	Advise mother when to return	|___|
	260	Follow up in 2 days	|___|
	999	None mentioned from the list	
**What are the main symptoms/signs for severe dehydration caused by diarrhea in newborns?**<br><br>Do not prompt<br>Select all mentioned	**For each: 1 = Yes 2 = No**		
	261	Limited or No movement even when stimulated	|___|
	262	Sunken eyes	|___|
	263	Skin pinch goes back VERY slowly	|___|
	999	None mentioned from the list	
**When the newborn presents signs of severe dehydration caused by diarrhea what initial steps do you take?**<br><br>Do not prompt<br>Select all mentioned			
	264	Give first dose of amoxicillin syrup	|___|
	265	Give first dose of IM Gentamycin	|___|
	266	Refer URGENTLY to health center/hospital	|___|
	267	Ensure mother gives child ORS on the way to health center/hospital	|___|
	268	Advise mother to breastfeed more	|___|

For peer review only - http://bmjopen.bmj.com/site/about/guidelines.xhtml

For peer review only

		frequently and longer	
	269	Advice mother to keep young infant warm	|___|
**What are the main signs and symptoms of pneumonia in a child aged 2-59 months?**<br><br>Do not prompt<br>Select all mentioned	**For each: 1 = Yes 2 = No**		
	270	Cough	|___|
	271	Difficult or fast breathing	|___|
	272	Chest indrawing	|___|
	273	Stridor	|___|
	999		
**When the child presents signs and symptoms of pneumonia what initial steps do you take?**<br><br>Do not prompt<br>Select all mentioned	**For each: 1 = Yes 2 = No**		
	274	Give antibiotics	|___|
	275	Advise on when/how to administer	|___|
	276	Keep the child warm	|___|
	277	Advise mother when to return	|___|
	999	None mentioned from the list	
**What are the main signs and symptoms of diarrhea in a child aged 2-59 months?**<br><br>Do not prompt<br>Select all mentioned	**For each: 1 = Yes 2 = No**		
	278	Diarrhea	
	279	Blood in stool	
	280	Restless or irritable	
	281	Sunken eyes	|___|
	282	Skin pinch goes back slowly	|___|
	999	None mentioned from the list	
**When the child presents signs and symptoms of diarrhea what initial steps do you take?**<br><br>Do not prompt<br>Select all mentioned	**For each: 1 = Yes 2 = No**		
	283	Give ORS fluids	|___|
	284	Give zinc treatment for 10 days	|___|
	285	Advise on when/how to administer	
	286	Advise mother to breastfeed / feed more frequently and longer	|___|
	287	Keep the child warm	|___|
	288	Advise mother when to return	|___|
	289	Follow up in 2 days	
	999	None mentioned from the list	
**What are the main signs and symptoms of malaria in a child aged 2-59 months?**<br><br>Do not prompt<br>Select all mentioned	**For each: 1 = Yes 2 = No**		
	290	Fever	|___|
	291	Chillis	|___|
**When the child presents signs and symptoms of malaria what initial steps do you take?**<br><br>Do not prompt<br>Select all mentioned	**For each: 1 = Yes 2 = No**		
	292	Use RDT to test for malaria	|___|
	293	Treat malaria with Coartem / ACT	|___|
	294	Advise on when/how to administer Coartem	
	295	Advise mother when to return	|___|
	999	None mentioned from the list	

For peer review only - http://bmjopen.bmj.com/site/about/guidelines.xhtml

**What are the main signs and symptoms of acute malnutrition in a child aged 2-59 months?**<br><br>Do not prompt<br>Select all mentioned	**For each: 1 = Yes 2 = No**		
	296	Pitting edema of both feet	|___|
	297	Visible severe wasting	|___|
	298	MUAC measurement <11cm (if 6 months or older)	|___|
	999	None mentioned from the list	
**When the child presents signs and symptoms of acute malnutrition what initial steps do you take?**<br><br>Do not prompt<br>Select all mentioned	**For each: 1 = Yes 2 = No**		
	299	Appetite test if 6 months or older	|___|
	300	Give with RUTF (Plumpy nut or BP 100)	|___|
	301	Advise on when/how to take RUTF	|___|
	302	Advise mother when to return	|___|
	999	None mentioned from the list	
**What are general danger signs in child aged 2-59 months?**<br><br>Do not prompt<br>Select all mentioned	**For each: 1 = Yes 2 = No**		
	303	Unable to drink or breastfeed	|___|
	304	Convulsions	|___|
	305	Movement only when stimulated or no movement even when stimulated	|___|
	999	None mentioned from the list	
**When the child presents with general danger signs and symptoms what initial steps do you take?**<br><br>Do not prompt<br>Select all mentioned	**For each: 1 = Yes 2 = No**		
	306	Refer to health centre	|___|
	307	Giver pre-refferal dose and refer to health center	|___|
	308	Give ORS	|___|
	999	None mentioned from the list	

For peer review

MODULE 4: TRAINING OF THE HEW

Interviewer*:* *I would now like to ask you some questions on your training*.

INTERVIEWER: PLEASE STATE THE START AND END MONTHS THAT CAPTURE THE PAST 12 MONTHS FROM THE DATE OF THE INTERVIEW AND INSERT IT INTO THE QUESTION BELOW.

Have you received training or training update from HC, Woreda health office or NGO in the last 12 months between ______________ (start month) and ______________ (end month) in?
[READ TOPIC]
FOR EACH QUESTION IF NO ASK THE FOLLOWING: *Did you receive a training or training update more than 12 months ago?*
REPEAT BOTH QUESTIONS FOR EACH TOPIC

		Yes, within past 12 months	Yes, over past 12 months ago	No in-service training or update	
309	CBNC	1	2	3	|___|

For peer review only - http://bmjopen.bmj.com/site/about/guidelines.xhtml

310	Providing antenatal services	**1**	**2**	**3**	|___|
311	PMTCT	**1**	**2**	**3**	|___|
312	Misoprostol use	**1**	**2**	**3**	|___|
313	Providing post-natal care to mother	**1**	**2**	**3**	|___|
314	Providing postnatal care to newborn	**1**	**2**	**3**	|___|
315	Clean cord care	**1**	**2**	**3**	|___|
316	Managing newborns with very severe disease	**1**	**2**	**3**	|___|
317	Managing newborn with local bacterial infection	**1**	**2**	**3**	|___|
318	Managing newborn neonates with jaundice/severe jaundice	**1**	**2**	**3**	|___|
319	Managing neonates with diarrhea	**1**	**2**	**3**	|___|
320	Managing neonates with feeding problem or who are underweight	**1**	**2**	**3**	|___|
321	iCCM	**1**	**2**	**3**	|___|
322	Using referral forms for VSD	**1**	**2**	**3**	|___|
323	Using/filling family folder	**1**	**2**	**3**	|___|
324	Integrated Refresher training on MNCH services	**1**	**2**	**3**	|___|
325	EPI	**1**	**2**	**3**	|___|
326	Can you tell us whether or not you were satisfied with the quality of training received for managing sick neonate?<br><br>**Do not give options to the respondent**	**1 = Yes was satisfied**<br>**2 = No was not satisfied – GO TO 328**<br>**3 = Neither satisfied nor dissatisfied – GO TO 329**<br>**4= No training in the last 12 months –GO TO 335**			|___|
327	**IF YES,** then what was the level of satisfaction<br><br>**Give both options to the respondent**	**1. Fully satisfied – GO TO 329**<br>**2. Somewhat satisfied – GO TO 329**			|___|
328	**IF NO,** then what was the level of dissatisfaction<br><br>**Give both options to the respondent**	**1. Fully dissatisfied**<br>**2. Somewhat dissatisfied**			|___|

How can the quality of the training be further improved **Read list. Select all that apply.**	**For each: 1 = Yes 2 = No**		
	329	More training	|___|
	330	More practice sessions	|___|
	331	More training aids	|___|
	332	More post training supervision	|___|
	333	Other – **GO TO 334**	|___|
	334	Specify___________________________	

For peer review only - http://bmjopen.bmj.com/site/about/guidelines.xhtml

MODULE 5: SUPPORTIVE SUPERVISION

INTERVIEWER:
I would now like to ask some questions about supportive supervision you have received. By supportive supervision I mean being visited by individuals from the region, zone, woredaand/or health center to discuss, review and give feedback on your TECHNICAL or PROFEESIONAL work.
INTERVIEWER: PLEASE STATE THE START AND END MONTHS THAT CAPTURE THE PAST 6 MONTHS FROM THE DATE OF THE INTERVIEW AND INSERT IT INTO THE QUESTION BELOW.

LAST 6 MONTHS: STATE THE START & END MONTHS
START MONTH ________________
END MONTH ________________

335	Have you received a supportive supervisory visit in the last 6 months? .	**1 = Yes**<br>**2 = No – GO TO 366**		|___|
**IF YES**: Who from?<br><br>**Select all that apply**		**For each: 1 = Yes 2 = No**		
		336	Woreda health office	|___|
		337	PHCU/health centre	|___|
		338	NGO	|___|
339	**IF YES:** How many times did you receive this visit in the last 6 months?	**Enter total number of times**<br>**IF 0 skip to 342**		|___|
340	How many of these visits were in last 3 months?	**Enter total number of times**<br>**IF 0 skip to 342**		|___|
341	How many of these visits were in last 1 month?	**Enter total number of times**		|___|
342	Who provided the most recent supervisory visit?<br><br>**Select one**	**1 = Woreda Health Office**<br>**2 = Health Centre**<br>**3 = NGO**<br>**4 = Woreda Health Office and Health center**<br>**5 = Woreda Health Office and NGO**<br>**6 = Health Center and NGO**<br>**7 = All three together (Woreda, Health Center, NGO)**		|___|
**If YES to 335**: Did that visit include any of the following?<br><br>**Read all the following**		**For each: 1 = Yes 2 = No**		
		343	Discussing early identification of pregnancy	|___|
		344	Discussing provision focused ANC	|___|
		345	Discussing promotion of institutional delivery	|___|
		346	Discussing safe and clean delivery	|___|
		347	Discussing immediate newborn care including cord care (chlorohexidine)	|___|
		348	Discussing recognition of asphyxia, initial stimulation, and resuscitation of newborn babies	|___|
		349	Discussing prevention and management of hypothermia	|___|
		350	Discussing management of pre-term and/or low	|___|

For peer review only - http://bmjopen.bmj.com/site/about/guidelines.xhtml

			birth weight neonates	
		351	Discussing management of very severe disease in newborns	|____|
		352	Discussing diagnosis or treatment of suspected pneumonia	|___|
		353	Discussing diagnosis or treatment of diarrheoa	|___|
		354	Discussing diagnosis or treatment of malaria	|___|
		355	Discussing diagnosis or treatment of acute malnutrition	|____|
		356	Discussing HEW activities with WDA	|____|
		357	Observing record keeping	|____|
		358	Checking the register for consistency and completeness	|____|
		359	Checking supplies including training manuals, job aides, request forms	|____|
		360	Delivering supplies including /training manuals, job aides, request forms	|____|
		361	Observing client Consultation with HEW	|____|
		362	Conducted postnatal household visits together to observe HEWs skill on checking general danger signs	|____|
		363	Checking if they visited a sick neonate under treatment or that has been treated,	|____|
		364	Providing WRTTTEN feedback to you on your work	|____|
		365	WRTTTEN feedback: copy of the last visit available and checked by the interviewer	|____|
366	Did you receive a follow up visit within 6 weeks of CBNC training to assess and support your **CBNC work**?	**1 = Yes**<br>**2 = No – GO TO 371**<br>**3 = No CBNC training –GO TO 371**		|____|
If received CBNC post-training visit who conducted 6 weeks follow up visit?<br><br>**Select all that apply**		**For each: 1 = Yes 2 = No**		
		367	Zone	|____|
		368	Woreda	|____|
		369	Health Center	|____|
		370	NGO	|___|

For peer review only - http://bmjopen.bmj.com/site/about/guidelines.xhtml

371	Can you tell us whether or not you were satisfied with the QUALITY of supportive supervision received in last six months?<br><br>**<u>Do not</u> read options**	**1 = Yes was satisfied**<br>**2 = No was not satisfied – GO TO 373**<br>**3 = Neither satisfied nor dissatisfied – GO TO 374**<br>**4 = no supportive supervision in the last 6 months GO TO 379**	|___|
372	**IF YES,** then what was the level of satisfaction?<br><br>**<u>Read</u> options**	**1. Fully satisfied -GO TO 379**<br>**2. Somewhat satisfied -GO TO 374**	|___|
373	**IF NO,** then what was the level of dissatisfaction?<br><br>**<u>Read</u> options**	**1. Fully dissatisfied**<br>**2. Somewhat dissatisfied**	|___|

How can the quality of the supervision be further improved:<br><br>**Read list**<br>**Select all that apply**	**For each: 1 = Yes 2 = No**		
	374	More visits	|____|
	375	More crash trainings during supervision	|____|
	376	More technical supervision	|____|
	377	Other **can the quality of the supervision be further improved**	|____|
	378	Specify ___________________	|____|

For peer review only

Interviewer:
I would now like to ask you some questions about Performance Review and Clinical Mentoring Meeting (PRCMM) By this I mean when NGO, health center and health post staff meet together to review records, discuss performance, and ways to improve your TECHNICAL skills and achieve targets for CBNC/ICCM?
INTERVIEWER: PLEASE STATE THE START AND END MONTHS THAT CAPTURE THE PAST 6 MONTHS FROM THE DATE OF THE INTERVIEW AND INSERT IT INTO THE QUESTION BELOW.

LAST 6 MONTHS: STATE THE START & END MONTHS
START MONTH ________________
END MONTH ________________

**379**	In the past 6 months, did you participate in a PRCMM meeting, where the health center, health post and/or NGO staff met together to discuss performance, targets, and ways to improve HEWs' skills and achieve targets for CBNC and/or ICCM?	**1 = Yes**<br>**2 = No**<br>**3 = No CBNC and/or ICCM training**	|___|
**380**	Since training of CBNC and/or ICCM, have you participated in any PRCM meeting conducted in your Health center catchment area?	**1 = Yes**<br>**2 = No – GO TO MODULE 6**	|___|
**381**	Did the meeting extract data from the HEW's 0-2 month (newborn) registers?	**1 = Yes**<br>**2 = No**	|___|
**382**	At that meeting, did your health center staff get a chance to offer mentoring on how to improve your newborn illness management skills?	**1 = Yes**<br>**2 = No**	|___|

Did that meeting cover discussions on performance and targets on the following?<br><br>**Read all the following**		**For each: 1 = Yes 2 = No**	
	383	Early identification of pregnancy	|___|
	384	Focused ANC	|___|
	385	Promotion of institutional delivery	|___|
	386	Safe and clean delivery	|___|
	387	Immediate newborn care including cord care (chlorohexidine)	|___|
	388	Recognition of asphyxia, initial stimulation and resuscitation of newborn babies	|___|
	389	Management of diarrhea among neonate	|___|
	390	Breast feeding among neonate	|___|
	391	Immunization among neonate	|___|
	392	Management of hypothermia	|___|
	393	Management of pre-term and/or low birth weight neonates	|___|
	394	Management of neonatal/very severe disease	|___|
	395	Register review	|___|
	396	Community level observation	|___|

For peer review only - http://bmjopen.bmj.com/site/about/guidelines.xhtml

MODULE 6: HEWS SERVICES PROVIDED IN THE LAST 3 MONTHS

Interviewer: ***I would now like to ask you about the services you provided in the last 3 months.***

REFER TO HEW'S RECORD BOOKS (AT THE HEALTH POST REGISTERS) TO COMPLETE THE FOLLOWING; <u>ONLY COUNT EVENTS ATTENDED BY THE SPECIFIC HEW BEING INTERVIEWED</u>:

INTERVIEWER: PLEASE STATE THE START AND END MONTHS THAT CAPTURE THE PAST 3 MONTHS FROM THE DATE OF THE INTERVIEW AND INSERT IT INTO THE QUESTION BELOW.
<u>LAST 3 MONTHS: STATE THE START & END MONTHS</u>
START MONTH ________________
END MONTH ________________

397	In the last three months did you use community forums and meeting to deliver maternal newborn and child health education?	**1 = Yes**<br>**2 = No – GO TO 406**	I___I

In the last three months did you meet any of the following to deliver maternal newborn and child health education?<br><br>**Read list. Choose all that apply.**	**For each: 1 = Yes 2 = No**		
	398	Religious leaders	I___I
	399	Edir (Traditional community organization whose members assist each other during the mourning process)	I___I
	400	Women's savings group	I___I
	401	Command post	I___I
	402	Traditional birth attendants	I___I
	403	Other	I___I
	404	Other Specify______________________	

405	When was the last time you used community forum and meeting to deliver maternal newborn and child health education?	I__II__I/I__II__I/I__II__I<br>dd / mm / yy	
406	Did you conduct pregnant women conference in the community in the last 3 months?	**1 = Yes**<br>**2 = No – GO TO 411**	I___I
407	When was the last time you conducted a pregnant women's conference?	I__II__I/I__II__I/I__II__I<br>dd / mm / yy	
408	How regularly do you conduct the pregnant women's conference?	**1 = Once a week**<br>**2 = Every two weeks**<br>**3 = Once a month**<br>**4 = Every other month**<br>**5 = Once every three months**	I___I

For peer review only

For peer review only - http://bmjopen.bmj.com/site/about/guidelines.xhtml

409	In the last pregnant women conference, how many women in your catchment area were pregnant?	**Enter number**<br>**Enter 999 if don't know**	|__|__|__|
410	Among them, how many of them attended the pregnant women's conference?<br>(The number should be ≤ numbers inserted in Q 408)	**Enter number**	|___|___|
411	Did you provide ANC to any women in the last three months?	**1 = Yes**<br>**2 = No – GO TO 412**	|___|
412	**IF YES:** how many?	**Enter number**	|___||___|
413	Did you refer any pregnant women from this health post to a health center or hospital in the last three months?	**1 = Yes**<br>**2 = No – GO TO 414**	|___|
414	**IF YES:** how many?	**Enter number**	|___||___|
415	Did you see any women to provide postpartum care in the last three months?	**1 = Yes**<br>**2 = No – GO TO 416**	|___|
416	**IF YES:** How many women did you see for postpartum care in the last three months?	**Enter number**	|___||___|
417	Did you refer any post-partum women from this health post to a health center or hospital in the past three months?	**1 = Yes**<br>**2 = No – GO TO 418**	|___|
418	**IF YES:** How many?	**Enter number**	|___||___|
419	Did you see any newborns to provide a postnatal check for in the last three months?	**1 = Yes**<br>**2 = No – GO TO 420**	|___|
420	**IF YES:** How many newborns did you provide a postnatal check for in the last three months?	**Enter number**	|___||___|
421	Did you refer any newborns from this health post to a health center or hospital in the past three months?	**1 = Yes**<br>**2 = No – GO TO 422**	|___|
422	**IF YES:** How many?	**Enter number**	|___||___|
423	Did you give care for prevention of hypothermia in the last three months?	**1 = Yes**<br>**2 = No – GO TO 424**	|___|
424	**IF YES**: How many?	**Enter number**	|___||___|
425	Did you give care for management of hypothermia in the last three months?	**1 = Yes**<br>**2 = No – GO TO 426**	|___|
426	**IF YES:** How many?	**Enter number**	|___||___|
427	Did you provide care for pre-term and/or low birth weight neonates in the last three months?	**1 = Yes**<br>**2 = No – GO TO 428**	|___|
428	**IF YES**: How many?	**Enter number**	|___|
429	Did you identify newborns with suspected very severe disease in the	**1 = Yes**<br>**2 = No – GO TO 430**	|___|

For peer review only - http://bmjopen.bmj.com/site/about/guidelines.xhtml

	last three months?		
430	**IF YES**: How many?	**Enter number**	|___|
431	Did you treat newborns with suspected very severe disease in the last three months?	**1 = Yes**<br>**2 = No – GO TO 433**<br>**3 = Antibiotics not available – GO TO 433**	|___|
432	**IF YES**: how many?	**Enter number**	|___|
433	**IF YES**: how many completed the treatment at the health post?	**Enter number**	|___|
434	Did you refer any newborns from this health post to a health center or hospital for very severe disease in the past three months?	**1 = Yes**<br>**2 = No – GO TO 435**	|___|
435	**IF YES**: How many?	**Enter number**	|___|
436	Did you see any newborns with diarrhea in the last three months?	**1 = Yes**<br>**2 = No – GO TO 437**	|___|
437	**IF YES**, how many?	**Enter number**	|___||___|
438	Did you see any newborns with jaundice in the last three months?	**1 = Yes**<br>**2 = No – GO TO 439**	|___|
439	**IF YES**, how many?	**Enter number**	|___||___|
440	Are the maternity record books completely up to date until the day before survey?(observe)	**1 = Yes**<br>**2 = No GO TO 442**	|___|
441	What is number of maternity cases maintained in the last three months in the maternity register?	**Enter number**	|___||___|
442	Did you see any children (2-59 months) for suspected pneumonia in the last three months?	**1 = Yes**<br>**2 = No – GO TO 443**	|___|
443	**IF YES**, how many?	**Enter number**	|___||___|
444	Did you see any children (2-59 months) for diarrhea in the last three months?	**1 = Yes**<br>**2 = No – GO TO 445**	|___|
445	**IF YES**, how many?	**Enter number**	|___||___|
446	Did you see any children (2-59 months) for malaria in the last three months?	**1 = Yes**<br>**2 = No – GO TO 447**	|___|
447	**IF YES**, how many?	**Enter number**	|___||___|
448	Did you see any children (2-59 months) for acute malnutrition in the last three months?	**1 = Yes**<br>**2 = No – GO TO 449**	|___|
449	**IF YES**, how many?	**Enter number**	|___||___|
450	Did you refer any children (2-59 months) from this health post to a health center or hospital for very severe disease in the past three months?	**1 = Yes**<br>**2 = No – Exit interview**	|___|
451	**IF YES**: How many?	**Enter number**	|___||___|

For peer review only

STROBE 2007 (v4) Statement—Checklist of items that should be included in reports of *cross-sectional studies*

Section/Topic	Item #	Recommendation	Reported on page #
**Title and abstract**	1	(*a*) Indicate the study's design with a commonly used term in the title or the abstract	1
		(*b*) Provide in the abstract an informative and balanced summary of what was done and what was found	2
**Introduction**			
Background/rationale	2	Explain the scientific background and rationale for the investigation being reported	3-4
Objectives	3	State specific objectives, including any prespecified hypotheses	4
**Methods**			
Study design	4	Present key elements of study design early in the paper	5
Setting	5	Describe the setting, locations, and relevant dates, including periods of recruitment, exposure, follow-up, and data collection	4-5
Participants	6	(*a*) Give the eligibility criteria, and the sources and methods of selection of participants	5
Variables	7	Clearly define all outcomes, exposures, predictors, potential confounders, and effect modifiers. Give diagnostic criteria, if applicable	6-7
Data sources/ measurement	8*	For each variable of interest, give sources of data and details of methods of assessment (measurement). Describe comparability of assessment methods if there is more than one group	6-7
Bias	9	Describe any efforts to address potential sources of bias	6-7
Study size	10	Explain how the study size was arrived at	5
Quantitative variables	11	Explain how quantitative variables were handled in the analyses. If applicable, describe which groupings were chosen and why	6–7
Statistical methods	12	(*a*) Describe all statistical methods, including those used to control for confounding	7
		(*b*) Describe any methods used to examine subgroups and interactions	7
		(*c*) Explain how missing data were addressed	7
		(*d*) If applicable, describe analytical methods taking account of sampling strategy	7
		(*e*) Describe any sensitivity analyses	-
**Results**			

For peer review only

Participants	13*	(a) Report numbers of individuals at each stage of study—eg numbers potentially eligible, examined for eligibility, confirmed eligible, included in the study, completing follow-up, and analysed	8
		(b) Give reasons for non-participation at each stage	-
		(c) Consider use of a flow diagram	-
Descriptive data	14*	(a) Give characteristics of study participants (eg demographic, clinical, social) and information on exposures and potential confounders	8-9
		(b) Indicate number of participants with missing data for each variable of interest	8, 11, 13, 14
Outcome data	15*	Report numbers of outcome events or summary measures	11
Main results	16	(*a*) Give unadjusted estimates and, if applicable, confounder-adjusted estimates and their precision (eg, 95% confidence interval). Make clear which confounders were adjusted for and why they were included	13-14
		(*b*) Report category boundaries when continuous variables were categorized	9
		(*c*) If relevant, consider translating estimates of relative risk into absolute risk for a meaningful time period	N/A
Other analyses	17	Report other analyses done—eg analyses of subgroups and interactions, and sensitivity analyses	12
**Discussion**			
Key results	18	Summarise key results with reference to study objectives	15
Limitations	19	Discuss limitations of the study, taking into account sources of potential bias or imprecision. Discuss both direction and magnitude of any potential bias	15
Interpretation	20	Give a cautious overall interpretation of results considering objectives, limitations, multiplicity of analyses, results from similar studies, and other relevant evidence	15 - 17
Generalisability	21	Discuss the generalisability (external validity) of the study results	15
**Other information**			
Funding	22	Give the source of funding and the role of the funders for the present study and, if applicable, for the original study on which the present article is based	18

*Give information separately for cases and controls in case-control studies and, if applicable, for exposed and unexposed groups in cohort and cross-sectional studies.

Note: An Explanation and Elaboration article discusses each checklist item and gives methodological background and published examples of transparent reporting. The STROBE checklist is best used in conjunction with this article (freely available on the Web sites of PLoS Medicine at http://www.plosmedicine.org/, Annals of Internal Medicine at http://www.annals.org/, and Epidemiology at http://www.epidem.com/). Information on the STROBE Initiative is available at www.strobe-statement.org.

For peer review only
